# Intramolecular Halo Stabilization of Silyl Cations—Silylated Halonium‐ and Bis‐Halo‐Substituted Siliconium Borates

**DOI:** 10.1002/chem.202004838

**Published:** 2021-01-18

**Authors:** Anastasia Merk, Lukas Bührmann, Natalie Kordts, Katharina Görtemaker, Marc Schmidtmann, Thomas Müller

**Affiliations:** ^1^ Institute of Chemistry Carl von Ossietzky University Oldenburg Carl von Ossietzky-Str. 9–11 26129 Oldenburg Germany, European Union

**Keywords:** hypercoordination, Lewis acidity, neighboring effect, silicon, silicon cations

## Abstract

The stabilizing neighboring effect of halo substituents on silyl cations was tested for a series of *peri*‐halo substituted acenaphthyl‐based silyl cations **3**. The chloro‐ (**3 b**), bromo‐ (**3 c**), and iodo‐ (**3 d**) stabilized cations were synthesized by the Corey protocol. Structural and NMR spectroscopic investigations for cations **3 b**–**d** supported by the results of density functional calculations, which indicate their halonium ion nature. According to the fluorobenzonitrile (FBN) method, the silyl Lewis acidity decreases along the series of halonium ions **3**, the fluoronium ion **3 a** being a very strong and the iodonium ion **3 d** a moderate Lewis acid. Halonium ions **3 b** and **3 c** react with starting silanes in a substituent redistribution reaction and form siliconium ions **4 b** and **4 c**. The structure of siliconium borate **4 c**_2_[B_12_Br_12_] reveals the trigonal bipyramidal coordination environment of the silicon atom with the two bromo substituents in the apical positions.

## Graphical abstract

**Silyl cation or halonium ion?** That′s the question: The Corey reaction of acenaphthyl‐ and naphthyl‐based silanes allows the synthesis of silyl halonium ions, the nature of which was confirmed by NMR spectroscopy, X‐ray diffraction analysis, and DFT calculations. Moreover, the chloro‐ and bromonium ion can react with the starting silane to form penta‐coordinated siliconium ions, which are intramolecularly stabilized by electron donation from both halogen atoms.

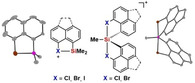

## Introduction

**Figure 1:**
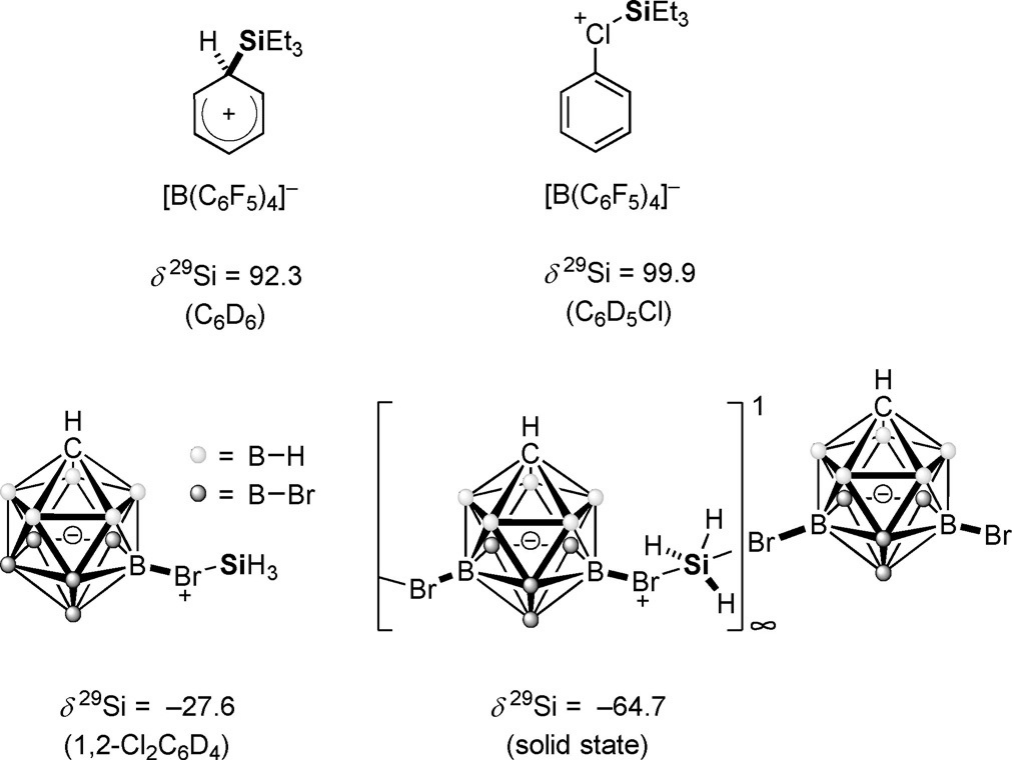
^29^Si NMR chemical shifts of triethylsilyl cation in different solvents and of silyl cation in solution and the solid state.[^2b^, ^6^]

**Figure 2:**
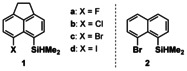
Acenaphthyl silanes **1** and naphthyl silane **2**.

Recent years have witnessed the transformation of silyl cations from laboratory curiosities to valuable reagents and catalysts in synthetic organic chemistry.^[1]^ These positively charged organosilicon compounds are of interest owing to their high electrophilicity and Lewis acidity, properties that severely hampered their isolation and application.^[2]^ Significant progress in the preparation methods for silyl cations was needed before their synthetic potential could be exploited. In particular, the quest for non‐coordinating reaction conditions has been omnipresent in silyl cation chemistry. This was guaranteed by the use of weakly coordinating anions (nearly exclusively halogenated tetraarylborates, aluminates, boranes, and carboranes) and solvents such as arenes and halogenated arenes.^[3]^ The halo substitution effect is of significant importance for the stability of the anions as it increases their oxidation potential and lowers their nucleophilicity. Halogenated arenes as solvents differ in their reactivity versus silyl cations from aromatic hydrocarbons in so far as the most nucleophilic side is the halo substituent and not the aromatic π‐system. This difference is clear from the recorded ^29^Si NMR chemical shifts^[2b]^ (Figure 1), it is manifested in solid‐state structures of the corresponding salts of the silyl cation solvent complexes^[4]^ and leads to distinctly different reactivity of the cation, for example, versus carbon dioxide.^[5]^ The recent synthesis of the parent silylium ion [SiH_3_]^+^ by the Oestreich group highlights the role of halogenated counter anions and halogenated arenes as solvents.^[6]^ In *o*‐dichlorobenzene solution, [SiH_3_]^+^ exists as a close ion pair with the brominated carborane anion with the silicon cation coordinated via a bromine atom to the anion (Figure 1). The solid‐state structure of the silylium carborane revealed a one‐dimensional polymeric structure with a trigonal‐bipyramidal‐coordinated silicon atom with two bromine substituents from two different carborane anions in the apical positions (Figure 1). In addition, the interaction between halo substituents and silyl cations is the first step of each C−F or C−Cl activation by silyl cations or of the decomposition of silyl cations in halogenated solvents.[^2b^, ^7^] Bis‐silylated halonium ions have been studied by our group^[8]^ and, more extensively, by the groups of Schulz and Wagner.^[9]^ The interaction between aryl halides and silyl cations has not been studied systematically. In view of the importance of halo stabilization in silyl cation chemistry, we included these substituents in our general study on neighboring effects on the properties of silylium ions.^[10]^ We synthesized the acenaphthyl silanes **1** and naphthyl silane **2** and studied their reactivity versus trityl cation (Figure 2).

## Results and Discussion

**Figure 3:**
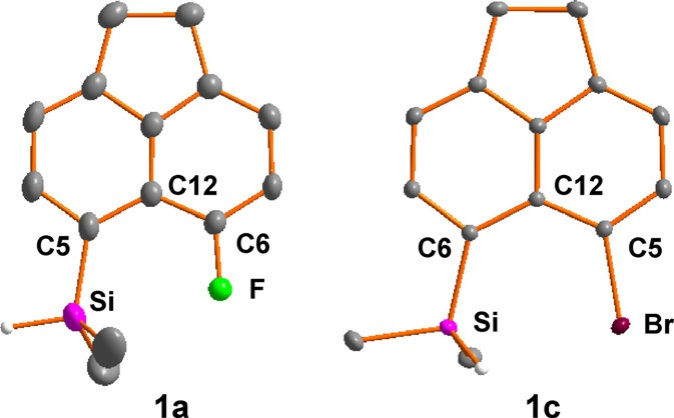
Molecular structure of silanes **1 a, c** in the crystal (thermal ellipsoids are shown at the 50 % probability level, hydrogen atoms are omitted for clarity; only the hydrogen atom attached to silicon is shown). Pertinent bond lengths [pm] and bond angles [°]: **1 a**: F−C^6^ 136.22(16), Si−C^5^ 186.83(16), F/Si 300.87(10), Si−H 138.(3), F‐C^6^‐C^12^ 117.89(12), Si‐C^5^‐C^12^ 126.81(11), C^5^‐C^12^‐C^6^ 128.40(13), Σ*β* 373.1, Σ*α*(SiC_3_) 336.7, F‐Si‐H 177.9; **1 c**: Br−C^5^ 190.28(6), Si−C^6^ 188.79(6), Si−H 140.0(12), Br‐C^5^‐C^12^ 122.89(4), Si‐C^6^‐C^12^ 128.69(4), C^5^‐C^12^‐C^6^ 130.14(5), Σ*β* 381.7, Σ*α*(SiC_3_) 327.3 H/Br 292.1.

*peri*‐Halo‐silyl‐substituted acenaphthenes **1** and the 8‐silyl‐substituted bromonaphthalene **2** were synthesized according to established protocols and were fully characterized by standard analytical techniques especially multinuclear NMR spectroscopy (see the Supporting Information). The molecular structures of silanes **1 a** and **1 c** were obtained from X‐ray diffraction analysis of suitable single crystals. Their molecular structures in the crystal are inconspicuous (Figure 3). Remarkable is that the fluoro‐substituted acenaphthyl silane **1 a** shows the on‐set of an intramolecular Si/F interaction.^[11]^ This is indicated by an almost linear alignment of the F/Si−H atoms (177.9°) and a Si/F separation of 300.9 pm, which is significantly shorter than the sum of the van der Waals radii (357 pm).^[12]^ In addition, a through‐space coupling of ^TS^ *J*=5.7 Hz was detected in both ^29^Si NMR (*δ* ^29^Si=12.4) and ^19^F NMR spectra (*δ* ^19^F=−119.4).^[13]^ The bromo‐substituted acenaphthyl silane **1 c** adopts a conformation that brings the Si−H into a *syn*‐arrangement with the bromine atom. The H/Br separation is 292 pm, almost exactly the expectation value from the sum of the van der Waals radii (293 pm).^[12]^ The sum of the bond angles Σ*β* involving the atoms defining the bay region of silane **1 c** (Si, C^6^, C^12^, C^5^, Br) is 381.7°, which is larger than the value reported for acenaphthene (Σ*β*=368°).^[14]^ This indicates repulsion between the silyl and the bromo substituent in silane **1 c**.^[15]^

**Scheme 1:**
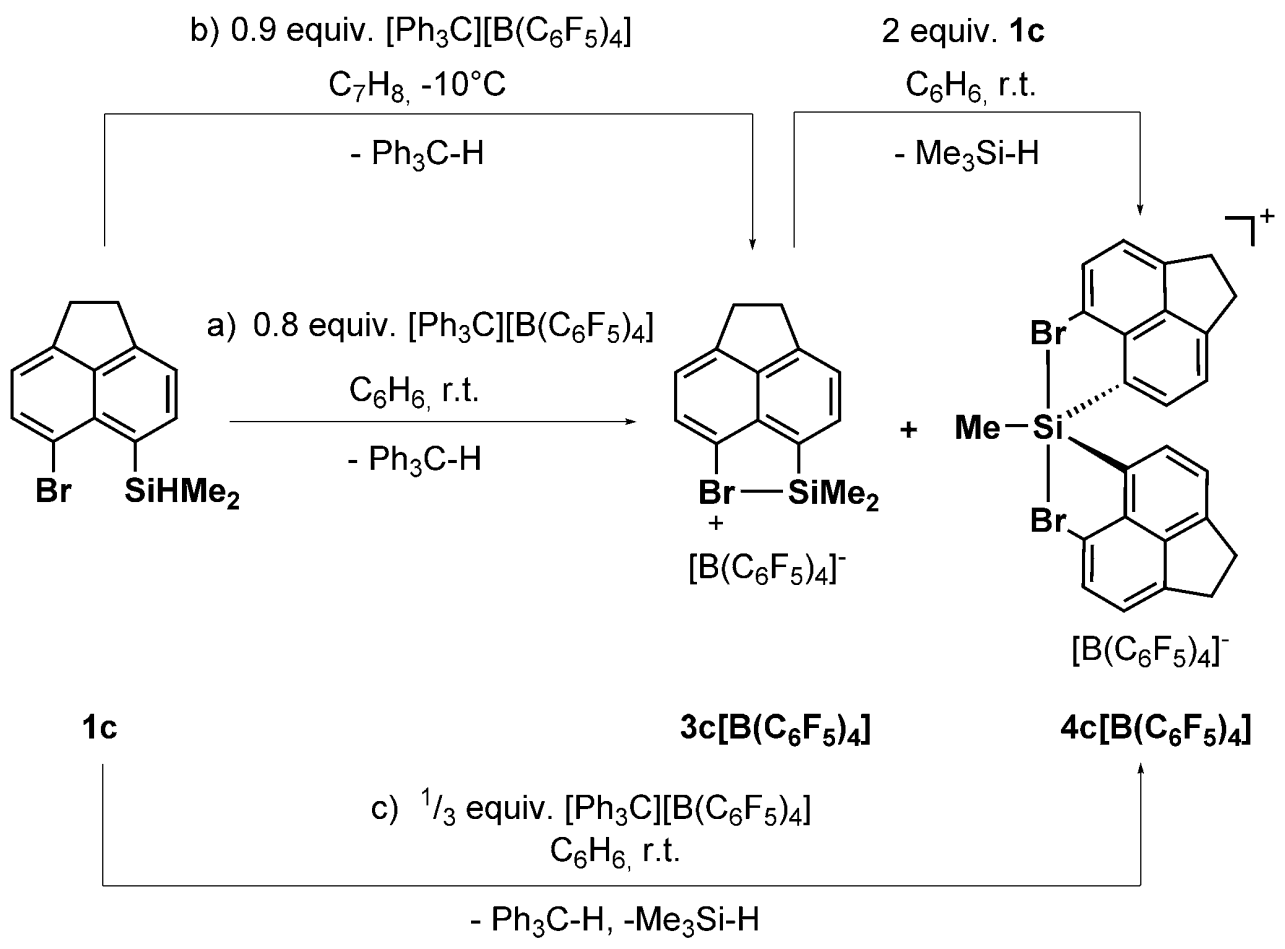
Synthesis of bromonium borate **3 c**[B(C_6_F_5_)_4_] and of siliconium borate **4 c**[B(C_6_F_5_)_4_] from acenaphthyl silane **1 c** applying the Corey protocol.

**Figure 4:**
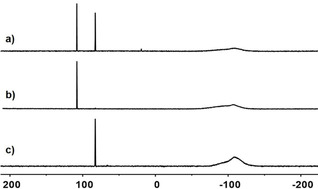
^29^Si{^1^H} NMR spectra of (a) the reaction of silane **1 c** with 0.8 equivalents [Ph_3_C][B(C_6_F_5_)_4_] at room temperature, in [D_6_]benzene; (b) 0.9 equivalents [Ph_3_C][B(C_6_F_5_)_4_] at −10 °C, in [D_8_]toluene; (c) 1/3 equivalents [Ph_3_C][B(C_6_F_5_)_4_] at room temperature, in [D_6_]benzene (broad signal around *δ* ^29^Si=−110 is due to borosilicate glass of the NMR tube and the probe head inlet).

**Figure 5:**
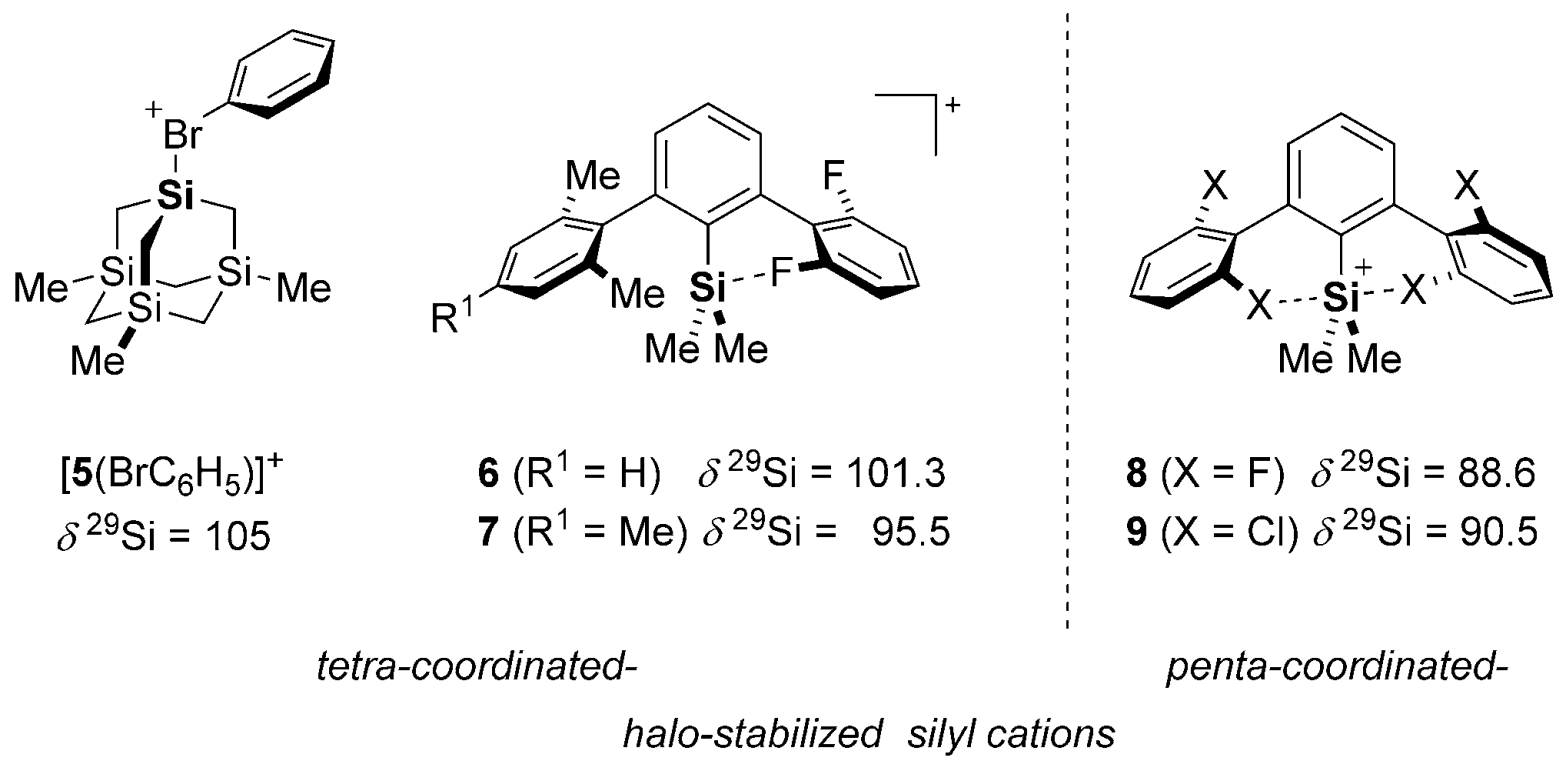
Examples of intermolecular ([**5**(BrC_6_H_5_)]^+^) and intramolecular halo stabilized silyl cations **6**–**9** with different coordination environments and their ^29^Si NMR chemical shifts.

Next, we attempted the synthesis of cyclic halonium borates **3**[B(C_6_F_5_)_4_] by using the standard Corey protocol (Scheme 1).^[16]^ Surprisingly, the reaction of silane **1 c** with [Ph_3_C][B(C_6_F_5_)_4_] at room temperature gave two silicon‐containing products, which are identified by ^29^Si NMR chemical shifts of *δ* ^29^Si=107.9 and *δ* ^29^Si=82.6 in a ratio of 65:35 (Scheme 1, path a), Figure 4 a). The trityl cation was completely consumed during the reaction. These low field shifted ^29^Si NMR signals indicated the formation of two silicon cationic species, which suggested that one product is formed in a consecutive reaction. To test this hypothesis, we run the reaction at *T*=−10 °C in toluene and observed indeed the selective formation of the compound with *δ* ^29^Si=107.5 (Scheme 1, path b, Figure 4 b). This ^29^Si NMR chemical shift is indicative of an intramolecular bromo‐stabilized silyl cation. The recorded ^29^Si NMR chemical shift is close to that reported for the silylated chloronium ion [Et_3_Si(Cl‐C_6_H_5_)]^+^ (*δ* ^29^Si=99.9)^[5]^ and in the range of related tetra‐coordinated halo‐stabilized silyl cations **5**–**7** (Figure 5).^[17]^ The detailed analysis of the spectroscopic NMR data confirmed the expected formation of the silylated bromonium ion **3 c** (see the Supporting Information). This finding was supported by the results of NMR chemical shift calculations at the GIAO/M06L/def2tzvp//M06‐2X/def2tzvp level of theory, which predict for an optimized molecular structure of cation **3 c**, a ^29^Si NMR chemical shift of *δ* ^29^Si(calc)=109.^[18]^ The second species with a ^29^Si NMR chemical shift of *δ* ^29^Si=82.6 could be selectively synthesized by adding additional silane **1 c** at room temperature to a toluene solution of **3 c**[B(C_6_F_5_)_4_]. Although for a stoichiometric conversion two equivalents of **1 c** are needed in theory, the reaction was more selective when a threefold excess of the starting silane **1 c** at room temperature was reacted with the trityl borate (Scheme 1, path c, Figure 4 c). The NMR spectroscopic data indicate the substitution of the cationic silicon atom with only one methyl group but with two acenaphthyl residues. Supportive of the structure elucidation of this cation was the detection of relatively large ^1^ *J*(SiC) couplings of ^1^ *J*(SiC(aryl))=82 Hz and ^1^ *J*(SiC(alkyl))=62 Hz compared with those found for cation **3 c** (^1^ *J*(SiC(aryl))=76 Hz and ^1^ *J*(SiC(alkyl))=54 Hz). These large ^1^ *J*(SiC) coupling constants indicate a higher contribution of atomic s orbitals to the Si−C bonds and suggest penta‐coordination for the cationic silicon atom with the carbon substituents at the trigonal base of the bipyramid. On the basis of the NMR data, we assign the siliconium ion structure **4 c** to the observed species. No significant change was observed by ^1^H NMR spectroscopy at *T*=−40 °C, which makes a dynamic equilibrium between two tetra‐coordinated species less likely. In favor of this claim are the ^29^Si NMR chemical shift calculations for an optimized molecular structure of siliconium ion **4 c**, which predict a ^29^Si NMR chemical shift of *δ* ^29^Si(calc)=85. In addition, the ^29^Si NMR data is in agreement with that reported for related terphenyl‐based siliconium ions **8** and **9** (Figure 5).^[19]^ Finally, the structure of siliconium ion **4 c** was determined by X‐ray diffraction (XRD) analysis from suitable single crystals of **4 c**_2_[B_12_Br_12_]. The electrophilicity of halonium ion **3 c** is high and it reacts with excess of the starting silane **1 c** to undergo a substituent redistribution reaction. These reactions between silyl cations and silanes are well documented for aryl‐ and alkyl‐substituted silylium ions.[^8^, ^20^] We were not able to detect the volatile trimethylsilane but tetramethylsilane, a common follow‐up product of further ligand exchange, was detected by NMR spectroscopy.^[21]^

**Scheme 2:**
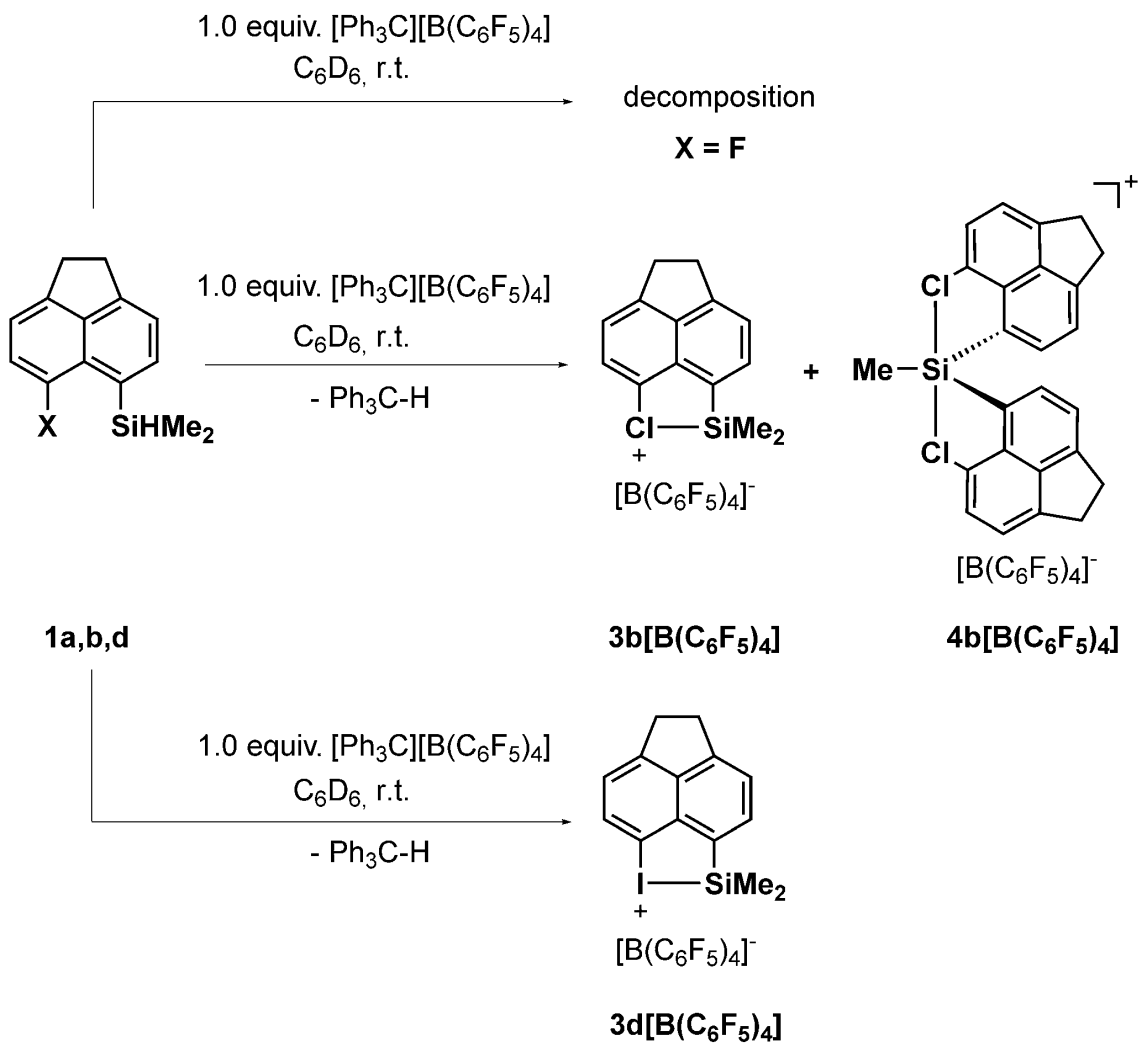
Reaction of silane **1 a**, **b**, **d** with trityl borate according to the Corey protocol (**1 a**: X=F, **1 b**: X=Cl, **1 d**: X=I).

The results of the Corey reaction of the other halo acenaphthyl silanes **1 a**, **b**, **d** depend on the halo substituent (Scheme 2). The chloro acenaphthyl silane **1 b** gave at ambient conditions in benzene a 40:60 mixture of the chloronium borate **3 b**[B(C_6_F_5_)_4_] (*δ* ^29^Si=116.2) and the siliconium borate **4 b**[B(C_6_F_5_)_4_] (*δ* ^29^Si=79.8). The assignments are supported by the results of ^29^Si NMR chemical shift calculations for **3 b** (*δ* ^29^Si(calc)=117) and **4 b** (*δ* ^29^Si(calc)=79). The ratio **3 b**/**4 b** changed to 81:19 at *T*=−40 °C in chlorobenzene as the solvent. The selective formation of chloronium borate **3 b** was, however, not possible. Both silyl cations were identified in the mixture by their characteristic NMR parameters (see the Supporting Information). In contrast, iodonium borate **3 d**[B(C_6_F_5_)_4_] is formed selectively and was characterized by NMR spectroscopy (Scheme 2 and the Supporting Information). Most significant is the ^29^Si NMR chemical shift of *δ* ^29^Si=89.5, which agrees with the theoretically predicted value of *δ* ^29^Si(calc)=101. Finally, the reaction of the fluoro‐substituted acenaphthyl silane **1 a** with [Ph_3_C][B(C_6_F_5_)_4_] in aromatic hydrocarbons gave according to ^29^Si NMR spectroscopy an intractable mixture of compounds, even at *T*=−40 °C in chlorobenzene and at *T*=−80 °C in methylene dichloride. This observation suggests uncontrolled C−F activation through the incipient silyl cation (Scheme 2).

**Scheme 3:**
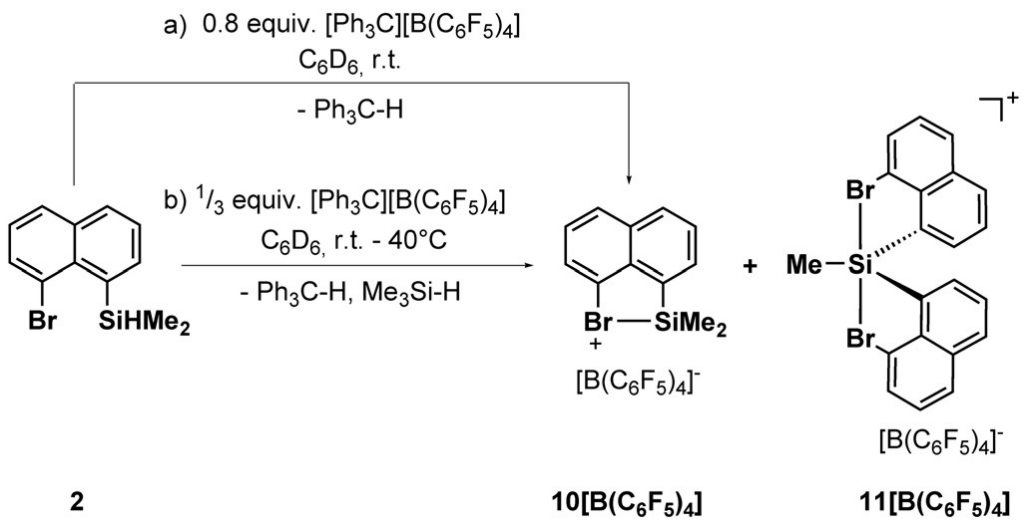
Synthesis of bromonium borate **10**[B(C_6_F_5_)_4_] and of siliconium borate **11**[B(C_6_F_5_)_4_] from naphthyl silane **2** by applying the Corey protocol.

The influence of the carbon skeleton on the formation of the halonium versus siliconium ion was tested with the bromonaphthyl silane **2**. In this case, we expected a more efficient stabilization of the silyl cation by the neighboring halo substituents.^[22]^ The standard Corey conditions (Scheme 3, path a) allowed the synthesis of the bromonium borate **10**[B(C_6_F_5_)_4_] (*δ* ^29^Si=94.6, *δ* ^29^Si(calc)=98). The use of three equivalents of silane **2** (Scheme 3, path b) did not result in the expected selective formation of siliconium borate **11**[B(C_6_F_5_)_4_] but gave even at higher reaction temperatures a mixture (25:75) of the cations **10** and **11**. A noticeable decomposition of both cations took place at temperatures above *T*=40 °C and prevented further attempts to drive the reaction to completeness. The experimental NMR data suggest for the silicon atom in cation **11** (*δ* ^29^Si=62.9) a trigonal bipyramidal coordination environment with one methyl group and two naphthyl substituents. NMR chemical shift computations for an optimized molecular structure of cation **11** support this assignment (*δ* ^29^Si(calc)=61). All prepared halonium ions **3** and **10** are very reactive in solution and decompose upon standing into non‐identified products over days. The siliconium ions **4** and **11** are more stable in solution and in the solid state but are still highly reactive with nucleophiles.

**Figure 6:**
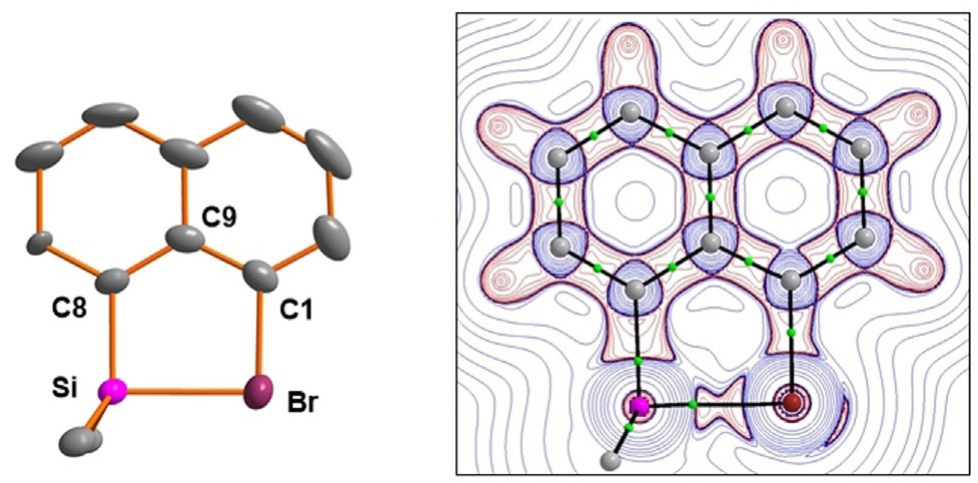
Left: Molecular structure of bromonium ion **10** in the crystal of [**10**][B(C_6_F_5_)_4_]**⋅**Li[B(C_6_F_5_)_4_]**⋅**0.5 C_6_H_6_ (thermal ellipsoids are shown at the 50 % probability level, hydrogen atoms are omitted for clarity). Pertinent bond lengths [pm] and bond angles [°]: Br−C^1^ 193.1(3), Si−C^8^ 183.8(3), Si−Br 242.52(8), C^1^‐Br‐Si 91.1(9), Br‐C^1^‐C^9^ 115.4(2), Si‐C^8^‐C^9^ 120.0(2), C^1^‐C^9^‐C^8^ 123.0(3), Σ*α*(SiC_3_) 349.5, sum of bay angles Σ*β* 358.5. Right: 2D contour plots of the calculated Laplacian of the electron density, ∇^2^ *ρ*(*r*), in the Si‐C^1^‐Br plane of cation **10**. The molecular graph of the cation is projected onto the respective contour plot. Solid black lines show the bond paths, which follow the line of maximum electron density between bonded atoms. The corresponding bond critical points (bcps) are shown as green circles. Red contours indicate regions of local charge accumulation (∇^2^ *ρ*(*r*)<0); blue contours indicate regions of local charge depletion (∇^2^ *ρ*(*r*)>0).

An XRD analysis of the bromonium borate **10**[B(C_6_F_5_)_4_] was possible from single crystals obtained from its mixture with Li[B(C_6_F_5_)_4_] in benzene at room temperature. Both borates co‐crystallized on standing at ambient temperature. In the crystal, the cation and anion are well separated. The closest Si/F or Br/F contacts are larger than the corresponding sum of the van der Waals radii (Si/F: 384 pm vs. ΣvdW*R*: 357 pm; Br/F: 362 pm vs. ΣvdW*R*: 330 pm).^[12]^ The molecular structure of the cation is shown in Figure 6. It closely resembles that of related acenaphthyl‐based, selanyl‐stabilized silyl cations.[^10a^, ^10b^] The silicon atom in **10** is tetra‐coordinated with a significant trigonal flattening of the tetrahedral coordination at the silicon atom (Σ*α*(SiC_3_)=349.5°). This is close to the values reported for silylium carboranes, for example, [ipr_3_Si][HCB_11_H_5_Br_6_] (Σ*α*(SiC_3_)=351°).^[23]^ The bromine atom is di‐coordinated with an almost orthogonal arrangement of both substituents (C^1^‐Br‐Si=91.1°). The silicon–bromine bond (242.5 pm) is significantly larger than the standard value (230 pm)^[24]^ and also longer than measured in the bis‐silyl‐bromonium borate [(Me_3_Si)_2_Br][B(C_6_F_5_)_4_] (238 pm).^[9a]^ The attractive interaction between the silicon and bromine atoms in cation **10** does not result in significant steric strain as both substituents are almost coplanar to the naphthyl backbone and the sum of the bay angles (Σ*β*=358.5°) is almost identical to that of naphthalene (Σ*β*=359°).[^15a^, ^25^]

**Figure 7:**
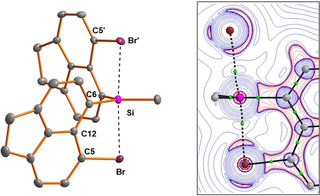
Left: Molecular structure of siliconium ion **4 c** in the crystal of **4 c**_2_[B_12_Br_12_]**⋅**3 C_6_H_4_Cl_2_ (thermal ellipsoids are shown at the 50 % probability level, hydrogen atoms are omitted for clarity). Pertinent bond lengths [pm] and bond angles [°]: Br(1)−C^5^ 191.2(2), Br(2)−C^5'^ 190.69(18), Si−C^6^ 186.8(2), Si−C^6'^ 187.73(19), Si−Br 265.08(6), Si−Br“ 270.00(6), Br‐C^5^‐C^12^ 114.98(13), Br′‐C^5'^‐C^12'^ 115.16(14), Si‐C^6^‐C^12^ 117.35(14), Si‐C^6'^‐C^12'^ 118.07(15), C^5^‐C^12^‐C^6^ 127.94(17), C^5^'‐C^12'^‐C^6^' 127.51(19), Br‐Si‐Br” 176.23(2), ∑*α*(SiC_3_) 360.0, sum of bay angles Σ*β* 360.3 and Σ*β*' 360.8. Right: 2D contour plot of the calculated Laplacian of the electron density, ∇^2^ *ρ*(*r*), in the Si‐C^6^‐Br plane of cation **4 c**. Relevant parts of the molecular graphs of the cation are projected onto the contour plot. Solid black lines show the bond paths, which follow the line of maximum electron density between bonded atoms. The corresponding bond critical points (bcps) are shown as green circles. Red contours indicate regions of local charge accumulation (∇^2^ *ρ*(*r*)<0); blue contours indicate regions of local charge depletion (∇^2^ *ρ*(*r*)>0).

Single crystals of siliconium dodecaborate **4 c**_2_[B_12_Br_12_] suitable for XRD analysis were obtained from 1,2‐dichlorobenzene solution at room temperature. The molecular structure of siliconium ion **4 c** is shown in Figure 7. The silicon atom is penta‐coordinated and adopts a trigonal bipyramidal coordination environment. The trigonal basis is defined by the three carbon substituents. The deviation of the silicon atom from this plane is insignificant (0.7 pm). The bromine atoms take up the axial positions and the Br‐Si‐Br axis deviate slightly from linearity (Br‐Si‐Br=176.2°). The Br−Si bonds are very long (265.1 and 270.0 pm) as expected for 3c‐4e Br‐Si‐Br bonds. These bonds are even longer than Si−Br linkages in penta‐ or hexa‐coordinated silicon complexes (227–252 pm).^[26]^ The molecular structure of siliconium ion **4 c** shows no structural indication of strain imposed by the two acenaphthyl substituents. The two C‐Br‐Si angles are almost perpendicular (88.8°, 87.3°) and the sum of the bay angles of both acenaphthyl ligands are close to 360°, only slightly smaller than that of acenaphthene (Σ*β*=368°).^[14]^ This indicates attractive interactions between silicon and both bromo substituents.^[15]^

**Figure 8:**
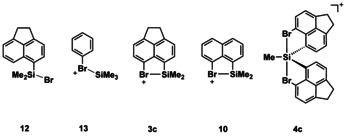
Compounds that are pertinent to the discussion.

**Figure 9:**
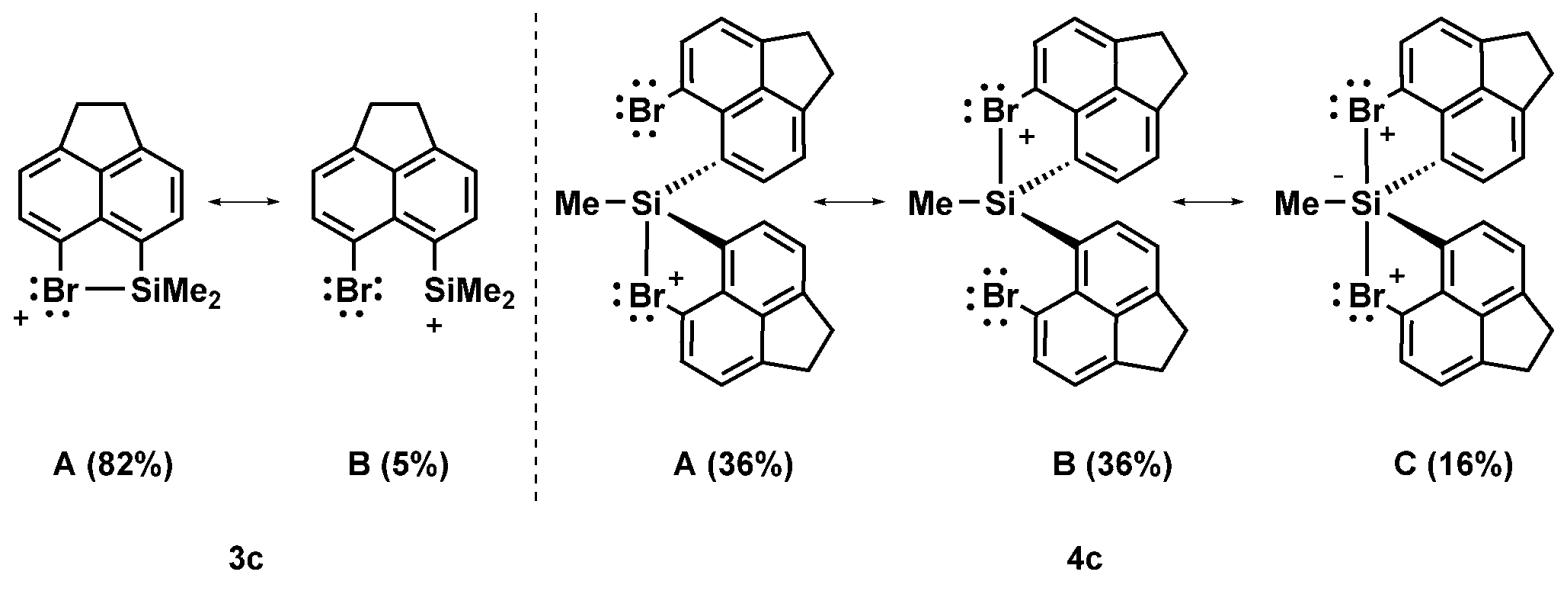
Leading reference structures of cation **3 c** and **4 c** according to the NBO/NRT method.

The results of our computational investigation by using the M06‐2X density functional and the def2tzvp basis set reveal the significant stabilization of the cations **3 c**, **10**, and **4 c** by the bromo substituents and classify cations **3 c** and **10** as silylated bromonium ions and cation **4 c** as a penta‐coordinated siliconium ion. This applied model chemistry provides calculated molecular structures for cations **3 c**, **10**, and **4 c**, which are close to those determined in the solid state by XRD. The deviation of the computed Si−Br bond lengths from the experimentally determined is less than 3 % (see Table 1 for a comparison of pertinent structural parameters). The Si/Br distances in halonium ions **3 c** and **10** are significantly smaller than the sum of the van der Waals radii (393 pm)^[12]^ but they are larger by 9 % (**3 c**) and 8 % (**10**) than calculated for the acenaphthylsilyl bromide **12**. They are almost identical to the predicted Si−Br bond lengths in trimethylsilylated bromobenzene **13** (see Table 1, Figure 8). As expected from previous studies, the change from the acenaphthyl‐based cation **3 c** to the naphthyl cation **10** is connected with a slight decrease of the Si−Br bond lengths by 3.1 pm.^[10]^ Common to all investigated bromonium ions **3 c**, **10**, and **13** is the pronounced trigonal flattening of the coordination environment of the silicon atom as indicated by the sum of the bonding angles around the silicon atom Σ*α*(SiC_3_) of approximately 350°. The calculated bond dissociation energy of the Si−Br bond in bromonium ion **10** is by 23 kJ mol^−1^ smaller than predicted for the complex **13** between bromobenzene and trimethylsilylium ion and it is additionally decreased by 27 kJ mol^−1^ for the acenaphthyl‐based cation **3 c** (Table 1 and the Supporting Information). This suggests significant destabilization of the Si−Br linkage in the cyclic bromonium ions **3 c** and **10**, which is not expected from structural indicators for strain as, for example, by the sum of the bay angles Σ*β*. This parameter is for both bromonium ions very close to the values calculated for the neutral hydrocarbons (naphthalene: Σ*β*=359.4°, acenaphthene: Σ*β*=366.7°). The analysis of the computed electron density of the cations **3 c**, **10**, and **13** in the framework of Bader's quantum theory of atoms in molecules (QTAIM)^[27]^ reveals for all cations topological molecular graphs that enclose bond paths between the bromine and the silicon atoms. 2D Laplacian contour plots of these cations are very similar and Figure 6 provides the Laplacian contour plot of bromonium cation **10** as a representative example. The bonding interaction between the bromine and the silicon atom in cation **10** is typified as being strongly polar covalent by the properties at the bond critical point (bcp).^[28]^ The bcp is shifted to the electropositive silicon atom and it shows a relatively large electron density *ρ*(bcp), a positive Laplacian ∇^2^ *ρ*(bcp), and a negative total energy density *H*(bcp) (see Table 1). The NBO analysis,^[29]^ which is complementary to the QTAIM method, reveals a similar charge distribution in all three bromonium ions and a substantial increase of the positive charge at the bromine atom when compared with neutral silyl bromide **12**. Finally, natural resonance theory (NRT)^[30]^ gives the halonium resonance structure **3 c**(**A**) overwhelming importance (Figure 9).

**Table 1:** Calculated characteristic parameters for the Si−Br linkage in cations **3 c**, **10**, **4 c**, and related compounds (at M06‐2X/def2tzvp, BDE: bond dissociation energy; WBI: Wiberg bond index, Ch: natural charge according to the NBO analysis, *ρ*: electron density at the bond critical point (bcp), ∇^2^ *ρ*: Laplacian of the electron density at the bcp; *H*: total energy at the bcp). Experimental structural parameter from XRD analysis are given in parenthesis.

	**12**	**13**	**3 c**	**10**	**4 c**
Si−Br [pm]	225.6	246.5	247.0	243.9 (242.5)	274.7; 272.1 (270.0; 265.1)
Σ*α*(SiC_3_) [°]	336.7	349.7	351.6	350.7 (349.5)	359.9 (360.0)
Σ*β* [°]	368.0	–	358.3	358.7 (358.5)	364.6; 364.2 (360.8; 360.3)
BDE^[a]^ [kJ mol^−1^]	–	136	86	113	76
WBI (SiBr)	0.88	0.54	0.56	0.57	0.33; 0.36
Ch(Si) [a.u.]	1.60	1.71	1.72	1.71	1.76
Ch(Br) [a.u.]	−0.35	0.27	0.27	0.27	0.20; 0.20
*ρ* [a.u.]	0.082	0.054	0.054	0.057	0.035; 0.035
∇ ^2^ *ρ* [a.u.]	0.092	0.031	0.032	0.042	0.009; 0.012
*H* [a.u.]	−0.044	−0.025	−0.026	−0.025	−0.001; −0.001

[a] Calculated by using appropriate isodesmic equations, see Schemes S3–S5 and Tables S8, S9 in the Supporting Information.

A similar detailed quantum mechanical analysis for siliconium ion **4 c** reveals the characteristic pattern of three‐center–four‐electron bonding involving a main group atom in a trigonal bipyramidal coordination environment. The two apical oriented Si−Br linkages in cation **4 c** are significantly longer than standard Si−Br bonds, but smaller than the sum of the van der Waals radii. The interaction with the two bromine atoms stabilizes cation **4 c** by 76 kJ mol^−1^ and the QTAIM as well as the natural bond orbital (NBO) analysis indicates rather ionic Si−Br bonds consistent with dominating halonium ion resonance structures **4 c**(**A**) and **4 c**(**B**) according to NRT calculations (Figures 7, 9, Table 1).

There are two slightly different mechanistic proposals for the substituent redistribution reactions between silyl cations and silanes.[^8^, ^20b^, ^20d^] Central to both mechanisms is the formation of the 1,1‐bis‐silylated arenium ion **14** by electrophilic attack of the bromonium **3 c** on silane **1 c**. There is the possibility of a direct 1,3‐methyl group migration between both silicon atoms generating one equivalent of trimethylsilane and cation **4 c**.[^8^, ^20b^] Alternatively, a preceding 1,2‐silyl shift to give the 1,2‐disilyl‐substituted arenium ion **15** and a subsequent 1,4‐Me‐shift is possible.^[20d]^

**Figure 10:**
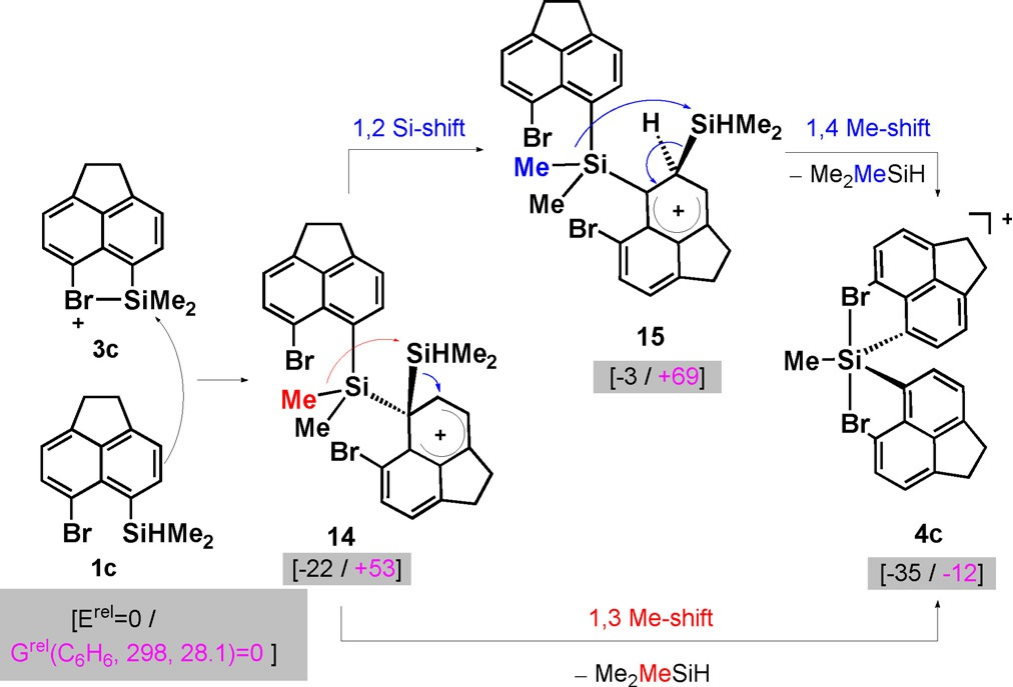
Proposed mechanism of the substituent redistribution reaction to give siliconium ion **4 c**. Relative absolute energies *E* ^rel^ and Gibbs enthalpies *G* ^rel^(C_6_H_6_, 298, 28.1) benzene, at *T*=298 K and *p*=28.1 MPa of the intermediates were calculated at PCM/M06‐2X/def2tzvp and are given in brackets in kJ mol^−1^.

Calculations at the M06‐2X/def2tzvp level of theory indicate that the overall reaction to give siliconium ion **4 c** and Me_3_SiH is slightly exothermic (Figure 10). Inclusion of entropy and solvent effects in our computational model render the reaction thermo‐neutral, which suggests that the formation of the volatile Me_3_SiH and its further disproportionation is an important driving force. The formation of key intermediates **14** and **15** is slightly exothermic but entropically not favored. The computed relative energies for both intermediates in question are plausible for a reaction proceeding at or slightly below room temperature.

**Scheme 4:**
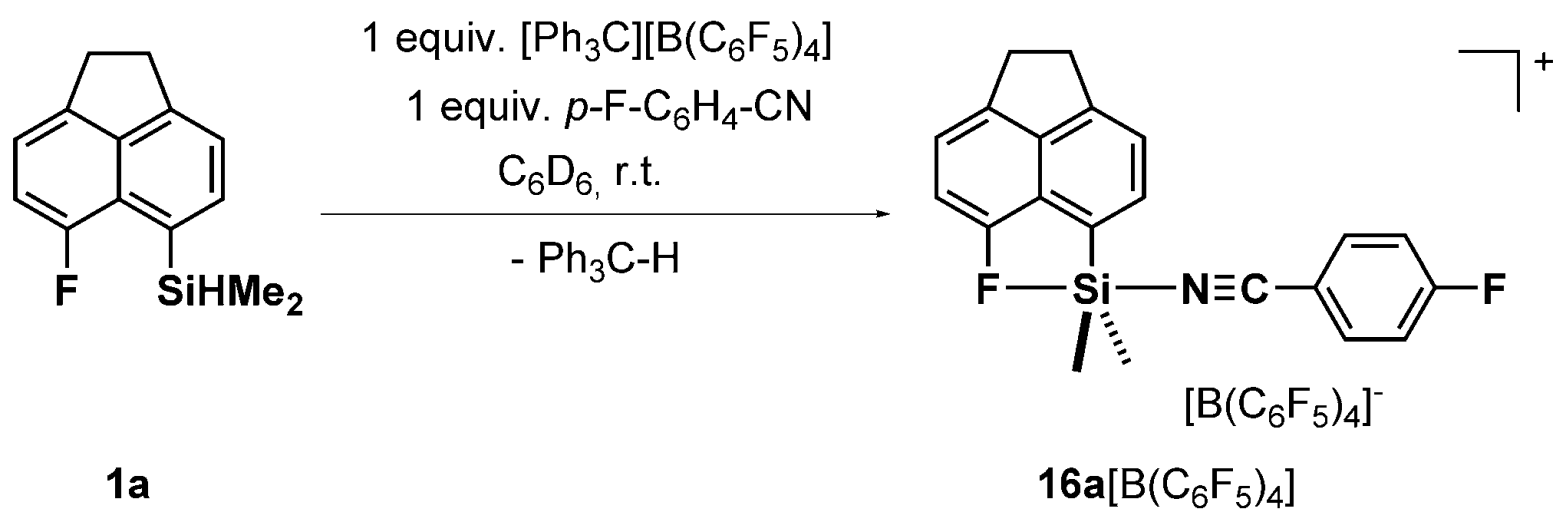
Synthesis of nitrilium borate **16 a**[B(C_6_F_5_)_4_] from acenaphthyl silane **1 a**.

**Figure 11:**
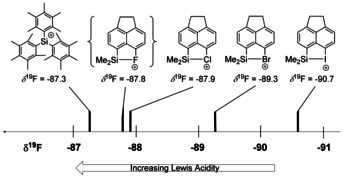
The FBN acidity scale for silyl halonium ions **3** and silylium ion [(Me_5_C_6_)_3_Si]^+^ in benzene.

Next, we tested the Lewis acidity of the series of silyl halonium ions **3** by using the recently developed *p*‐fluorobenzonitrile (FBN) probe, which uses the ^19^F NMR chemical shift of *p*‐fluorobenzonitrile complexes of Lewis acids to gauge their acidity.^[10b]^ To include the fluoronium **3 a** in this comparison, we synthesized cation **3 a** in the presence of one equivalent of FBN in [D_6_]benzene (Scheme 4). The ionization of **1 a** in the presence of one equivalent of FBN yields quantitatively the nitrilium borate **16 a**[B(C_6_F_5_)_4_] (*δ* ^29^Si=22.1, ^1^ *J*(SiC)=63 and 83 Hz, *δ* ^19^F(CF)=−122.13, see also the Supporting Information). The increase of the ^1^ *J*(SiC) coupling constants upon ionization indicates an increase of the coordination number (CN) for the silicon atom from CN=4 (**1 a**) to CN=5 (**16 a**) (**1 a**: ^1^ *J*(SiC)=53 and 66 Hz). This suggests simultaneous coordination of the nitrile and the fluoro substituent to the silicon. The decisive ^19^F NMR chemical shift of the *para*‐fluorine nuclei is *δ* ^19^F=−87.8. This places fluoronium ion **3 a** at the very Lewis acidic end of the FBN scale, close to that of non‐stabilized triarylsilylium ions (Me_5_C_6_)_3_Si^+^ (*δ* ^19^F=−87.3). The FBN value of the chloronium ion **3 b** was determined from the obtained mixture with the siliconium ion **4 b** and those of the halonium ions **3 c**, **d** from freshly prepared samples in benzene. The results shown in Figure 11 indicate the expected decrease of the silyl Lewis acidity from fluorine to iodine as the remote substituent and it mirrors the increase of the strength of the silicon–halogen bond from fluorine to iodine (BDE(Si−F)=25, BDE(Si−Cl)=72, BDE(Si−Br)=86, BDE(Si−I)=98 kJ mol^−1^, see Table S9 in the Supporting Information). Interestingly, the different Lewis acidity of cations **3** reflects also their reactivity in the formation of the corresponding siliconium ion **4**. The iodonium ion **3 d** is not reactive enough to undergo the initial electrophilic attack on silane **1 d** (Figure 10). The chloronium ion **3 b** is more Lewis acidic and undergoes the addition to silane **1 b** even at low temperatures. Finally, for the bromonium ion **3 c** the formation of siliconium ion **4 c** can be controlled by the temperature and stoichiometry of the reaction.

## Conclusion

The intramolecular interaction between remote halo substituents and silyl cations was studied for *peri*‐substituted acenaphthenes and for one *peri*‐bromo‐substituted naphthalene. Although the acenaphthyl‐based silylated fluoronium ion **3 a** could not be synthesized, the corresponding iodonium ion **3 d** was formed selectively by using the Corey hydride transfer reaction. Similarly, the chloronium and bromonium borates **3 b**[B(C_6_F_5_)_4_] and **3 c**[B(C_6_F_5_)_4_] were synthesized. Both silylated halonium ions are, however, reactive toward the starting silane **1 b** and **1 c** and undergo substituent redistribution reactions to form penta‐coordinated siliconium ions **4 b** and **4 c**. Control over the reaction conditions allows the selective synthesis of the silylated bromonium ion **3 c** and the corresponding siliconium ion **4 c**. Analysis of the XRD data supported by the results of DFT calculations identifies cation **3 c** and likewise also the chloro and iodo species as silylated halonium ions. Siliconium ions **4 b** and **4 c** represent rare examples of organo‐substituted silicon cations with two stabilizing halogen substituents and bromo stabilized species **4 c** is the first structurally characterized example of a dibromo organosiliconium ion.[^26^, ^31^] The Lewis acidity of the silylated halonium ions was evaluated by using the recently developed FBN method.^[10b]^ This method shows the expected trend of decreasing silyl Lewis acidity from fluoro to iodo substituents, which is paralleled by an increase of the silicon–halogen bond energy. The silyl fluoronium ion **3 a** is a strong Lewis acid, reaching the acidity of non‐stabilized silylium ions, and the Lewis acidity of the iodo species is slightly higher than that of related sulfur‐stabilized cations.^[10b]^

## Conflict of interest

The authors declare no conflict of interest.

## Supporting information

As a service to our authors and readers, this journal provides supporting information supplied by the authors. Such materials are peer reviewed and may be re‐organized for online delivery, but are not copy‐edited or typeset. Technical support issues arising from supporting information (other than missing files) should be addressed to the authors.

Supplementary
