# Supplementary material for: Intramolecular Halo Stabilization of Silyl Cations—Silylated Halonium‐ and Bis‐Halo‐Substituted Siliconium Borates
Source: Chemistry. 2021 Jan 18;27(10):3496–503. doi: 10.1002/chem.202004838 (PMC7898513; doi:10.1002/chem.202004838)
Supplement: Supplementary file 1 — Supplementary [file CHEM-27-3496-s001.pdf]

Chemistry–A European Journal

Supporting Information

Intramolecular Halo Stabilization of Silyl Cations—Silylated Halonium- and Bis-Halo-Substituted Siliconium Borates

Anastasia Merk, Lukas Bührmann, Natalie Kordts, Katharina Görtemaker, Marc Schmidtmann, and Thomas Müller*[a]

Supporting Information

Table of Contents

**1.** Experimental part	S2
**1.1** General remarks	S2
**1.2** Synthesis of acenaphthene derivatives	S4
**1.3** Synthesis of silanes	S8
**1.4** Synthesis of halonium and siliconium ions	S21
**1.5** Synthesis of nitrilium ions	S51
**1.6** Data from X-ray diffraction	S68
**2.** Computational part	S76
**3.** Reference	S80

1. Experimental part

1.1 General Remarks

Due to the sensitivity of the compounds all reactions were carried out under inert conditions using standard Schlenk techniques or a standard glove box (Braun Unilab), except for the synthesis of 5-chloroacenaphthene **17** and 5-bromo-6-chloroacenaphthene **18**. Commercially available argon 5.0 or nitrogen 5.0 was used as inert gas. The glassware was stored in an oven at 160°C and evacuated prior to use. The solvents *n*-hexane and tetrahydrofuran were dried over sodium-potassium alloy and distilled under inert atmosphere. The solvents *n*-pentane and benzene as well as the deuterated solvents were first dried over sodium-potassium alloy and then either condensed before use or stored over molecular sieve (4 Å). Acetonitrile-d_3 was dried over molecular sieve (4 Å). *N*-Butyl lithium (*n*BuLi) was used as a 1.6 M solution in *n*-hexane. 5,6-Dibromoacenaphtene [S1], 1,8-Dibromonaphthalene [S2], 5-bromo-6-dimethylsilylacenaphthene **1c** [S3], 5-iodo-6-dimethylsilylacenaphthene **1d** [S3], 1-bromo-8-dimethylsilylnaphthalene **2** [S4] and trityl borate $[Ph_3C][B(C_6F_5)_4]$ [S5] were synthesized according to literature procedures.

All other chemicals are commercially available and used as delivered if not mentioned otherwise. Commercially available solid materials were stored and weighted in a glove box or dried at high vacuum before use.

Thin-layer chromatography was performed using commercial available aluminum foil (Fluka) coated with silica gel 60 and fluorescent indicator F254. For the column chromatography silica gel of the mesh size 60 from Merck was used.

NMR spectra were recorded on Bruker Fourier 300, Avance DRX 500, Bruker Avance III 500, Bruker Avance III WB500 spectrometer. ^{1}H NMR spectra were referenced to the residual solvent resonance as internal standard (benzene-d_6: $\delta^1H(C_6D_5H)$ = 7.20, toluene-d_8: $\delta^1H(C_6D_5CD_2H)$ = 2.08, acetonitrile-d_3: $\delta^1H(CD_2HCN)$ = 1.94) and ^{13}C NMR spectra by using the central line of the solvent signal (benzene-d_6: $\delta^{13}C(C_6D_6)$ = 128.0, toluene-d_8: $\delta^{13}C(C_6D_5CD_3)$ = 20.4, acetonitrile-d_3: $\delta^{13}C(CD_3CN)$ = 1.32). ^{29}Si NMR spectra were referenced to an external standard ($\delta\ ^{29}Si(Me_2SiHCl)$ = 11.1 versus tetramethylsilane (TMS)), ^{11}B NMR spectra against $BF_3{\cdot}OEt_2$ ($\delta^{11}B(BF_3{\cdot}OEt_2)$ = 0.0) and ^{19}F NMR spectra against external $CFCl_3$ ($\delta^{19}F(CFCl_3)$ = 0.0). The ^{29}Si NMR inverse gated spectra were recorded with a relaxation delay D1 = 10 s. The ^{29}Si INEPT spectra were recorded with delays D3 = 0.0084 s and D4 = 0.0313 s, if not given otherwise.

High resolution mass spectra were measured on a *Thermo Scientific DFS* spectrometer using EI.

Infrared spectra were performed on a *Bruker Tensor 27* spectrometer with a *MKII Reflection Golden Gate Single Diamond ATR system*.

Combustion analyses (C, H, N, S) were obtained on a Euro EA Element Analyzer with EuroVector equipment. For silanes, combustion analysis values for carbon show often too low values, what may be attributed to the formation and incomplete combustion of silicon carbide, although vanadium pentoxide as combustion aid was used. Satisfactory combustion analyses could not be obtained from all silyl borates due to their high reactivity.

1 Synthesis of the educts

5-chloroacenaphthene ***17***

Acenaphthene (1.0 equiv, 5 g, 32.42 mmol) was dissolved in 50 mL of *N*,*N*-dimethylformamide and a solution of *N*-chlorosuccinimide (NCS) (1.0 equiv, 4.33 g, 32.42 mmol) in 50 mL of *N*,*N*-dimethylformamide was added dropwise at 0 °C. The reaction mixture was allowed to warm to r.t. overnight and stirred for a total period of 24 hours, before it was concentrated to nearly 10 mL. Purified water was added and the precipitate was filtered off. After that the filtrate was extracted with *n*-hexane (2 x 20 mL), the organic phases were combined and dried over $MgSO_4$. The solvent was removed under low pressure. Both the precipitate and the residue of the organic phase contained 5-chloroacenaphthene, were combined and subjected to column chromatography eluted with petroleum ether 40/60 (R_f = 0.56) to give the product (3.91 g, 20.72 mmol, 64 %, **M.p.**: 59-61 °C) as a yellow solid.

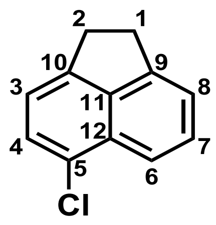

17

^{1}H NMR (500.13 MHz, 300.7 K, $CDCl_3$): δ = 3.33-3.42 (m, 4H, CH_2, H-1, H-2), 7.15-7.17 (m, 1H, 3-H), 7.32 (d, 1H, $^{3}J_{H,H}$ = 6.8 Hz, H-8), 7.46 (d, 1H, $^{3}J_{H,H}$ = 7.3 Hz, H-4), 7.51-7.54 (m, 1H, H-7), 7.81 (d, 1H, $^{3}J_{H,H}$ = 8.3 Hz, 6-H). **^{13}C{^{1}H} NMR** (125.77 MHz, 300.7 K, $CDCl_3$): δ = 29.8 (CH_2, C-2), 30.7 (CH_2, C-1), 119.4 (CH), 119.5 (CH),120.1 (CH, C-8), 126.8 (C, C-5), 127.3 (CH, C-4), 128.8 (CH, C-7), 129.4 (C, C-12), 140.2 (C, C-11), 145.1 (C, C-10), 146.1 (C, C-9).

5-bromo-6-chloroacenaphthene ***18***

A solution of *N*-bromosuccinimide (NBS) (1.0 equiv, 3.49 g, 19.56 mmol) in 50 mL of *N*,*N*-dimethylformamide was added dropwise to an ice-cooled solution of 5-chloroacenaphthene **17** (1.0 equiv, 3.69 g, 19.56 mmol) in 50 mL of *N*,*N*-dimethylformamide and was stirred for 20 min. The reaction mixture was allowed to warm to r.t. overnight and stirred for a total period of 18 hours. The previously yellow solution turned orange. The solvent was removed and the residue was dissolved in *n*-hexane. Purified water was added and the precipitate was filtered off. After that the filtrate was extracted with *n*-hexane (2 x 100 mL), the organic phases were combined and dried over $MgSO_4$. The solvent was removed under low pressure. Both the precipitate and the residue of the organic phase contained 5-bromo-6-chloroacenaphthene **18**, were combined and subjected to column chromatography eluted with petroleum ether 40/60 (R_f = 0.45) to give the product (1.13 g, 4.21 mmol, 22 %, **M.p.**: 150-153 °C) as a pale yellow solid.

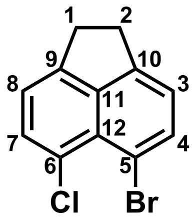

18

^{1}H NMR (500.13 MHz, 299.9 K, $CDCl_3$): δ = 3.27 (s, 4H, CH_2, H-1, H-2), 7.04 (d, 1H, $^3J_{H,H}$ = 7.3 Hz, H-3), 7.11 (d, 1H, $^3J_{H,H}$ = 7.4 Hz, H-8), 7.48 (d, 1H, $^3J_{H,H}$ = 7.3 Hz, H-7), 7.71 (d, 1H, $^3J_{H,H}$ = 7.4 Hz, H-4). **^{13}C{^{1}H} NMR** (125.77 MHz, 300.0 K, $CDCl_3$): δ = 30.0 (CH_2), 30.1 (CH_2), 113.0 (C, C-5), 120.3 (CH, C-8), 120.9 (CH, C-3), 126.2 (C, C-6), 126.8 (C, C-12), 131.5 (CH, C-7), 135.5 (CH, C-4), 141.9 (C, C-11), 146.1 (C, C-9), 146.7 (C, C-10).

5-bromo-6-fluoroacenaphthene ***19***

5,6-Dibromoacenaphthene (1.0 equiv, 2.72 g, 8.72 mmol) was dissolved in 100 mL of THF and cooled to -80°C. Then *n*-butyl lithium (1.6 M in *n*-hexane, 1.05 equiv, 5.7 mL) was added dropwise and the reaction mixture was stirred for at least one hour at the same temperature. After that *N*-fluorobenzenesulfonimide (NFSI) (1.2 equiv, 3.30 g, 10.47 mmol) in 100 mL THF was added slowly and the mixture was stirred for additional 60 minutes at -80 °C. Then the reaction mixture was allowed to warm to r.t. overnight and poured into NH_4Cl solution (50 mL). After that the aqueous phase was extracted with *n*-hexane (3 x 50 mL), the organic phases were combined and dried over $MgSO_4$. The solvent was removed under low pressure. The product was purified by column chromatography using *n*-pentane (R_f = 0.41) as eluent. Yield: 1.76 g (6.99 mmol, 80 %, **M.p.**: 99-101 °C).

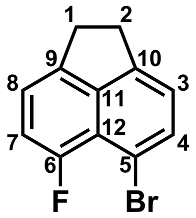

19

^{1}H NMR (499.87 MHz, 305.1 K, $CDCl_3$): δ = 3.31-3.34 (m, 4H, CH_2, H-1, H-2), 7.07 (d, 1H, $^3J_{H,H}$ = 7.4 Hz, 3-H), 7.10-7.16 (m, 2H, H-7, H-8), 7.62 (d, 1H, $^3J_{H,H}$ = 7.5 Hz, H-4). **^{13}C{^{1}H} NMR** (125.71 MHz, 305.0 K, $CDCl_3$): δ = 29.9 (s, CH_2, C-1), 30.5 (s, CH_2, C-2), 109.9-100.0 (m, C, C-5), 113.7 (d, $^2J_{C,F}$ = 22.8 Hz CH, C-7), 119.8 (d, $^3J_{C,F}$ = 7.6 Hz, CH, C-8), 120.8 (m, C, C-12), 120.9 (m, CH, C-3), 133.3 (s, CH, C-4), 141.8 (m, C), 142.3 (m, C), 145.5 (m, C, C-10), 155.5 (d, $^1J_{C,F}$ = 253.9 Hz, C, C-6). **^{19}F{^{1}H} NMR** (470.30 MHz, 305.1 K, $CDCl_3$): δ = -122.5.

*5,6-diiodoacenaphthene **20***

The original procedure [S6] was slightly modified. 5,6-Dibromoacenaphthene (1.0 equiv, 6 g, 19.23 mmol) was dissolved in 400 mL of Diethylether and cooled to -10°C – 0 °C. Then a mixture containing *n*-butyl lithium (1.6 M in *n*-hexane, 2.4 equiv, 29 mL) and tetramethylethylenediamine (TMEDA) (2.66 equiv, 8 mL) was added dropwise and the reaction mixture was stirred for at least one hour at -10°C – 0 °C. After iodine (2.2 equiv, 10.74 g, 42.31 mmol) in 120 mL diethylether was added dropwise over a period of 30 min at -10 °C – 0 °C. The dark mixture was stirred for 1 h at the same temperature and was allowed to warm to room temperature overnight. After that 500 mL of aqueous 5 % sodium thiosulfate was added to the mixture with vigorous stirring. The organic phase was first washed with aqueous 5 % sodium thiosulfate, then with water and dried over $MgSO_4$. The solvent was removed under low pressure. The product was purified by column chromatography using *n*-pentane (R_f = 0.41) as eluent. Yield: 2.30 g (5.67 mmol, 29.5 %).

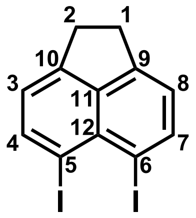

20

^{1}H NMR (499.87 MHz, 305.0 K, $CDCl_3$): δ = 3.24-3.28 (m, 4H, CH_2, H-1, H-2), 6.91 (d, 2H, $^3J_{H,H}$ = 7.4 Hz, H-3, H-8), 8.23 (d, 2H, $^3J_{H,H}$ = 7.4 Hz, H-4, H-7). **^{13}C{^{1}H} NMR** (125.71 MHz, 305.0 K, $CDCl_3$): δ = 29.9 (CH_2, C-1, C-2), 89.4 (C, C-5, C-6), 121.8 (CH), 130.9 (C), 140.3 (C), 144.6 (CH), 148.9 (C).

2 Synthesis of the silanes

General procedure A: The starting material was dissolved in THF and cooled to -80°C. Then *n*-butyl lithium (*n*BuLi) was added dropwise and the reaction mixture was stirred for at least one hour at the same temperature. After that chlorodimethylsilane was added and the mixture was stirred for additional 60 minutes at -80 °C. Then the reaction mixture was allowed to warm to r.t. overnight. After adding NH_4Cl solution (10 mL) to the reaction mixture and extraction with Et_2O or *n*-pentane (3 x 20 mL), the organic phases were combined, dried over $MgSO_4$ and the solvent was removed under low pressure. The silanes were purified by column chromatography or crystallization.

6-fluoro-5-dimethylsilylacenaphtene ***1a***

The synthesis of the title compound was performed according to general procedure **A** using 5-bromo-6-fluoroacenaphthene **19** (1.0 equiv, 1.80 g, 7.17 mmol), *n*BuLi (1.6 M in *n*-hexane, 1 equiv, 4.5 mL, 7.17 mmol) and chlorodimethylsilane (1.0 equiv, 0.78 mL, 7.17 mmol). Silane **1a** was obtained as a colorless solid after column chromatography using *n*-pentane (R_f = 0.65) as eluent. Yield: 1.45 g (6.28 mmol, 88 %, **M.p.**: 40-42 °C)

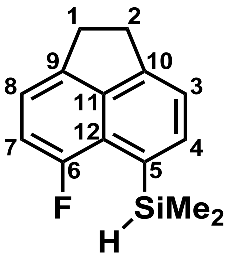

1a

^{1}H NMR (499.87 MHz, 305.0 K, C_6D_6): δ = 0.58-0.60 (m, 6H, $Si(CH_3)_2$), 2.85-2.99 (m, 4H, CH_2, H-1, H-2), 5.10 (dsept, 1H, $^{TS}J_{H,F}$ = 11.0 Hz, $^3J_{H,H}$ = 3.7 Hz, $^1J_{H,Si}$ = 191.4 Hz, Si-H), 6.80-6.82 (m, 1H, H-8), 6.99 (dd, 1H, $^3J_{H,H}$ = 7.5 Hz, $^3J_{H,F}$ = 12.5 Hz, H-7), 7.04 (d, 1H, $^3J_{H,H}$ = 7.0 Hz, H-3), 7.79 (d, 1H, $^3J_{H,H}$ = 6.8 Hz, H-4). **^{1}H NMR** (300.14 MHz, 305.2 K, C_6D_6): δ = 0.58-0.60 (m, 6H, $Si(CH_3)_2$), 2.86-2.98 (m, 4H, H-1, H-2), 5.10 (dsept, 1H, $^{TS}J_{H,F}$ = 11.0 Hz, $^3J_{H,H}$ = 3.7 Hz, $^1J_{H,Si}$ = 191.4 Hz, Si-H), 6.79-6.83 (m, 1H, H-8), 6.95-7.05 (m, 2H, H-7, H-3), 7.79 (d, 1H, $^3J_{H,H}$ = 6.8 Hz, H-4). **^{13}C{^{1}H} NMR** (125.71 MHz, 305.0 K, C_6D_6): δ = -2.4 (CH_3, d, $^{TS}J_{C,F}$ = 7.4 Hz, $^1J_{C,Si}$ = 52.6 Hz, $Si(CH_3)_2$), 29.5 (s, CH_2, C-1), 30.9 (s, CH_2, C-2), 112.3 (d, $^2J_{C,F}$ = 22.6 Hz, CH, C-7), 119.0 (d, $^3J_{C,F}$ = 8.0 Hz, CH,C-8), 120.1 (s, CH, C-3), 125.6 (d, $^2J_{C,F}$ = 18.8 Hz, C, C-12), 126.8 (d, $^{TS}J_{C,F}$ = 2.9 Hz, $^1J_{C,Si}$ = 66.5 Hz, C, C-5), 137.4 (s, CH, C-4), 141.3 (d, $^3J_{C,F}$ = 7.9 Hz, C, C-11), 142.1 (d, $^4J_{C,F}$ = 3.1 Hz, C, C-9), 148.0 (d, $^4J_{C,F}$ = 3.0 Hz, C, C-10), 158.1 (d, $^1J_{C,F}$ = 247.9 Hz, C, C-6). **^{13}C{^{1}H} NMR** (75.48 MHz, 305.0 K, C_6D_6): δ = -2.4 (CH_3, d, $^{TS}J_{C,F}$ = 7.5

Hz, $^1J_{C,Si}$ = 52.6 Hz, $Si(CH_3)_2$), 29.6 (s, CH_2, C-1), 30.9 (s, CH_2, C-2), 112.3 (d, $^2J_{C,F}$ = 22.8 Hz, CH, C-7), 119.0 (d, $^3J_{C,F}$ = 7.9 Hz, CH,C-8), 120.1 (s, CH, C-3), 125.6 (d, $^2J_{C,F}$ = 18.7 Hz, C, C-12), 126.81-128.85 (m, C, C-5), 137.4 (s, CH, C-4), 141.3 (d, $^3J_{C,F}$ = 7.8 Hz, C, C-11), 142.10-142.14 (m, C, C-9), 147.95-147.99 (m, C, C-10), 158.1 (d, $^1J_{C,F}$ = 247.7 Hz, C, C-6). **^{13}C{^{19}F} NMR** (125.85 MHz, 303.1K, C_6D_6): δ = -2.5 (CH_3, qm, $^1J_{C,H}$ = 120 Hz, $Si(CH_3)_2$), 29.6 (tm, $^1J_{C,H}$ = 132 Hz, CH_2, C-1), 30.9 (tm, $^1J_{C,H}$ = 132 Hz, CH_2, C-2), 112.3 (dm, $^1J_{C,H}$ = 158 Hz, CH, C-7), 119.0 (dm, $^1J_{C,H}$ = 160 Hz, CH,C-8), 120.1 (dm, $^1J_{C,H}$ = 159 Hz, CH, C-3), 125.6-125.7 (m, C, C-12), 126.8-127.0 (m, C, C-5), 137.5 (dm, $^1J_{C,H}$ = 157 Hz, CH, C-4), 141.3-141.4 (m, C, C-11), 142.0-142.2 (m, C, C-9), 147.9-148.1 (m, C, C-10), 158.0-158.2 (m, C, C-6). **^{29}Si{^{1}H} NMR** (99.31 MHz, 305.0 K, C_6D_6): δ = -12.4. **^{29}Si{^{1}H} INEPT NMR** (99.31 MHz, 305.0 K, C_6D_6): δ = -12.5 (d, $^{TS}J_{Si,F}$ = 5.7 Hz, $^1J_{Si,C}$ = 52.8 Hz ($Si(CH_3)_2$), $^1J_{Si,C}$ = 65.5 Hz (Si–C-5)). **^{29}Si INEPT NMR** (99.31 MHz, 305.0 K, C_6D_6): δ = -12.3 (dm, $^1J_{Si,H}$ = 191.3 Hz). **^{19}F{^{1}H} NMR** (470.30 MHz, 305.1 K, C_6D_6δ = -119.4. **^{19}F{^{1}H} NMR** (470.29 MHz, 305.0 K, C_6D_6): δ = -119.4 (s, $^{TS}J_{Si,F}$ = 5.7 Hz). **IR** (ATR, neat): $\tilde{\nu}$(SiH) = 2113 cm^{-1}. **HR/MS** (EI): [$^{12}C_{14}{}^{1}H_{15}{}^{19}F\,{}^{28}Si$], calculated 230.093; measured: 230.0924. **EA**: $C_{14}H_{15}FSi$, calculated: C 73.00, H 6.56; measured: C 73.41, H 6.82.

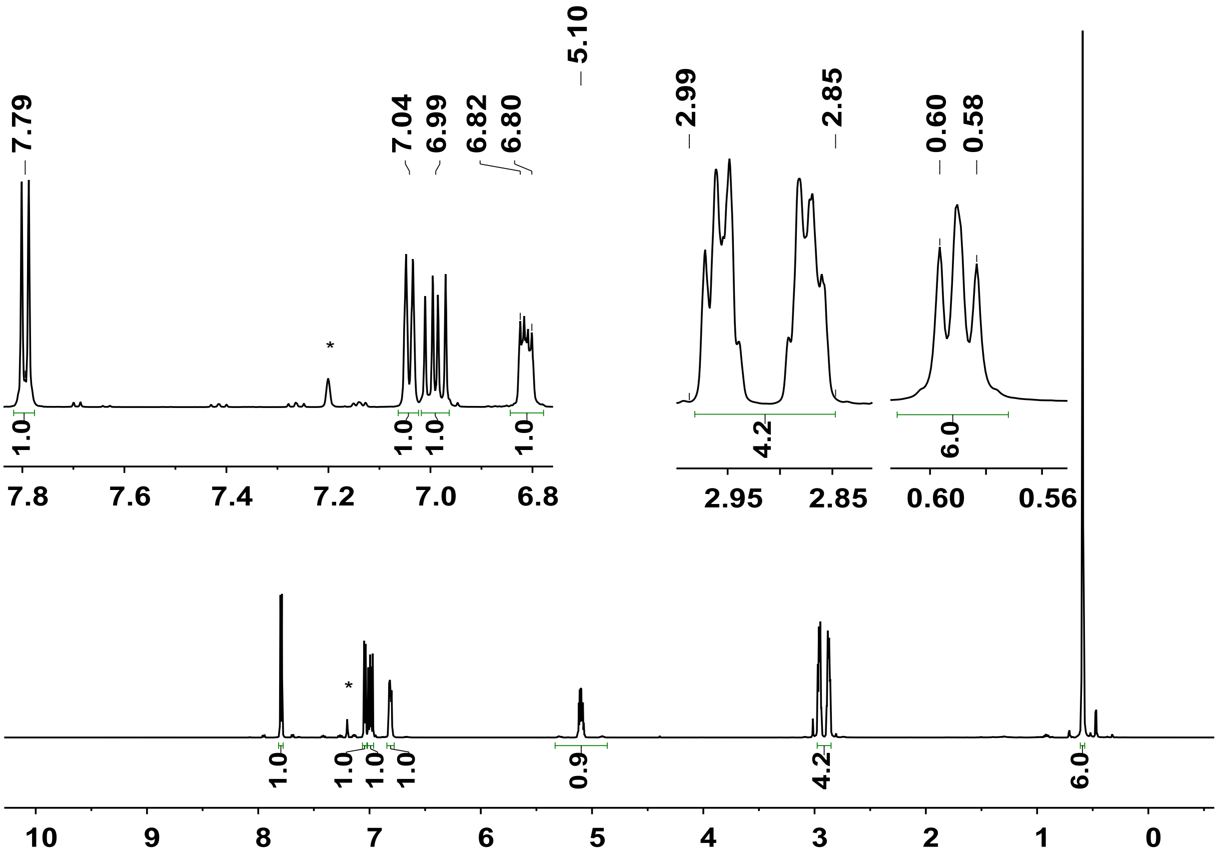

Figure S1 – ^{1}H NMR spectrum (499.87 MHz, 305.0 K, C_6D_6) of silane **1a** (*C_6D_5H).

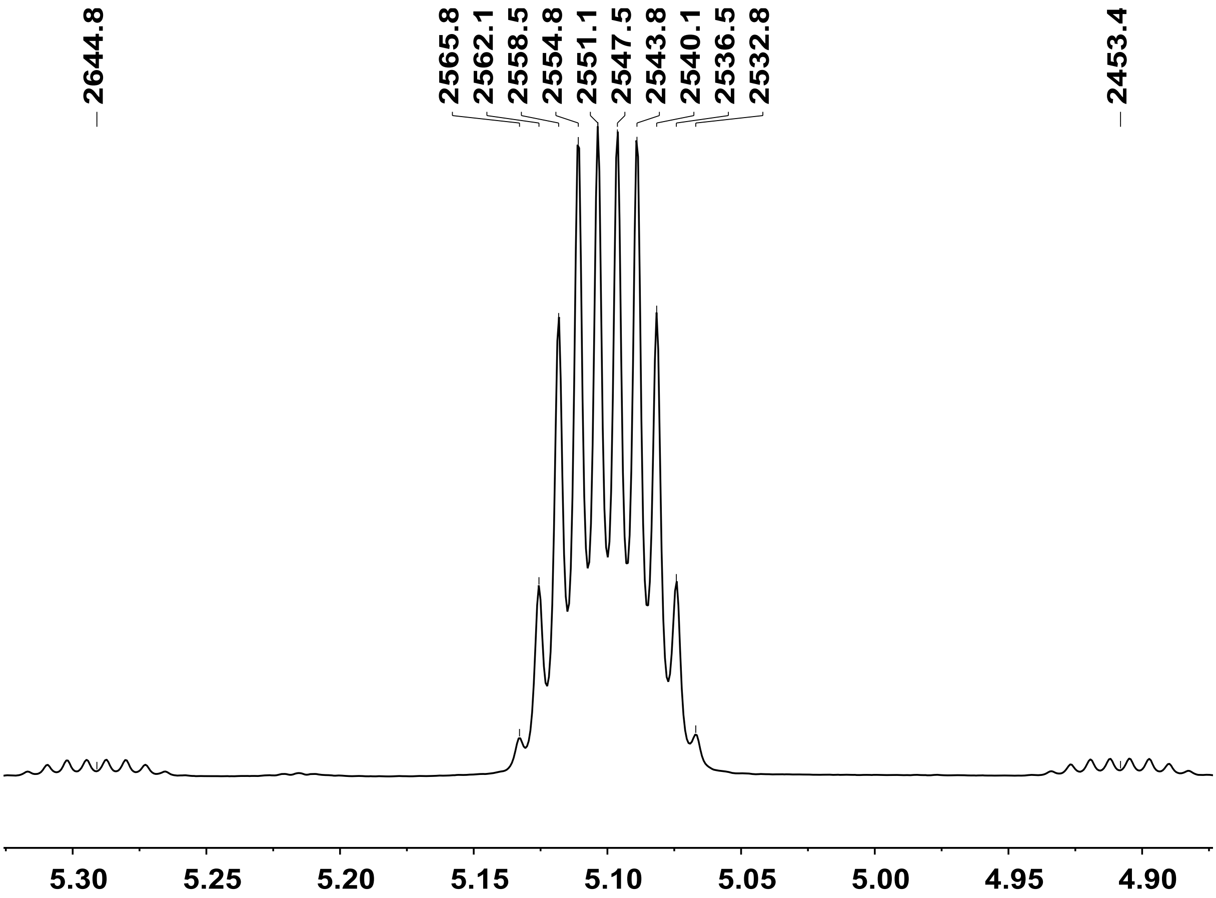

Figure S2 – Part of the ^{1}H NMR spectrum (499.87 MHz, 305.0 K, C_6D_6) of silane **1a** including the signal of Si–H.

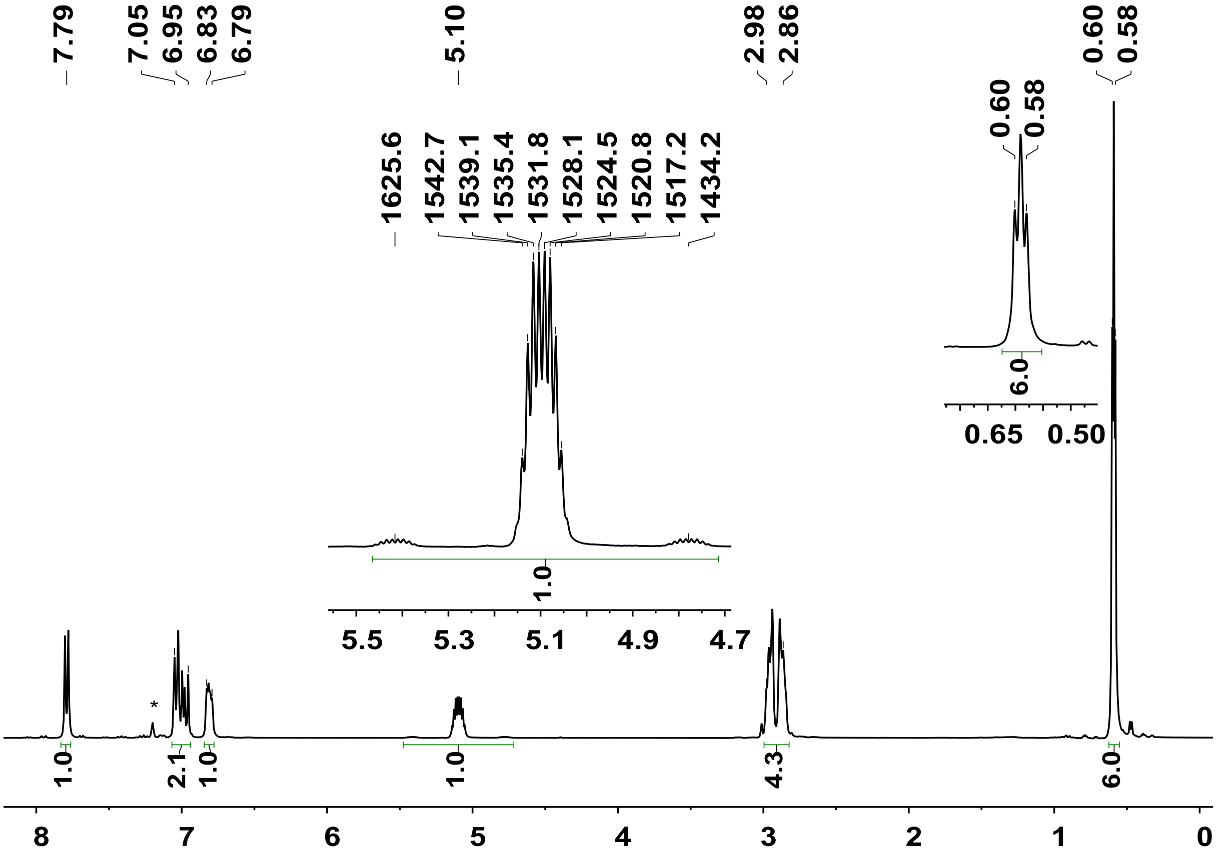

Figure S3 – ^{1}H NMR spectrum (300.14 MHz, 305.2 K, C_6D_6) of silane **1a** (*C_6D_5H).

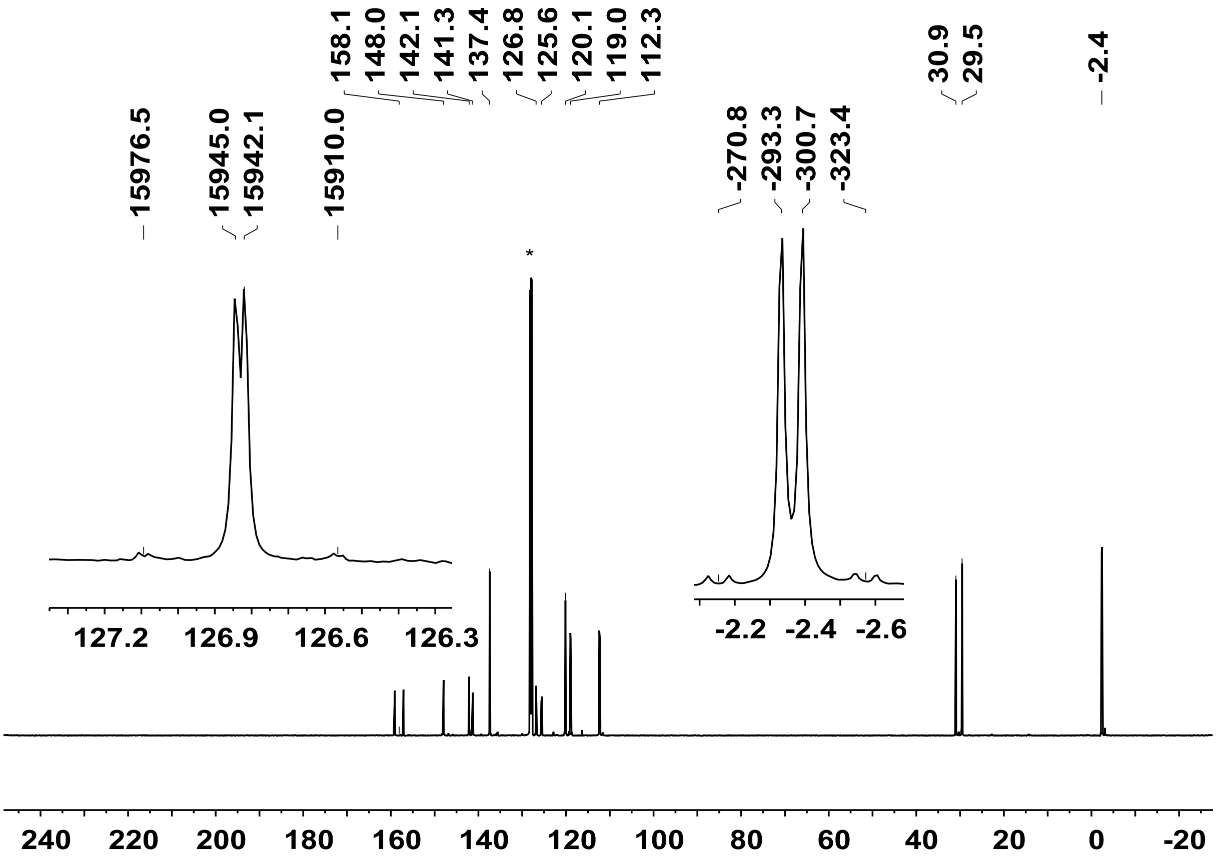

Figure S4 – $^{13}C\{^1H\}$ NMR spectrum (125.71 MHz, 305.0 K, C_6D_6) of silane **1a** (*C_6D_6).

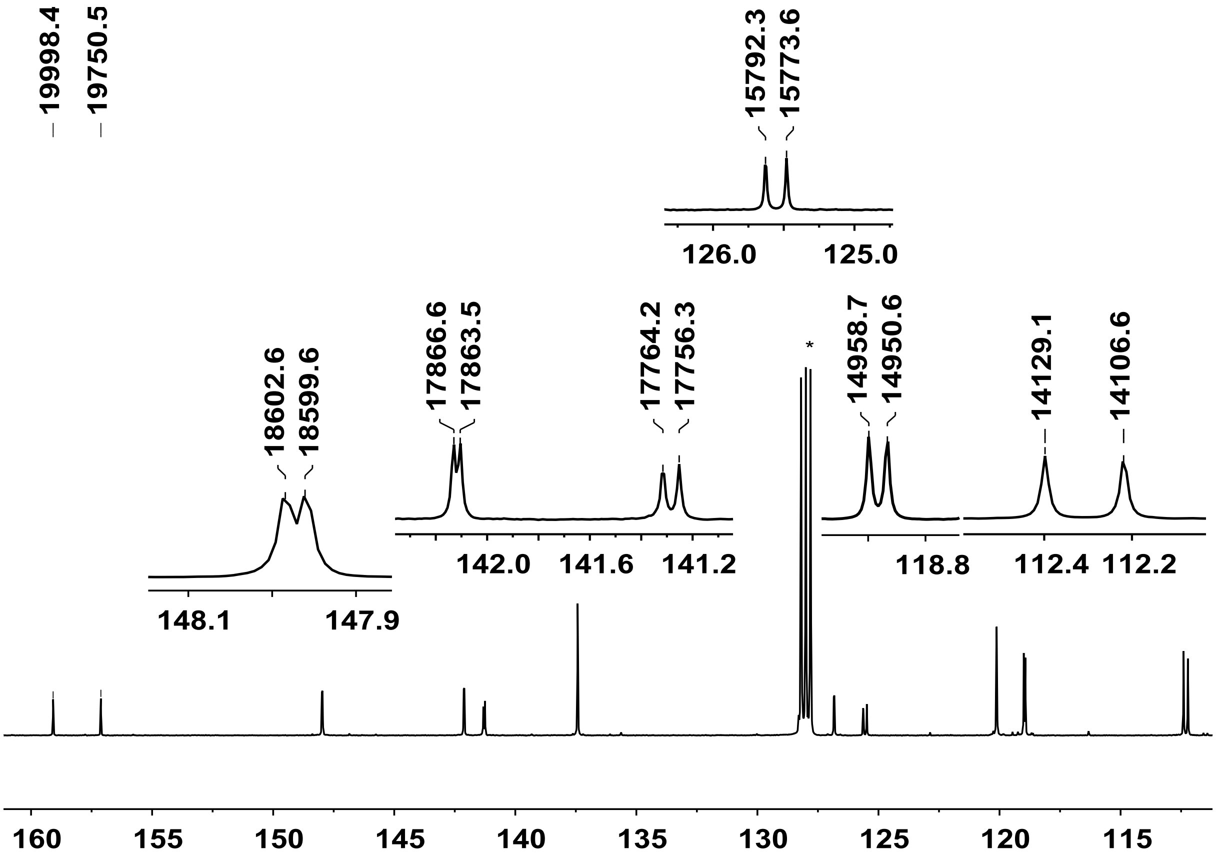

Figure S5 – Part of the $^{13}C\{^1H\}$ NMR spectrum (125.71 MHz, 305.0 K, C_6D_6) of silane **1a** (*C_6D_6).

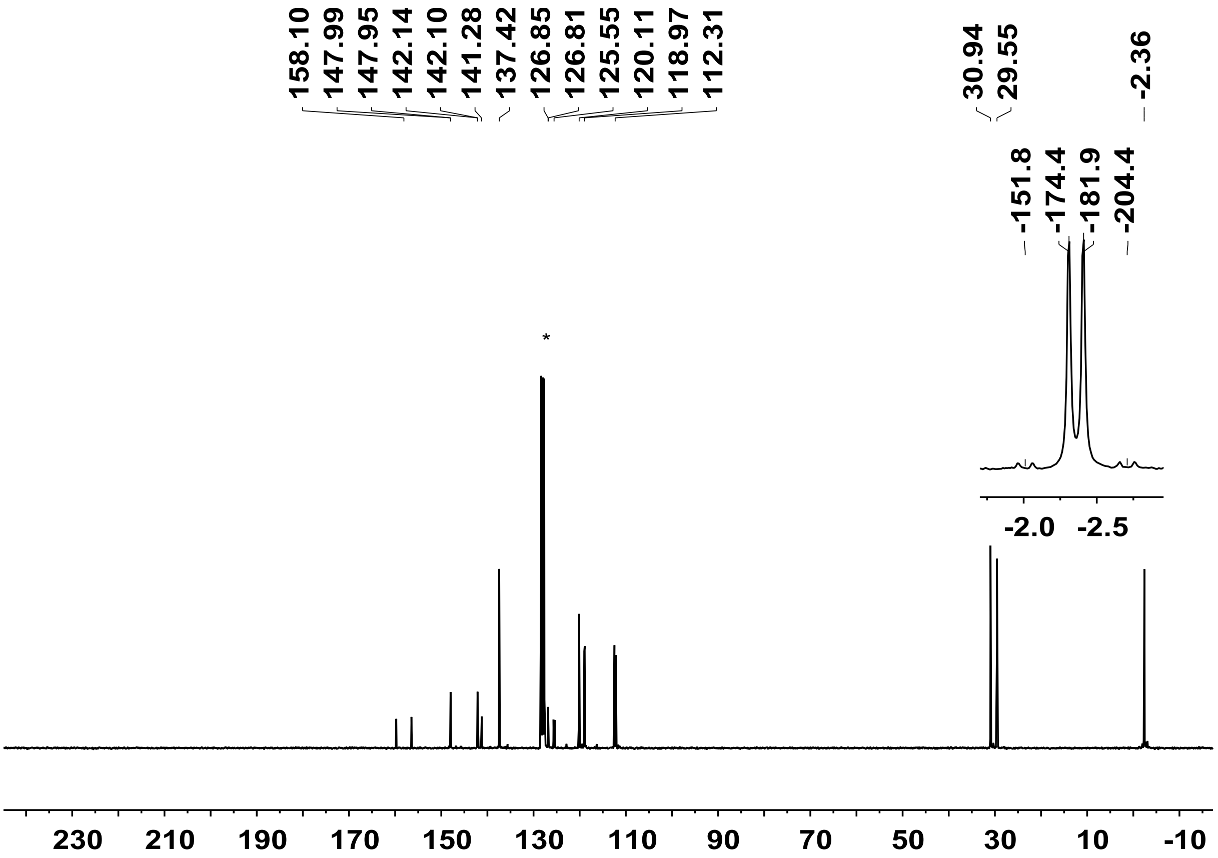

Figure S6 – $^{13}C\{^{1}H\}$ NMR spectrum (75.48 MHz, 305.0 K, C_6D_6, *C_6D_6) of silane **1a** (*C_6D_6).

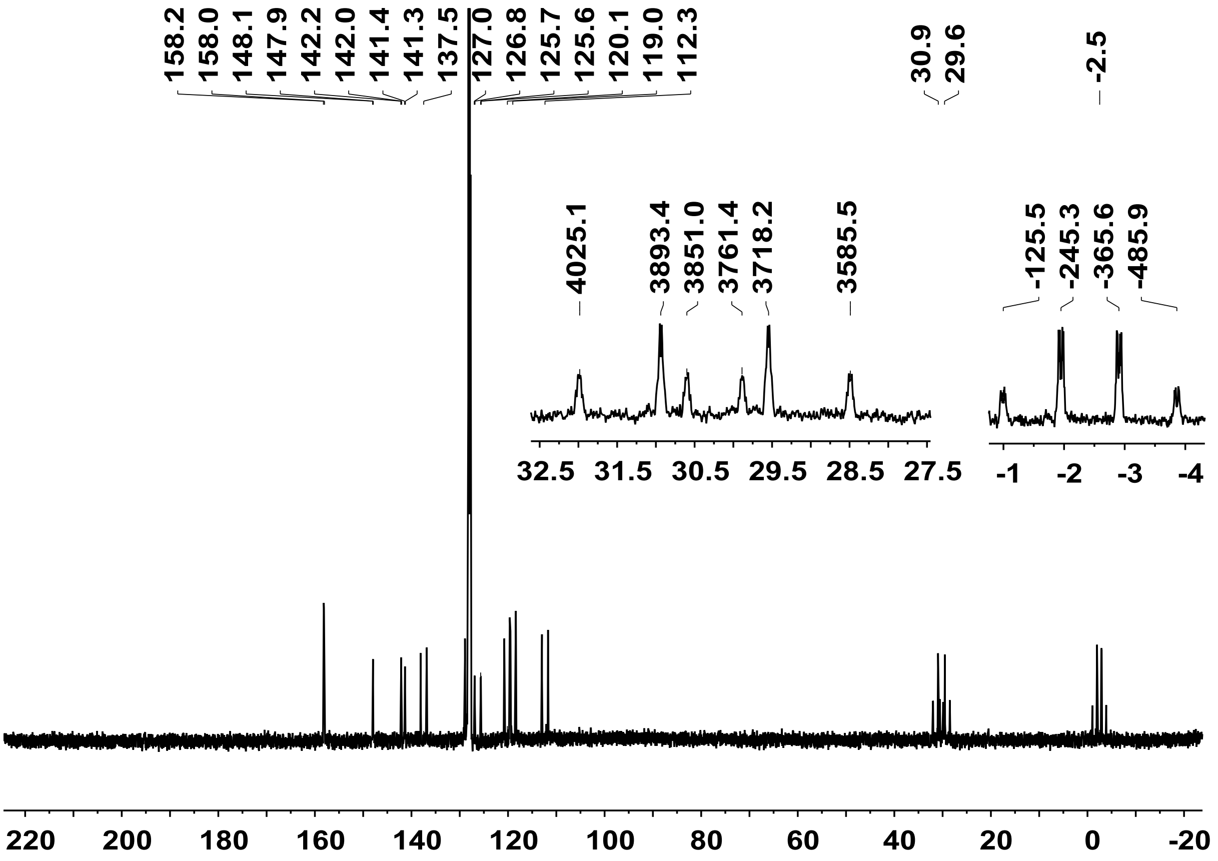

Figure S7 – $^{13}C\{^{19}F\}$ NMR spectrum (125.85 MHz, 303.1 K, C_6D_6) of silane **1a.**

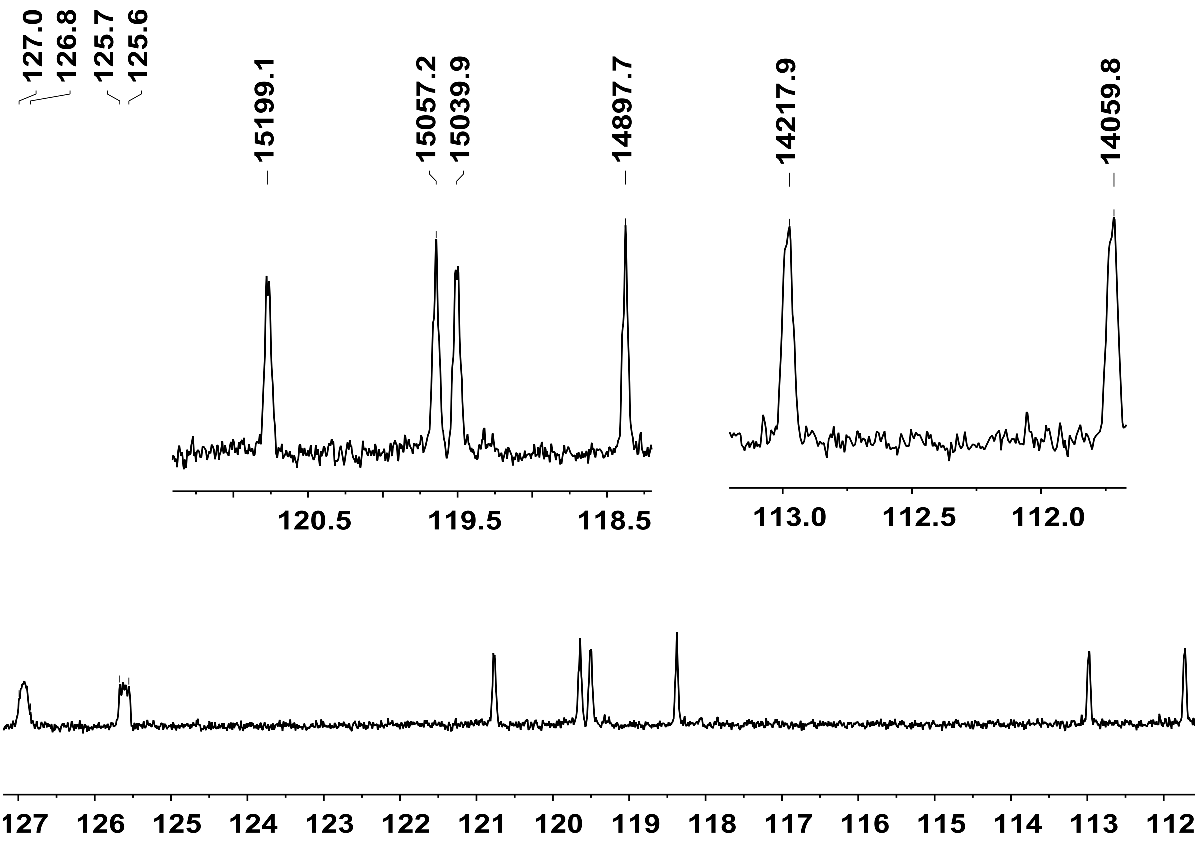

Figure S8 – Part of the $^{13}C\{^{19}F\}$ NMR spectrum (125.85 MHz, 303.1 K, C_6D_6) in the range of 112-127 ppm of silane **1a**.

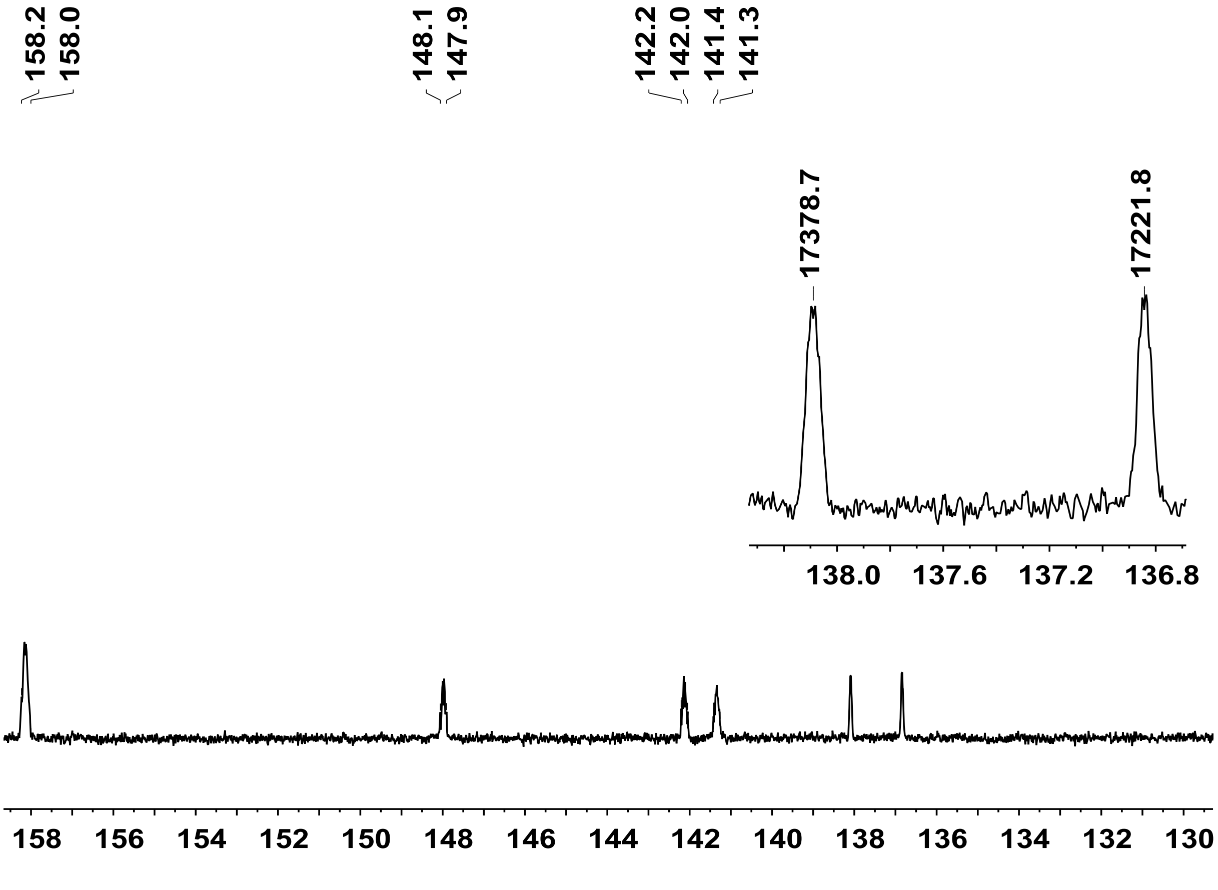

Figure S9 – Part of the $^{13}C\{^{19}F\}$ NMR spectrum (125.85 MHz, 303.1 K, C_6D_6) in the range of 130-159 ppm of silane **1a**.

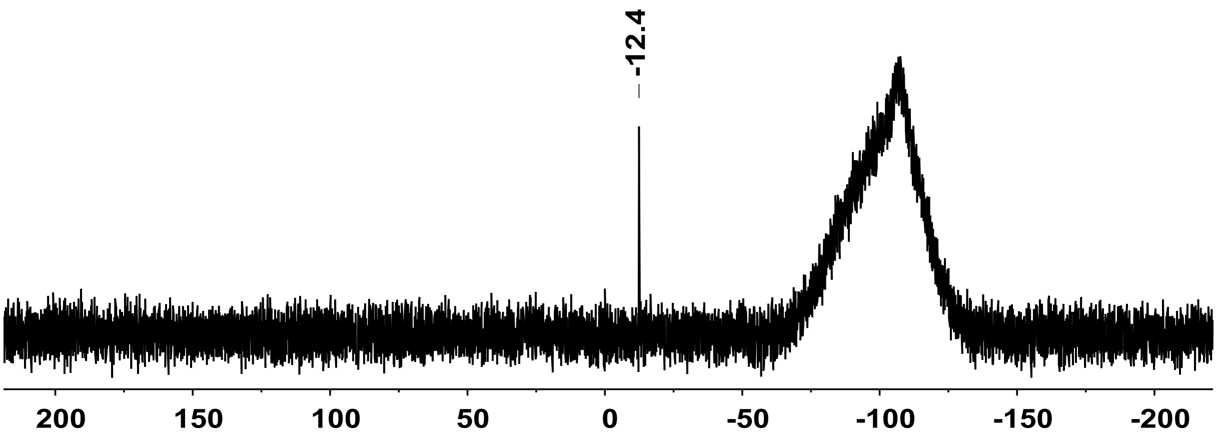

Figure S10 – $^{29}Si\{^1H\}$ NMR spectrum (99.31MHz, 305.0 K, C_6D_6) of silane **1a**.

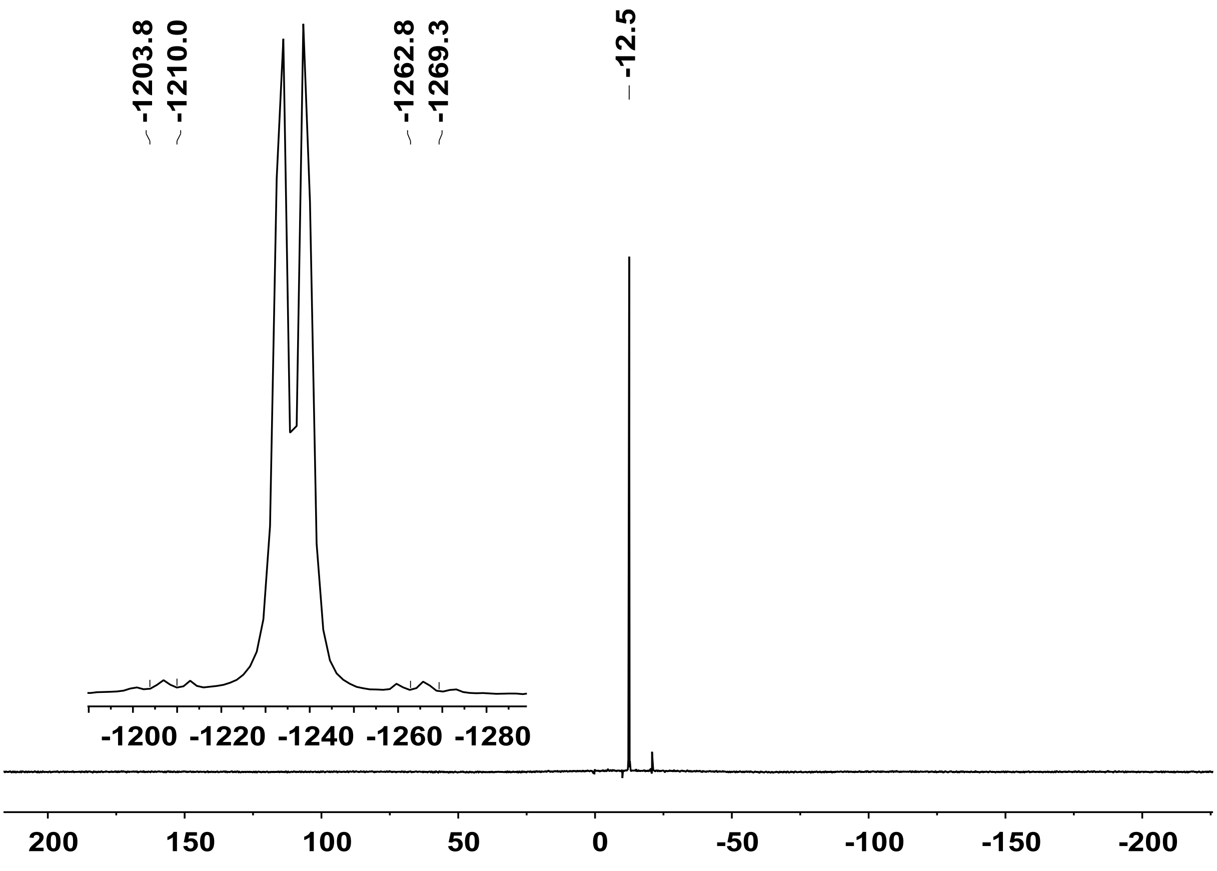

Figure S11 – $^{29}Si\{^1H\}$ INEPT NMR spectrum (99.31MHz, 305.0 K, C_6D_6) of silane **1a**.

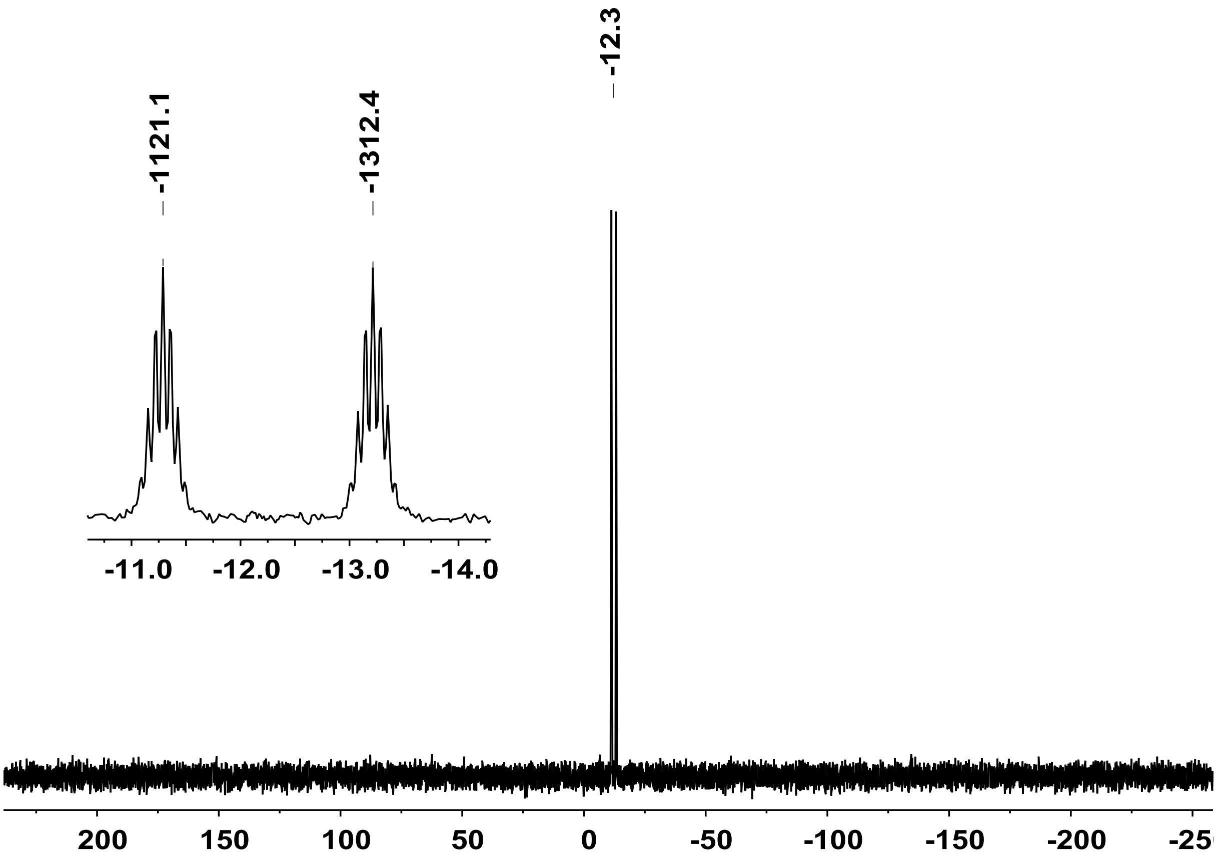

Figure S12 – ^{29}Si INEPT NMR spectrum (99.31MHz, 305.0 K, C_6D_6) of silane **1a**; optimized for Si-H.

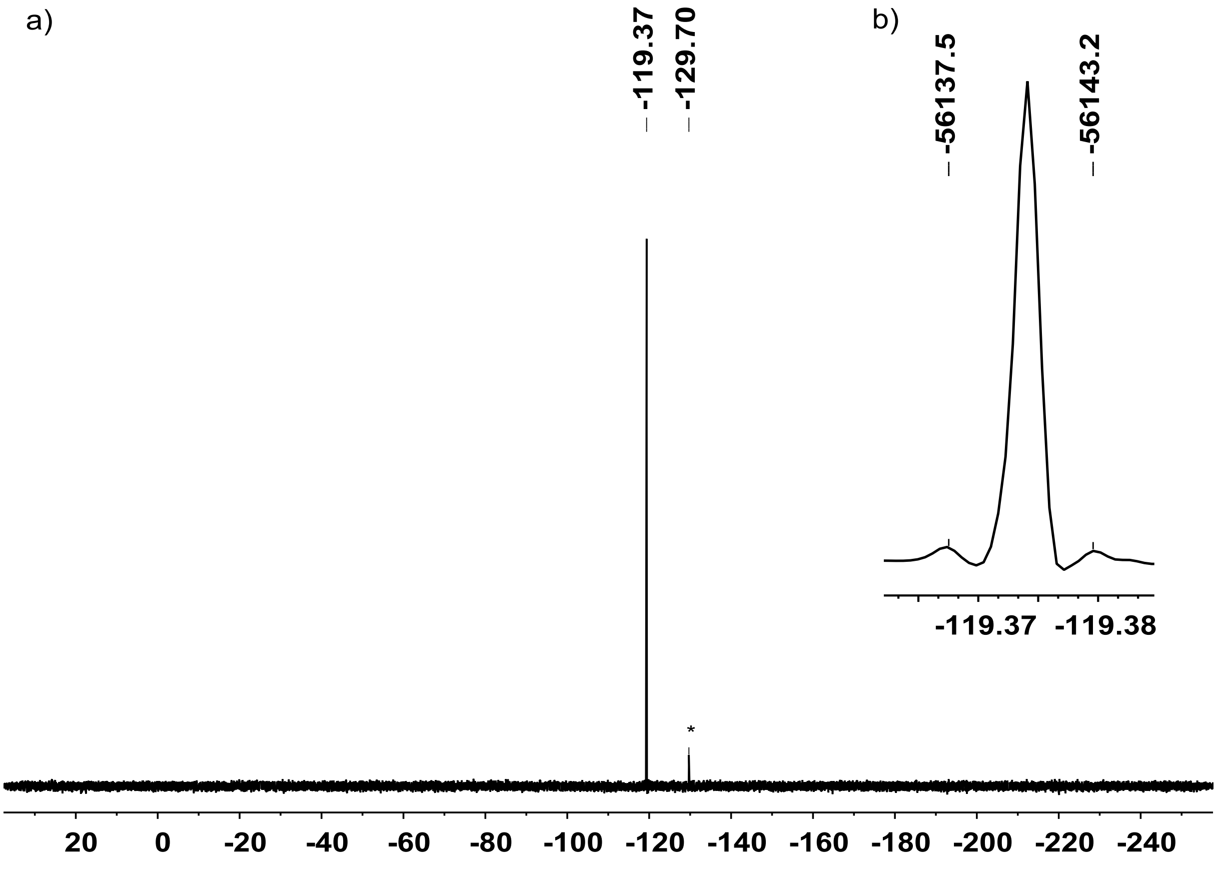

Figure S13 – a) $^{19}F\{^1H\}$ NMR spectrum (470.30 MHz, 305.1 K, C_6D_6) of silane **1a** (lb = 0.3, gb = 1.0, digital resolution = 2.1 Hzpt^{-1}, *5-fluoroacenaphthene); b) $^{19}F\{^1H\}$ NMR spectrum (470.29 MHz, 305.0 K, C_6D_6) of silane **1a** (lb = -0.8, gb = 0.7, digital resolution = 0.3 Hzpt^{-1}).

5-chloro-6-dimethylsilylacenaphtene ***1b***

The synthesis of the title compound was performed according to general procedure **A** using 5-bromo-6-chloroacenaphthene (1.0 equiv, 0.96 g, 3.59 mmol), *n*BuLi (1.6 M in *n*-hexane, 1 equiv, 2.3 mL, 3.59 mmol) and chlorodimethylsilane (1.0 equiv, 0.4 mL, 3.59 mmol). Silane **1b** was obtained in a reaction mixture with 5-chloroacenaphthene **17** (silane **1b** 87%, 5-chloroacenaphthene **17** 13 %) as a yellow oil after column chromatography using petroleum ether 40/60 (R_f = 0.53) as eluent. Yield: 0.83 g, 3.38 mmol, 94 %.

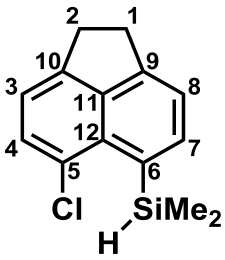

1b

^{1}H NMR (499.87 MHz, 305.1 K, C_6D_6): δ = 0.65 (d, 6H, $^3J_{H,H}$ = 3.7 Hz, $Si(CH_3)_2$), 2.81-2.94 (m, 4H, CH_2, H-1, H-2), 5.37 (sept, 1H, $^3J_{H,H}$ = 3.7 Hz, $^1J_{H,Si}$ = 194.8 Hz, Si-H), 6.78-6.80 (m, 1H, H-3), 7.03-7.05 (m, 1H, H-8), 7.42 (d, 1H, $^3J_{H,H}$ = 7.4 Hz, H-4), 7.95 (d, 1H, $^3J_{H,H}$ = 6.8 Hz, H-7). **^{13}C{^{1}H} NMR** (125.71 MHz, 305.0 K, C_6D_6): δ = 0.1 ($Si(CH_3)_2$), 29.6 (CH_2, C-2), 30.2 (CH_2, C-1), 119.9 (CH), 120.0 (CH), 128.5 (C, C-5), 129.3 (CH, C-4), 130.0 (C, C-6), 134.3 (C, C-12), 139.6 (CH, C-7), 141.2 (C, C-11), 146.1 (C, C-10), 149.0 (C, C-9).**^{29}Si{^{1}H} NMR** (99.31 MHz, 305.0 K, C_6D_6): δ = -12.7. **^{29}Si{^{1}H} INEPT NMR** (99.31 MHz, 305.0 K, C_6D_6): δ = -12.7 ($^1J_{Si,C}$ = 52.7 Hz ($Si(CH_3)_2$), $^1J_{Si,C}$ = 67.3 Hz (Si–C-6)) **^{29}Si INEPT NMR** (99.31 MHz, 305.0 K, C_6D_6): δ = -12.6 (dm, $^1J_{Si,H}$ = 194.6 Hz). **IR** (ATR, neat): $\tilde{\nu}$(SiH) = 2141, 2106 cm^{-1}. **HR/MS** (EI): [$^{12}C_{14}{}^1H_{15}{}^{35}Cl^{28}Si$], calculated 246.063; measured: 246.0609.

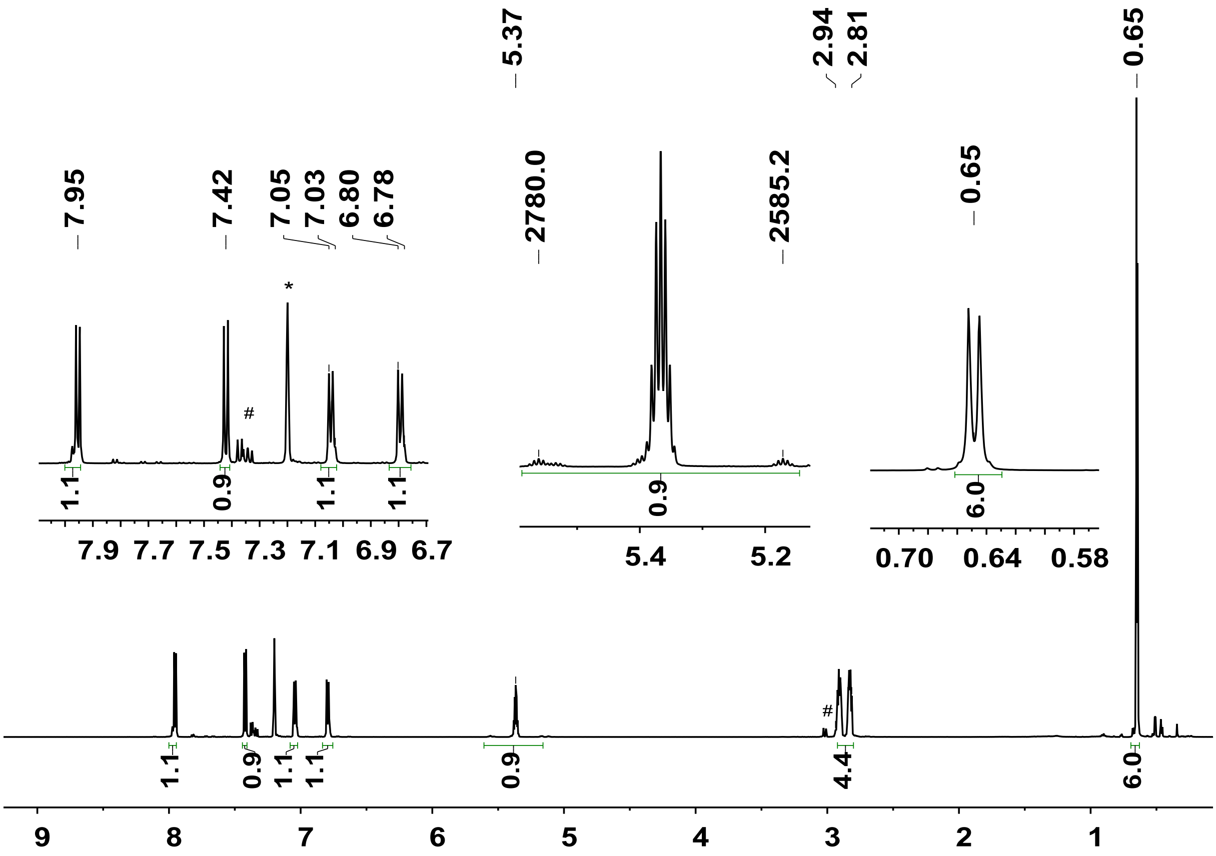

Figure S14 – ^{1}H NMR spectrum (499.87 MHz, 305.1 K, C_6D_6) of silane **1b** ($^{*}C_6D_5H$, $^{\#}$ 5-chloroacenaphthene **17**).

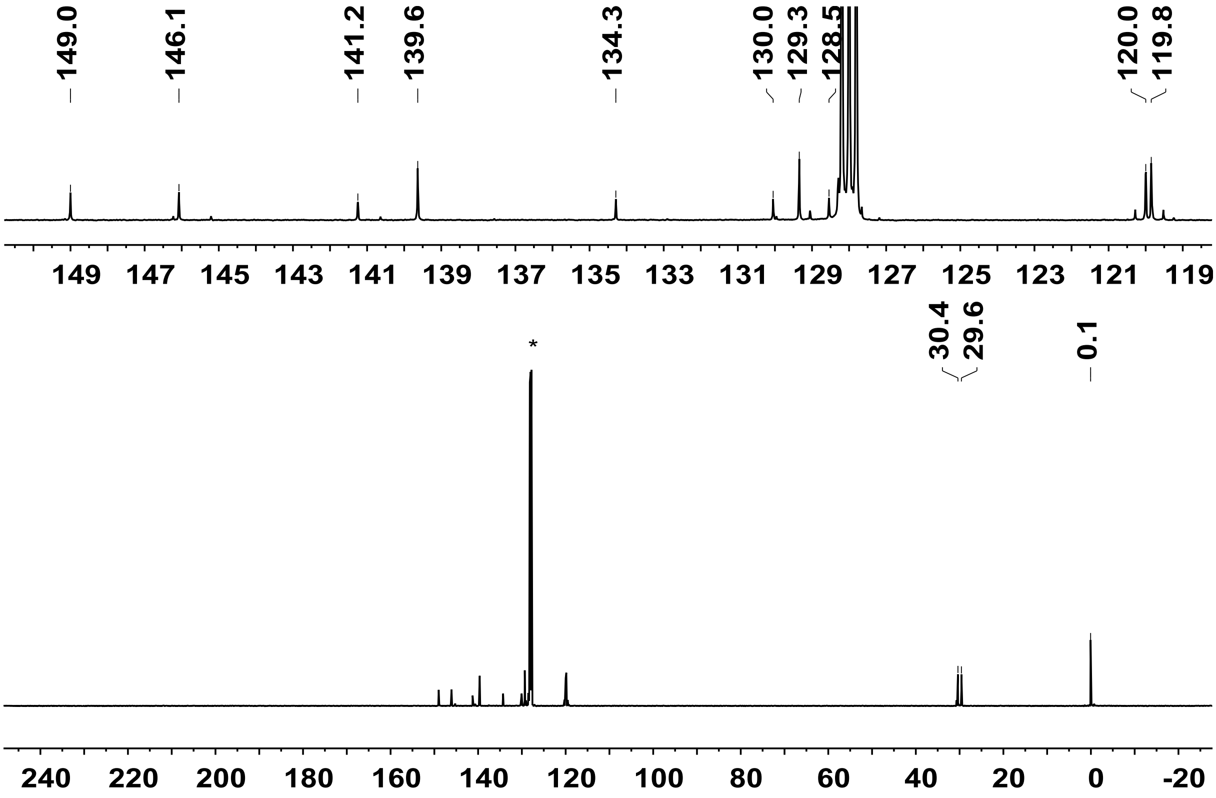

Figure S15 – $^{13}C\{^{1}H\}$ NMR spectrum (125.71 MHz, 305.0 K, C_6D_6) of silane **1b** ($^{*}C_6D_6$).

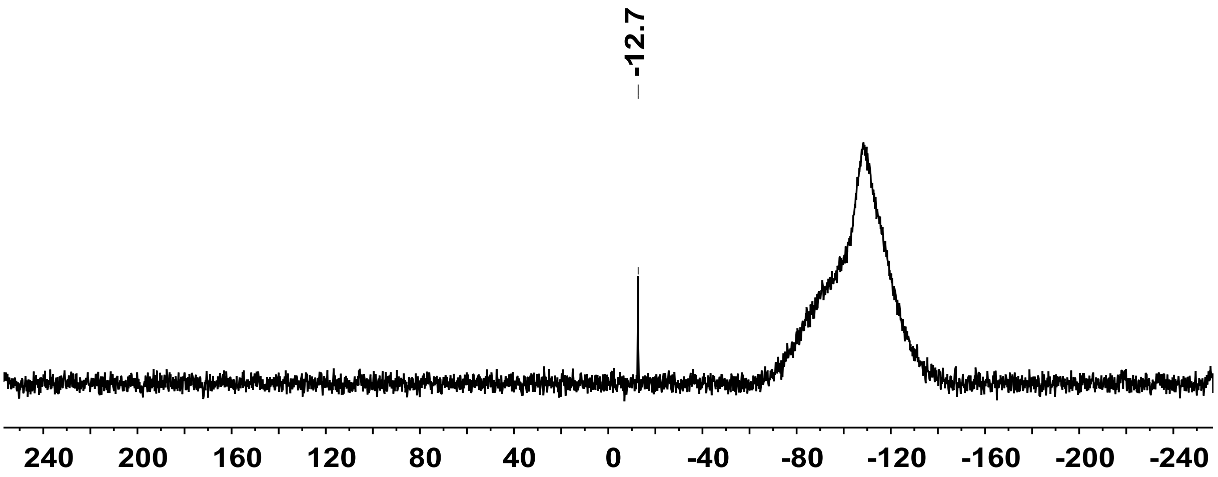

Figure S16 – $^{29}Si\{^{1}H\}$ NMR spectrum (99.31MHz, 305.0 K, C_6D_6) of silane **1b**.

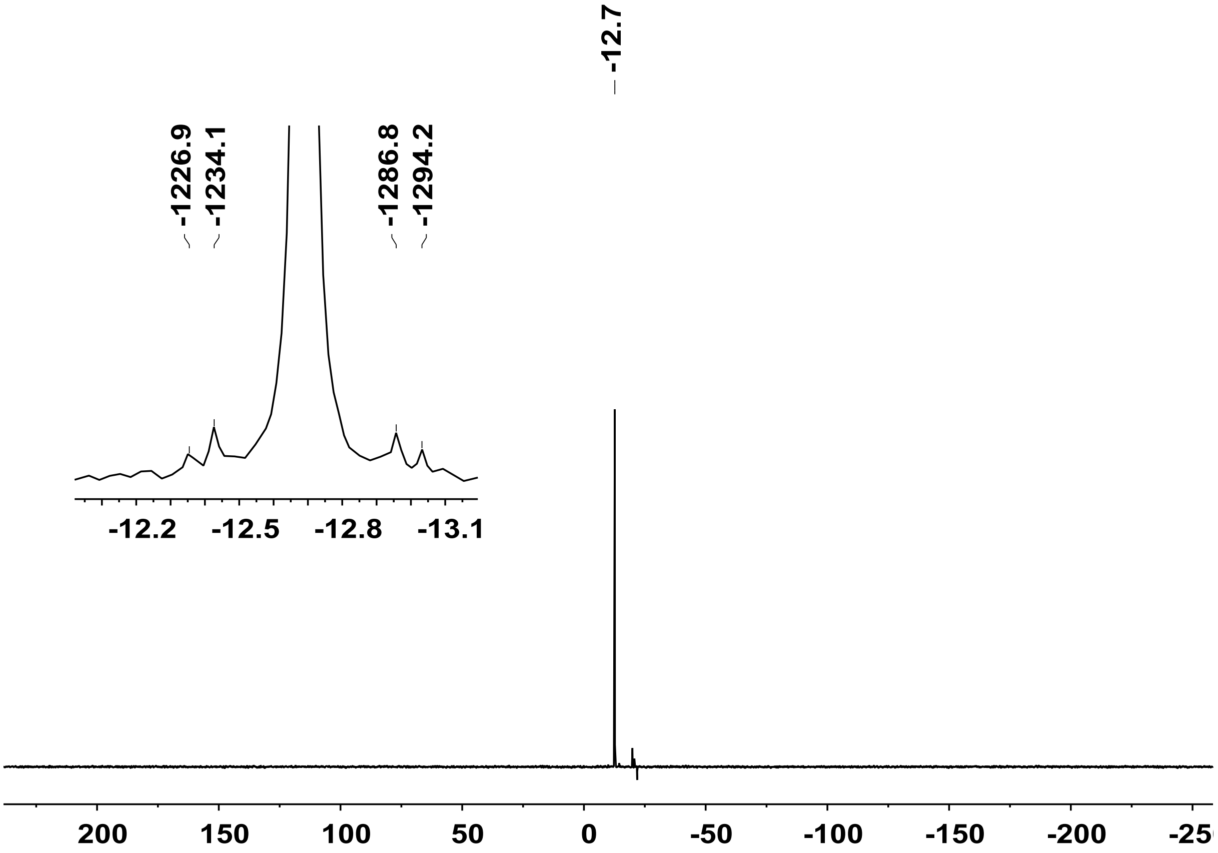

Figure S17 – $^{29}Si\{^{1}H\}$ INEPT NMR spectrum (99.31MHz, 305.0 K, C_6D_6) of silane **1b**.

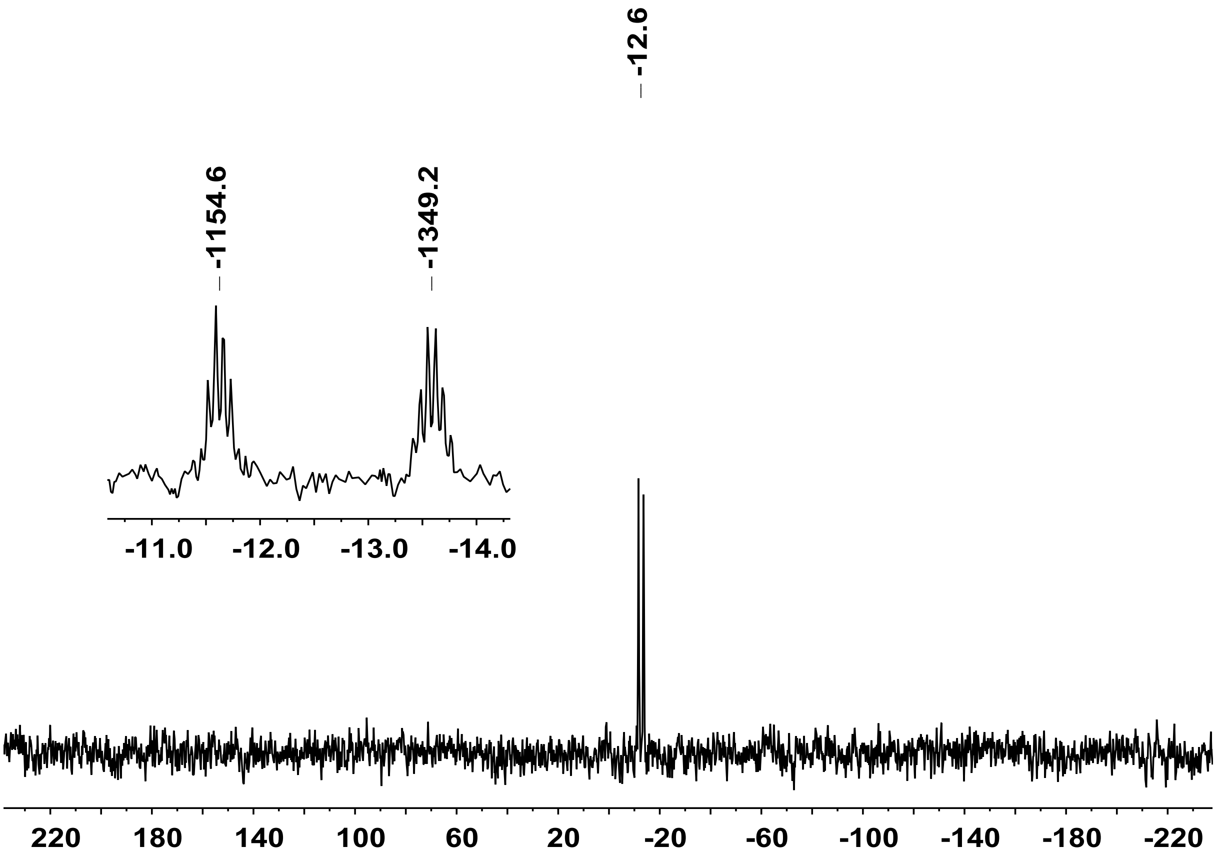

Figure S18 – ^{29}Si INEPT NMR spectrum (99.31MHz, 305.0 K, C_6D_6) of silane **1b**; optimized for Si-H.

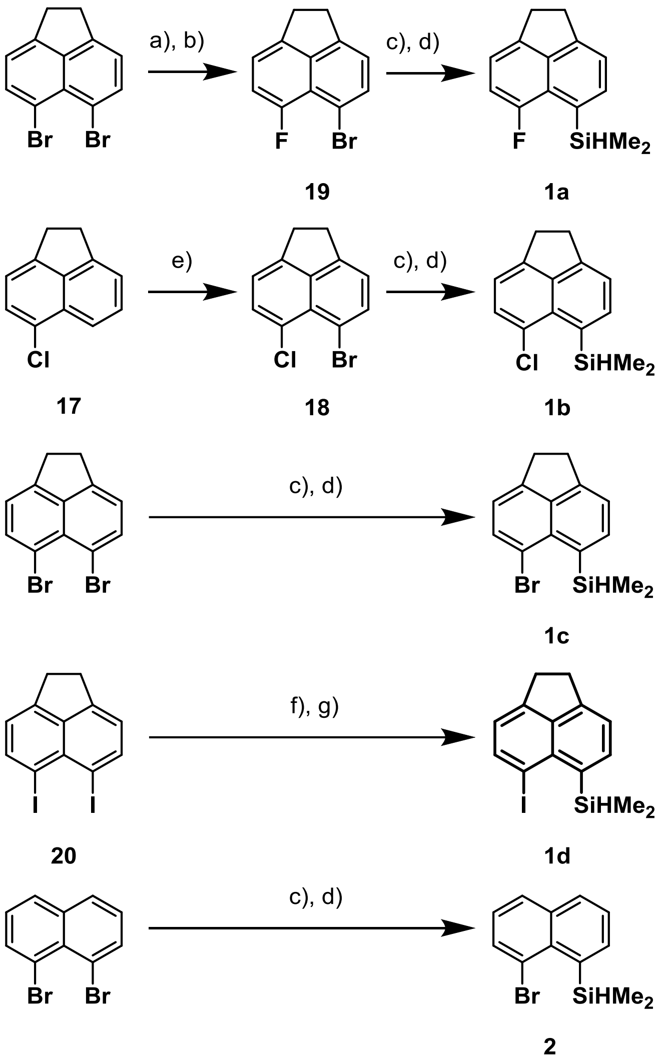

Scheme S1 - Synthesis of precursor silanes **1**. a) 1.05 equiv *n*BuLi, THF, -80 °C; b) 1.2 equiv NFSI, THF, -80 °C; c) 1 equiv *n*BuLi, THF, -80 °C; d) 1 equiv Me_2SiHCl, THF, -80 °C; e) 1 equiv NBS, DMF, 0 °C; f) 1 equiv *n*BuLi, Et_2O, -80 °C; g) 1 equiv Me_2SiHCl, Et_2O, -50 °C.

3 Synthesis of the halonium and siliconium ions

*chloronium ion **3b** and siliconium ion **4b***

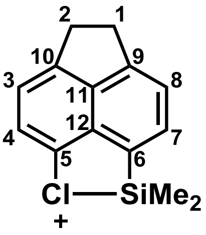

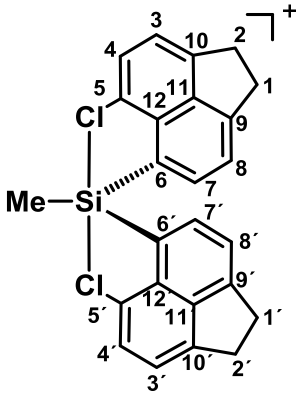

3b $[B(C_6F_5)_4]^-$ **4b** $[B(C_6F_5)_4]^-$

A solution of trityl borate $[Ph_3C][B(C_6F_5)_4]$ (1.0 equiv, 434 µmol, 400 mg) in benzene-d$_6$ was added to a solution of 5-chloro-6-dimethylsilylacenaphthene **1b** (1.0 equiv, 434 µmol, 107 mg) in benzene-d$_6$. During the addition, the reaction mixture was cooled with cold water and stirred for 30 min at room temperature. The color of the reaction mixture changed from yellow orange to dark green. Then, the upper, nonpolar phase was removed and the polar phase was analyzed by NMR spectroscopy, which indicated the formation of chloronium borate **3b**$[B(C_6F_5)_4]$ and siliconium borate **4b**$[B(C_6F_5)_4]$ in the ratio of 40:60 and full conversion of the trityl cation. Small amounts of $B(C_6F_5)_3$ were also detected.
The inverse mode of addition, that is addition of 5-chloro-6-dimethylsilylacenaphthene **1b** in benzene to a solution of trityl borate $[Ph_3C][B(C_6F_5)_4$ in benzene at room temperature still resulted in a mixture, containing chloronium borate **3b**$[B(C_6F_5)_4]$ and siliconium borate **4b**$[B(C_6F_5)_4]$ in a comparable ratio.

^{1}H NMR (499.87 MHz, 305.1 K, C_6D_6): δ = 0.60 (s, $Si(CH_3)_2$, **3b**), 1.22 (s, $SiCH_3$, **4b**), 2.88-3.10 (m, CH_2, **3b** and **4b**), 6.90 (d, $^3J_{H,H}$ = 7.8 Hz, H-3, **3b**), 6.96 (d, $^3J_{H,H}$ = 7.8 Hz, H-3, H-3´, **4b**), 6.98 (d, $^3J_{H,H}$ = 7.8 Hz, H-4, **3b**), 7.11-7.18 (m, H-8, **3b** and H-8, H-8´, **4b**), 7.24 (d, $^3J_{H,H}$ = 7.8 Hz, H-4, H-4´, **4b**), 7.34 (d, $^3J_{H,H}$ = 7.2 Hz, H-7, **3b**), 7.99 (d, $^3J_{H,H}$ = 7.3 Hz, H-7, H-7´, **4b**). **^{13}C{^{1}H} NMR** (125.71 MHz, 305.0 K, C_6D_6): δ = 2.5 ($Si(CH_3)_2$, **3b**), 10.2 ($SiCH_3$, **4b**), 30.1 (CH_2, C-2, C-2´, **4b**), 30.2 (CH_2, C-2, **3b**), 31.1 (CH_2, C-1, C-1´, **4b**), 31.6 (CH_2, C-1, **3b**), 117.9 (C, C-6, C-6´, **4b**), 118.5 (C, C-6, **3b**), 121.7 (CH, **4b**), 121.8(CH, C-3, **3b**), 122.0 (CH, **4b**), 122.8 (CH, C-4, **3b**), 123.7 (C, C-5, C-5´, **4b**), 124.0 (CH), 124.9 (brs, C, $[B(C_6F_5)_4]^-$), 125.8 (CH, C-4, C-4´ **4b**), 127.2 (C, C-5, **3b**), 131.1 (C, C-12, **3b**), 132.3 (C, C-12, C-12´, **4b**), 135.8

(CH, C-7, **3b**), 137.1 (dm, $^{1}J_{C,F}$ = 241.0 Hz, CF, $[B(C_6F_5)_4]^-$), 139.0 (dm, $^{1}J_{C,F}$ = 240.2 Hz, CF, $[B(C_6F_5)_4]^-$), 139.7 (CH, C-7, C-7´, **4b**), 140.2 (C, C-11, **3b**), 140.7 (C, C-11, C-11´, **4b**), 149.2 (dm, $^{1}J_{C,F}$ = 241.4 Hz, CF, $[B(C_6F_5)_4]^-$), 149.5 (C, C-10, C-10´, **4b**), 150.5 (C, C-10, **3b**), 153.2 (C, C-9, **3b**), 154.1 (C, C-9, C-9´, **4b**). **^{29}Si{^{1}H} NMR** (99.31 MHz, 305.0 K, C_6D_6): δ = 79.8 (**4b**), 115.9-116.4 (brs, **3b**). **^{29}Si{^{1}H} INEPT NMR** (99.31 MHz, 305.0 K, C_6D_6):): δ = 79.8 (**4b**), 117.0 (**3b**). **^{19}F{^{1}H} NMR** (470.30 MHz, 305.0 K, C_6D_6): δ = -166.7-(-166.4) (m, 8 F, *m*-F, $[B(C_6F_5)_4]^-$), -162.6 (t, $^{3}J_{F,F}$ = 21.0 Hz, 4 F, *p*-F, $[B(C_6F_5)_4]^-$), -132.3-(-132.0) (m, 8 F, *o*-F, $[B(C_6F_5)_4]^-$). **^{11}B{^{1}H} NMR** (160.38 MHz, 305.0 K, C_6D_6): δ = -16.0.

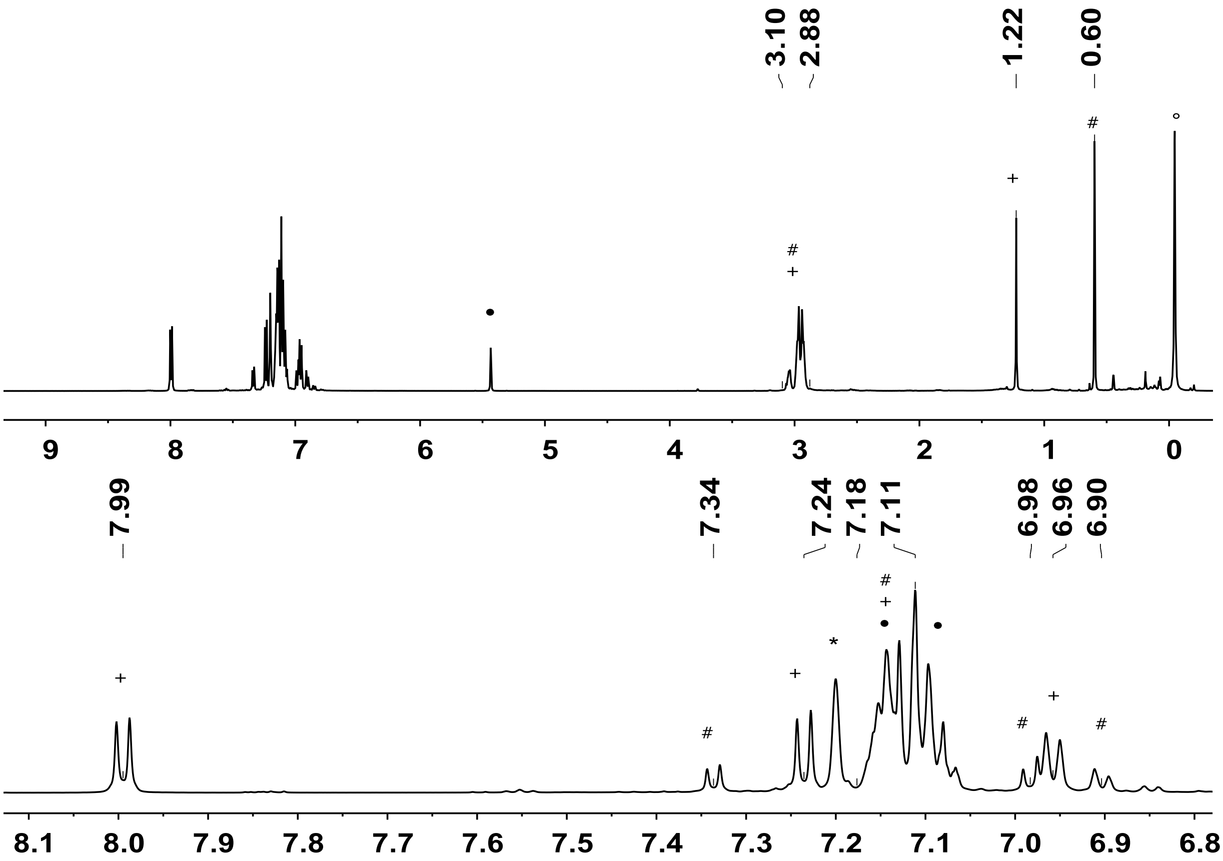

Figure S19 – ^{1}H NMR spectrum (499.87 MHz, 305.1 K, C_6D_6) of chloronium borate **3b**$[B(C_6F_5)_4]$ and siliconium borate **4b**$[B(C_6F_5)_4]$ (* C_6D_5H, # chloronium ion **3b**, + siliconium ion **4b**, • triphenylmethane, °impurities possibly due to side reactions).

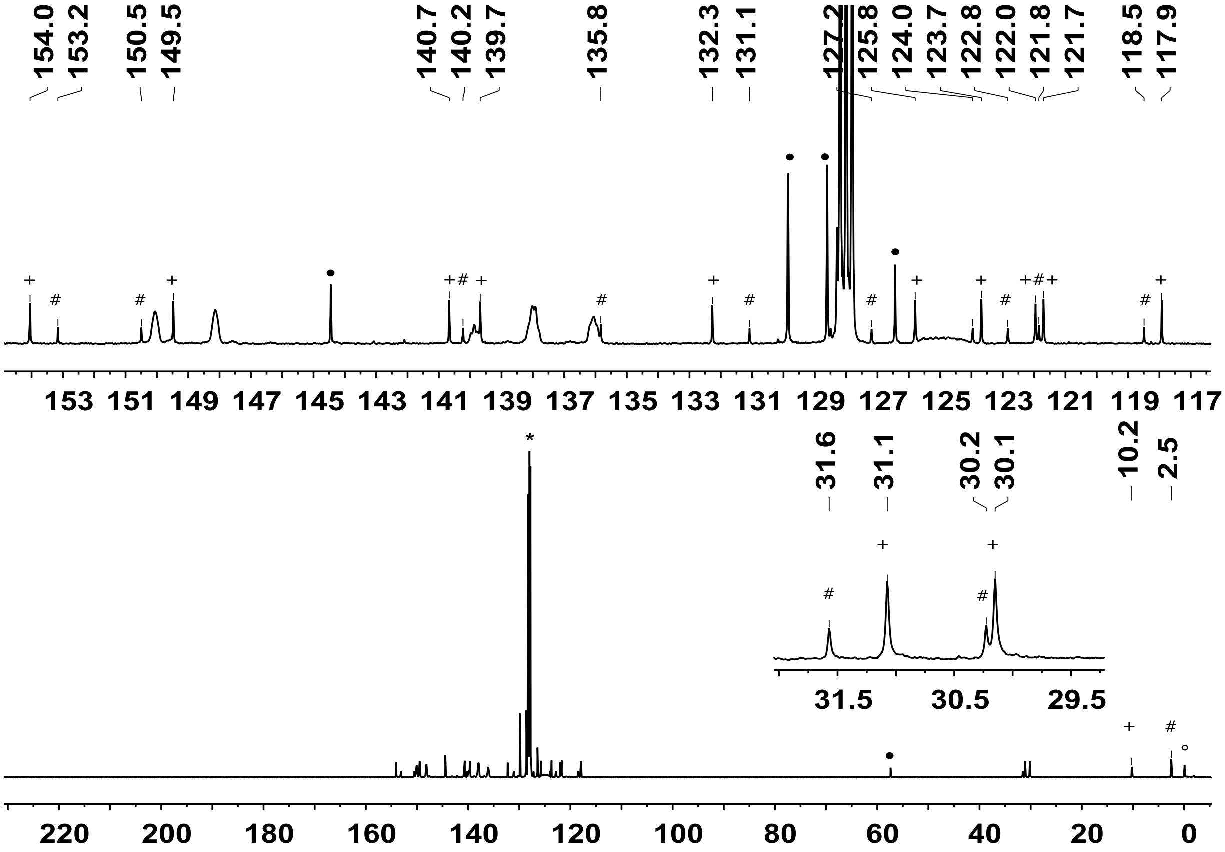

Figure S20 – $^{13}C\{^{1}H\}$ NMR spectrum (125.71 MHz, 305.0 K, C_6D_6) of chloronium borate **3b**[$B(C_6F_5)_4$] and siliconium borate **4b**[$B(C_6F_5)_4$] (* C_6D_5H, # chloronium ion **3b**, + siliconium ion **4b**, • triphenylmethane, °impurities possibly due to side reactions).

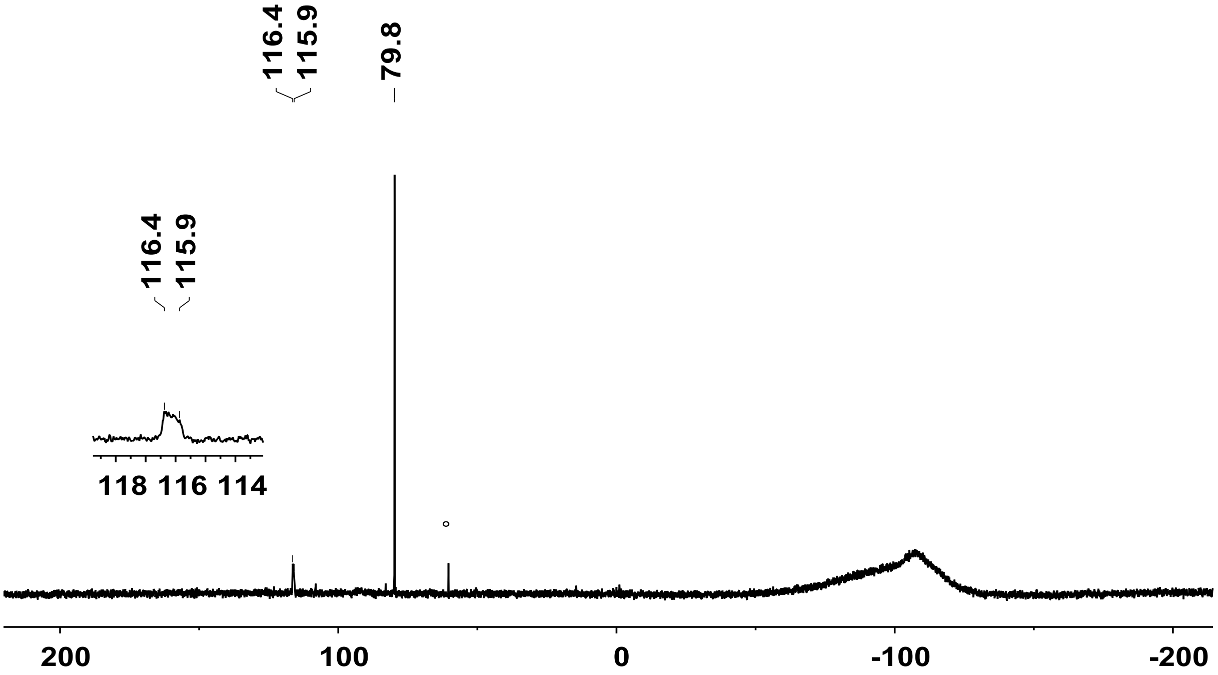

Figure S21 – $^{29}Si\{^{1}H\}$ NMR spectrum (99.31 MHz, 305.0K, C_6D_6) of chloronium borate **3b**[$B(C_6F_5)_4$] and siliconium borate **4b**[$B(C_6F_5)_4$] (°impurities possibly due to side reactions).

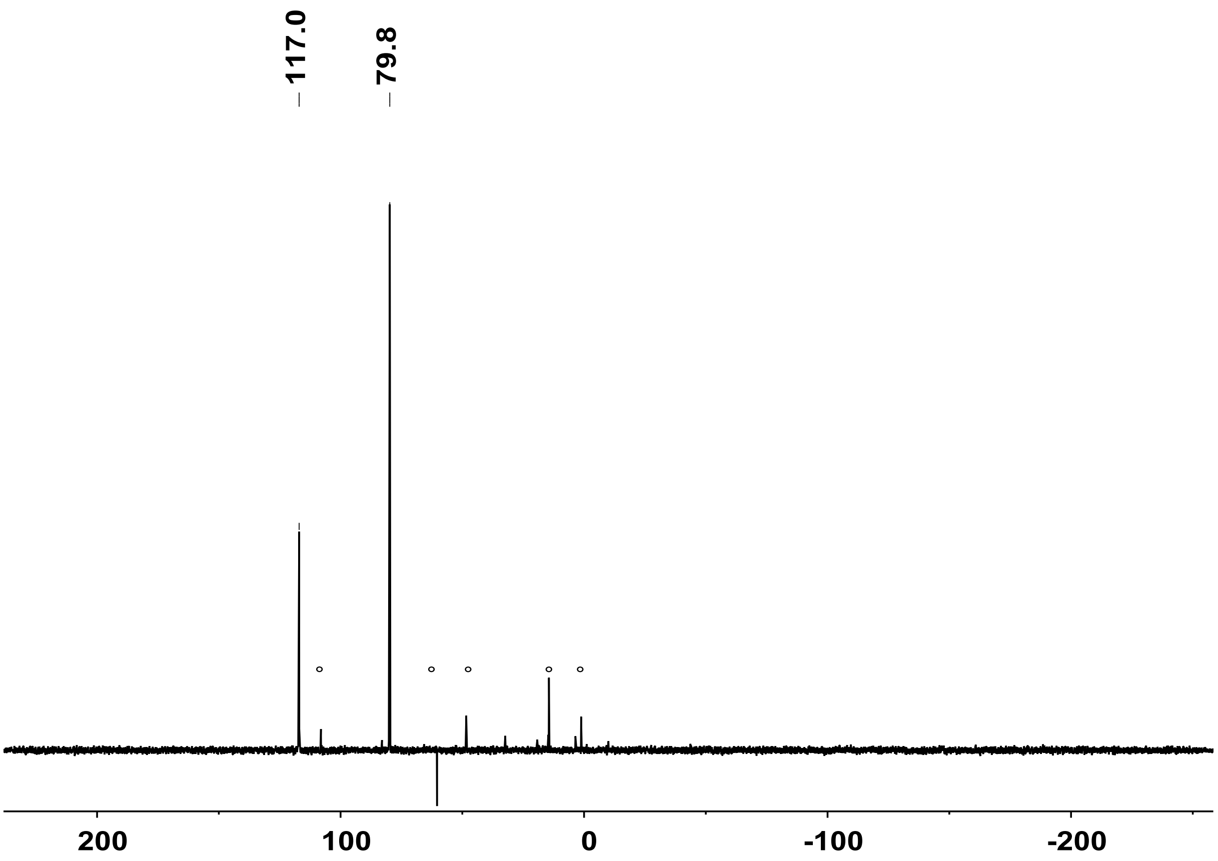

Figure S22 – $^{29}Si\{^1H\}$ INEPT NMR spectrum (99.31 MHz, 305.0 K, C_6D_6) of chloronium borate **3b**[$B(C_6F_5)_4$] and siliconium borate **4b**[$B(C_6F_5)_4$] (°impurities possibly due to side reactions).

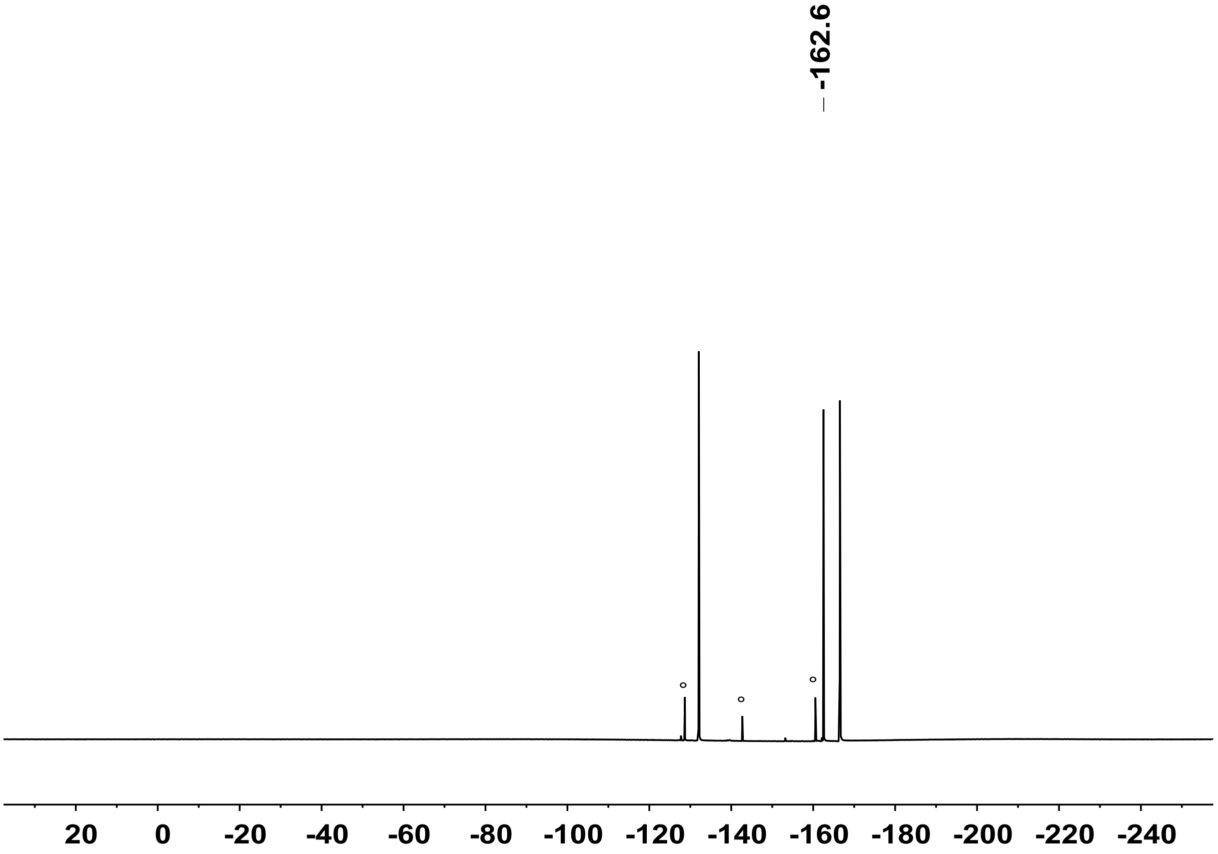

Figure S23 – $^{19}F\{^1H\}$ NMR spectrum (470.30 MHz, 305.0 K, C_6D_6) of chloronium borate **3b**[$B(C_6F_5)_4$] and siliconium borate **4b**[$B(C_6F_5)_4$] (° $B(C_6F_5)_3$).

A solution of 5-chloro-6-dimethylsilylacenaphthene **1b** (0.9 equiv, 142 µmol, 35 mg) in chlorobenzene-d_5 was cooled to -40 °C and added to a solution of trityl borate $[Ph_3C][B(C_6F_5)_4$ (1.0 equiv, 163 µmol, 150 mg) in chlorobenzene-d_5 at -40 °C in small portions. The reaction mixture was stirred for 30 min at the same temperature before it was allowed to warm to room temperature and analyzed by NMR spectroscopy, which indicated the formation of chloronium borate **3b**$[B(C_6F_5)_4]$ and siliconium borate **4b**$[B(C_6F_5)_4]$ in the ratio of 81:19 (according to ^1H NMR spectroscopy) and incomplete conversion of the trityl cation. In this reaction also $B(C_6F_5)_3$ was detected.

^{1}H NMR (499.87 MHz, 305.1 K, C_6D_5Cl): δ = 0.88 (s, $Si(CH_3)_2$, **3b**), 1.40 (s, $SiCH_3$, **4b**), 2.99-3.10 (m, CH_2, **3b** and **4b**), 6.95-7.54 (m, H-3, H-4, H-8, **3b**, H-3, H-3´, H-4, H-4´, H-8, H-8´, **4b**; overlapping with triphenylmethane and trityl cation), 7.54 (d, $^3J_{H,H}$ = 7.1 Hz, H-7, **3b**), 8.05 (d, $^3J_{H,H}$ = 7.3 Hz, H-7, H-7´, **4b**). **^{29}Si{^{1}H} NMR** (99.31 MHz, 305.0 K, C_6D_5Cl): δ = 79.6 (**4b**), 116.3-116.6 (brs, **3b**). **^{19}F{^{1}H} NMR** (470.30 MHz, 305.0 K, C_6D_5Cl): δ = -166.7-(-166.4) (m, 8 F, *m*-F, $[B(C_6F_5)_4]^-$), -162.6 (t, $^3J_{F,F}$ = 21.0 Hz, 4 F, *p*-F, $[B(C_6F_5)_4]^-$), -132.3-(-132.0) (m, 8 F, *o*-F, $[B(C_6F_5)_4]^-$).

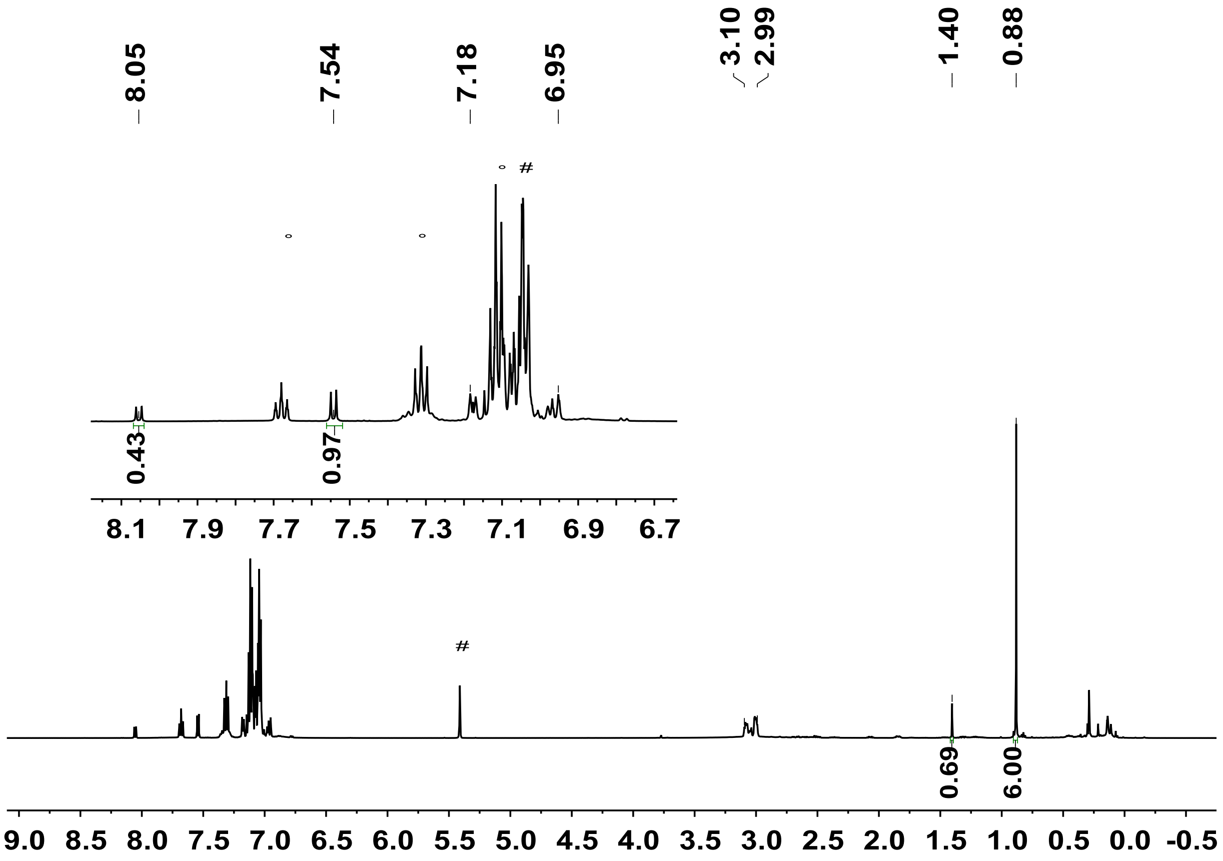

Figure S24 – ^{1}H NMR spectrum (499.87 MHz, 305.1 K, C_6D_5Cl) of chloronium borate **3b**$[B(C_6F_5)_4]$ and siliconium borate **4b**$[B(C_6F_5)_4]$ ($^{\#}$ triphenylmethane, °trityl cation).

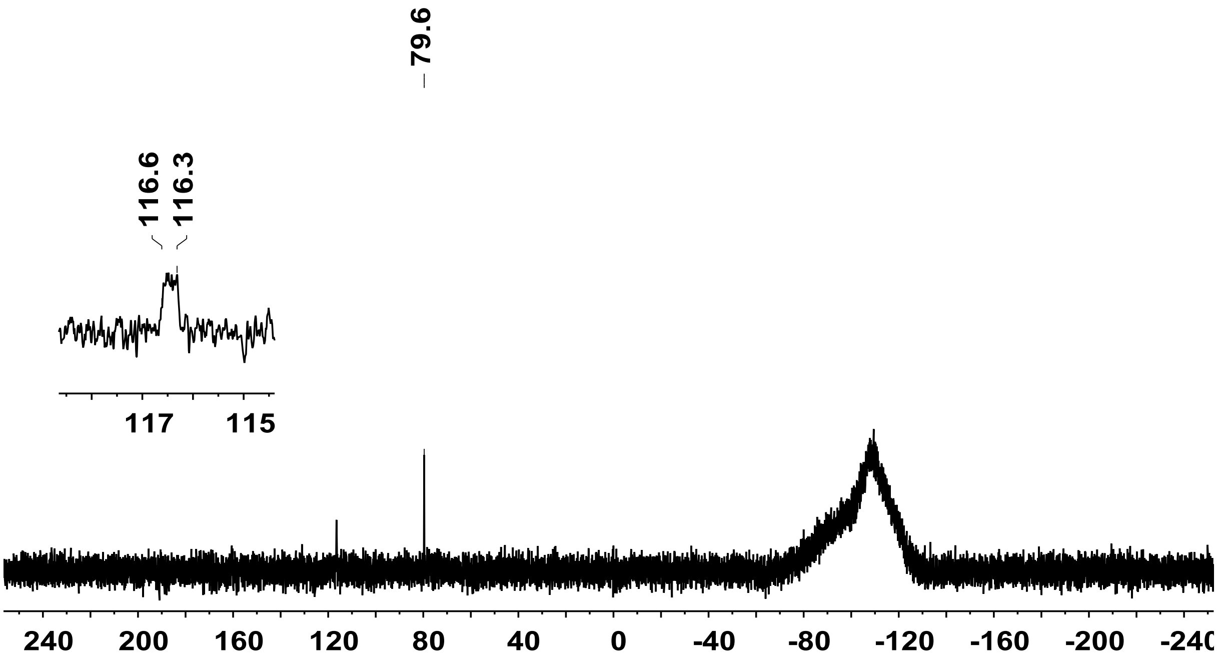

Figure S25 – ^{29}Si{^{1}H} NMR spectrum (99.31 MHz, 305.0K, C_6D_5Cl) of chloronium borate **3b**[B(C_6F_5)$_4$] and siliconium borate **4b**[B(C_6F_5)$_4$].

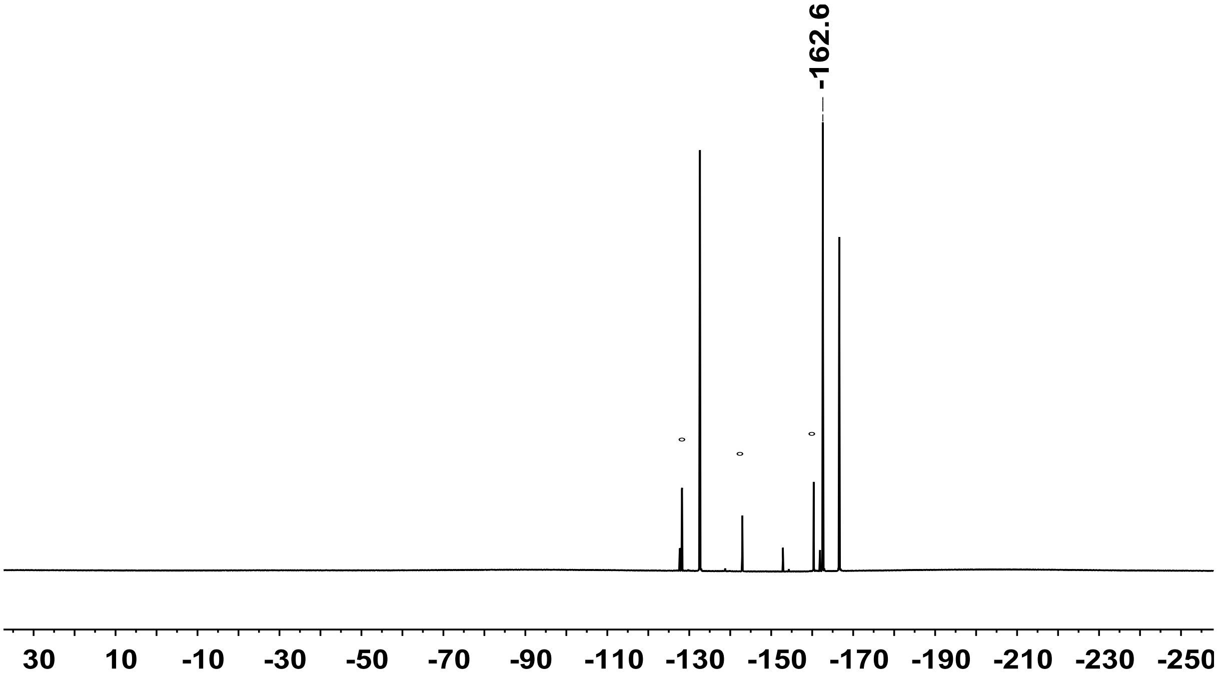

Figure S26 – ^{19}F{^{1}H} NMR spectrum (470.30 MHz, 305.0 K, C_6D_5Cl) of chloronium borate **3b**[B(C_6F_5)$_4$] and siliconium borate **4b**[B(C_6F_5)$_4$] (° B(C_6F_5)$_3$).

*bromonium ion **3c** and siliconium ion **4c***

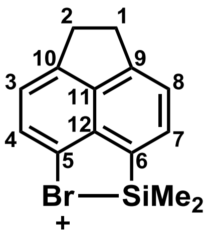

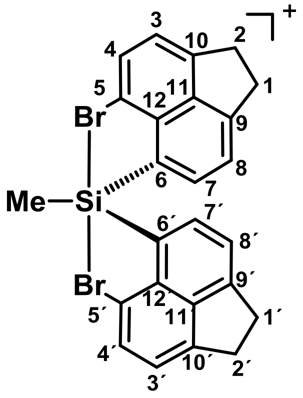

3c $[B(C_6F_5)_4]^-$ **4c** $[B(C_6F_5)_4]^-$

A solution of trityl borate $[Ph_3C][B(C_6F_5)_4]$ (0.8 equiv, 401 µmol, 370 mg) in benzene was added to a solution of 5-bromo-6-dimethylsilylacenaphthene **1c** (1.0 equiv, 501 µmol, 146 mg) in benzene at r.t. and stirred vigorously for 30 min. Then, the upper, nonpolar phase was removed and the polar phase was washed twice with benzene. After removing the solvent under reduced pressure, the residue was dissolved in benzene-d_6 and analyzed by NMR spectroscopy, which indicated the formation of bromonium borate **3c**$[B(C_6F_5)_4]$ and siliconium borate **4c**$[B(C_6F_5)_4]$ in the ratio of 65:35 and full conversion of the trityl cation.

^{1}H NMR (499.87 MHz, 305.2 K, C_6D_6): δ = 0.62 (s, $Si(CH_3)_2$, **3c**), 1.36 (s, $SiCH_3$, **4c**), 2.90-3.05 (m, CH_2, **3c** and **4c**), 6.90 (d, $^3J_{H,H}$ = 7.8 Hz, H-3, **3c**), 6.93-6.99 (m, H-4, **3c** and H-3, H-3´, **4c**), 7.09-7.15 (m, H-4, **3c** and H-8, H-8´, **4c**), 7.25 (d, $^3J_{H,H}$ = 7.2 Hz, H-8, **3c**), 7.31 (d, $^3J_{H,H}$ = 7.7 Hz, H-4, H-4´, **4c**), 7.87 (d, $^3J_{H,H}$ = 7.2 Hz, H-7, H-7´, **4c**). **^{29}Si{^{1}H} NMR** (99.31 MHz, 305.0 K, C_6D_6): δ = 82.7 (**4c**), 108.0 (**3c**). **^{29}Si{^{1}H} INEPT NMR** (99.31 MHz, 305.0 K, C_6D_6): δ = 82.7 ($^1J_{Si,C}$ = 81.6 Hz, $^1J_{Si,C}$ = 62.2 Hz, **4c**), 108.0 ($^1J_{Si,C}$ = 74.3 Hz, $^1J_{Si,C}$ = 53.9 Hz, **3c**).

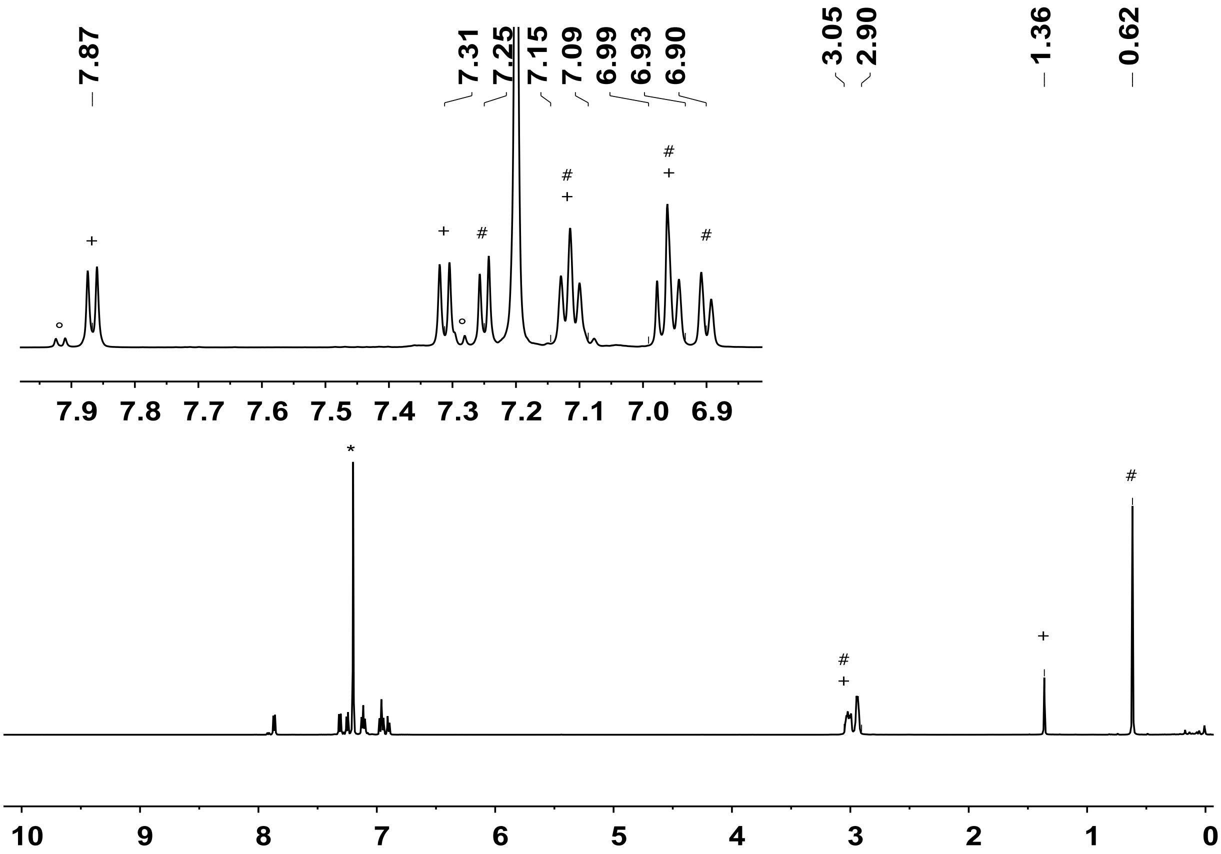

Figure S27 – ^{1}H NMR spectrum (499.87 MHz, 305.2 K, C_6D_6) of bromonium borate **3c**[$B(C_6F_5)_4$] and siliconium borate **4c**[$B(C_6F_5)_4$] (* C_6D_5H, # bromonium ion **3c**, + siliconium ion **4c**, ° impurities possibly due to side reactions).

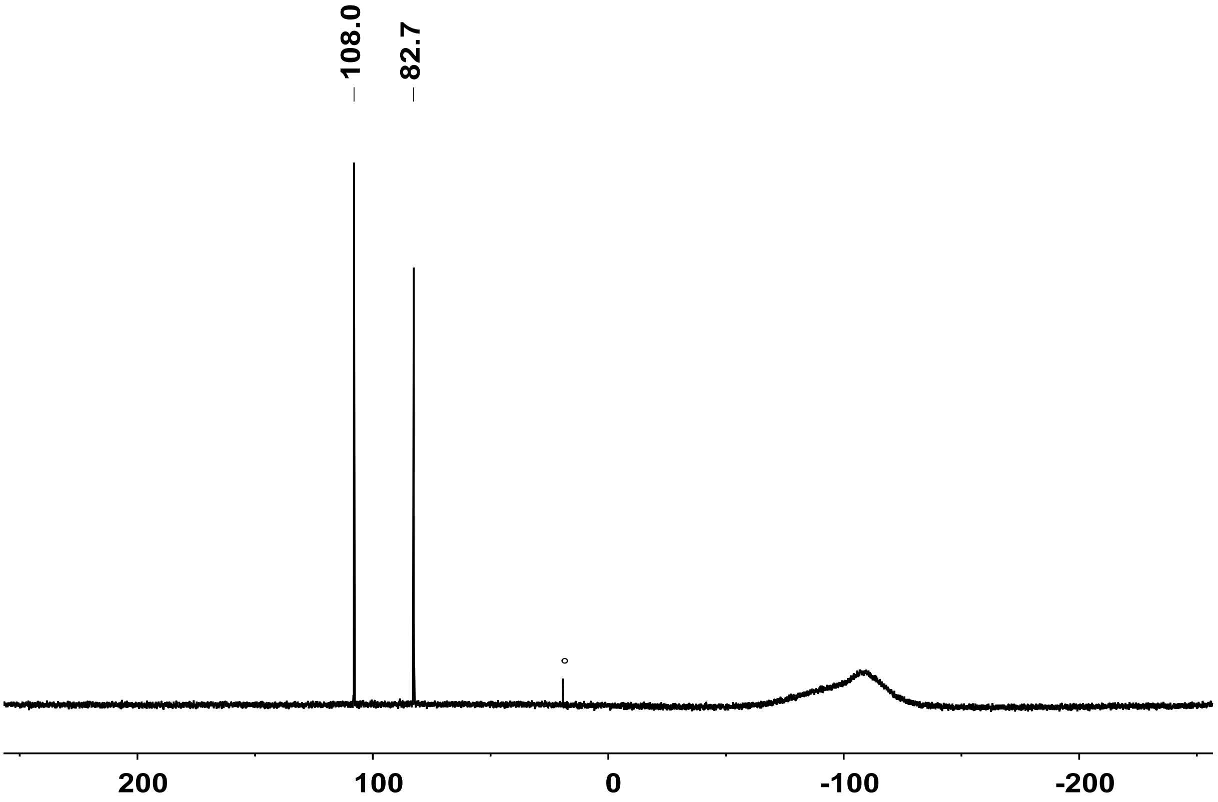

Figure S28 – $^{29}Si\{^{1}H\}$ NMR spectrum (99.31 MHz, 305.0K, C_6D_6)of bromonium borate **3c**[$B(C_6F_5)_4$] and siliconium borate **4c**[$B(C_6F_5)_4$] (° impurities possibly due to side reactions).

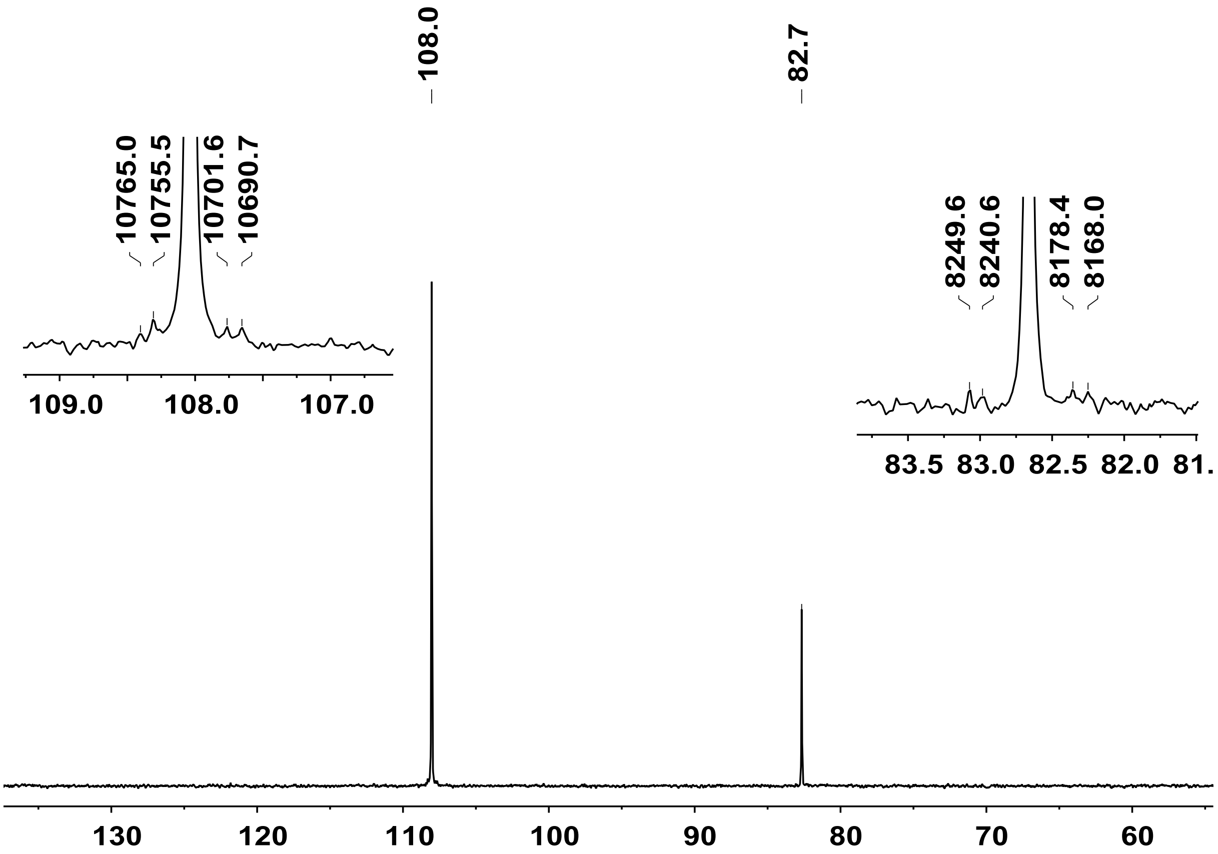

Figure S29 – $^{29}Si\{^{1}H\}$ INEPT NMR spectrum (99.31 MHz, 305.0 K, C_6D_6) of bromonium borate **3c**[B(C_6F_5)$_4$] and siliconium borate **4c**[B(C_6F_5)$_4$].

*bromonium ion **3c***

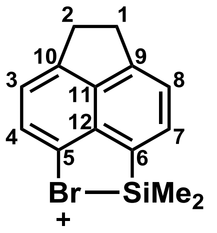

3c **$[B(C_6F_5)_4]^-$**

In toluene-d$_8$: A solution of trityl borate $[Ph_3C][B(C_6F_5)_4]$ (1.0 equiv, 434 µmol, 400 mg) in toluene-d$_8$ was cooled to -10 °C and added to a solution of 5-bromo-6-dimethylsilylacenaphthene **1c** (1.1 equiv, 477 µmol, 138 mg) in toluene-d$_8$ at -10 °C. Then, the mixture was stirred for 30 min at the same temperature, while the color changed from yellow orange to dark green. After that, the biphasic reaction mixture was allowed to warm to room temperature and the upper, nonpolar phase was removed. The polar phase was washed with 0.3 mL toluene-d$_8$ and bromonium borate **3c**$[B(C_6F_5)_4]$ was subsequently analyzed by NMR spectroscopy at T = -10 °C.

In benzene-d$_6$: One third of a solution of 5-bromo-6-dimethylsilylacenaphthene **1c** (1.02 equiv, 409 µmol, 119 mg) in 5 mL benzene-d$_6$ was added to a solution of trityl borate $[Ph_3C][B(C_6F_5)_4]$ (1.0 equiv, 401 µmol, 370 mg) in benzene-d$_6$ in small portions. During the addition, the reaction mixture was cooled with cold water. Since benzene-d$_6$ was freezing, the mixture was brought to 12 °C and the remaining silane solution was further added in small portions and stirred vigorously for 30 min at room temperature while warming to room temperature. The color of the reaction mixture changed from yellow orange to brown. Then, the upper, nonpolar phase was removed and the polar phase, containing the bromonium borate **3c**$[B(C_6F_5)_4]$, was analyzed by NMR spectroscopy.

^{1}H NMR (499.87 MHz, 263.0 K, C_7D_8): δ = 0.57 (s, 6H, $Si(CH_3)_2$), 2.86-3.00 (m, 4H, CH_2, H-1, H-2), 6.84 (d, 1H, $^3J_{H,H}$ = 7.9 Hz, H-3), 6.87 (d, 1H, $^3J_{H,H}$ = 7.9 Hz, H-4), 7.04-7.09 (m, 1H, H-8), 7.19 (d, 1H, $^3J_{H,H}$ = 7.1 Hz, H-7). **^{1}H NMR** (499.87 MHz, 305.1 K, C_6D_6): δ = 0.62 (s, 6H, $Si(CH_3)_2$), 2.93-3.05 (m, 4H, CH_2, H-1, H-2), 6.89-6.92 (m, 1H, H-3), 6.97 (d, 1H, $^3J_{H,H}$ = 7.9 Hz, H-4), 7.11-7.13 (m, 1H, H-8, overlapping with triphenylmethane), 7.25 (d, 1H, $^3J_{H,H}$ = 7.1 Hz, H-7). **^{13}C{^{1}H} NMR** (125.71 MHz, 263.0 K, C_7D_8): δ = 2.8 ($Si(CH_3)_2$, $^1J_{C,Si}$ = 54.3 Hz), 30.2 (CH_2, C-2), 31.4 (CH_2, C-1), 121.8 (C, $^1J_{C,Si}$ = 74.1 Hz, C-6), 122.6 (CH, C-3 or C-8), 122.7 (CH, C-3 or C-8), 122.9 (C, C-5), 124.8 (brs, C, $[B(C_6F_5)_4]^-$), 126.5 (CH, C-4), 133.2 (C, C-12),

136.2 (CH, C-7), 137.0 (dm, $^1J_{C,F}$ = 240.1 Hz, CF, $[B(C_6F_5)_4]^-$), 138.9 (dm, $^1J_{C,F}$ = 239.2 Hz, CF, $[B(C_6F_5)_4]^-$),141.0 (C, C-11), 149.1 (dm, $^1J_{C,F}$ = 240.7 Hz, CF, $[B(C_6F_5)_4]^-$), 150.8 (C, C-10), 153.1 (C, C-9). **^{13}C{^{1}H} NMR** (125.77 MHz, 299.3 K, C_6D_6): δ = 2.9 ($Si(CH_3)_2$, $^1J_{C,Si}$ = 54.0 Hz), 30.1 (CH_2, C-2), 31.3 (CH_2, C-1), 121.8 (C, C-6), 122.59 (CH, C-3 or C-8), 122.63 (CH, C-3 or C-8), 122.8 (C, C-5), 124.9 (brs, C, $[B(C_6F_5)_4]^-$), 126.5 (CH, C-4), 133.1 (C, C-12), 136.1 (CH, C-7), 137.0 (dm, $^1J_{C,F}$ = 240.5 Hz, CF, $[B(C_6F_5)_4]^-$), 138.0 (dm, $^1J_{C,F}$ = 239.5 Hz, CF, $[B(C_6F_5)_4]^-$),141.0 (C, C-11), 149.1 (dm, $^1J_{C,F}$ = 241.5 Hz, CF, $[B(C_6F_5)_4]^-$), 150.7 (C, C-10), 153.0 (C, C-9). **^{29}Si{^{1}H} NMR** (99.31 MHz, 263.0 K, C_7D_8): δ = 107.5 (s, $^1J_{Si,C}$ = 53.5 Hz, $^1J_{Si,C}$ = 73.8 Hz). **^{29}Si{^{1}H} NMR** (99.36 MHz, 299.1 K, C_6D_6): δ = 107.9 (s, $^1J_{Si,C}$ = 53.9 Hz, $^1J_{C,Si}$ = 75.5 Hz). **^{29}Si{^{1}H} INEPT NMR** (99.31 MHz, 263.0 K, C_7D_8): δ = 107.5 (s, $^1J_{Si,C}$ = 54.1 Hz, $^1J_{Si,C}$ = 75.0 Hz). **^{29}Si{^{1}H} INEPT NMR** (99.36 MHz, 299.0 K, C_6D_6): δ = 107.9. **^{19}F{^{1}H} NMR** (470.30 MHz, 263.1 K, C_7D_8): δ = -167.6-(-167.0) (m, 8F, CF), -163.6-(-163.2) (m, 4F, CF), -133.5-(-132.8) (m, 8F, CF). **^{19}F{^{1}H} NMR** (470.30 MHz, 305.0 K, C_6D_6): δ = -167.1-(-165.9) (m, 8 F, *m*-F, $[B(C_6F_5)_4]^-$), -162.5 (t, $^3J_{F,F}$ = 21.0 Hz, 4 F, *p*-F, $[B(C_6F_5)_4]^-$), -133.2-(-131.2) (m, 8 F, *o*-F, $[B(C_6F_5)_4]^-$). **^{11}B{^{1}H} NMR** (160.38 MHz, 263.2 K, C_7D_8): δ = -16.7. **^{11}B{^{1}H} NMR** (160.38 MHz, 305.0 K, C_6D_6): δ = -16.0.

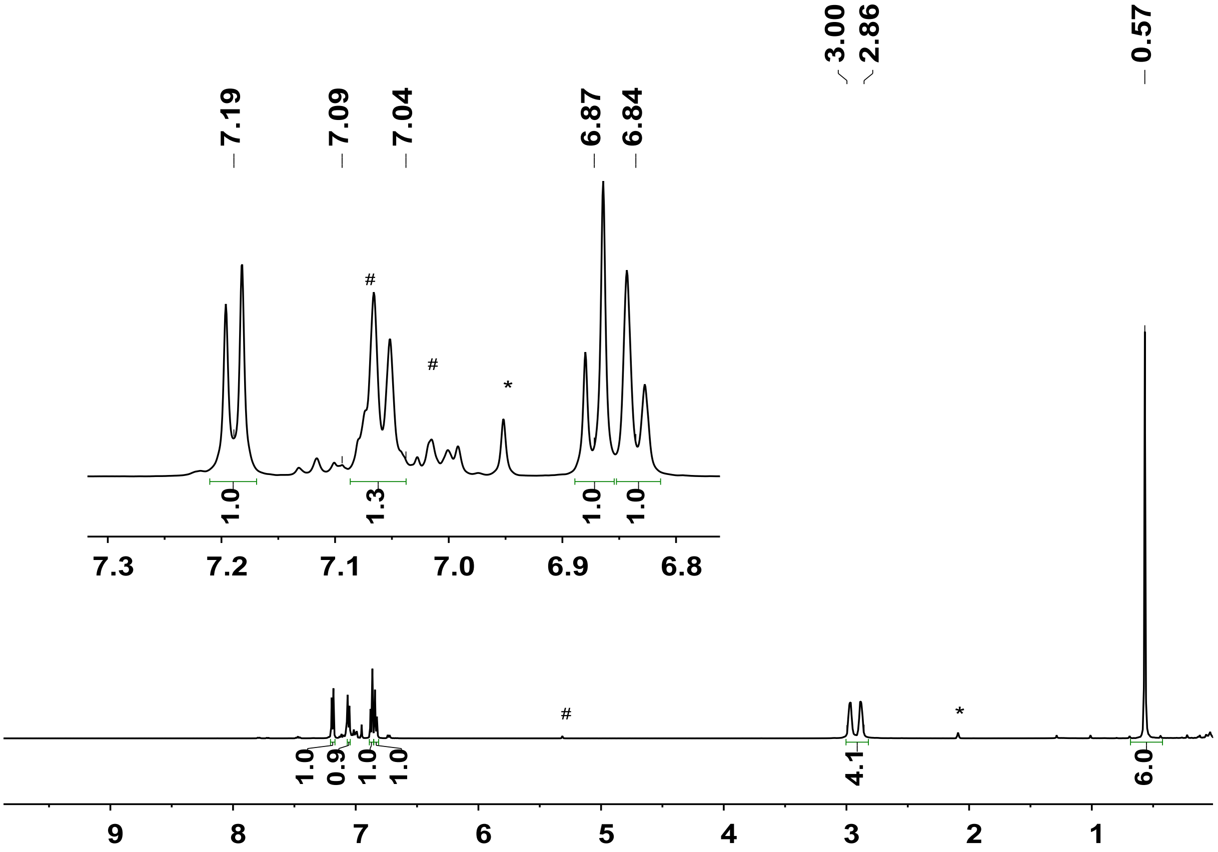

Figure S30 – ^{1}H NMR spectrum (499.87 MHz, 263.0 K, C_7D_8) of bromonium borate **3c**$[B(C_6F_5)_4]$ (* $C_6D_5CD_2H$, # triphenylmethane).

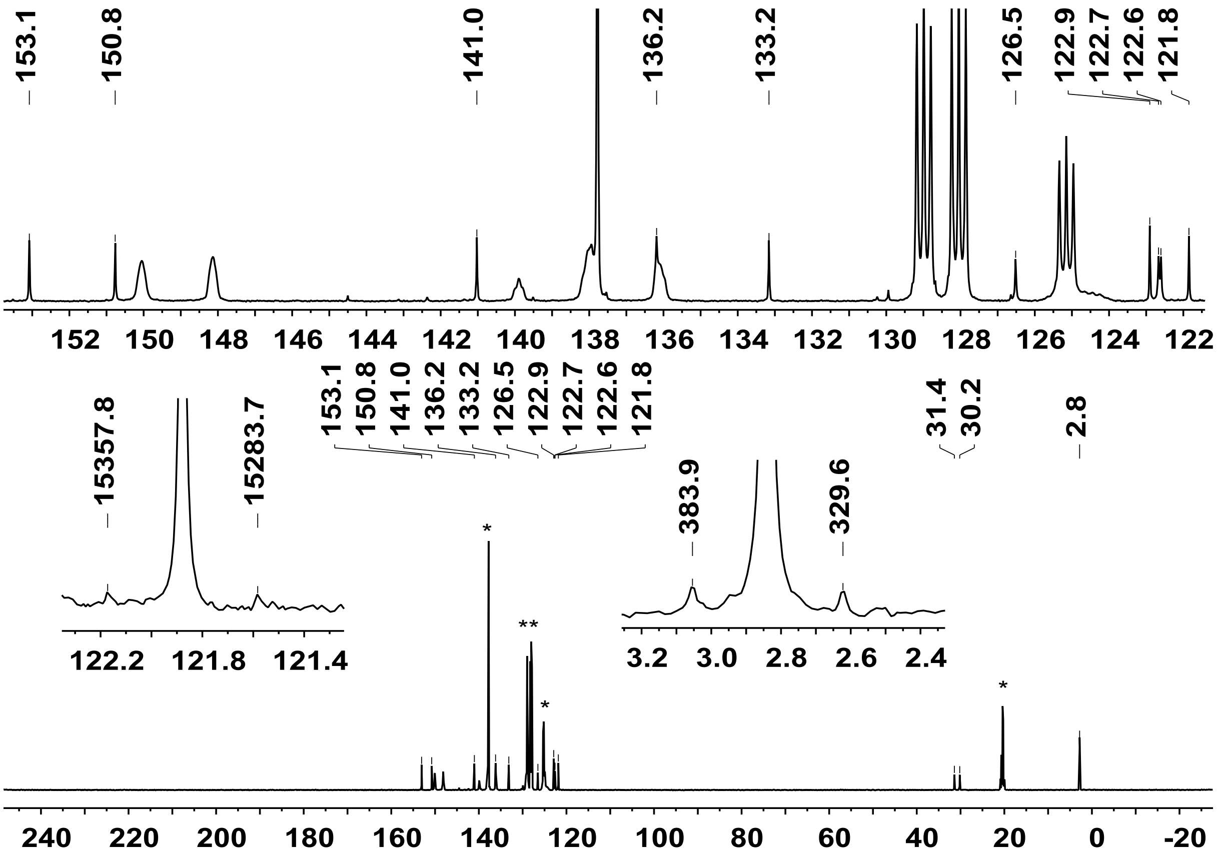

Figure S31 – $^{13}C\{^1H\}$ NMR spectrum (125.71 MHz, 263.0 K, C_7D_8) of bromonium borate **3c**[B(C_6F_5)$_4$] (* C_7D_8).

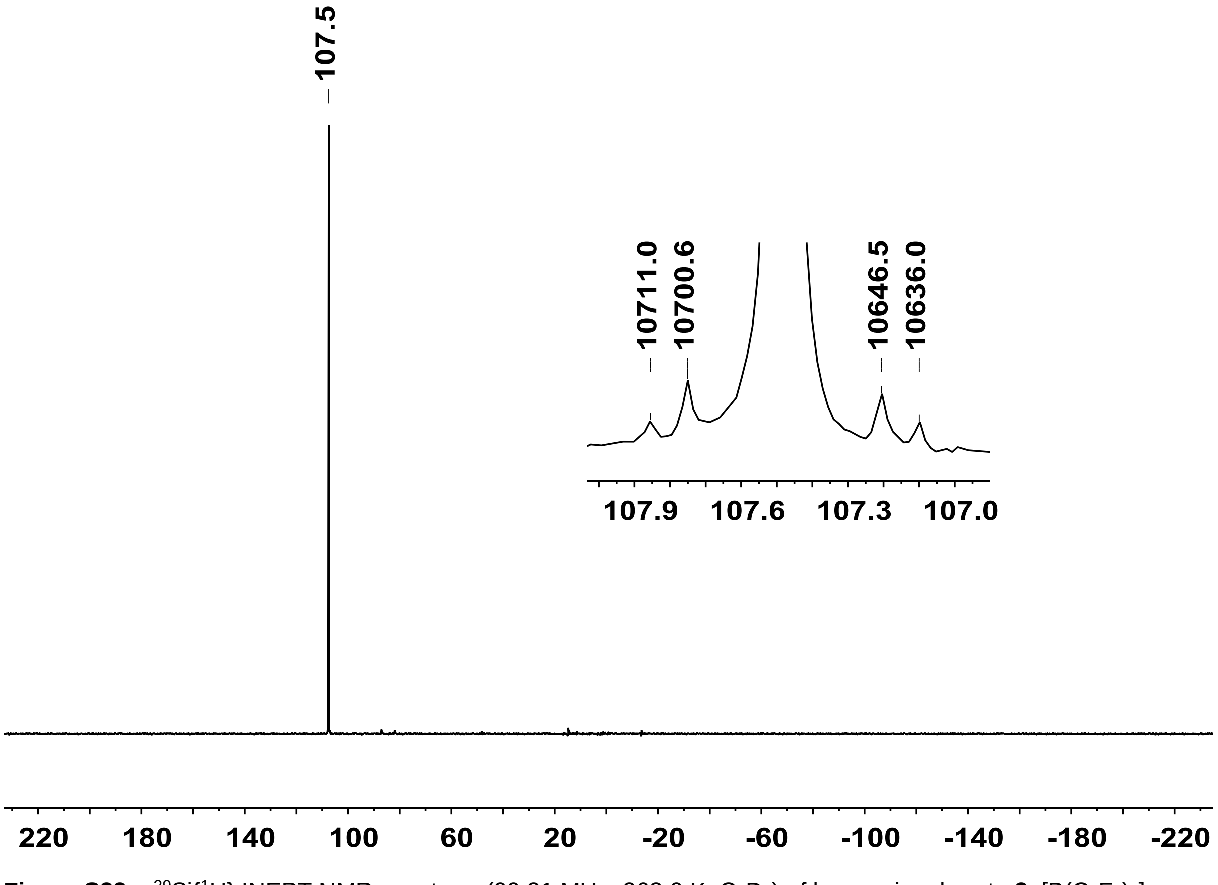

Figure S32 – $^{29}Si\{^1H\}$ INEPT NMR spectrum (99.31 MHz, 263.0 K, C_7D_8) of bromonium borate **3c**[B(C_6F_5)$_4$].

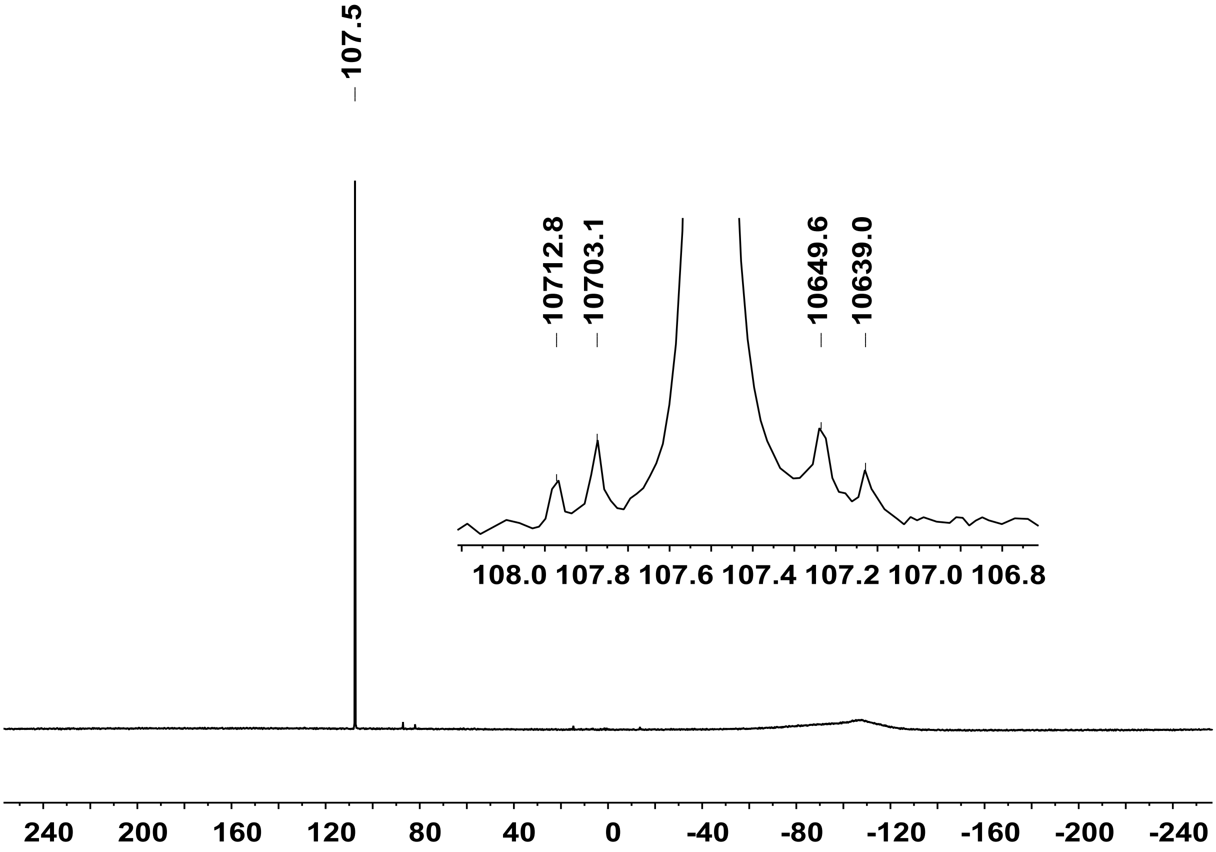

Figure S33 – ^{29}Si{^{1}H} NMR spectrum (99.31 MHz, 263.0K, C_7D_8) of bromonium borate **3c**[B(C_6F_5)$_4$].

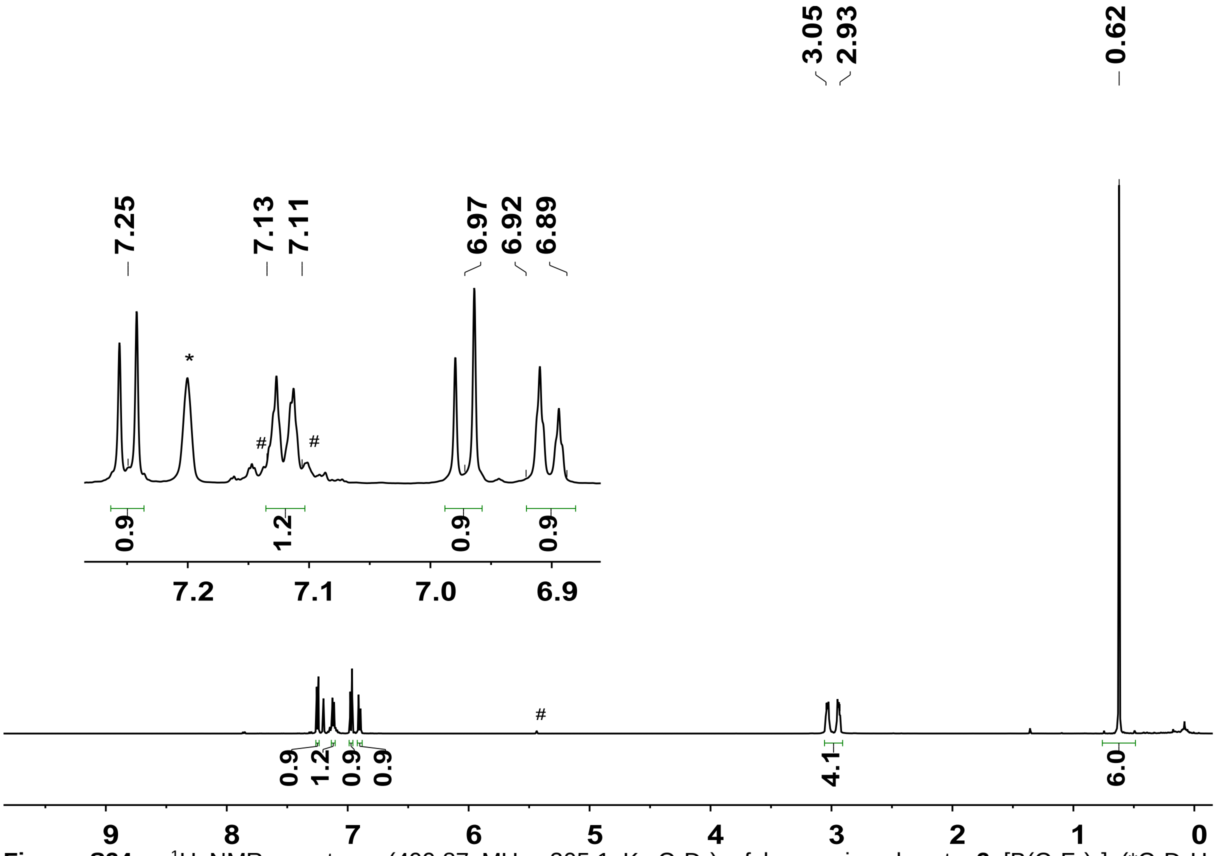

Figure S34 – ^{1}H NMR spectrum (499.87 MHz, 305.1 K, C_6D_6) of bromonium borate **3c**[B(C_6F_5)$_4$] (*C_6D_5H, # triphenylmethane).

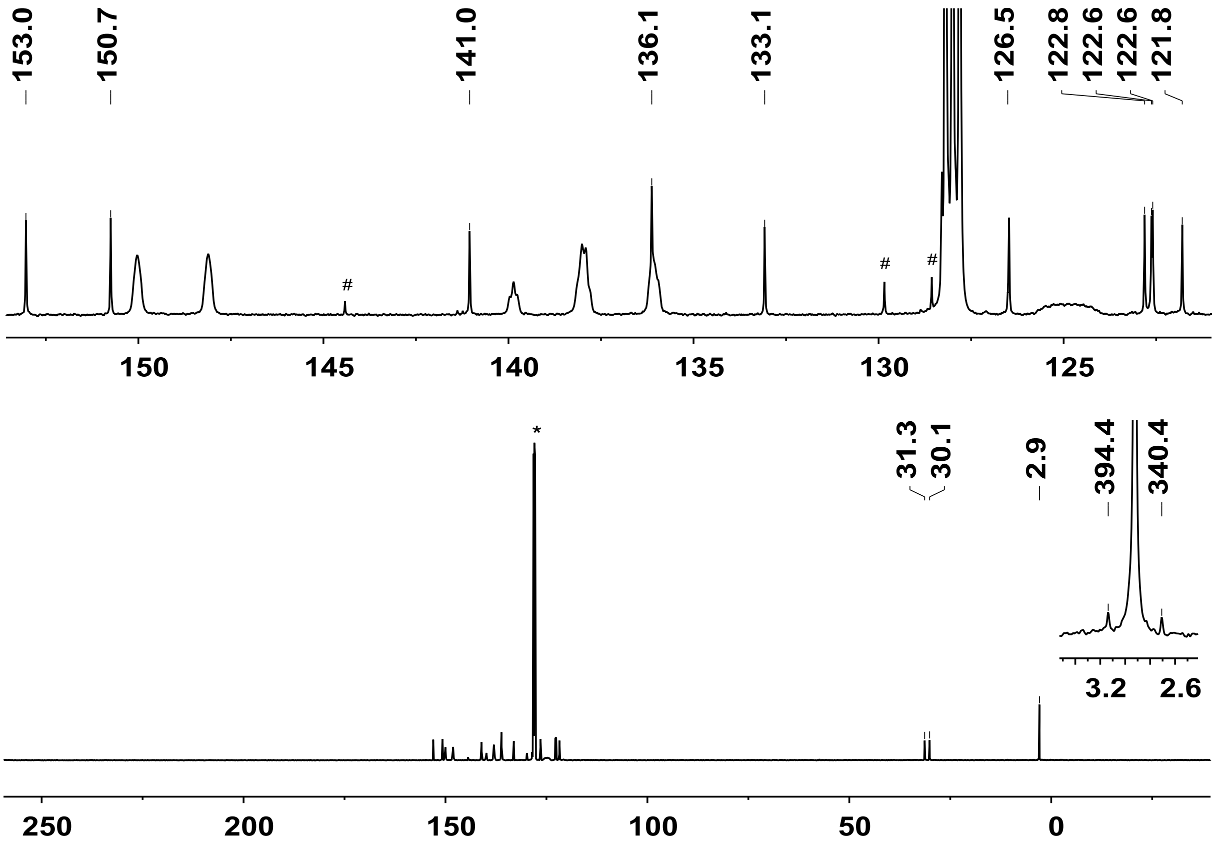

Figure S35 – $^{13}C\{^{1}H\}$ NMR spectrum (125.77 MHz, 299.3 K, C_6D_6) of bromonium borate **3c**[$B(C_6F_5)_4$] (* C_6D_6).

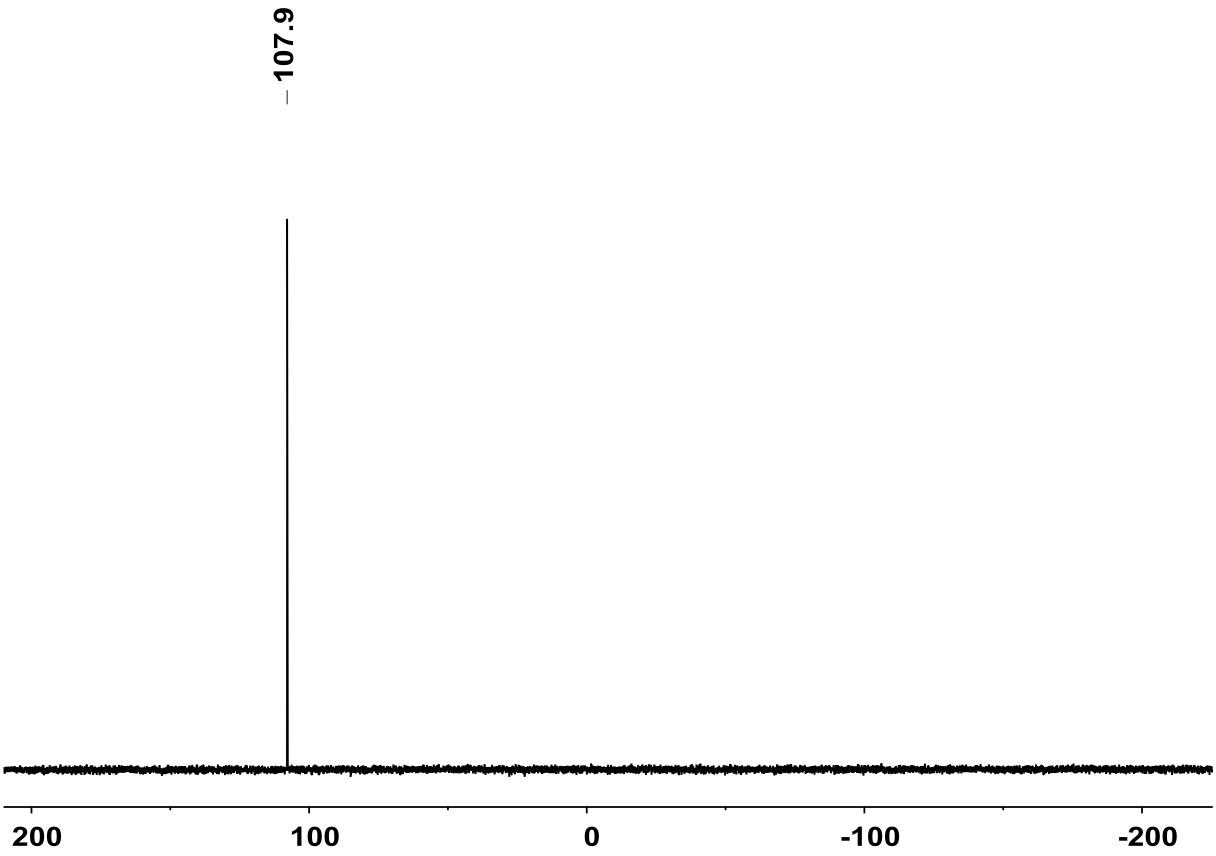

Figure S36 – $^{29}Si\{^{1}H\}$ INEPT NMR spectrum (99.36 MHz, 299.0 K, C_6D_6) of bromonium borate **3c**[$B(C_6F_5)_4$].

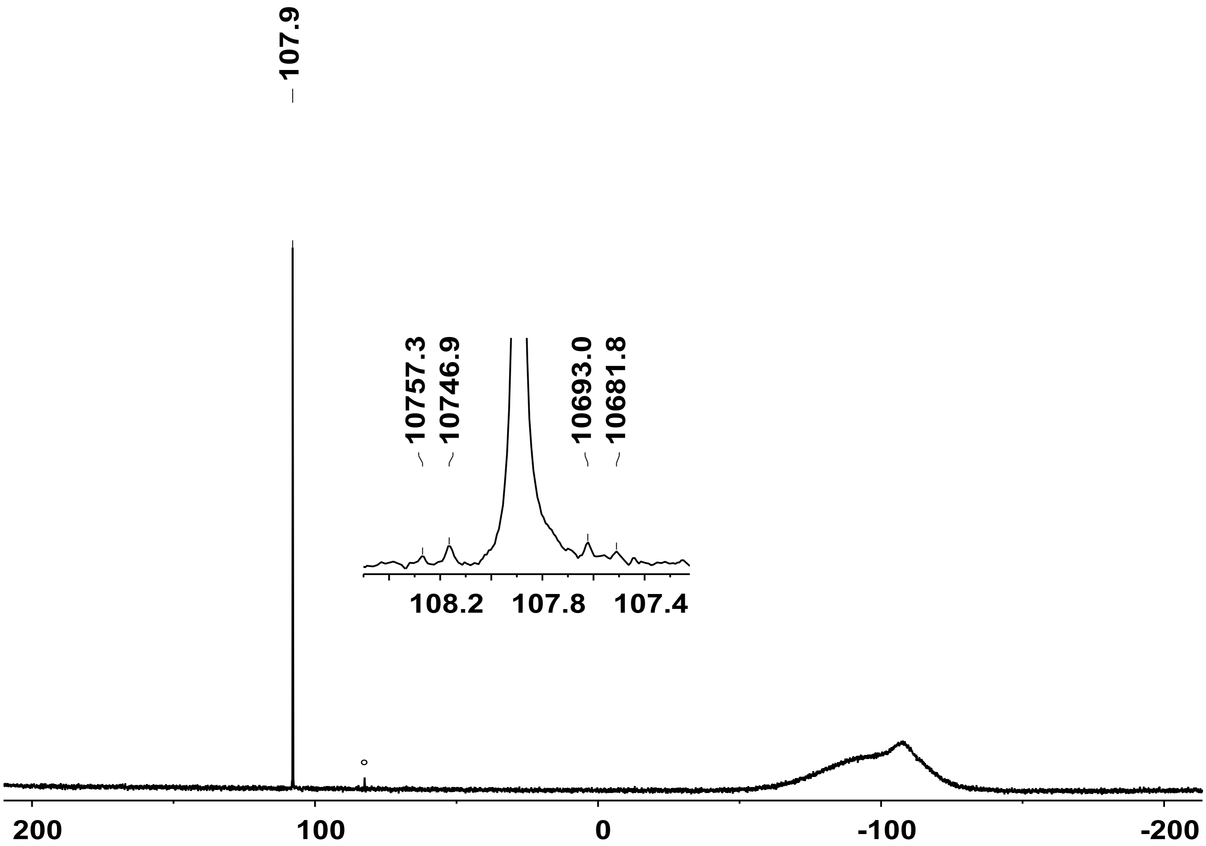

Figure S37 – $^{29}Si\{^{1}H\}$ NMR spectrum (99.36 MHz, 299.1 K, C_6D_6) of bromonium borate **3c**[B(C_6F_5)$_4$] (°siliconium borate **4c**[B(C_6F_5)$_4$]).

siliconium ion ***4c***

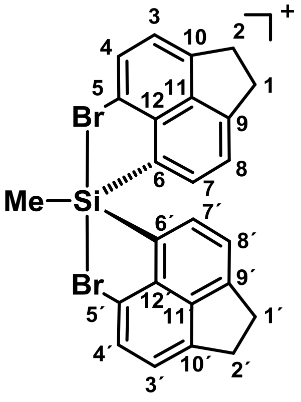

10c $[B(C_6F_5)_4]^-$

A solution of 5-bromo-6-dimethylsilylacenaphthene **1c** (3.0 equiv, 1.30 mmol, 379 mg) in benzene-d_6 was added to a solution of trityl borate $[Ph_3C][B(C_6F_5)_4]$ (1.0 equiv, 434 µmol, 400 mg) in benzene-d_6 at r.t. and stirred vigorously for 30 min. The color of the reaction mixture changed from yellow orange to brown. Then, the phases of the biphasic reaction mixture were analyzed separately by NMR spectroscopy. The upper, nonpolar phase did not show any evidence for 5-bromo-6-dimethylsilylacenaphthene **1c**. The polar phase showed almost complete conversion to siliconium ion **4c** and was first washed with benzene and then with a solvent mixture containing benzene and pentane in a ratio of 1:1. After removing the solvents under reduced pressure, the residue was dissolved in benzene-d_6 and analyzed by NMR spectroscopy.

^{1}H NMR (499.87 MHz, 305.1 K, C_6D_6): δ = 1.36 (s, 3H, CH_3, 2.89-3.04 (m, 8H, CH_2, H-1, H-2, H-1´,H-2´), 6.94 (d, 2H, $^3J_{H,H}$ = 7.7 Hz, H-3, H-3´), 7.11 (d, 2H, $^3J_{H,H}$ = 7.2 Hz, H-8, H-8´), 7.31 (d, 2H, $^3J_{H,H}$ = 7.7 Hz, H-4, H-4´), 7.87 (d, 2H, $^3J_{H,H}$ = 7.2 Hz, H-7, H-7´). **^{13}C{^{1}H} NMR** (125.71 MHz, 305.0 K, C_6D_6): δ = 13.0 (CH_3), 30.0 (CH_2, C-2, C-2´), 31.0 (CH_2, C-1, C-1´), 117.9 (C, C-5, C-5´), 120.9 (C, C-6, C-6´), 121.4 (CH, C-8, C-8´), 122.7 (CH, C-3, C-3´), 125.1 (brs, C, $[B(C_6F_5)_4]^-$), 129.9 (CH, C-4, C-4´), 134.1 (C, C-12, C-12´), 137.0 (dm, $^1J_{C,F}$ = 239.5 Hz, CF, $[B(C_6F_5)_4]^-$), 139.0 (dm, $^1J_{C,F}$ = 238.0 Hz, CF, $[B(C_6F_5)_4]^-$), 141.3 (CH, C-7, C-7´), 141.4 (C, C-11, C-11´), 149.2 (dm, $^1J_{C,F}$ = 240.9 Hz, CF, $[B(C_6F_5)_4]^-$), 149.9 (C, C-10, C-10´), 154.8 (C, C-9, C-9´). **^{29}Si{^{1}H} NMR** (99.31 MHz, 305.0 K, C_6D_6): δ = 82.6. **^{29}Si{^{1}H} INEPT NMR** (99.36 MHz, 299.0 K, C_6D_6): δ = 82.6. **^{19}F{^{1}H} NMR** (470.30 MHz, 305.0 K, C_6D_6): δ = -166.6-(-166.3) (m, 8 F, *m*-F, $[B(C_6F_5)_4]^-$), -162.5 (t, $^3J_{F,F}$ = 20.6 Hz, 4 F, *p*-F, $[B(C_6F_5)_4]^-$), -131.9-(-131.7) (m, 8 F, *o*-F, $[B(C_6F_5)_4]^-$). **^{11}B{^{1}H} NMR** (160.38 MHz, 305.0 K, C_6D_6): δ = -15.9.

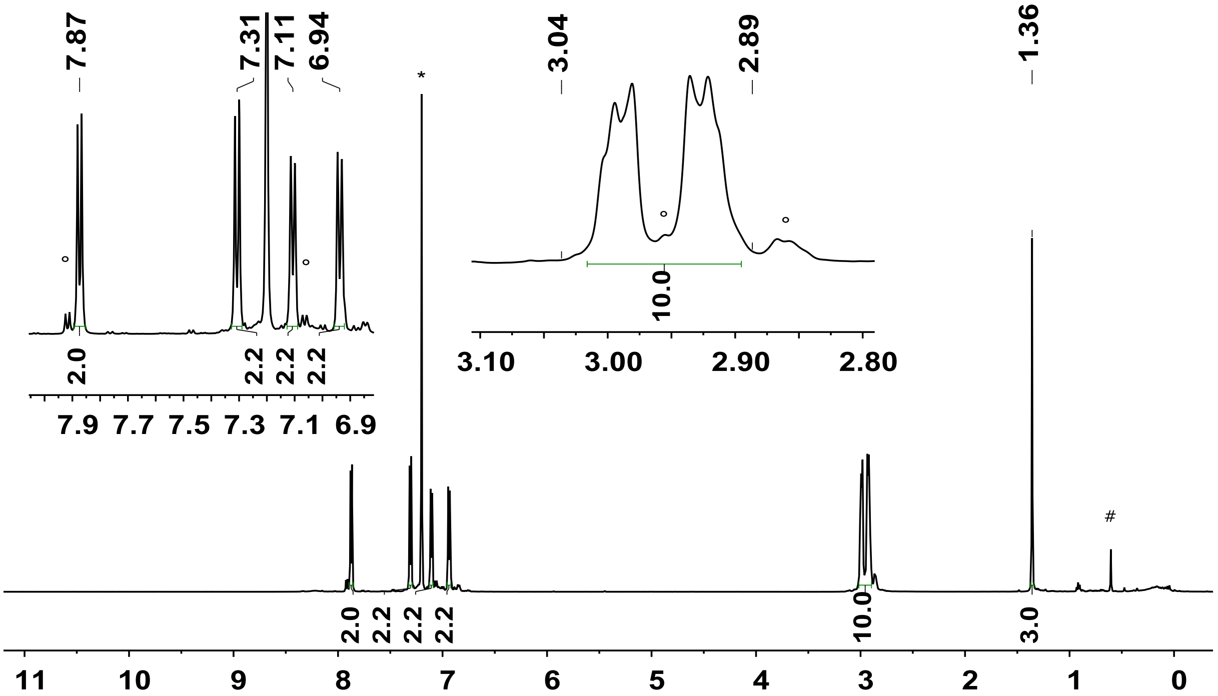

Figure S38 – ^{1}H NMR spectrum (499.87 MHz, 305.1 K, C_6D_6) of siliconium borate **4c**[B(C_6F_5)$_4$] (* C_6D_5H, # bromonium ion **3c**, °impurities possibly due to side reactions).

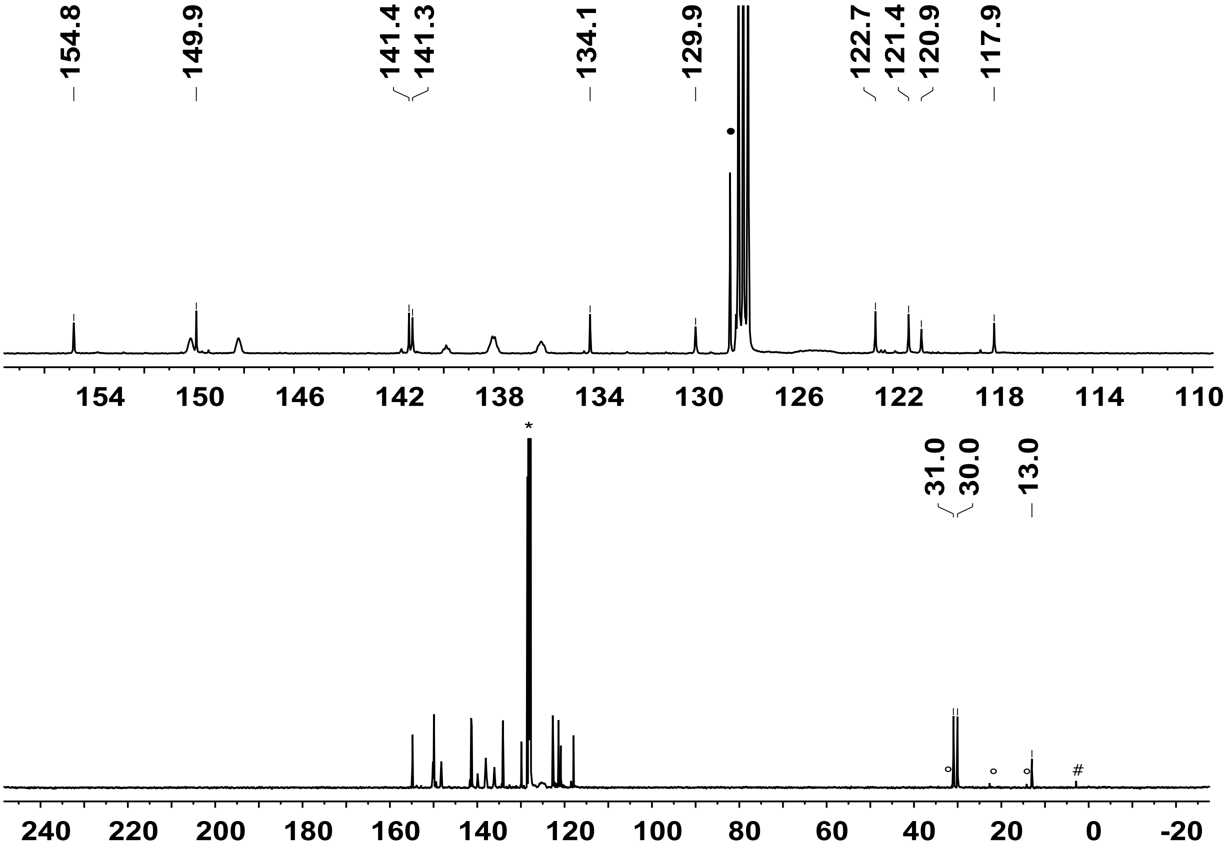

Figure S39 – ^{13}C{^{1}H} NMR spectrum (125.71 MHz, 305.0 K, C_6D_6) of siliconium borate **4c**[B(C_6F_5)$_4$] (* C_6D_6, # bromonium ion **3c**, ° *n*-pentane, • residual C_6H_6).

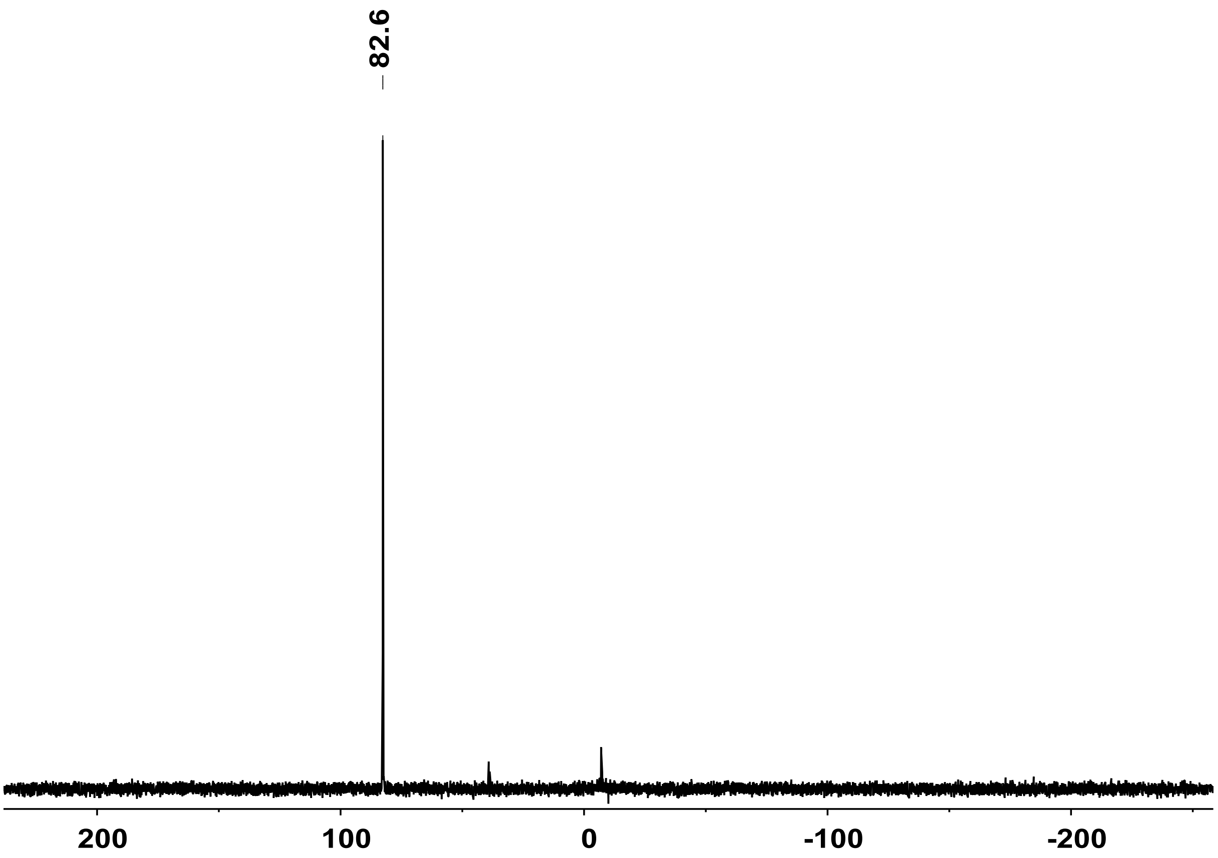

Figure S40 – $^{29}Si\{^{1}H\}$ INEPT NMR spectrum (99.31 MHz, 305.0 K, C_6D_6) of siliconium borate **4c**[$B(C_6F_5)_4$].

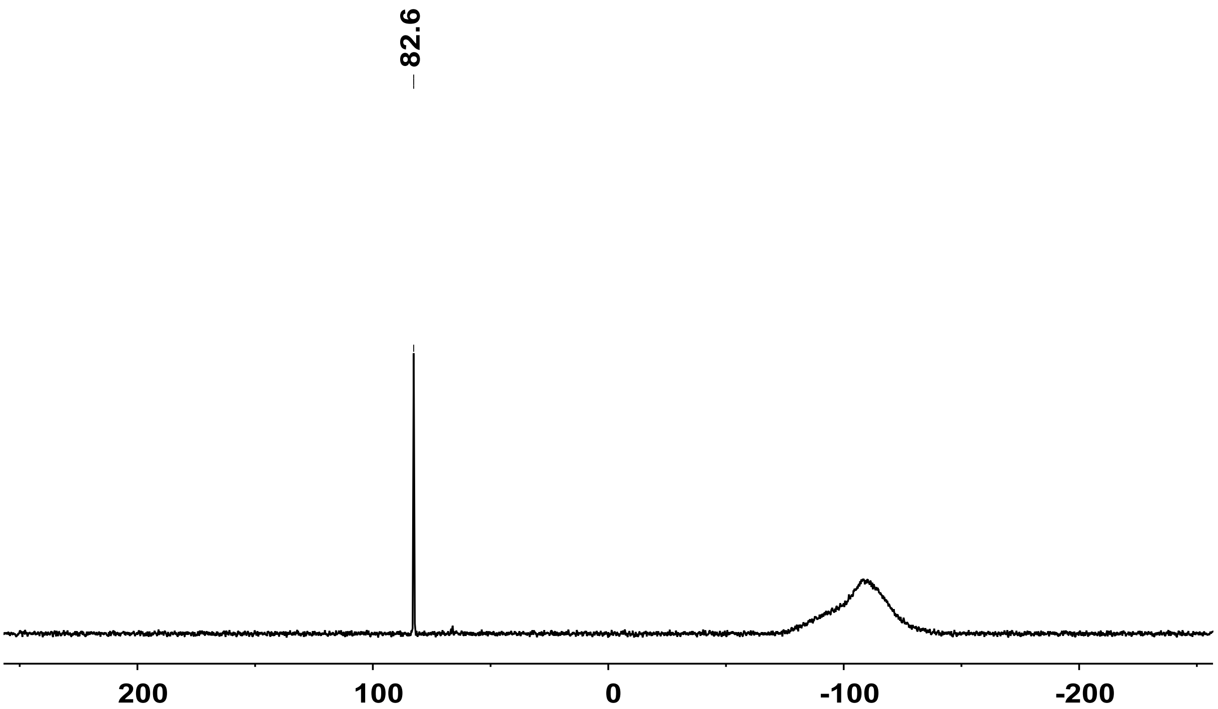

Figure S41 – $^{29}Si\{^{1}H\}$ NMR spectrum (99.31 MHz, 305.0K, C_6D_6) of siliconium borate **4c**[$B(C_6F_5)_4$].

Using 2.15 equiv of 5-bromo-6-dimethylsilylacenaphthene 1c did not show full conversion to the siliconium borate: A solution of trityl borate $[Ph_3C][B(C_6F_5)_4]$ (1 equiv, 163 µmol, 150 mg) in chlorobenzene-d_5 was added to a solution of 5-bromo-6-dimethylsilylacenaphthene **1c** (2.15 equiv, 350 µmol, 102 mg) in chlorobenzene-d_5 at r.t., stirred vigorously for 30 min and analyzed by NMR spectroscopy, which indicated the formation of bromonium borate **3c**$[B(C_6F_5)_4]$ and siliconium borate **4c**$[B(C_6F_5)_4]$ in the ratio of 13:87. Then, 1 mL *n*-hexane was added to the mixture to create a biphasic reaction mixture and the upper, nonpolar phase was removed. The polar phase was washed with *n*-hexane. After removing the solvent under reduced pressure, the residue was dissolved in chlorobenzene-d_5 and again analyzed by NMR spectroscopy, which gave a 5:95 ratio of bromonium borate **3c**$[B(C_6F_5)_4]$ and siliconium borate **4c**$[B(C_6F_5)_4]$, possibly caused by decomposition of the bromonium borate **3c**$[B(C_6F_5)_4]$. This sample was then analyzed by NMR spectroscopy at -40 °C.

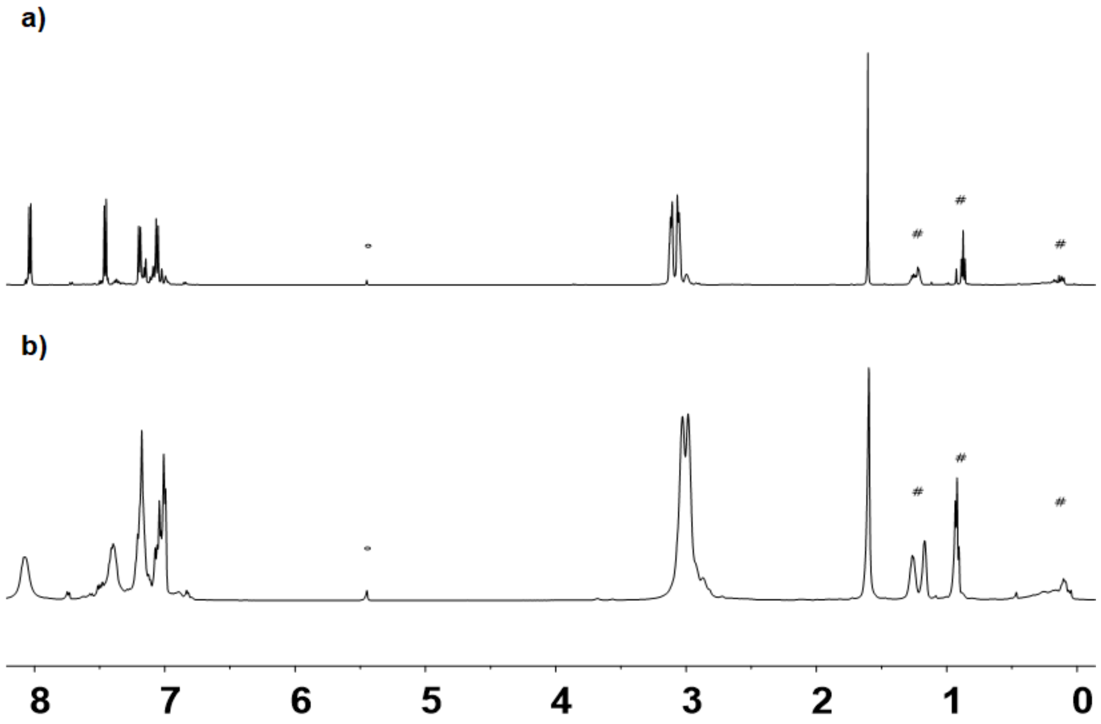

Figure S42 – ^{1}H NMR spectra (500 MHz, C_6D_5Cl) of siliconium borate **4c**$[B(C_6F_5)_4]$ # impurities possibly due to decomposition, ° triphenylmethane). a) at 305.0 K; b) at 232.5 K.

*bromonium ion **10***

A solution of 1-bromo-8-dimethylsilylnaphthalene **2** (1.2 equiv, 481 µmol, 128 mg) in benzene-d_6 was added to a solution of trityl borate (1.0 equiv, 401 µmol, 370 mg) in benzene-d_6 at r.t. and stirred for 30 min. Then, the upper, nonpolar phase was removed and the polar phase was washed with 0.5 mL benzene-d_6. Afterwards, bromonium borate **10**$[B(C_6F_5)_4]$ was analyzed by NMR spectroscopy. The sample showed a broad signal for the cation and was measured again several days later, where decomposition was already observed.

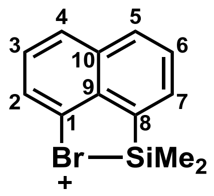

10 **$[B(C_6F_5)_4]^-$**

^{1}H NMR (499.87 MHz, 305.0 K, C_6D_6): δ = 0.55 (s, 6H, $Si(CH_3)_2$), 6.98 (d, 1H, $^3J_{H,H}$ = 7.8 Hz, H-2), 7.07-7.10 (m, 1H, H-3), 7.16-7.19 (m, 1H, H-7), 7.30-7.33 (m, 1H, H-6), 7.54 (d, 1H, $^3J_{H,H}$ = 8.1 Hz, H-4), 7.60 (d, 1H, $^3J_{H,H}$ = 8.1 Hz, H-5). **^{13}C{^{1}H} NMR** (125.77 MHz, 305.0K, C_6D_6): δ = 2.0 ($Si(CH_3)_2$, $^1J_{C,Si}$ = 54.6 Hz), 124.8 (brs, C, $[B(C_6F_5)_4]^-$), 126.1 (CH, C-2), 127.7-128.3 (C, C-8, overlapping with C_6D_6)), 128.5 (CH, C-3 or C-6), 128.6 (CH, C-3 or C-6), 129.2 (C, C1), 131.2 (CH, C-4), 132.8 (CH, C-5), 132.9 (C, C-9), 134.6 (CH, C-7), 135.8 (C, C-10), 137.0 (dm, $^1J_{C,F}$ = 241.0 Hz, CF, $[B(C_6F_5)_4]^-$), 139.0 (dm, $^1J_{C,F}$ = 240.2 Hz, CF, $[B(C_6F_5)_4]^-$), 149.0 (dm, $^1J_{C,F}$ = 241.0 Hz, CF, $[B(C_6F_5)_4]^-$). **^{29}Si{^{1}H} INEPT NMR** (99.31 MHz, 305.0 K, C_6D_6): δ = 94.6 ($^1J_{Si,C}$ = 72.4 Hz, $^1J_{Si,C}$ = 53.4 Hz). **^{29}Si{^{1}H} NMR** (99.31 MHz, 305.0 K, C_6D_6): δ = 94.6. **^{19}F{^{1}H} NMR** (470.30 MHz, 305.1 K, C_6D_6): δ = -167.5-(-167.2) (m, 8 F, *m*-F, $[B(C_6F_5)_4]^-$), -163.4 (t, $^3J_{F,F}$ = 20.5 Hz, 4 F, *p*-F, $[B(C_6F_5)_4]^-$), -133.1-(-132.5) (m, 8 F, *o*-F, $[B(C_6F_5)_4]^-$). **^{11}B{^{1}H} NMR** (160.38 MHz, 305.0K, C_6D_6): δ = -16.0.

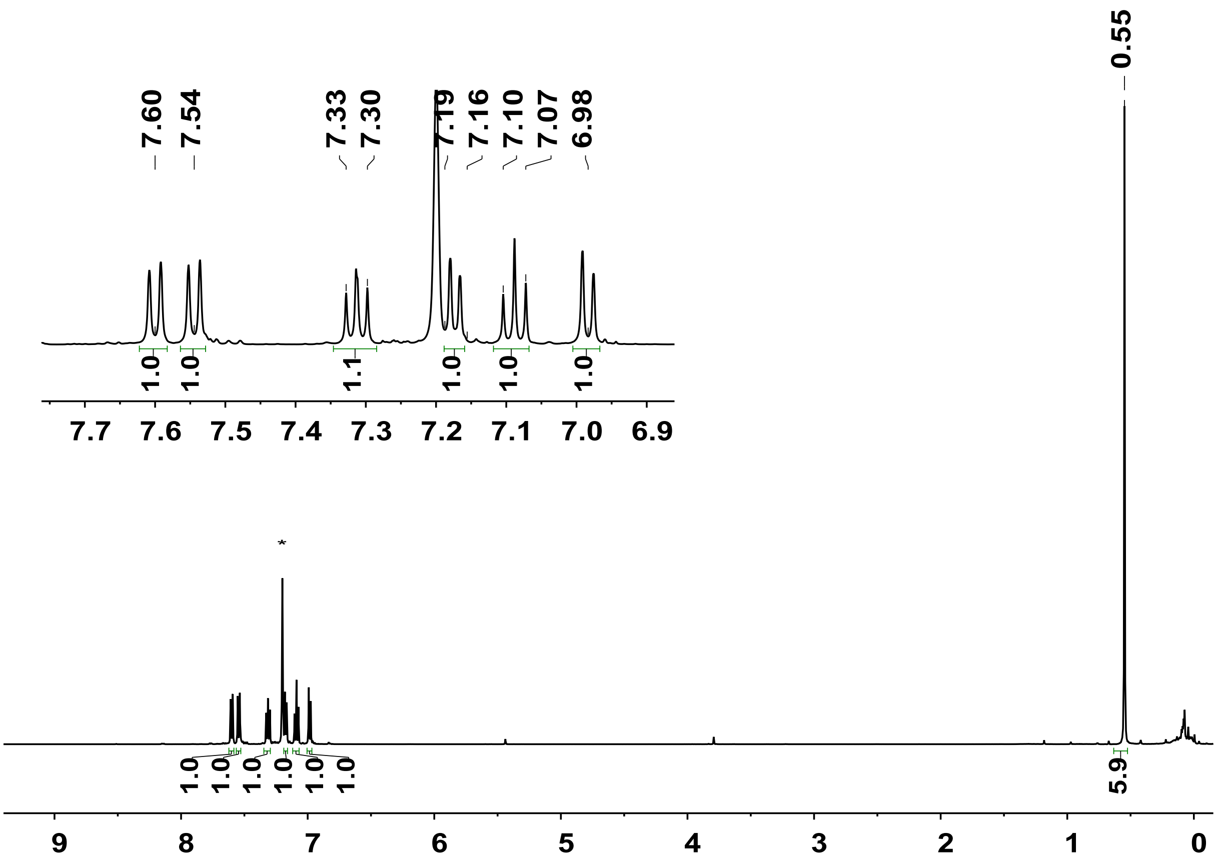

Figure S43 – ^{1}H NMR spectrum (499.87 MHz, 305.0 K, C_6D_6) of bromonium ion **10**[B(C_6F_5)$_4$] (* C_6D_5H).

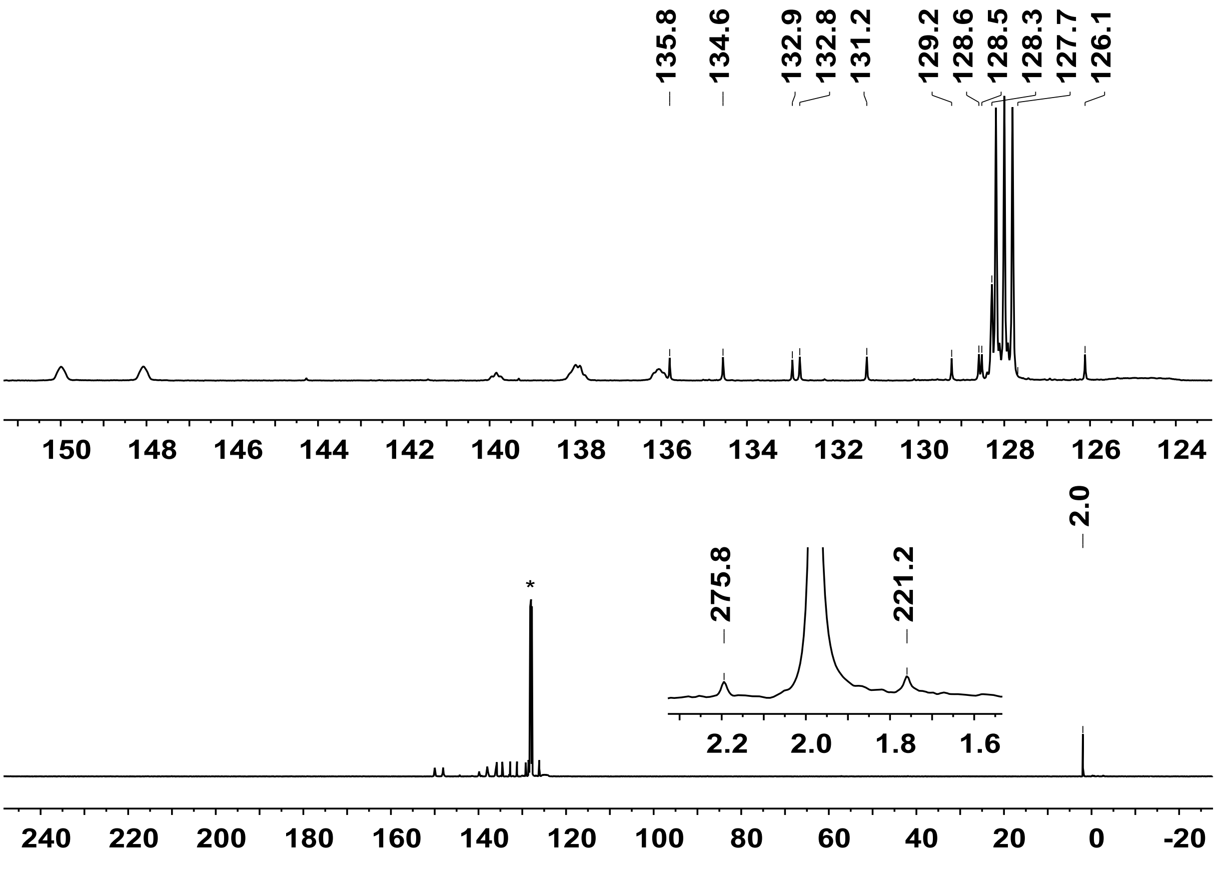

Figure S44 – ^{13}C{^{1}H} NMR spectrum (125.71 MHz, 305.0 K, C_6D_6) of bromonium ion **10**[B(C_6F_5)$_4$] (* C_6D_6).

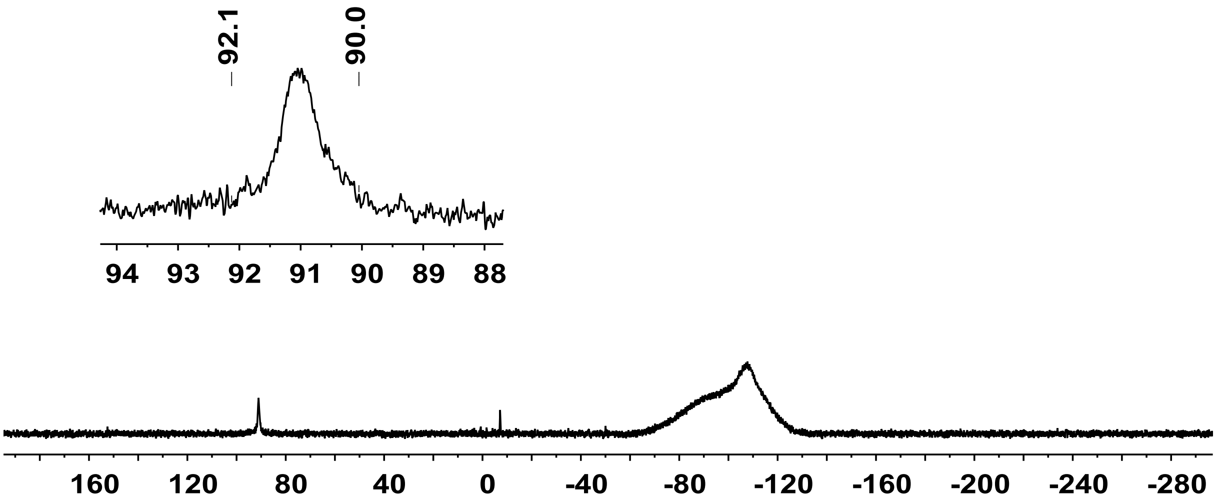

Figure S45 – $^{29}Si\{^1H\}$ NMR spectrum (99.36 MHz, 298.3 K, C_6D_6) of bromonium ion **10**[$B(C_6F_5)_4$] on the day of preparation.

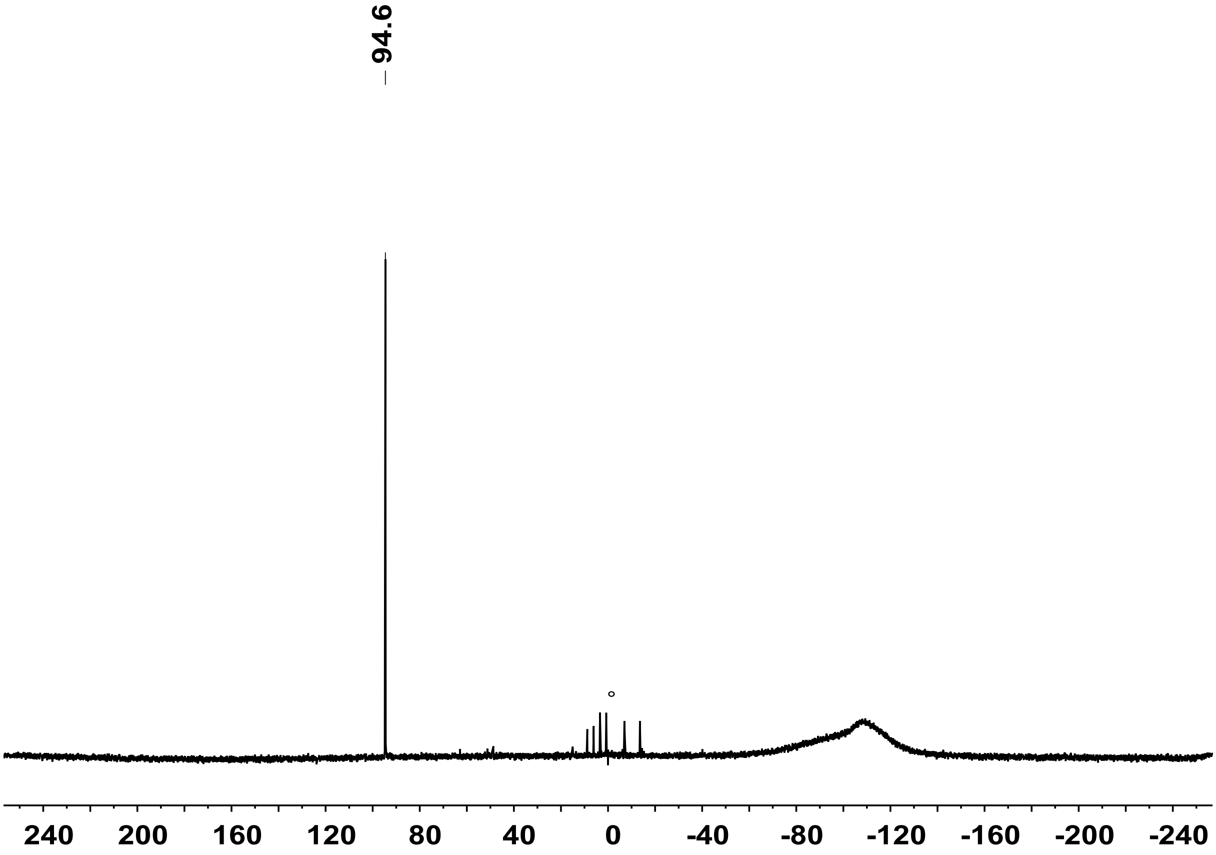

Figure S46 – $^{29}Si\{^1H\}$ NMR spectrum (99.31 MHz, 305.0K, C_6D_6) of bromonium ion **10**[$B(C_6F_5)_4$] several days later (°impurities possibly due to decomposition).

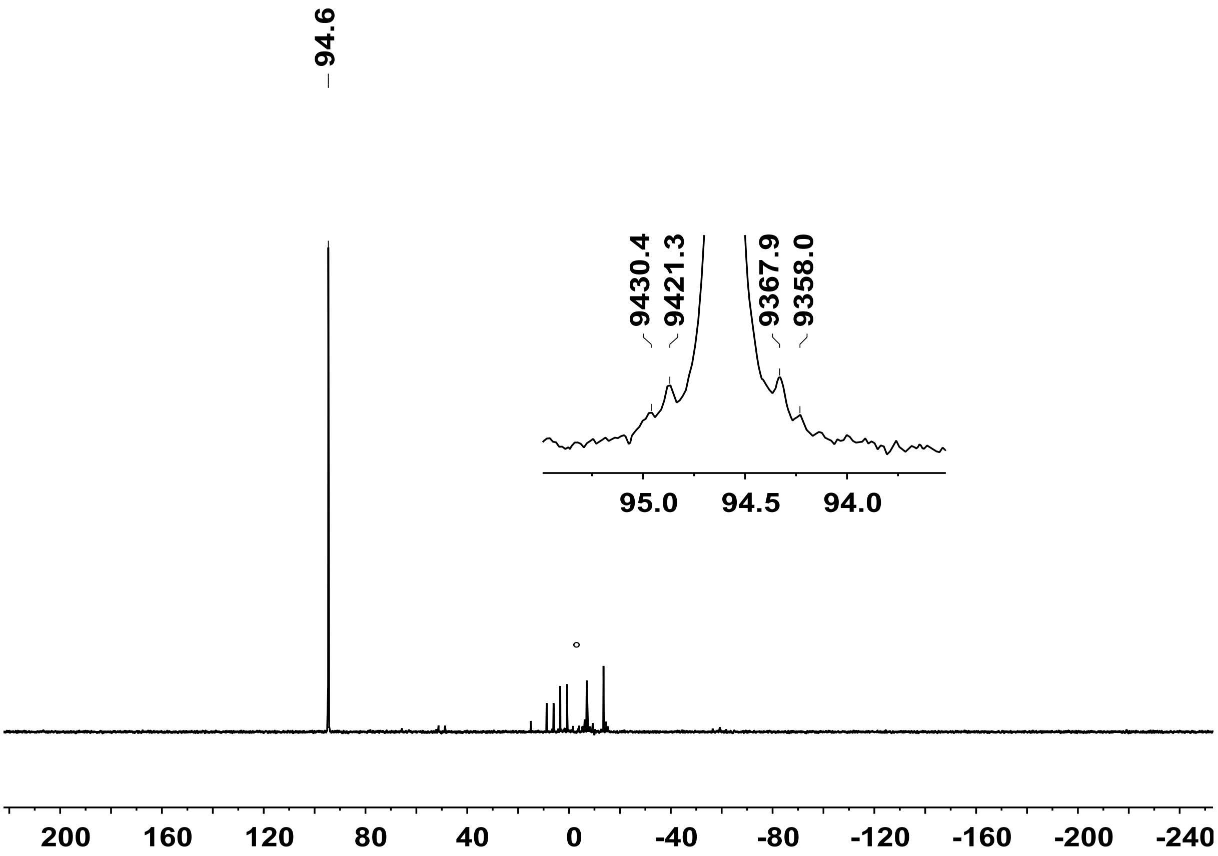

Figure S47 – $^{29}Si\{^1H\}$ INEPT NMR spectrum (99.31 MHz, 305.0 K, C_6D_6) of bromonium ion **10**[B(C_6F_5)$_4$] several days later (°impurities possibly due to decomposition).

*bromonium ion **10** and siliconium ion **11** and **21***

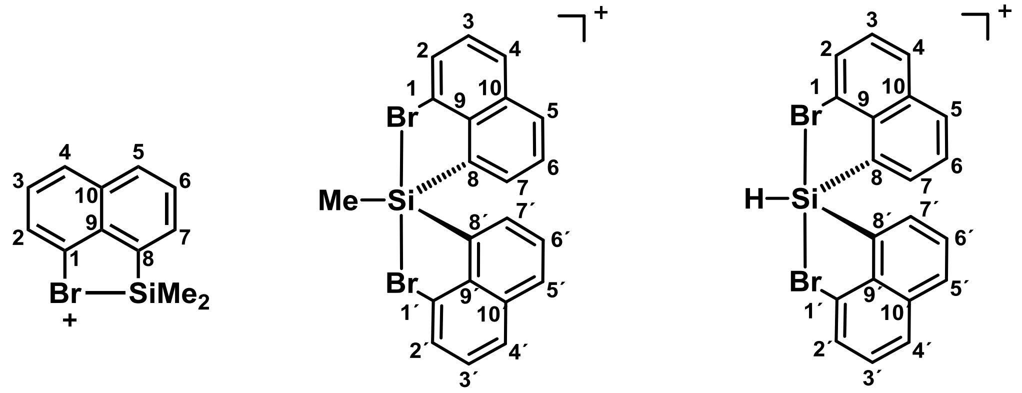

10 $[B(C_6F_5)_4]^-$ **11** $[B(C_6F_5)_4]^-$ **21** $[B(C_6F_5)_4]^-$

A solution of 1-bromo-8-dimethylsilylnaphthalene **2** (3.0 equiv, 1.20 mmol, 319 mg) in benzene-d_6 was added to a solution of trityl borate $[Ph_3C][B(C_6F_5)_4]$ (1.0 equiv, 401 µmol, 370 mg) in benzene-d_6 at r.t. and stirred for 30 min. The color of the reaction mixture changed from yellow orange to light brown. Then, the lower, polar phase of the biphasic reaction mixture was analyzed by NMR spectroscopy, which indicated the formation of bromonium borate **10**$[B(C_6F_5)_4]$ and siliconium borate **11**$[B(C_6F_5)_4]$ in the ratio of 64:36. After, the polar phase was transferred back to the nonpolar phase and the reaction mixture was heated at 40 °C for 1 h. Afterwards, the phases of the biphasic reaction mixture were analyzed separately by NMR spectroscopy. The upper, nonpolar phase did not show any evidence for 1-bromo-8-dimethylsilylnaphthalene **2**. The polar phase contained the bromonium borate **10**$[B(C_6F_5)_4]$ and siliconium borate **11**$[B(C_6F_5)_4]$ in the ratio of 28:72 and was first washed with benzene and then with a solvent mixture containing benzene and pentane in a ratio of 1:1. After removing the solvents under reduced pressure, the residue was dissolved in benzene-d_6 and analyzed by NMR spectroscopy again.

^{1}H NMR (499.87 MHz, 305.0 K, C_6D_6): δ = 1.19 (s, $SiCH_3$, **11**), 7.14 (t, $^3J_{H,H}$ = 8.0 Hz, 2H, H-3, H-3´, **11**), 7.23-7.28 (m, 2H, H-6, H-6´, **11**), 7.30-7.34 (m, 2H, H-2, H-2´, **11**, overlapping with bromonium ion **10**), 7.59 (d, $^3J_{H,H}$ = 8.0 Hz, 2H, H-4, H-4´, **11**), 7.64-7.69 (m, 2H, H-5, H-5´, **11**, overlapping with bromonium ion **10**), 7.75-7.81 (m, 2H, H-7, H-7´, **11**). **^{13}C{^{1}H} NMR** (125.71 MHz, 305.0 K, C_6D_6): δ = 13.3 ($SiCH_3$, **11**), 123.3 (C, C-1, C-1´, **11**), 125.0 (brs, C, $[B(C_6F_5)_4]^-$), 126.2 (C, C-8, C-8´, **11**), 127.1 (CH, C-6, C-6´, **11**), 128.3 (CH, C-3, C-3´, **11**, overlapping with C_6D_6), 128.9 (CH, C-2, C-2´, **11**), 131.1 (CH, C-4, C-4´, **11**), 134.6 (CH or C, C-5, C-5´ or C-9, C-9´, **11**), 134.7 (CH or C, C-5, C-5´ or C-9, C-9´, **11**), 136.1 (C, C-10, C-10´, **11**), 137.0 (dm, $^1J_{C,F}$ = 240.5 Hz, CF, $[B(C_6F_5)_4]^-$), 138.9 (dm, $^1J_{C,F}$ = 239.7 Hz, CF, $[B(C_6F_5)_4]^-$), 140.1 (CH, C-7, C-7´, **11**), 149.1 (dm, $^1J_{C,F}$ = 240.9 Hz, CF, $[B(C_6F_5)_4]^-$). **^{29}Si{^{1}H} NMR** (99.31 MHz,

305.0 K, C_6D_6): δ = 1.1 (**21**), 62.9 (**11**), 95.0 (**10**). **^{19}F{^{1}H} NMR** (470.30 MHz, 305.0 K, C_6D_6): δ = -166.5-(-166.2) (m, 8 F, *m*-F, $[B(C_6F_5)_4]^-$), -162.4 (t, $^{3}J_{F,F}$ = 20.8 Hz, 4 F, *p*-F, $[B(C_6F_5)_4]^-$), -132.1-(-131.7) (m, 8 F, *o*-F, $[B(C_6F_5)_4]^-$). **^{11}B{^{1}H} NMR** (160.38 MHz, 305.1 K, C_6D_6): δ = -16.0.

^{29}Si NMR spectra recorded with the INEPT pulse sequence with parameter sensitive to Si-H groups with large coupling constants were used to identify the by-product with δ^{29}Si = 1.1 (see Figure S52).

^{29}Si{^{1}H} INEPT NMR (99.31 MHz, 305.0 K, C_6D_5Cl): δ = 0.8. **^{29}Si INEPT NMR** (99.31 MHz, 305.0 K, C_6D_5Cl): δ = 0.8 ($^{1}J_{Si,H}$ = 328 Hz).

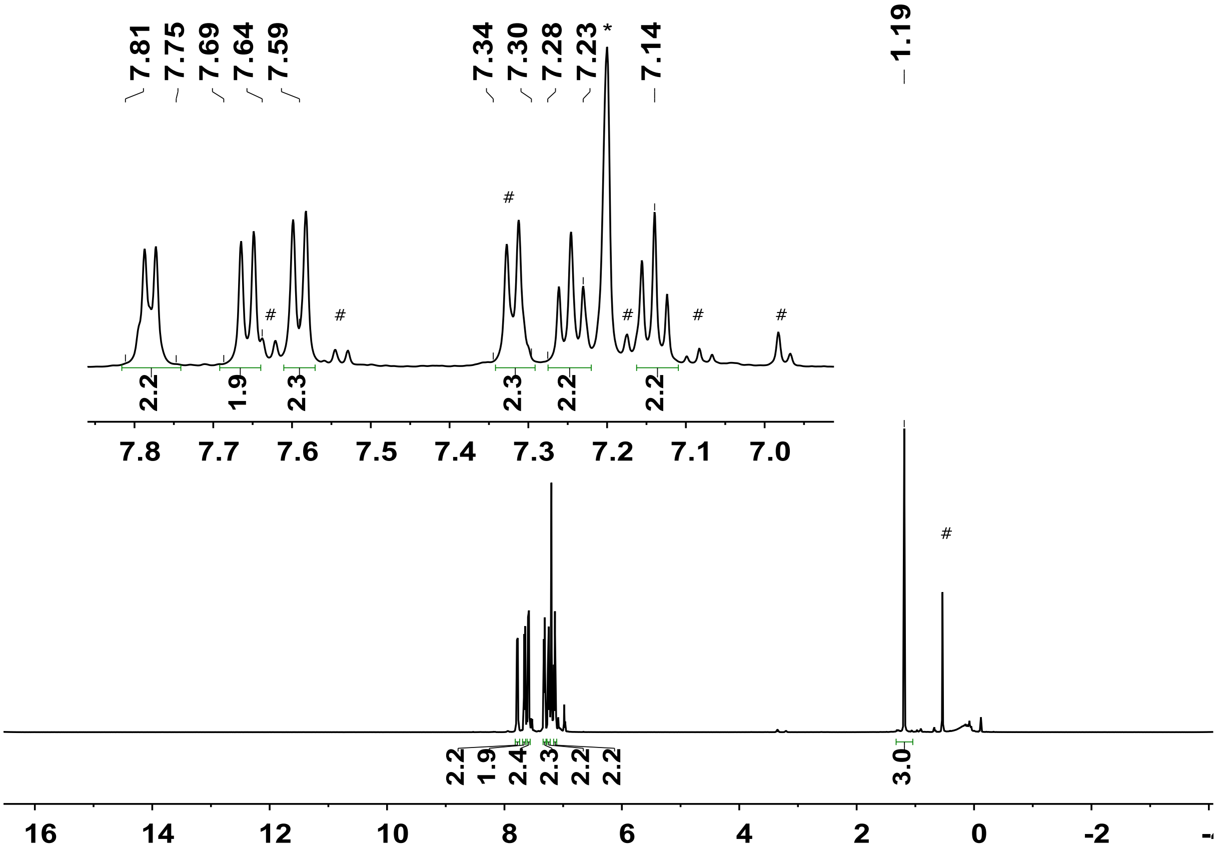

Figure S48 – ^{1}H NMR spectrum (499.87 MHz, 305.0 K, C_6D_6) of the reaction mixture; signals of siliconium borate **11**$[B(C_6F_5)_4]$ are picked (* C_6D_5H, # bromonium borate **10**$[B(C_6F_5)_4]$).

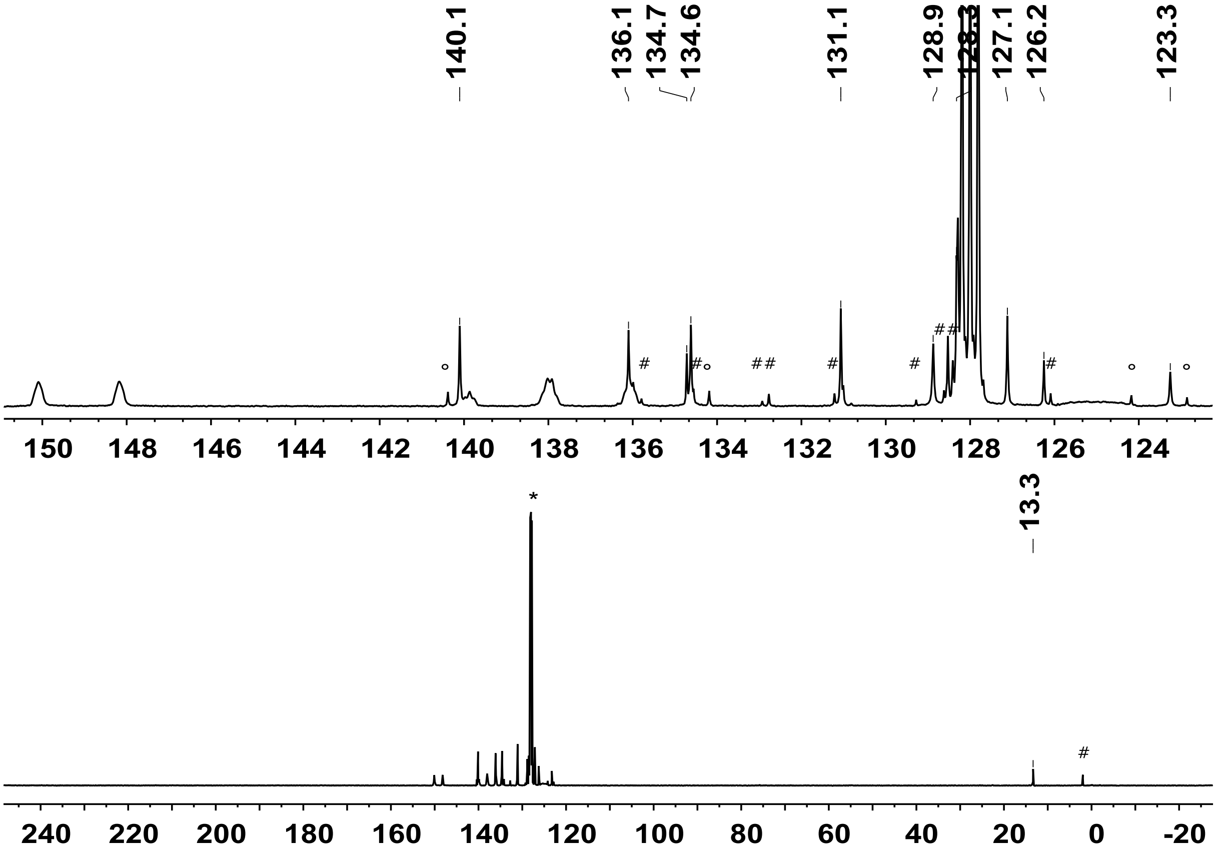

Figure S49 – $^{13}C\{^1H\}$ NMR spectrum (125.71 MHz, 305.0 K, C_6D_6) of the reaction mixture; signals of siliconium borate **11**[B(C_6F_5)$_4$] are picked (* C_6D_5H, # bromonium borate **10**[B(C_6F_5)$_4$], °impurities that belong to siliconium borate **21** [B(C_6F_5)$_4$]).

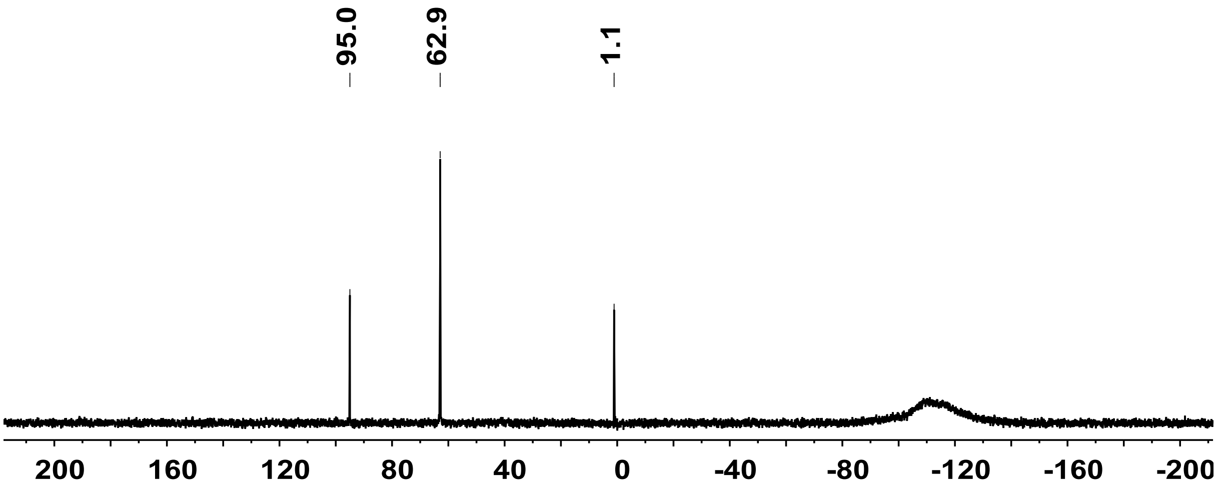

Figure S50 – $^{29}Si\{^1H\}$ NMR spectrum (99.31 MHz, 305.0K, C_6D_6) of the reaction mixture including bromonium borate **10**[B(C_6F_5)$_4$] ($\delta^{29}Si$ = 95.0), siliconium borate **11**[B(C_6F_5)$_4$] ($\delta^{29}Si$ = 62.9) and siliconium borate **21**[B(C_6F_5)$_4$] ($\delta^{29}Si$ = 1.1).

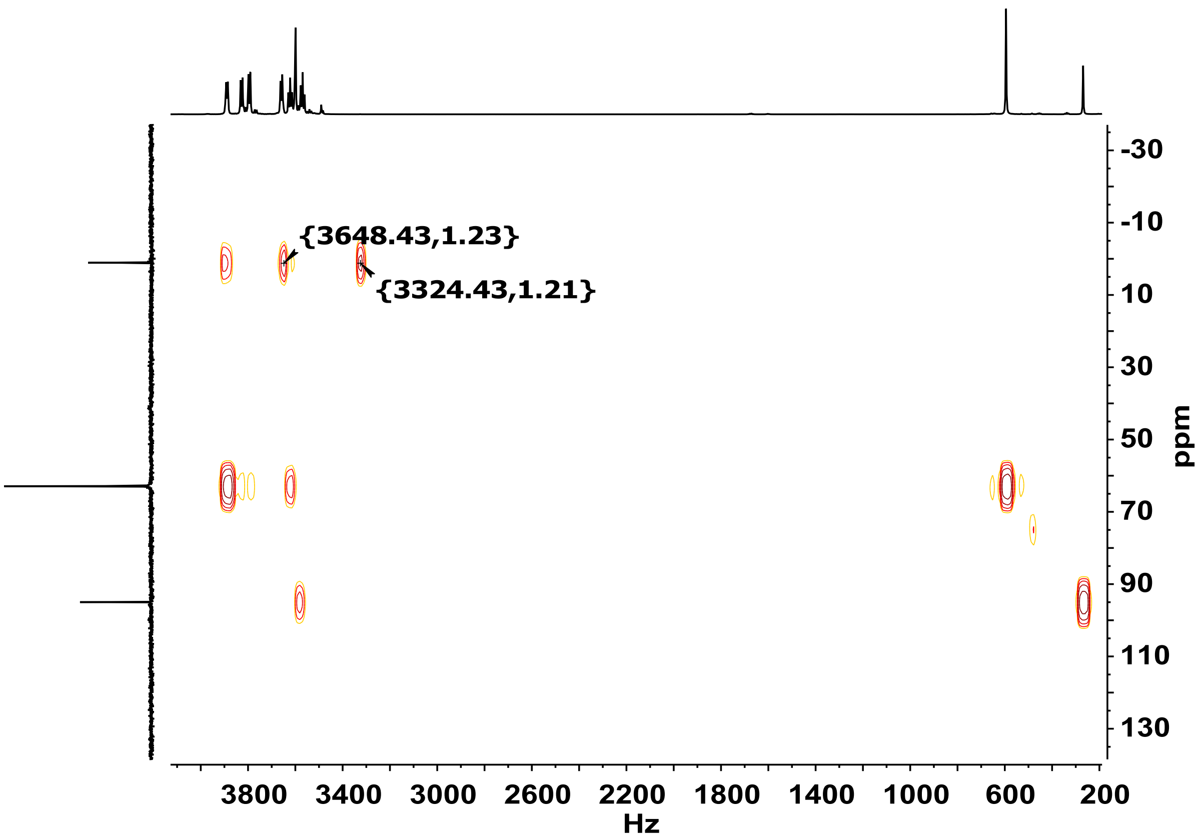

Figure S51 –^{1}H^{29}Si HMBC NMR spectrum (499.87 MHz, 305.0K, C_6D_6) of the reaction mixture including bromonium borate **10**[$B(C_6F_5)_4$] (δ^{29}Si = 95.0), siliconium borate **11**[$B(C_6F_5)_4$] (δ^{29}Si = 62.9) and siliconium borate **21**[$B(C_6F_5)_4$] (δ^{29}Si = 1.2).

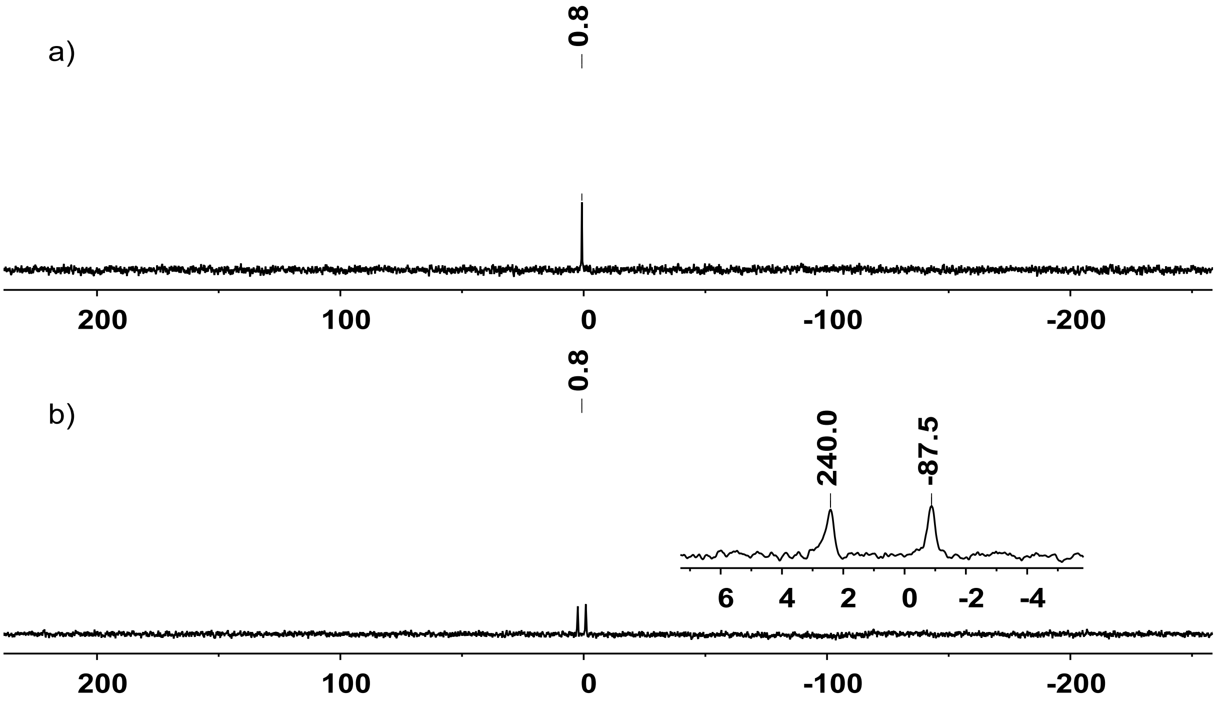

Figure S52 – a) ^{29}Si{^{1}H} INEPT NMR spectrum (99.31 MHz, 305.0 K, C_6D_5Cl) and b) ^{29}Si INEPT NMR spectrum (99.31 MHz, 305.0 K, C_6D_5Cl) of siliconium borate **21**[$B(C_6F_5)_4$] (lb = 10, gb = 1.0), optimized for Si-H ($^{1}J_{Si,H}$ = 324 Hz).

*iodonium ion **3d***

A reaction mixture containing 5-iodo-6-dimethylsilylacenaphthene **1d** as main product (with 5-iodoacenaphthene and a small amount of 5-dimethylsilylacenaphthene [S3]) was used for the synthesis of iodonium ion **3d**. To ensure a small excess of the silane **1d** compared to the trityl borate for complete conversion of the trityl salt, 170 mg of the reaction mixture (containing 1.03 equiv, 414 µmol of the silane **1d**) in 1 mL benzene-d_6 was added to a solution of trityl borate (401 µmol, 370 mg) in 0.5 mL benzene-d_6 at r.t. and stirred for 30 min. The upper, nonpolar phase was removed and iodonium borate **3d**[$B(C_6F_5)_4$] was analyzed by NMR spectroscopy.

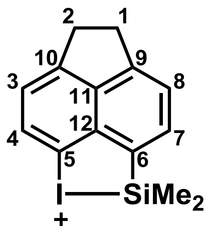

3d **$[B(C_6F_5)_4]^-$** ii

^{1}H NMR (499.87 MHz, 305.0 K, C_6D_6): δ = 0.71 (s, 6H, $Si(CH_3)_2$), 2.92-3.05 (m, 4H, CH_2, H-1, H-2), 6.91 (d, 1H, $^3J_{H,H}$ = 7.7 Hz, H-3), 6.98 (d, 1H, $^3J_{H,H}$ = 7.7 Hz, H-4), 7.07 (d, 1H, $^3J_{H,H}$ = 7.3 Hz, H-8), 7.16 (d, 1H, $^3J_{H,H}$ = 7.3 Hz, H-7). **^{13}C{^{1}H} NMR** (125.77 MHz, 305.0K, C_6D_6): δ = 3.9 ($Si(CH_3)_2$, $^1J_{C,Si}$ = 53.7 Hz), 30.0 (CH_2, C-2), 30.9 (CH_2,C-1), 103.3 (C, C-5), 122.2 (CH, C-8), 123.5 (CH, C-3), 125.0 (brs, C, $[B(C_6F_5)_4]^-$), 127.6 (C, C-6), 132.7 (CH, C-4), 136.5 (CH, C-7), 137.1 (dm, $^1J_{C,F}$ = 239.5 Hz, CF, $[B(C_6F_5)_4]^-$), 137.5 (C, C-12), 139.0 (dm, $^1J_{C,F}$ = 239.5 Hz, CF, $[B(C_6F_5)_4]^-$), 141.7 (C, C-11), 149.1 (dm, $^1J_{C,F}$ = 240.5 Hz, CF, $[B(C_6F_5)_4]^-$), 151.4 (C, C-10), 153.0 (C, C-9). **^{29}Si{^{1}H} NMR** (99.31 MHz, 305.0 K, C_6D_6): δ = 89.5 ($^1J_{Si,C}$ = 72.4 Hz, $^1J_{Si,C}$ = 53.7 Hz). **^{29}Si{^{1}H} INEPT NMR** (99.31 MHz, 305.0 K, C_6D_6): δ = 89.5 ($^1J_{Si,C}$ = 72.2 Hz, $^1J_{Si,C}$ = 53.9 Hz). **^{19}F{^{1}H} NMR** (470.30 MHz, 305.1 K, C_6D_6): δ = -167.5-(-167.2) (m, 8 F, *m*-F, $[B(C_6F_5)_4]^-$), -163.4 (t, $^3J_{F,F}$ = 20.5 Hz, 4 F, *p*-F, $[B(C_6F_5)_4]^-$), -133.1-(-132.5) (m, 8 F, *o*-F, $[B(C_6F_5)_4]^-$). **^{11}B{^{1}H} NMR** (160.38 MHz, 305.0K, C_6D_6): δ = -16.0.

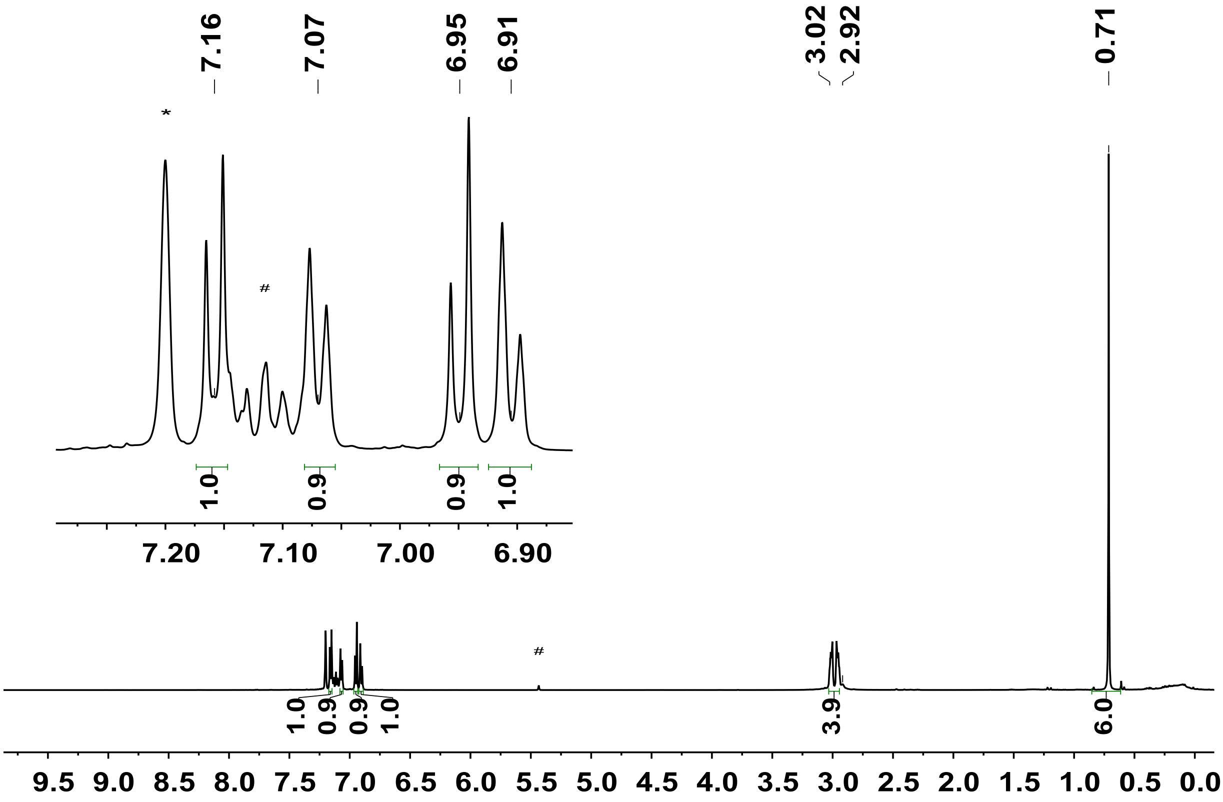

Figure S53 – ^{1}H NMR spectrum (499.87 MHz, 305.0 K, C_6D_6) of iodonium borate **3d**[$B(C_6F_5)_4$] (* C_6D_5H, # triphenylmethane).

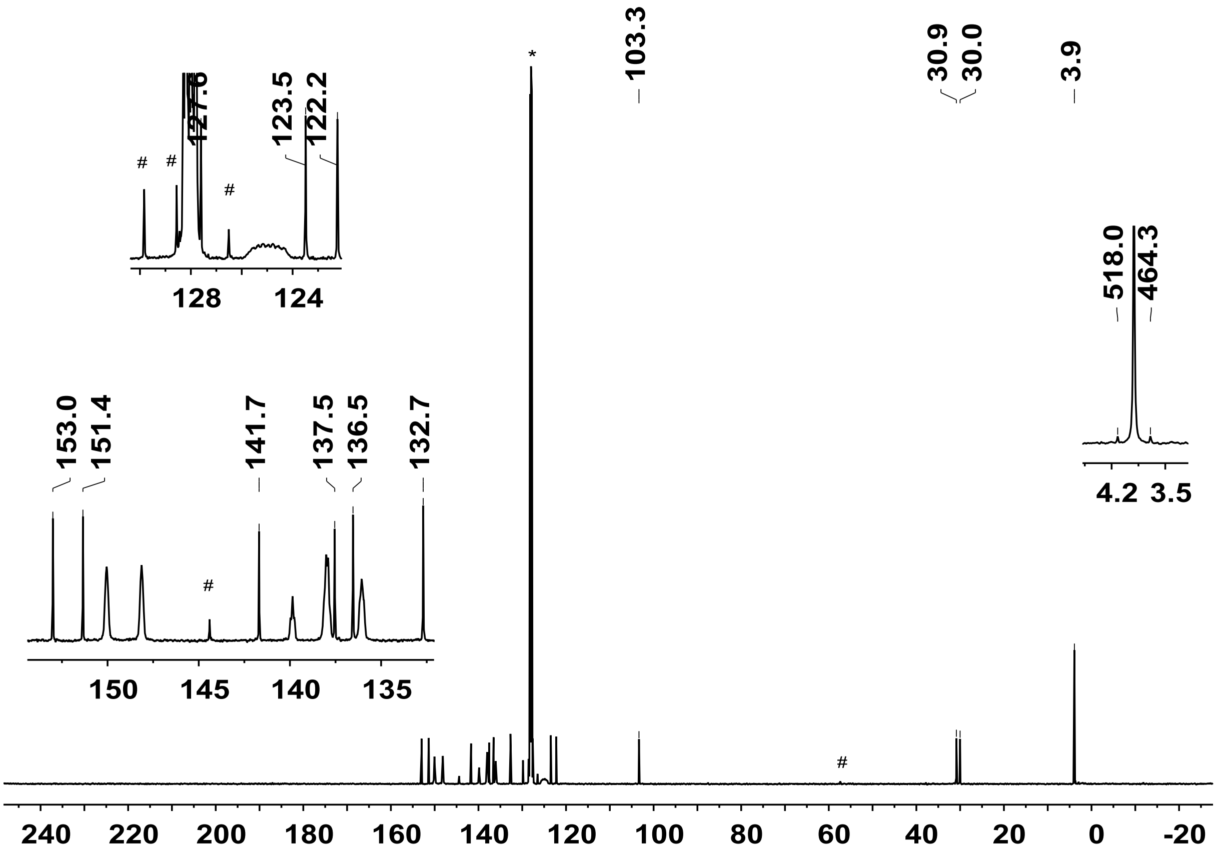

Figure S54 – $^{13}C\{^{1}H\}$ NMR spectrum (125.71 MHz, 305.0 K, C_6D_6) of iodonium borate **3d**[$B(C_6F_5)_4$] (* C_6D_6, # triphenylmethane).

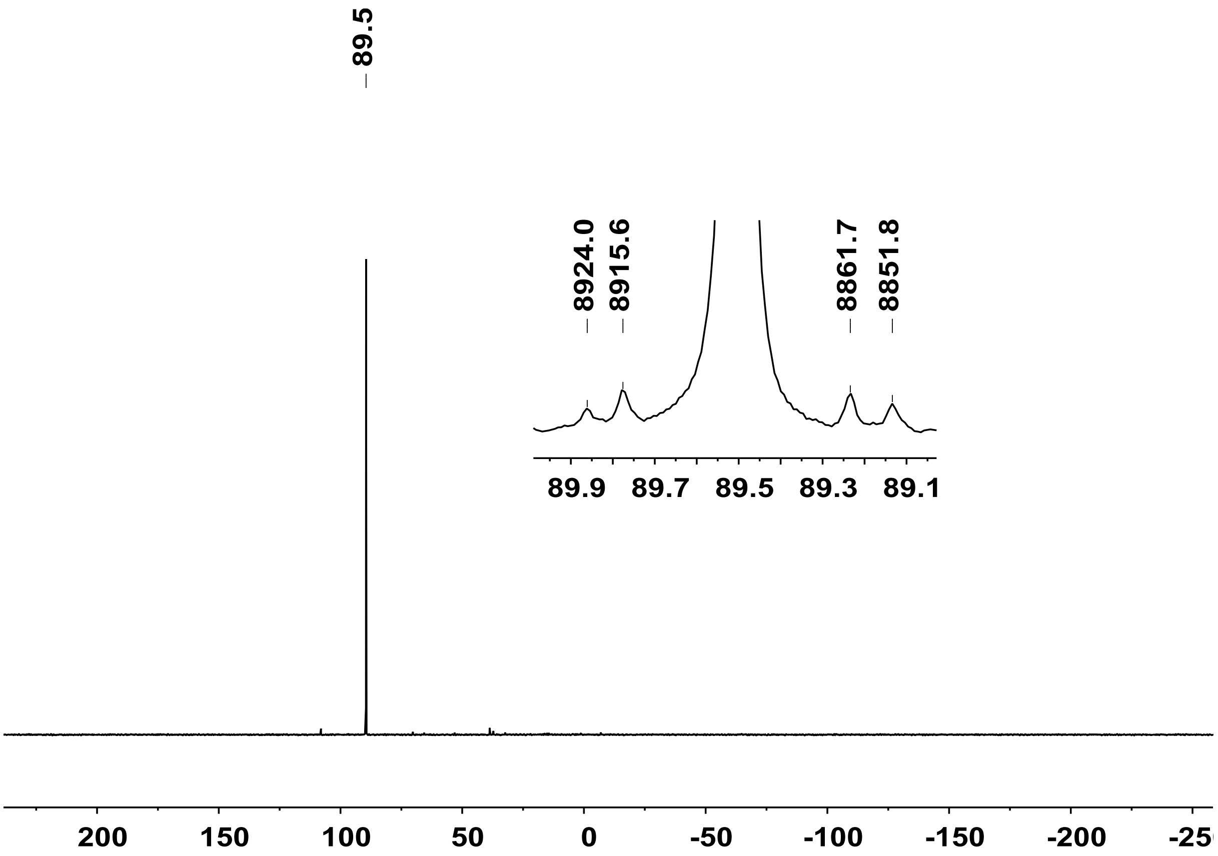

Figure S55 – $^{29}Si\{^1H\}$ INEPT NMR spectrum (99.31 MHz, 305.0 K, C_6D_6) of iodonium borate **3d**[$B(C_6F_5)_4$].

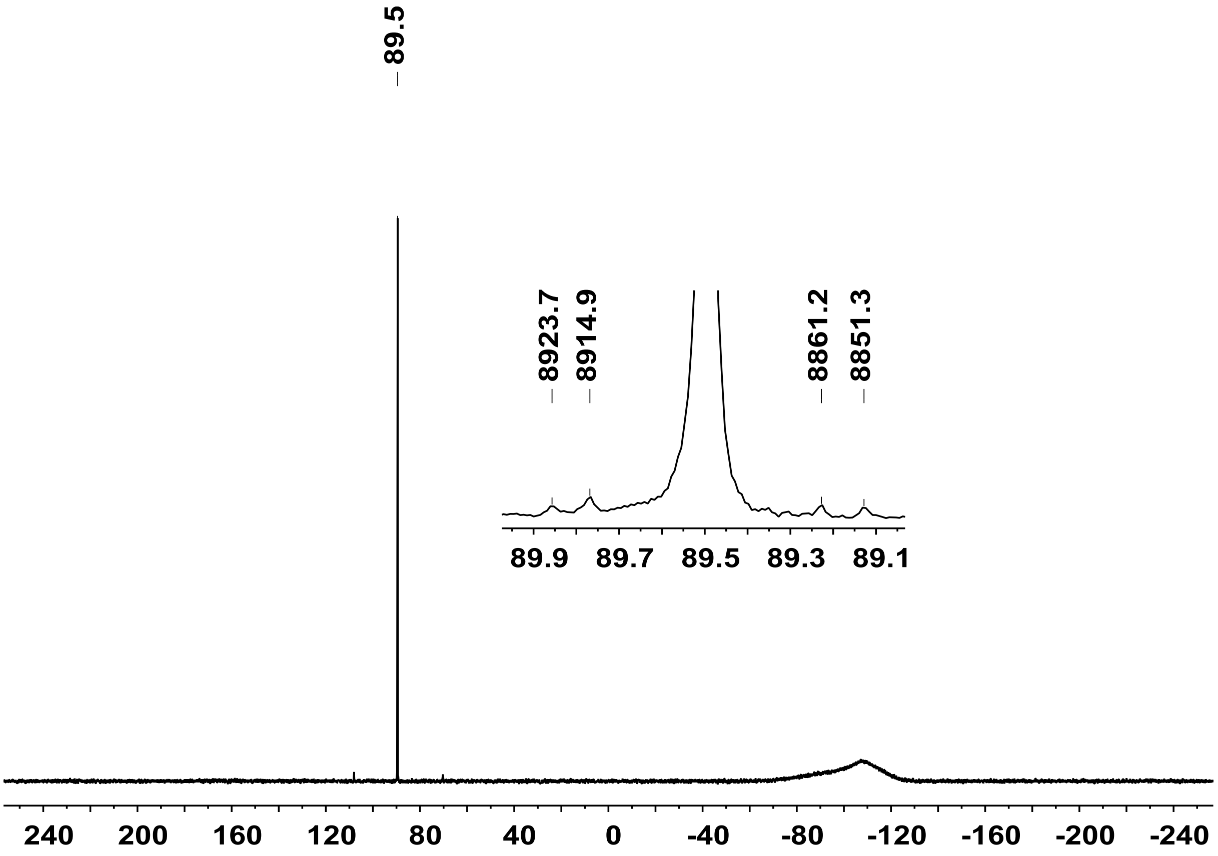

Figure S56 – $^{29}Si\{^1H\}$ NMR spectrum (99.31 MHz, 305.0K, C_6D_6) of iodonium borate **3d**[$B(C_6F_5)_4$].

4 Synthesis of Nitrilium Ions

General procedure A: A solution of trityl borate $[Ph_3C][B(C_6F_5)_4]$ in benzene was added to a solution of the corresponding silane and 4-fluorobenzonitrile in benzene. During the addition, the reaction mixture was cooled with cold water and the biphasic reaction mixture was stirred for 30 min at room temperature. Subsequently, the phases were separated, the upper, nonpolar phase was removed and the polar phase was washed twice with benzene and then with *n*-pentane. After removing the solvent under reduced pressure, the residue was dissolved in benzene-d_6 and analyzed by NMR spectroscopy.

General procedure B: Trityl borate $[Ph_3C][B(C_6F_5)_4]$ and 4-fluorobenzonitrile were dissolved in benzene-d_6. A solution of the corresponding silane in benzene-d_6 was added in small portions to the mixture, while it was cooled with cold water, and stirred for 30 min at room temperature. After the NMR spectroscopic analysis of the polar phase, the NMR sample was transferred into a Schlenk tube and washed with *n*-pentane. The solvent was removed under reduced pressure, the residue was dissolved in benzene-d_6 and analyzed by NMR spectroscopy.

General procedure C: After the synthesis of the corresponding halonium ion, as described in **1.4**, 4-fluorobenzonitrile was added. Afterwards, the reaction mixture was stirred for 15 min and nitrilium borate **16**$[B(C_6F_5)_4]$ was subsequently analyzed by NMR spectroscopy.

*nitrilium ion **16a***

The nitrilium ion **16a**[B(C_6F_5)$_4$] was synthesized according to general procedure **A** using 6-fluoro-5-dimethylsilylacenaphthene **1a** (1.0 equiv, 401 µmol, 93 mg), trityl borate (1.0 equiv, 401 µmol, 370 mg) and 4-fluorobenzonitrile (1.0 equiv, 401 µmol, 49 mg).

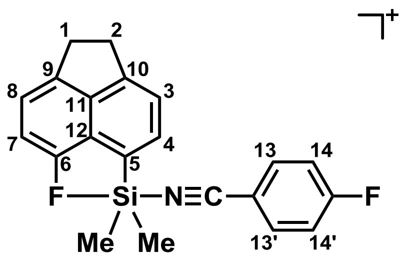

16a **[B(C_6F_5)$_4$]$^-$**

^{1}H NMR (499.87 MHz, 305.0 K, C_6D_6): δ = 0.70 (d, $^3J_{H,F}$ = 2.6 Hz, 6 H, Si(CH_3)$_2$), 2.96-3.14 (m, 4H, CH_2, H-1, H-2), 6.59 (t, $^3J_{H,H}$ = 8.3 Hz, 2 H, H-14, H-14'), 7.01-7.05 (m, 1 H, H-8), 7.05-7.09 (m, 2 H, H-13, H-13'), 7.13-7.20 (m, 2 H, H-7, H-3, overlapping with trityl cation), 7.56 (d, $^3J_{H,H}$ = 7.0 Hz, 1 H, H-4). **^{13}C{^{1}H} NMR** (125.71 MHz, 305.0 K, C_6D_6): δ = -1.8 (d, $^2J_{C,F}$ = 6.5 Hz, $^1J_{C,Si}$ = 63.4 Hz, Si(CH_3)$_2$), 29.5 (s, CH_2), 31.3 (s, CH_2), 97.3 (s, C, C-CN), 113.9 (d, $^2J_{C,F}$ = 22.4 Hz, CH, C-7), 114.3-114.6 (m, C, C-5), 118.5 (d, $^2J_{C,F}$ = 23.5 Hz, CH, C-14, C-14'), 120.3 (s, CH, C-3), 120.9 (d, $^3J_{C,F}$ = 7.7 Hz, CH, C-8), 123.1 (s, C, CN), 123.4 (d, $^2J_{C,F}$ = 18.3 Hz, C, C-12), 125.3 (brs, C, [B(C_6F_5)$_4$]$^-$), 137.0 (dm, $^1J_{C,F}$ = 240.2 Hz, CF, [B(C_6F_5)$_4$]$^-$), 138.3 (d, $^3J_{C,F}$ = 11.3 Hz, CH, C-13, C-13'), 138.6 (s, CH, C-4), 138.9 (dm, $^1J_{C,F}$ = 239.8 Hz, CF, [B(C_6F_5)$_4$]$^-$), 141.1 (d, $^3J_{C,F}$ = 7.4 Hz, C, C-11), 144.3 (d, $^4J_{C,F}$ = 2.1 Hz, C, C-9), 149.1 (dm, $^1J_{C,F}$ = 241.3 Hz, CF, [B(C_6F_5)$_4$]$^-$), 153.9 (d, $^4J_{C,F}$ = 2.2 Hz, C, C-10), 156.3 (d, $^1J_{C,F}$ = 241.1 Hz, CF, C-6), 169.1 (d, $^1J_{C,F}$ = 269.8 Hz, CF, F-Ph). **^{19}F{^{1}H} NMR** (470.29 MHz, 305.0 K, C_6D_6): δ = -167.5-(-167.2) (m, 8 F, *m*-F, [B(C_6F_5)$_4$]$^-$), -163.4 (t, $^3J_{F,F}$ = 20.4 Hz, 4 F, *o*-F, [B(C_6F_5)$_4$]$^-$), -133.0-(-132.8) (m, 8 F, *o*-F, [B(C_6F_5)$_4$]$^-$), -122.1 (s, 1F, CF, F-6), -87.8 (brs, 1 F, CF, F-Ph). **^{29}Si{^{1}H} INEPT NMR** (99.31 MHz, 305.0 K, C_6D_6): δ = 22.1 ($^1J_{Si,C}$ = 82.9 Hz, $^1J_{Si,C}$ = 63.0 Hz). **^{29}Si{^{1}H} NMR** (99.31 MHz, 305.0 K, C_6D_6): δ = 22.1. **^{11}B{^{1}H} NMR** (160.38 MHz, 305.0 K, C_6D_6): δ = -16.1.

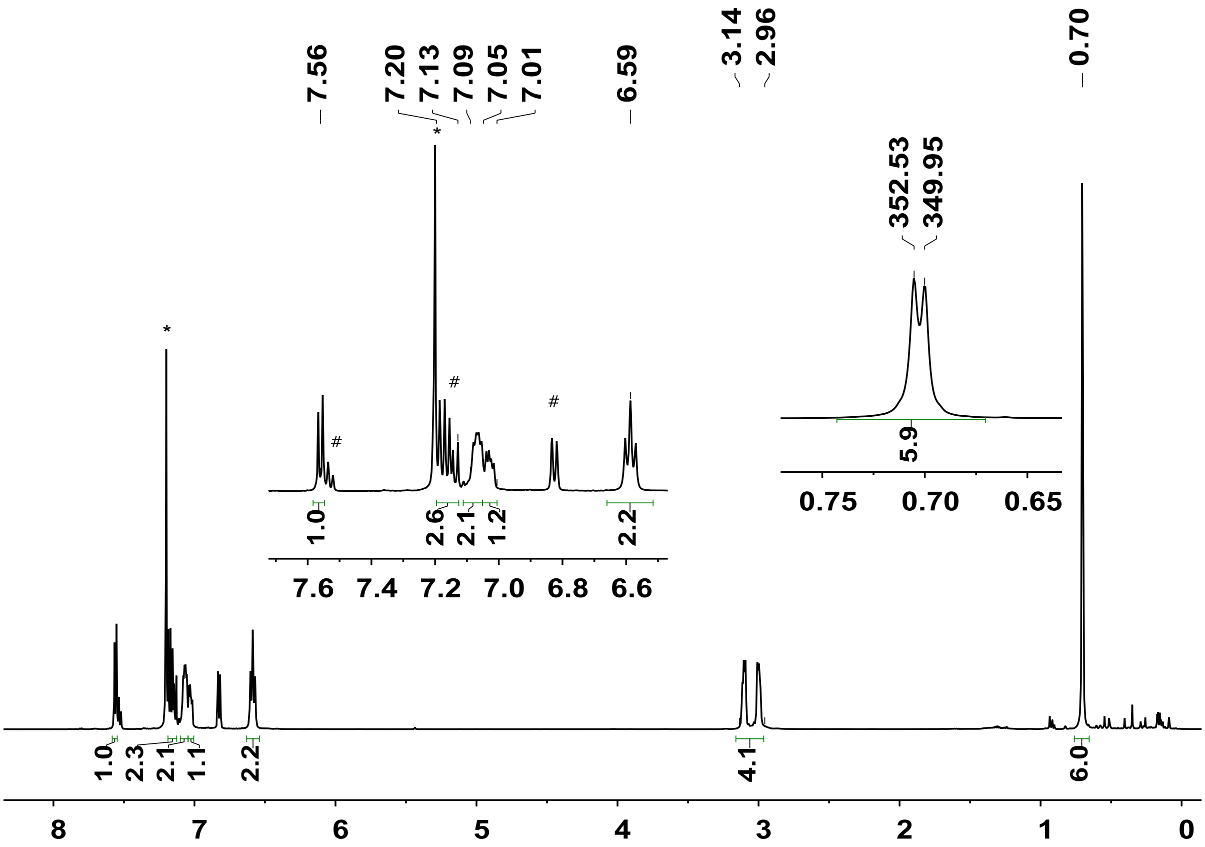

Figure S57 – ^{1}H NMR spectrum (499.87 MHz, 305.0 K, C_6D_6) of nitrilium borate **16a**[B(C_6F_5)$_4$] (* C_6D_5H, # trityl cation).

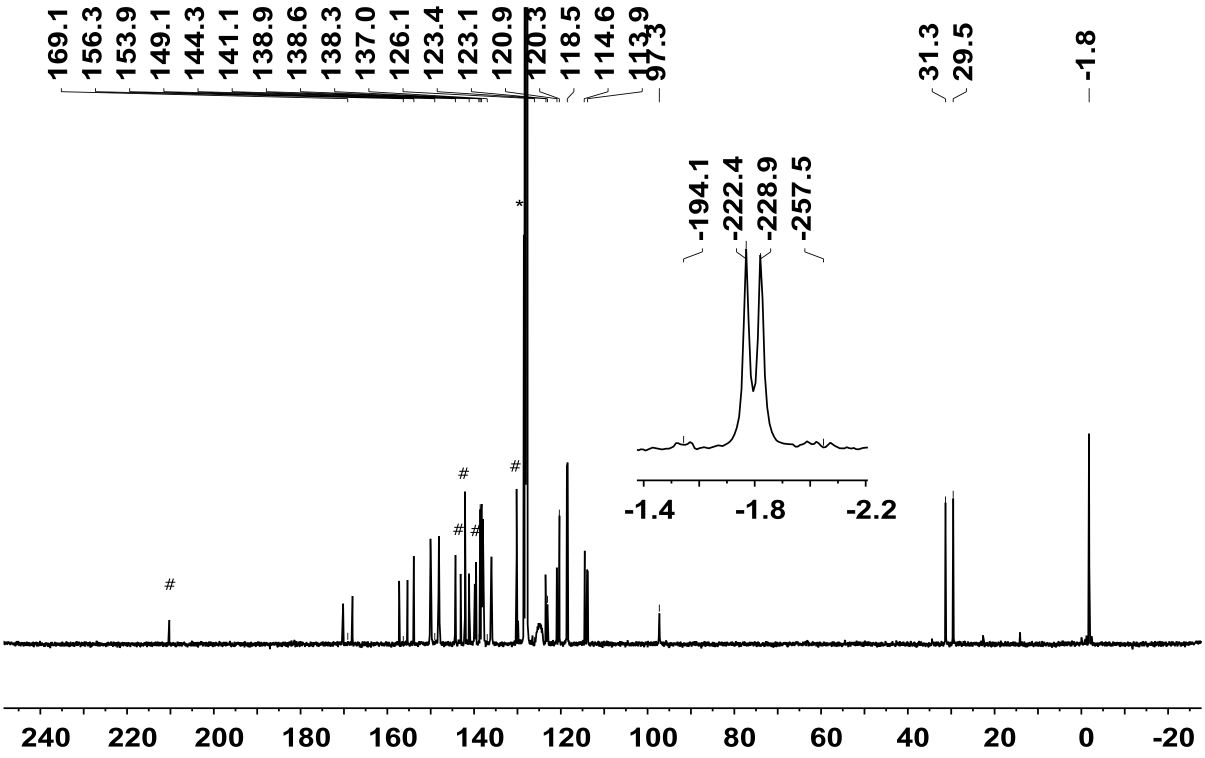

Figure S58 – ^{13}C{^{1}H} NMR spectrum (125.71 MHz, 305.0 K, C_6D_6) of nitrilium borate **16a**[B(C_6F_5)$_4$] ([#]trityl cation, *C_6H_6).

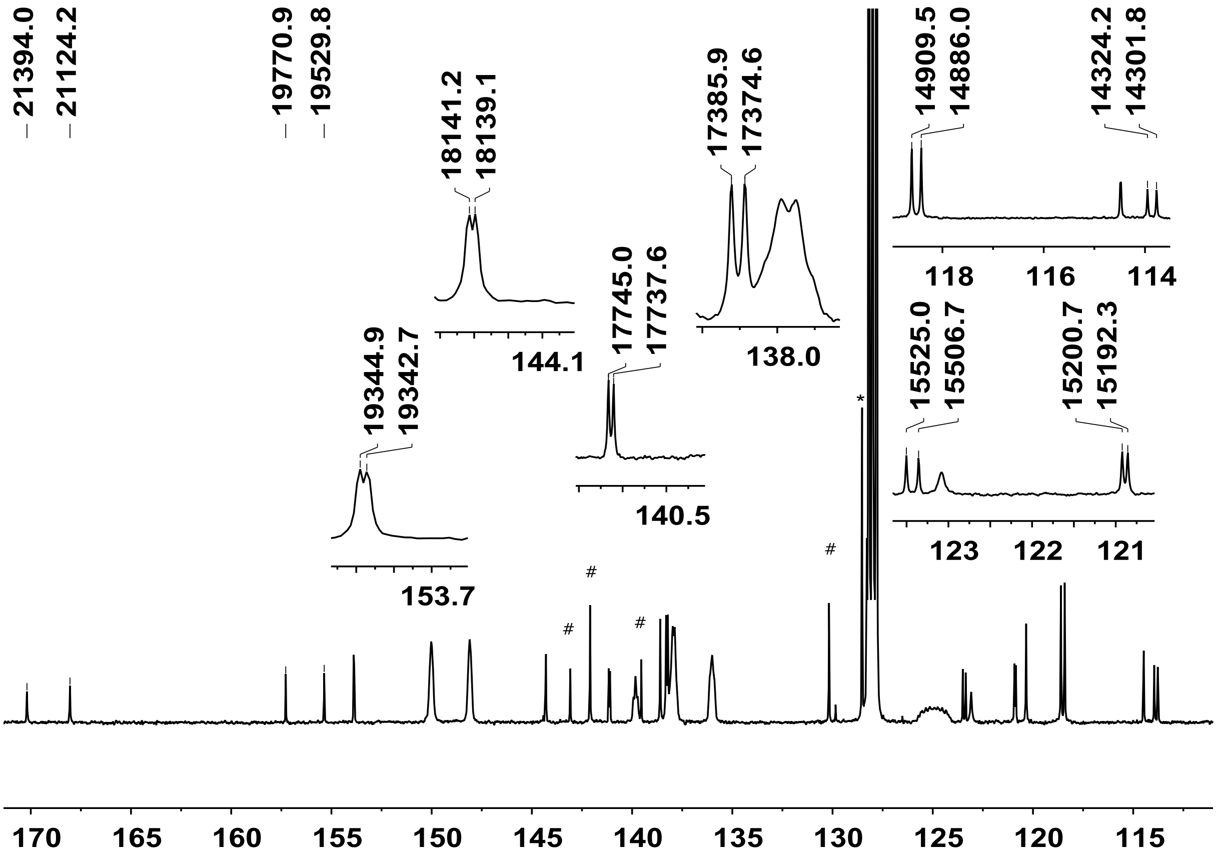

Figure S59 – Part of the $^{13}C\{^{1}H\}$ NMR spectrum (125.71 MHz, 305.0 K, C_6D_6) of nitrilium borate **16a** [$B(C_6F_5)_4$] ($^{\#}$ trityl cation, *C_6H_6).

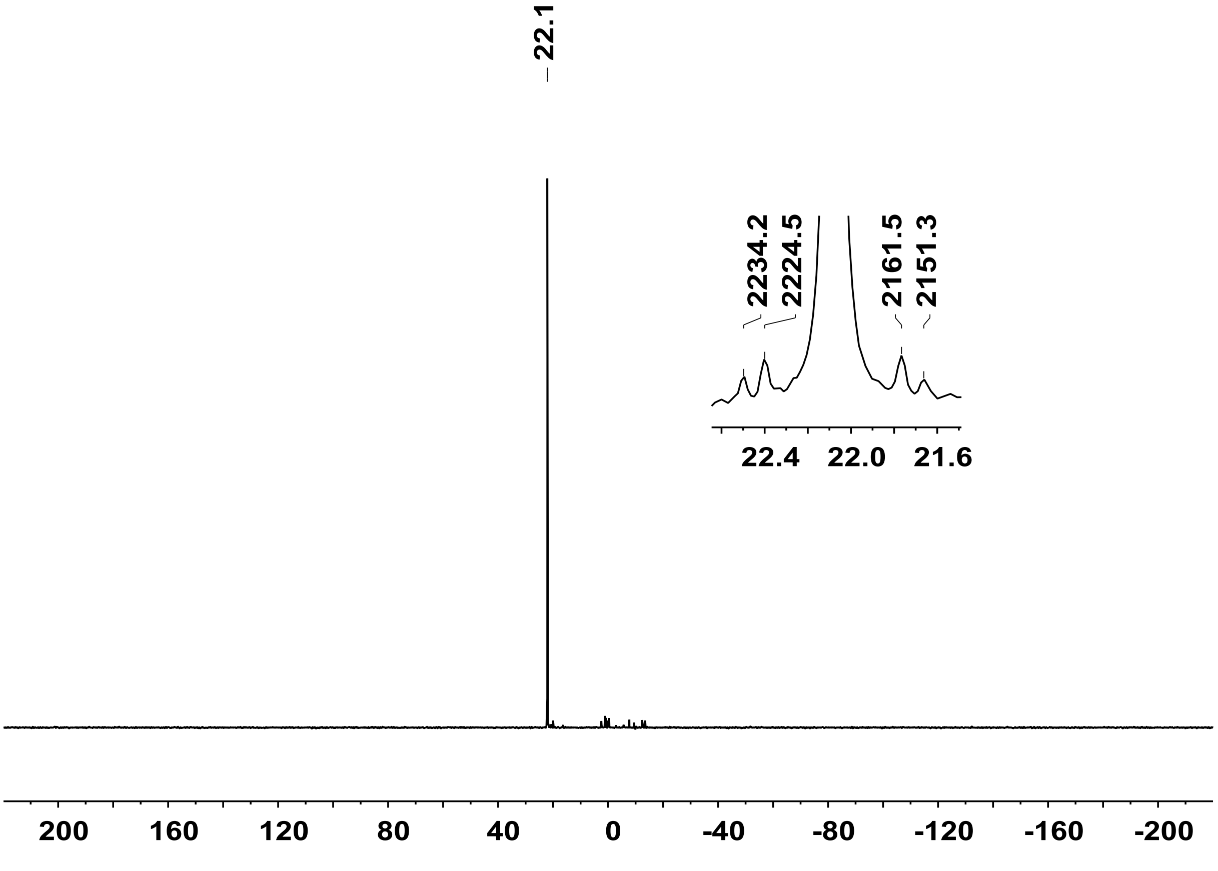

Figure S60 – $^{29}Si\{^{1}H\}$ INEPT NMR spectrum (99.31 MHz, 305.0 K, C_6D_6) of nitrilium borate **16a**[$B(C_6F_5)_4$].

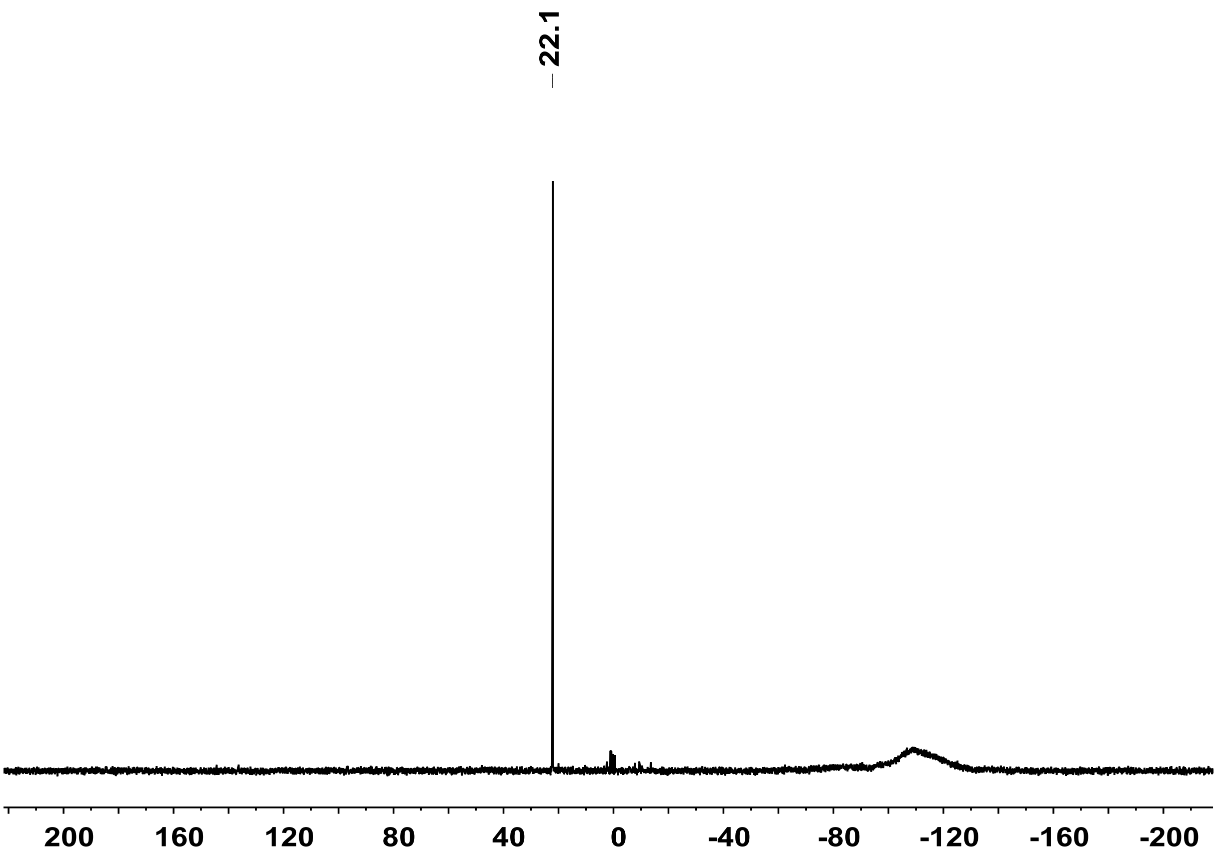

Figure S61 – $^{29}Si\{^1H\}$ NMR spectrum (99.31 MHz, 305.0 K, C_6D_6) of nitrilium borate **16a**[B(C_6F_5)$_4$].

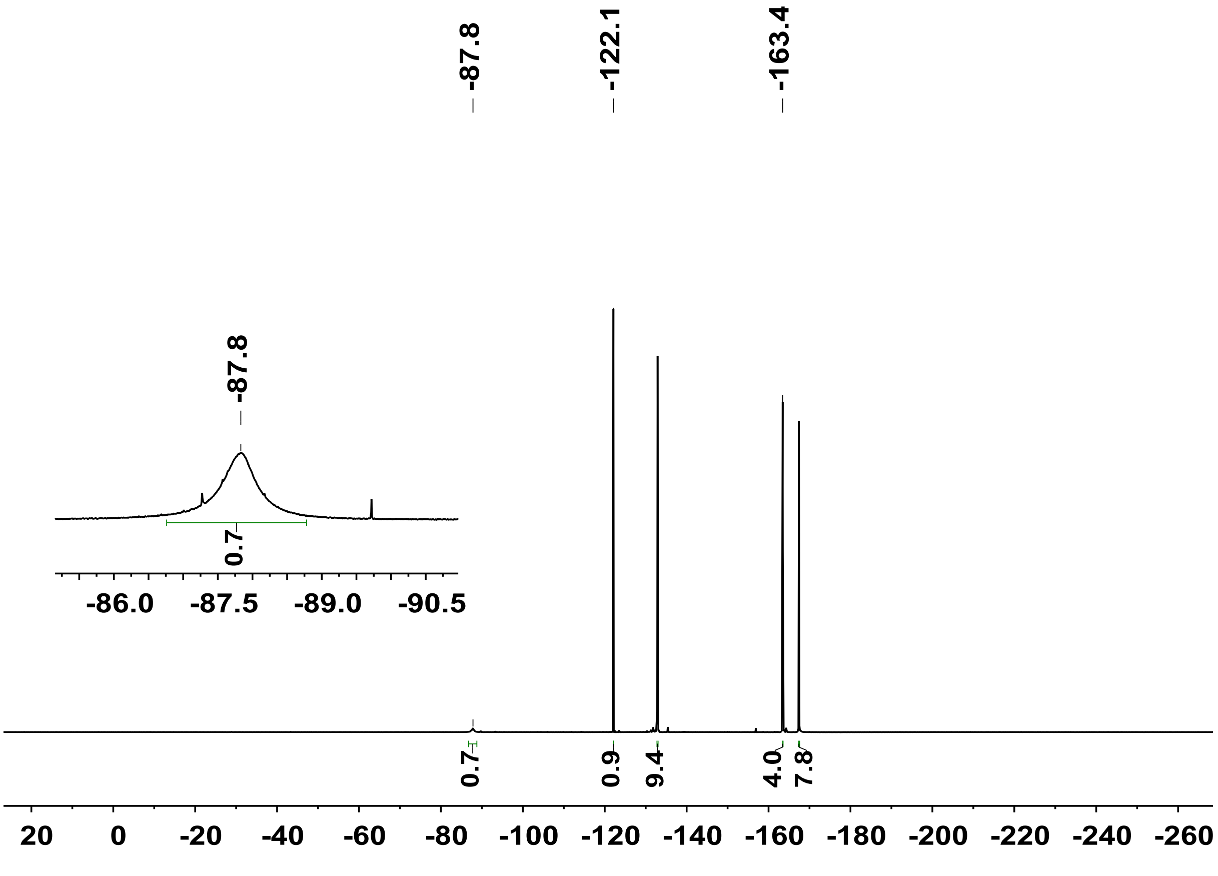

Figure S62 – $^{19}F\{^1H\}$ NMR spectrum (470.29 MHz, 305.0 K, C_6D_6) of nitrilium borate **16a**[B(C_6F_5)$_4$].

nitrilium ion ***16b***

The nitrilium ion **16b**[$B(C_6F_5)_4$] was synthesized according to general procedure **B** using 5-chloro-6-dimethylsilylacenaphthene **1b** (122 mg in a mixture with 13 % 5-chloroacenaphthene), trityl borate (1.0 equiv, 401 µmol, 370 mg) and 4-fluorobenzonitrile (0.9 equiv, 363 µmol, 44 mg).

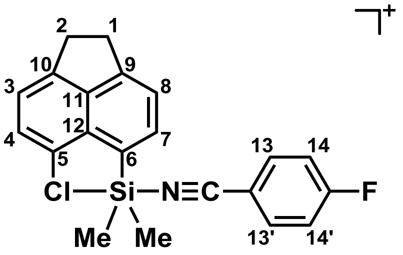

16b **[$B(C_6F_5)_4]^-$**

^{1}H NMR (500.45 MHz, 300.0 K, C_6D_6): δ = 0.85 (s, 6H, $Si(CH_3)_2$), 2.91-3.00 (m, 2H, CH_2), 3.06-3.08 (m, 2H, CH_2), 6.65-6.70 (m, 3H, H-14, H-14'), 6.97 (d, 1H, $^3J_{H,H}$ = 7.5 Hz, H-3), 7.10-7.13 (m, 4H, H-13, H-13', H-8), 7.40 (d, 1H, $^3J_{H,H}$ = 7.5 Hz, H-4), 7.75 (d, 1H, $^3J_{H,H}$ = 7.1 Hz, H-7). **^{13}C{^{1}H} NMR** (125.85 MHz, 300.0 K, C_6D_6): δ = 2.6 ($Si(CH_3)_2$), 29.7 (CH_2), 30.7 (CH_2), 97.4 (d, $^4J_{C,F}$ = 3.2 Hz, C, C-CN), 117.8 (C, C-6), 118.6 (d, $^2J_{C,F}$ = 23.6 Hz, CH, C-14, C-14'), 120.1, 121.7 (CH, C-3), 123.7, 125.0 (brs, C, $[B(C_6F_5)_4]^-$), 125,1 (C, C-5), 130.0 (CH, C-4), 132.9 (C, C-12), 137.0 (dm, $^1J_{C,F}$ = 242.0 Hz, CF, $[B(C_6F_5)_4]^-$), 138.2 (d, $^3J_{C,F}$ = 11.5 Hz, CH, C-13, C-13'), 138.9 (dm, $^1J_{C,F}$ = 241.7 Hz, CF, $[B(C_6F_5)_4]^-$), 139.2 (CH, C-7), 141.2 (C, C-11), 148.1 (C, C-10), 149.0 (dm, $^1J_{C,F}$ = 243.0 Hz, CF, $[B(C_6F_5)_4]^-$), 154.4 (C, C-9), 169.2 (d, $^1J_{C,F}$ = 269.9 Hz, CF, F-Ph). **^{19}F NMR** (470.84 MHz, 300.0 K, C_6D_6): δ = -167.6-(-167.3) (m, 8 F, *m*-F, $[B(C_6F_5)_4]^-$), -163.4 (t, $^3J_{F,F}$ = 20.6 Hz, 4 F, *o*-F, $[B(C_6F_5)_4]^-$), -133.5-(-132.7) (m, 8 F, *o*-F, $[B(C_6F_5)_4]^-$), -87.9-(-87.8) (m, 1 F, CF, F-Ph). **^{29}Si{^{1}H} INEPT NMR** (99.42 MHz, 300.0 K, C_6D_6): δ = 20.9 ($^1J_{Si,C}$ = 85.1 Hz, $^1J_{Si,C}$ = 65.2 Hz). **^{29}Si{^{1}H} NMR** (99.42 MHz, 300.0 K, C_6D_6): δ = 20.9. **^{11}B{^{1}H} NMR** (160.56 MHz, 300.0 K, C_6D_6): δ = -16.1.

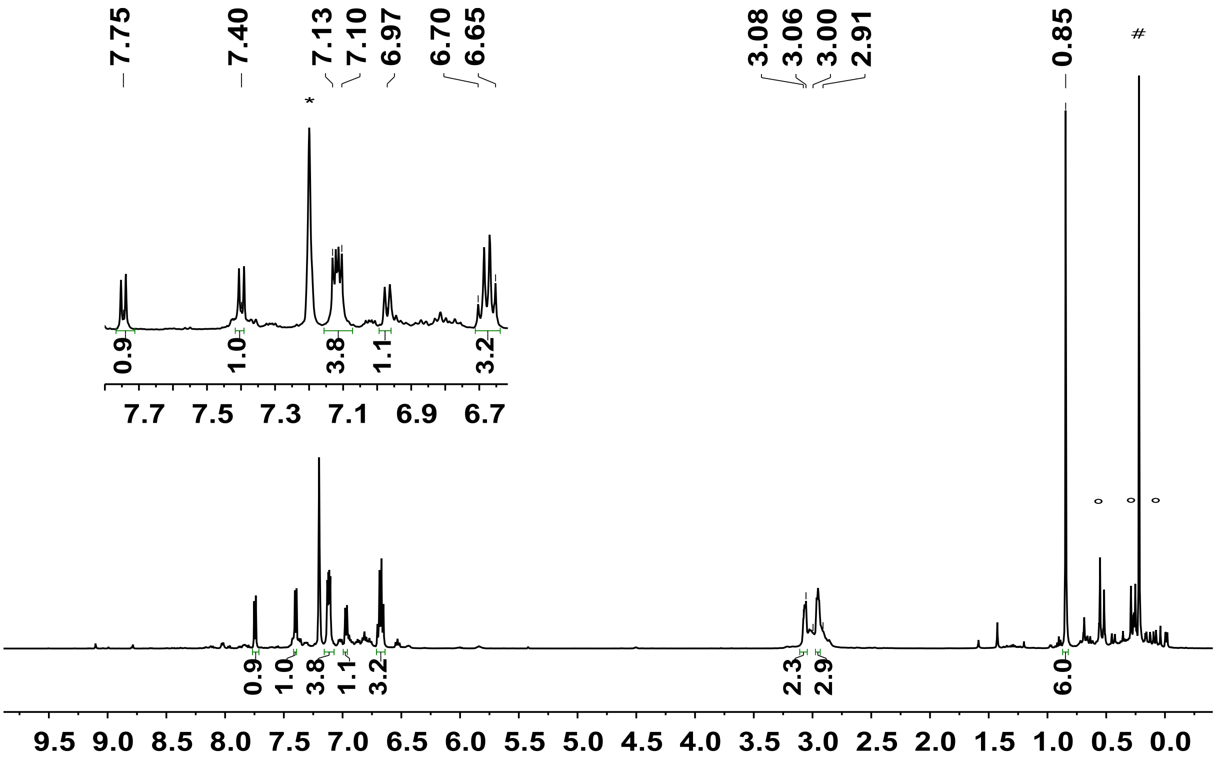

Figure S63 – ^{1}H NMR spectrum (500.45 MHz, 300.0 K, C_6D_6) of nitrilium borate **16b**[B(C_6F_5)$_4$] after washing with *n*-pentane (* C_6D_5H, # impurities possibly due to side reactions, ° impurities possibly due to decomposition).

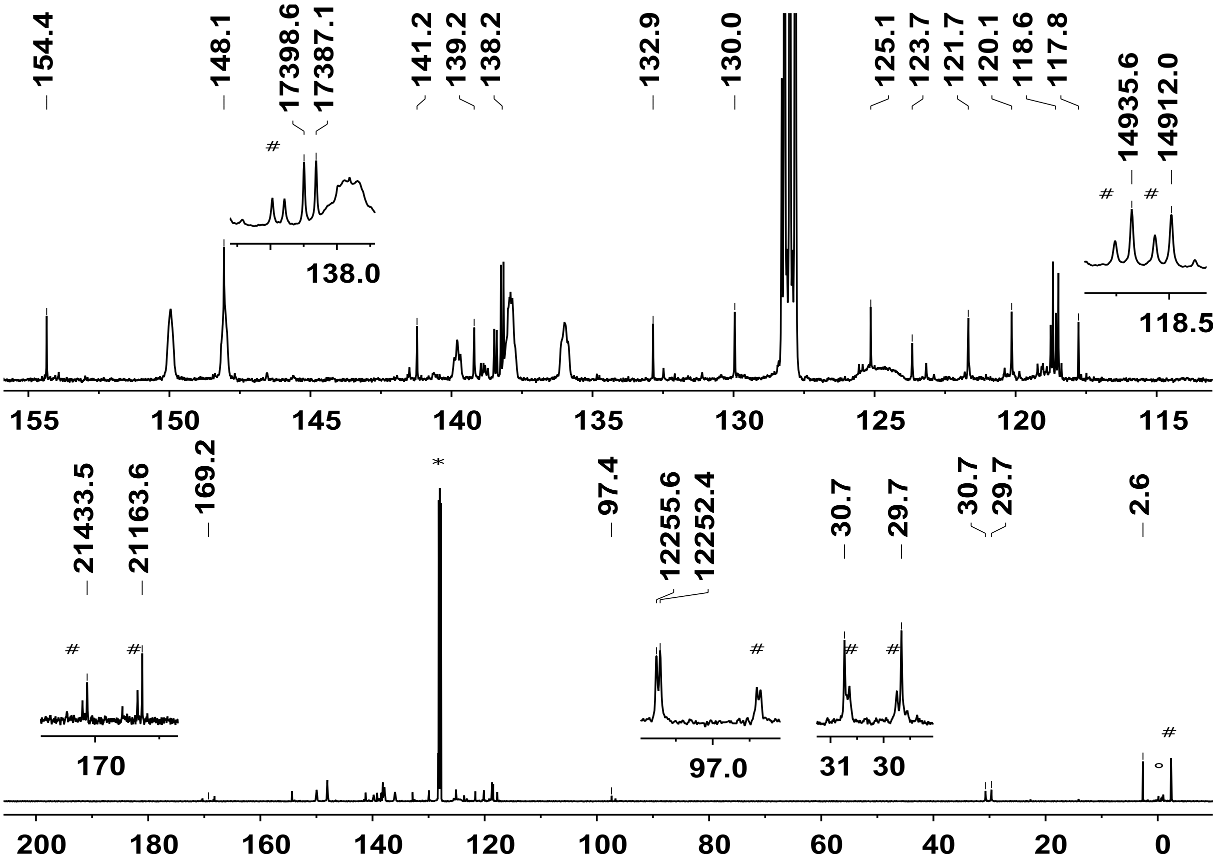

Figure S64 – $^{13}C\{^{1}H\}$ NMR spectrum (125.85 MHz, 300.0 K, C_6D_6) of nitrilium borate **16b**[B(C_6F_5)$_4$] after washing with *n*-pentane (* C_6D_6, # impurities possibly due to side reactions, ° impurities possibly due to decomposition).

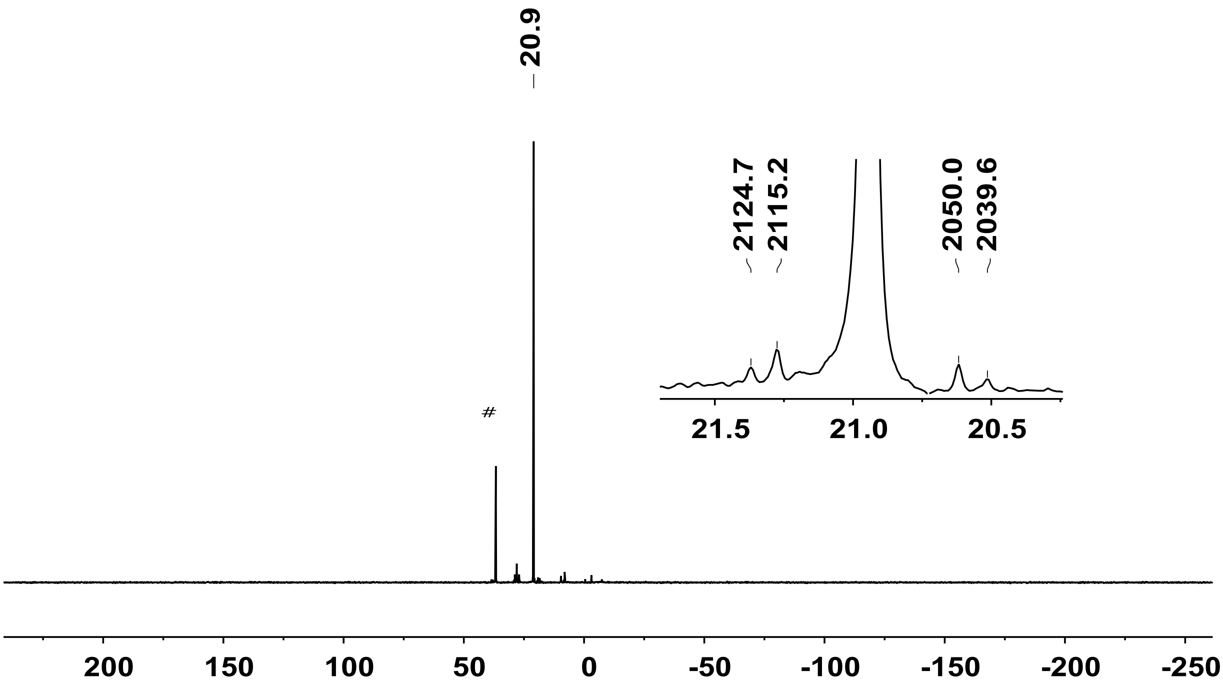

Figure S65 – $^{29}Si\{^{1}H\}$ INEPT NMR spectrum (99.42 MHz, 300.0 K, C_6D_6) of nitrilium borate **16b**[B(C_6F_5)$_4$] before washing with *n*-pentane (lb = 2.0, gb = 0.9) ($^{\#}$ impurities possibly due to side reactions).

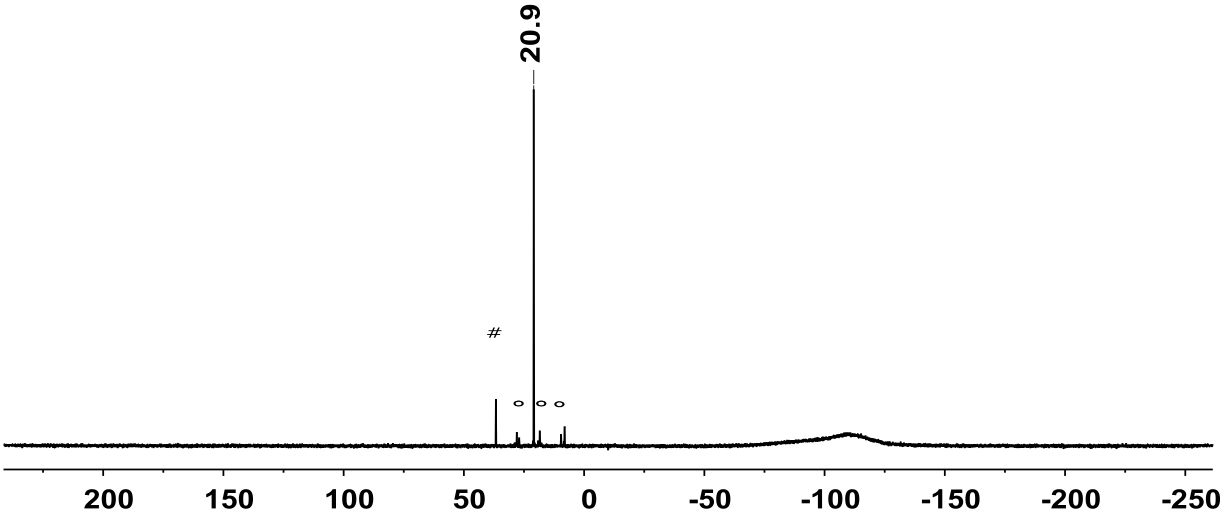

Figure S66 – $^{29}Si\{^{1}H\}$ NMR spectrum (99.42 MHz, 300.0 K, C_6D_6) of nitrilium borate **16b**[B(C_6F_5)$_4$] before washing with *n*-pentane ($^{\#}$ impurities possibly due to side reactions, ° impurities possibly due to decomposition).

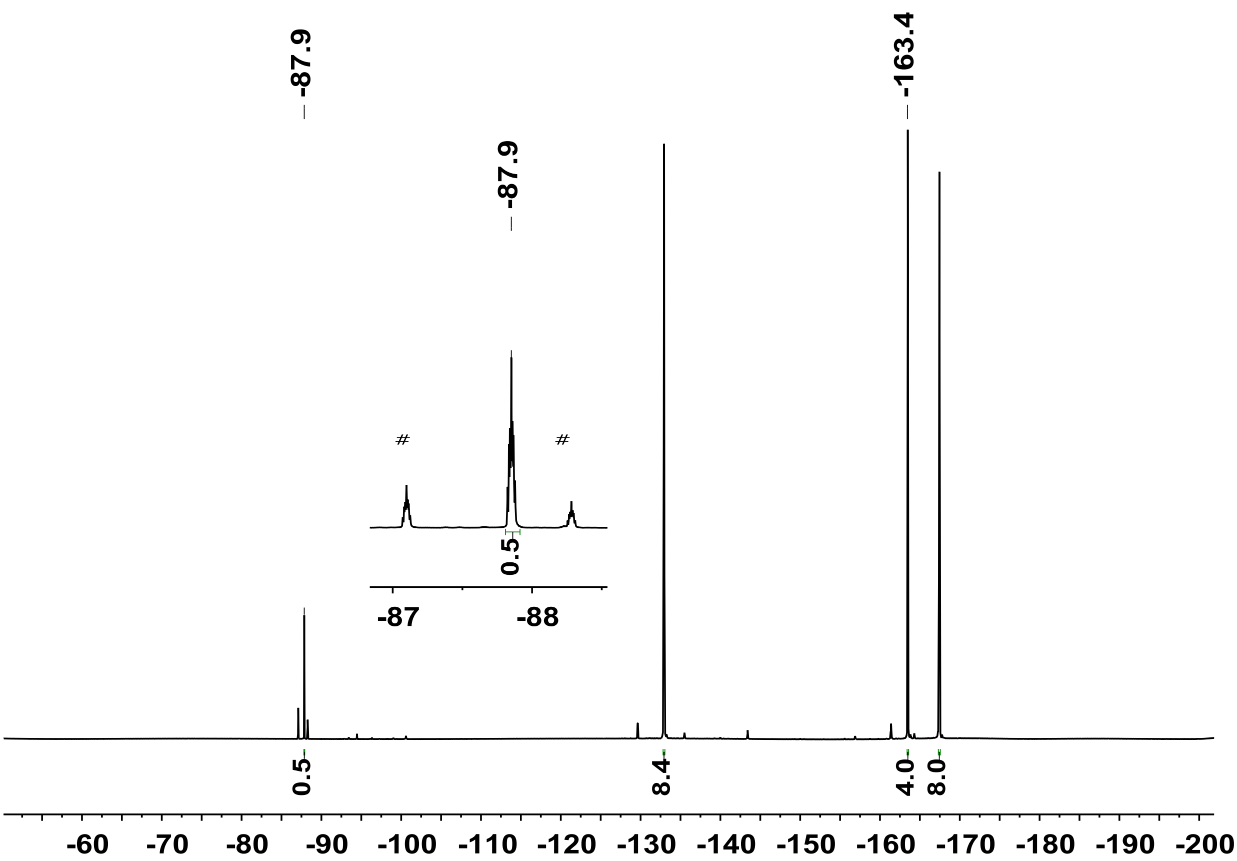

Figure S67 – ^{19}F NMR spectrum (470.84 MHz, 300.0 K, C_6D_6) of nitrilium borate **16b**[B(C_6F_5)$_4$] before washing with *n*-pentane ($^{\#}$ impurities possibly due to side reactions).

*nitrilium ion **16c***

The nitrilium ion **16c**[B(C_6F_5)$_4$] was synthesized according to general procedure **C** in toluene-d_8 using 5-bromo-6-dimethylsilylacenaphthene **1c** (1.1 equiv, 477 µmol, 138 mg), trityl borate (1.0 equiv, 434 µmol, 400 mg) and 4-fluorobenzonitrile (1.0 equiv, 434 µmol, 43 mg). After the addition of 4-fluorobenzonitrile, the reaction mixture was washed with a small amount of toluene-d_8 and analyzed by NMR spectroscopy, before the solvent was removed under reduced pressure. The residue was dissolved in benzene-d_6 and analyzed by NMR spectroscopy.

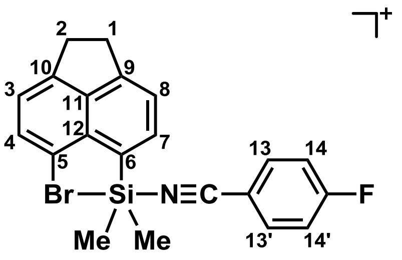

16c [B(C_6F_5)$_4$]$^-$

^{1}H NMR (499.87 MHz, 305.0 K, C_6D_6): δ = 0.93 (s, 6H, $Si(CH_3)_2$), 2.92-2.94 (m, 2H, CH_2), 3.04-3.07 (m, 2H, CH_2), 6.69-6.72 (m, 3H, H-14, H-14'), 6.90 (d, 1H, $^3J_{H,H}$ = 7.5 Hz, H-3), 7.17-7.22 (m, 4H, H-13, H-13', H-8, overlapping with benzene-d$_6$), 7.55 (d, 1H, $^3J_{H,H}$ = 7.5 Hz, H-4), 7.79 (d, 1H, $^3J_{H,H}$ = 7.2 Hz, H-7). **^{13}C{^{1}H} NMR** (125.71 MHz, 305.0 K, C_6D_6): δ = 4.4 ($Si(CH_3)_2$, $^1J_{C,Si}$ = 65.8 Hz), 29.7 (CH_2, C-2), 30.6 (CH_2, C-1), 98.5 (s, C, C-CN), 115.4 (C, C-5), 118.5 (d, $^2J_{C,F}$ = 23.5 Hz, CH, C-14, C-14'), 119.6 (C, C-6), 120.1 (CH, C-8), 122.3 (CH, C-3), 122.7 (s, C, CN), 124.9 (brs, C, $[B(C_6F_5)_4]^-$),133.6 (CH, C-4), 134.9 (C, C-12), 137.0 (dm, $^1J_{C,F}$ = 239.4 Hz, CF, $[B(C_6F_5)_4]^-$), 137.8 (d, $^3J_{C,F}$ = 11.1 Hz, CH, C-13, C-13'), 138.9 (dm, $^1J_{C,F}$ = 239.3 Hz, CF, $[B(C_6F_5)_4]^-$), 139.4 (CH, C-7), 141.6 (C, C-11), 148.7 (C, C-10), 149.0 (dm, $^1J_{C,F}$ = 242.4 Hz, CF, $[B(C_6F_5)_4]^-$), 154.4 (C, C-9), 168.9 (d, $^1J_{C,F}$ = 268.8 Hz, CF, F-Ph). **^{19}F{^{1}H} NMR** (470.30 MHz, 305.1 K, C_6D_6): δ = -167.7-(-167.0) (m, 8 F, *m*-F, $[B(C_6F_5)_4]^-$), -163.4 (t, $^3J_{F,F}$ = 19.7 Hz, 4 F, *p*-F, $[B(C_6F_5)_4]^-$), -133.1-(-132.4) (m, 8 F, *o*-F, $[B(C_6F_5)_4]^-$), -89.3 (s, 1 F, CF, F-Ph). **^{29}Si(^{1}H) INEPT NMR** (99.31 MHz, 305.0 K, C_6D_6): δ = 20.1 ($^1J_{Si,C}$ = 85.2 Hz, $^1J_{Si,C}$ = 65.7 Hz). **^{29}Si{^{1}H} NMR** (99.31 MHz, 305.1 K, C_6D_6,): δ = 20.0.

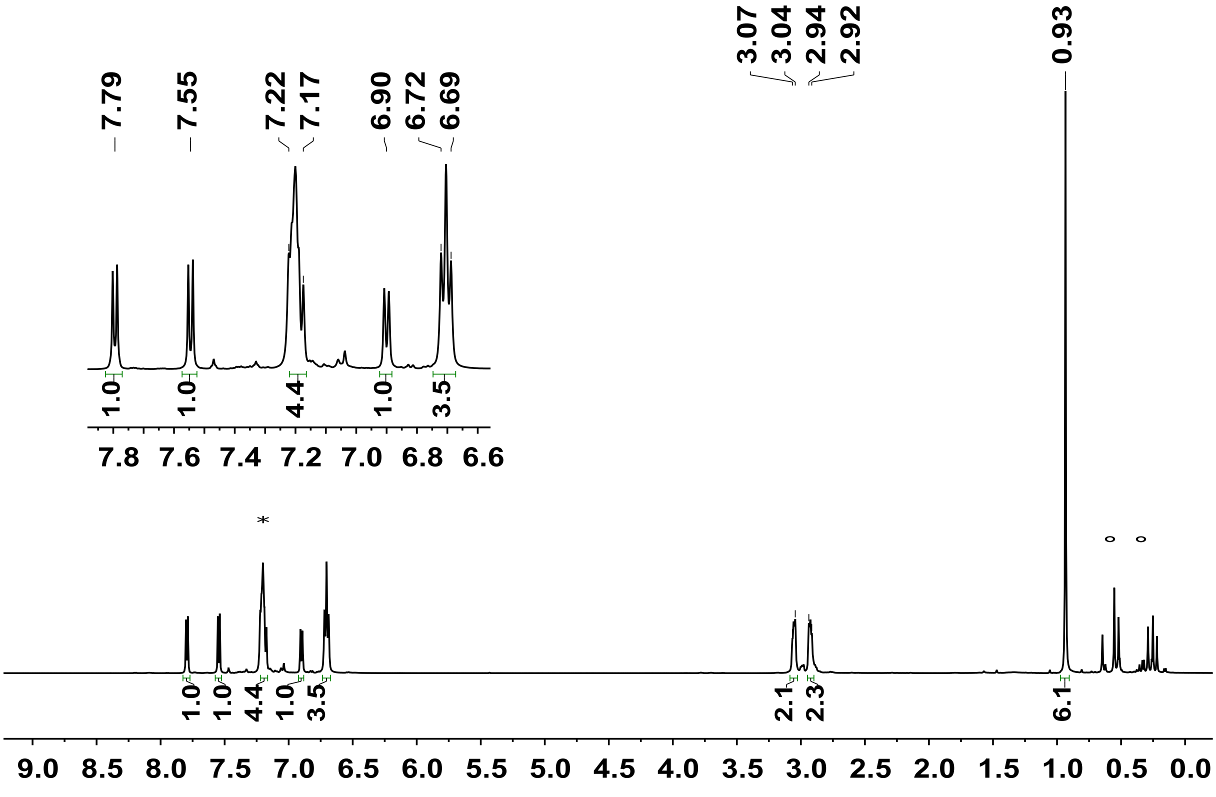

Figure S68 – ^{1}H NMR spectrum (499.87 MHz, 305.0 K, C_6D_6) of nitrilium borate **16c**$[B(C_6F_5)_4]$ (* C_6D_5H, ° impurities possibly due to decomposition).

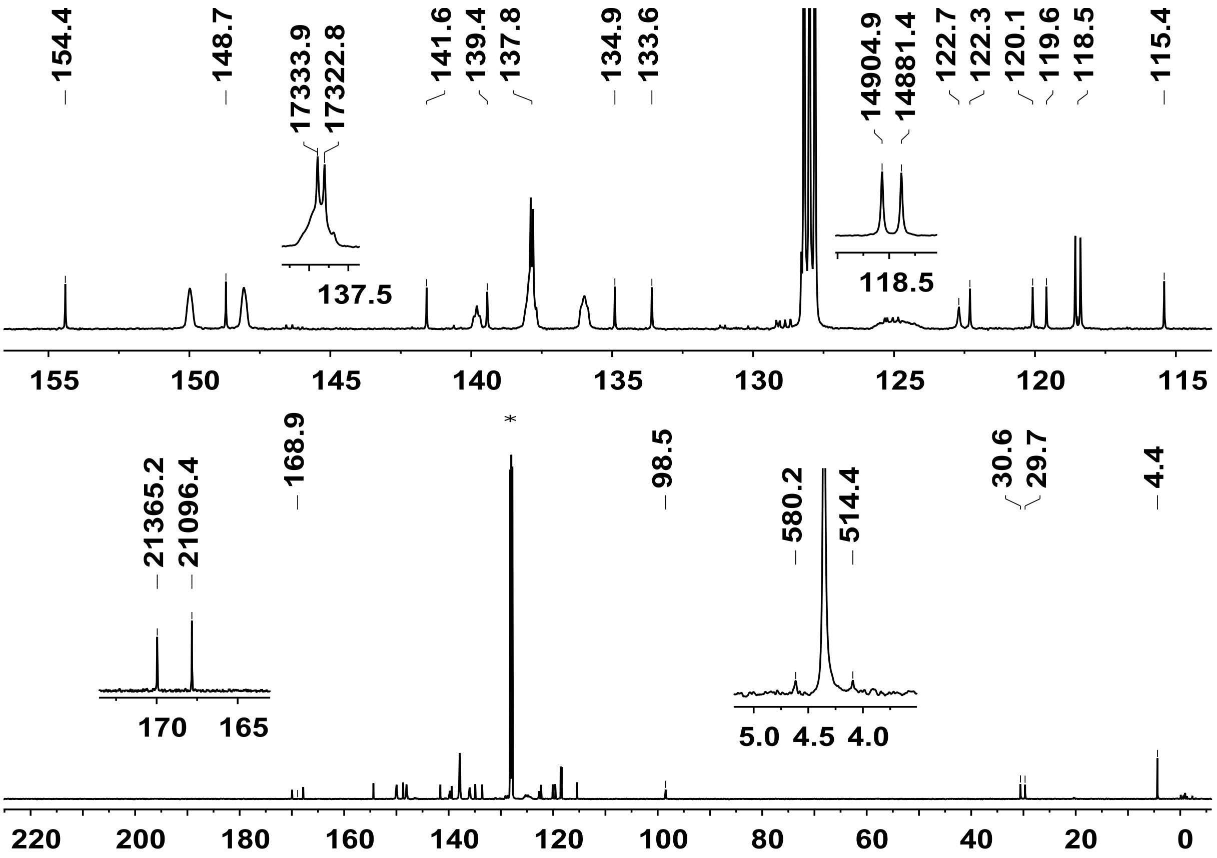

Figure S69 – $^{13}C\{^{1}H\}$ NMR spectrum (125.71 MHz, 305.0 K, C_6D_6) of nitrilium borate **16c**[$B(C_6F_5)_4$] (* C_6D_6).

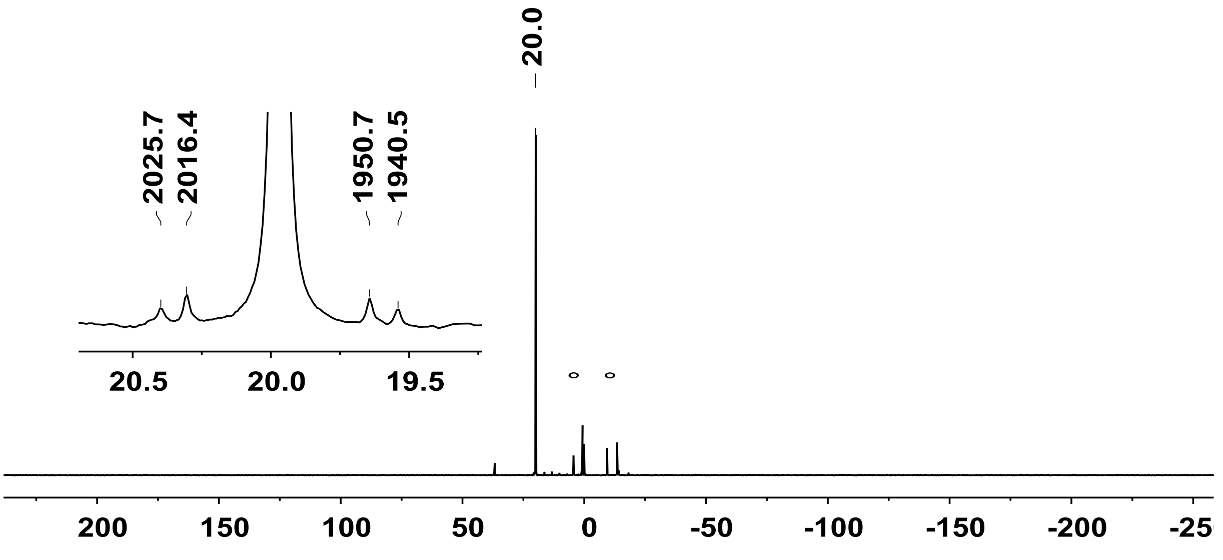

Figure S70 – $^{29}Si\{^{1}H\}$ INEPT NMR spectrum (99.31 MHz, 305.0 K, C_6D_6) of nitrilium borate **16c**[$B(C_6F_5)_4$] (° impurities possibly due to decomposition).

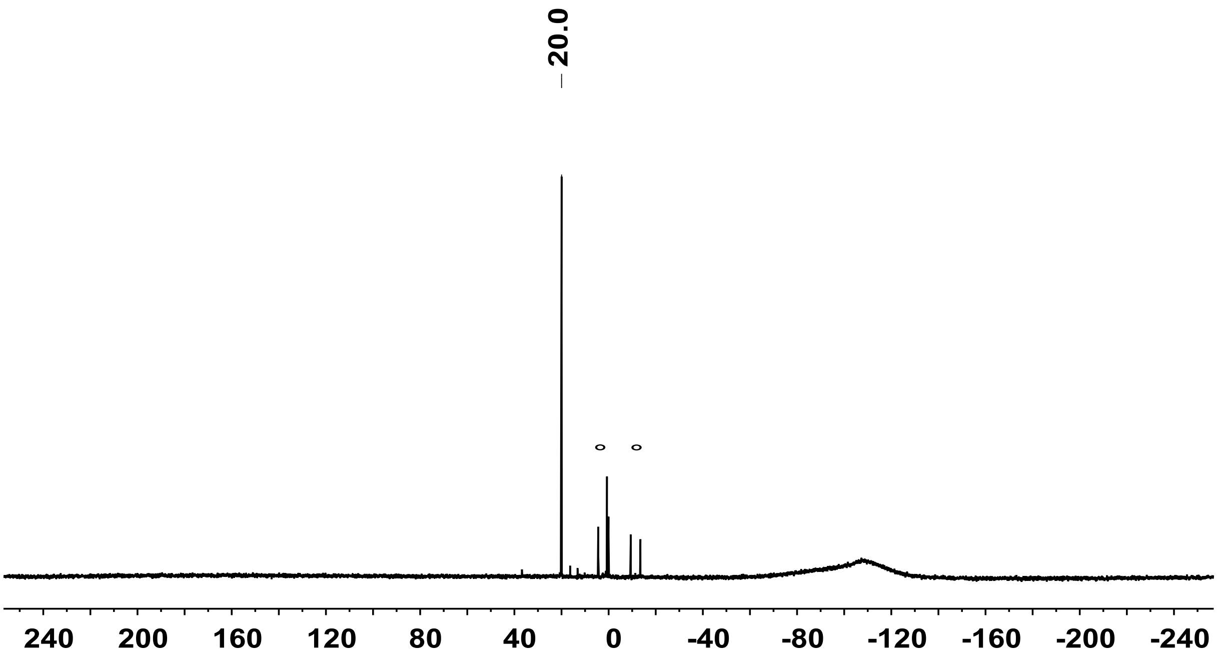

Figure S71 – $^{29}Si\{^1H\}$ NMR spectrum (99.31 MHz, 305.1 K, C_6D_6) of nitrilium borate **16c**[$B(C_6F_5)_4$ (° impurities possibly due to decomposition).

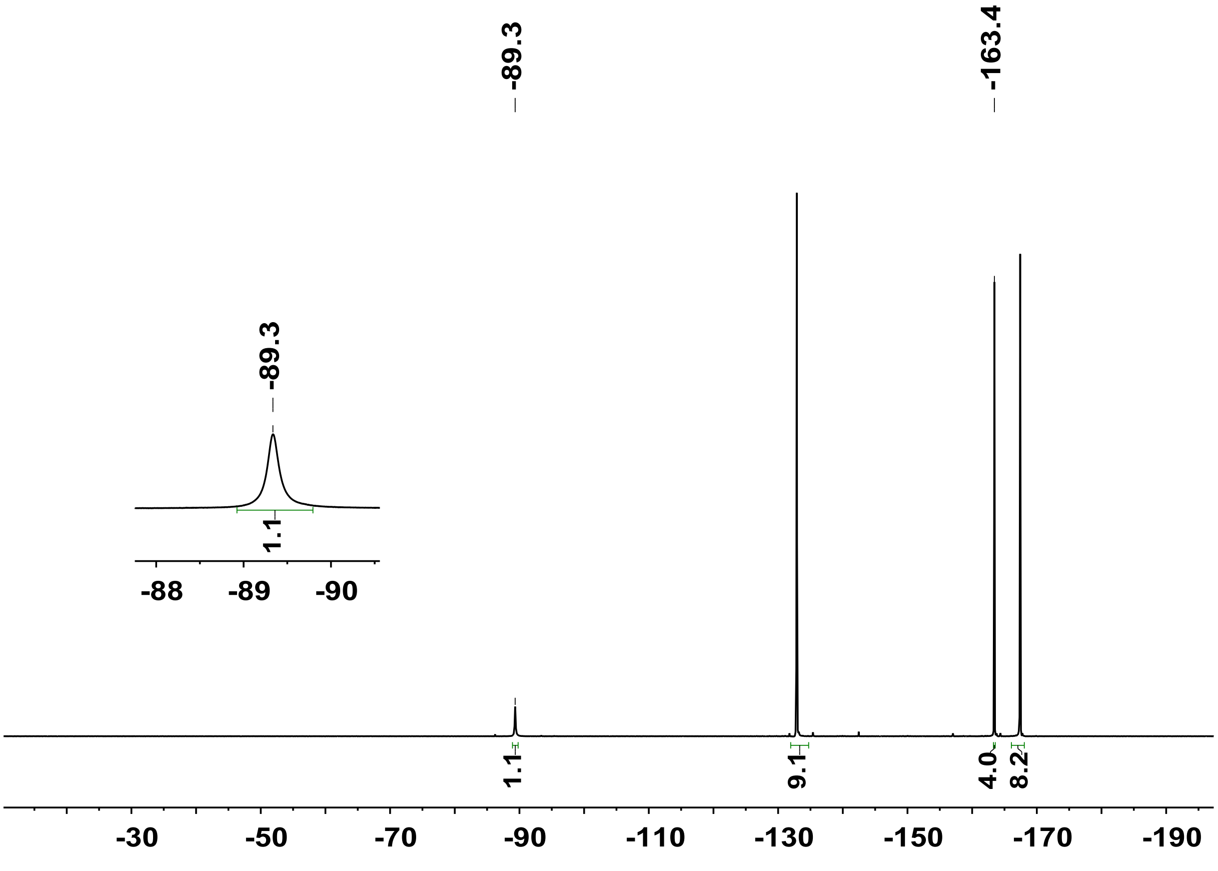

Figure S72 – $^{19}F\{^1H\}$ NMR spectrum (470.30 MHz, 305.1 K, C_6D_6) of nitrilium borate **16c**[$B(C_6F_5)_4$].

nitrilium ion ***16d***

The nitrilium ion **16d**[B(C_6F_5)$_4$] was synthesized according to general procedure **C** using a reaction mixture containing 5-iodo-6-dimethylsilylacenaphthene **1d** as main product (with 5-iodoacenaphthene and a small amount of 5-dimethylsilylacenaphthene [S3]) (162 mg), trityl borate (1.0 equiv, 384 µmol, 355 mg) and 4-fluorobenzonitrile (0.8 equiv, 315 µmol, 38 mg). The reaction mixture containing 5-iodo-6-dimethylsilylacenaphthene **1d** was used in excess compared to the amount of the trityl borate. The NMR spectroscopic analysis revealed broad signals in ^{1}H and ^{29}Si{^{1}H} NMR spectra. Thereafter, the NMR sample was transferred into a Schlenk tube, 4-fluorobenzonitrile (124 µmol, 15 mg) was added, the reaction mixture was stirred for 15 min and analyzed by NMR spectroscopy again.

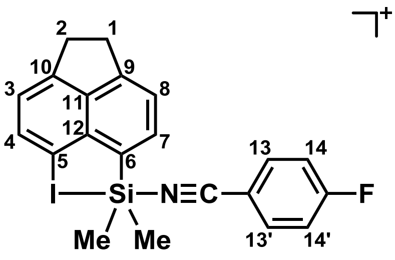

16d **[B(C_6F_5)$_4$]$^-$**

^{1}H NMR (499.87 MHz, 295.8.0 K, C_6D_6): δ = 1.08 (s, 6H, Si(CH_3)$_2$), 2.86-2.90 (m, 2H, CH_2, H-2), 3.00-3.03 (m, 2H, CH_2, H-1), 6.61-6.65 (m, 3H, H-14, H-14'), 6.76 (d, 1H, $^3J_{H,H}$ = 7.4 Hz, H-3), 7.13-7.16 (m, 3H, H-13, H-13', H-8), 7.79 (d, 1H, $^3J_{H,H}$ = 7.4 Hz, H-4), 7.82 (d, 1H, $^3J_{H,H}$ = 7.2 Hz, H-7). **^{13}C{^{1}H} NMR** (125.71 MHz, 298.4.0 K, C_6D_6): δ = 6.9 (Si(<u>CH</u>$_3$)$_2$, $^1J_{C,Si}$ = 66.4 Hz), 29.7 (CH_2, C-2), 30.2 (CH_2, C-1), 90.0 (C, C-5), 99.3 (s, <u>C</u>-CN), 118.2 (d, $^2J_{C,F}$ = 23.5 Hz, CH, C-14, C-14'), 119.8 (CH, C-8), 122.3 (C), 122.5 (C, C-6), 123.1 (CH, C-3), 124.9 (brs, C, [B(C_6F_5)$_4$]$^-$), 136.9 (dm, $^1J_{C,F}$ = 241.3 Hz, CF, [B(C_6F_5)$_4$]$^-$), 137.4 (d, $^3J_{C,F}$ = 11.0 Hz, CH, C-13, C-13'), 138.8 (dm, $^1J_{C,F}$ = 241.3 Hz, CF, [B(C_6F_5)$_4$]$^-$), 139.0 (C), 139.8 (CH), 141.5 (CH), 141.6 (C, C-11), 149.0 (dm, $^1J_{C,F}$ = 241.3 Hz, CF, [B(C_6F_5)$_4$]$^-$), 149.5 (C, C-10), 154.6 (C, C-9), 168.5 (d, $^1J_{C,F}$ = 267.6 Hz, CF, F-Ph). **^{19}F{^{1}H} NMR** (470.30 MHz, 296.0 K, C_6D_6): δ = -167.8-(-167.0) (m, 8F, *m*-F, [B(C_6F_5)$_4$]), -163.4 (t, $^3J_{F,F}$ = 20.4 Hz, 4F, *p*-F, [B(C_6F_5)$_4$]$^-$), -133.9-(-132.1) (m, 8F, *o*-F, [B(C_6F_5)$_4$]$^-$), -90.7 (brs, 1F, CF, F-Ph). **^{29}Si(^{1}H) INEPT NMR** (99.31 MHz, 305.0 K, C_6D_6): δ = 18.9 ($^1J_{Si,C}$ = 85.0 Hz, $^1J_{Si,C}$ = 66.1 Hz).

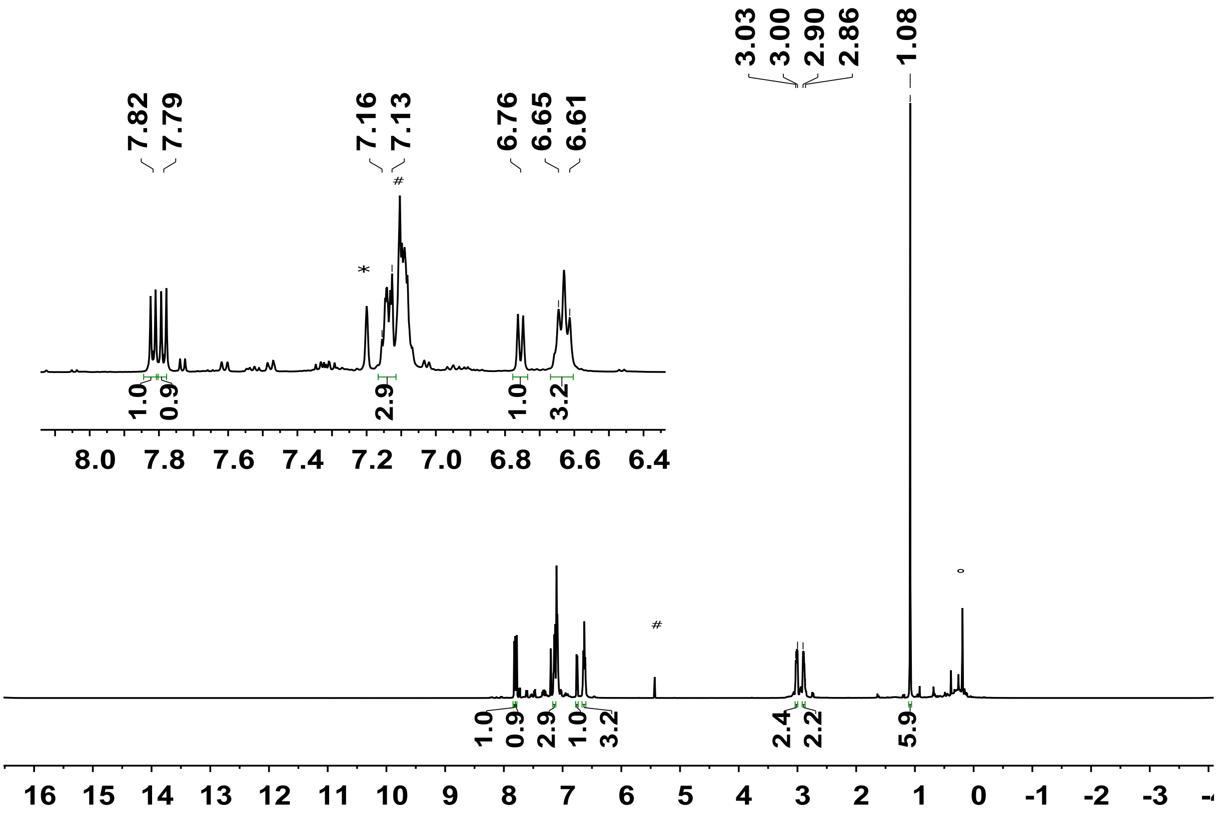

Figure S73 – ^{1}H NMR spectrum (499.87 MHz, 295.8 K, C_6D_6) of nitrilium borate **16d**[$B(C_6F_5)_4$] (* C_6D_5H, # triphenylmethane, ° impurities possibly due to side reactions).

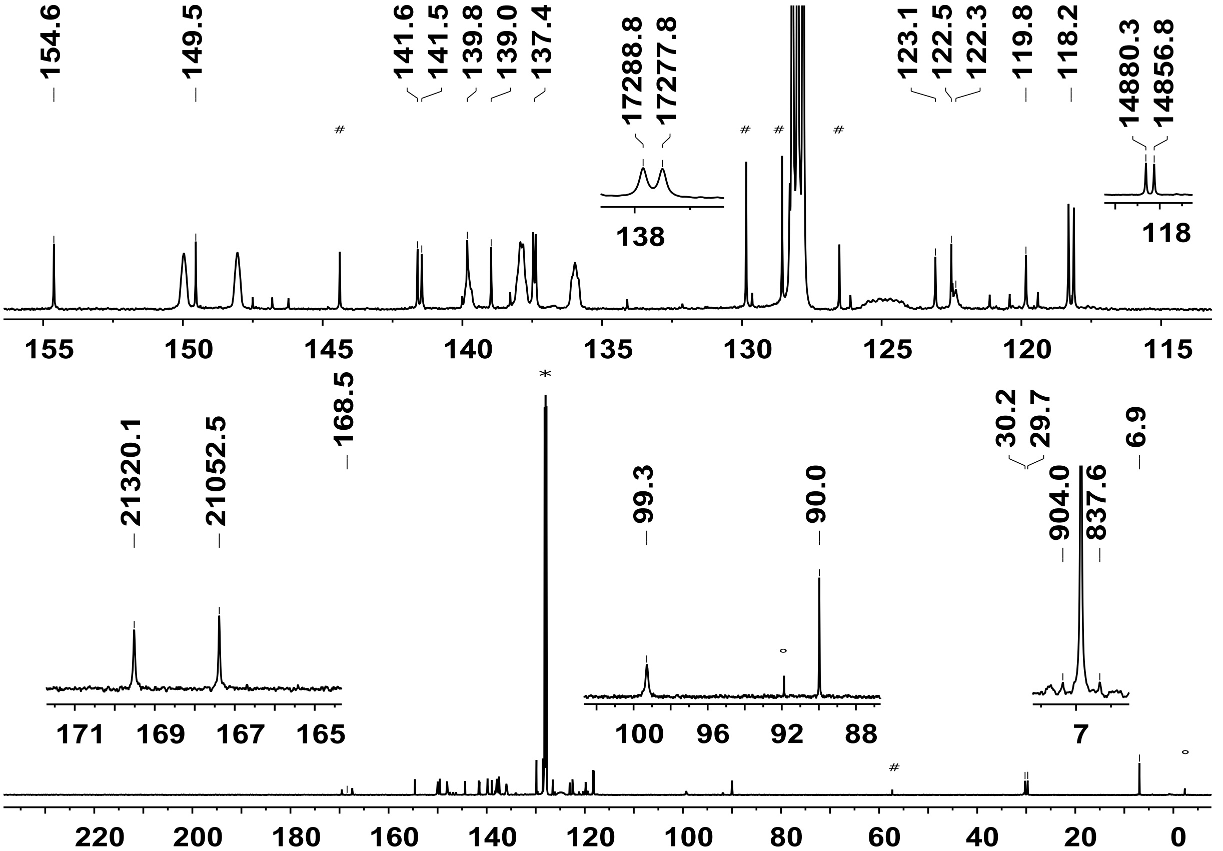

Figure S74 – $^{13}C\{^{1}H\}$ NMR spectrum (125.77 MHz, 298.4 K, C_6D_6) of nitrilium borate **16d**[$B(C_6F_5)_4$] (* C_6D_6, # triphenylmethane, ° impurities possibly due to side reactions).

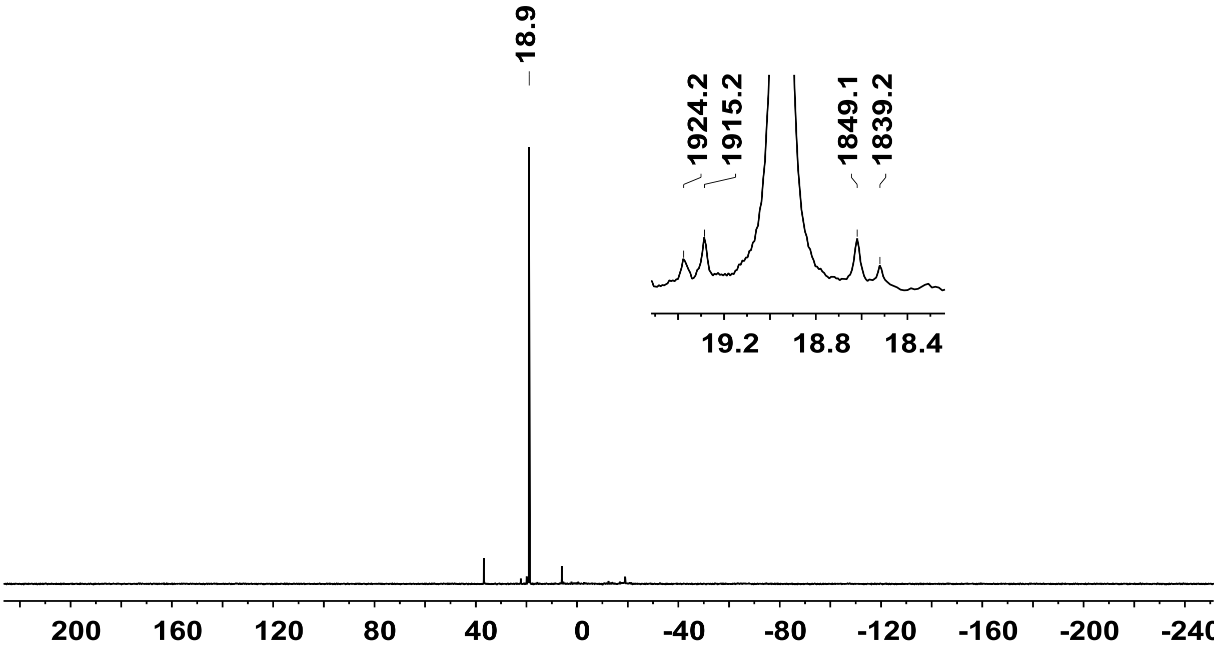

Figure S75 – $^{29}Si\{^1H\}$ INEPT NMR spectrum (99.31 MHz, 305.0 K, C_6D_6) of nitrilium borate **16d**[$B(C_6F_5)_4$].

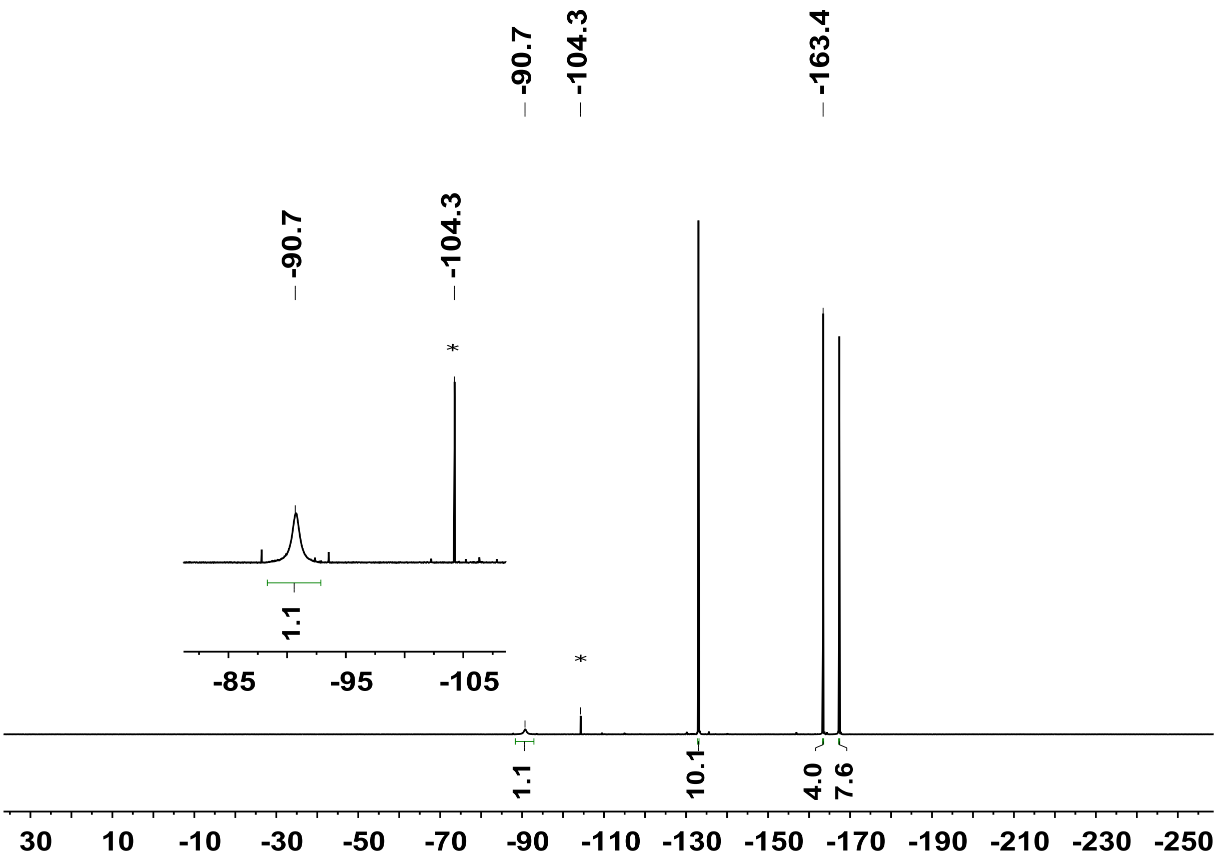

Figure S76 – $^{19}F\{^1H\}$ NMR spectrum (470.30 MHz, 296.0 K, C_6D_6) of nitrilium borate **16d**[$B(C_6F_5)_4$] (* 4-fluorobenzonitrile).

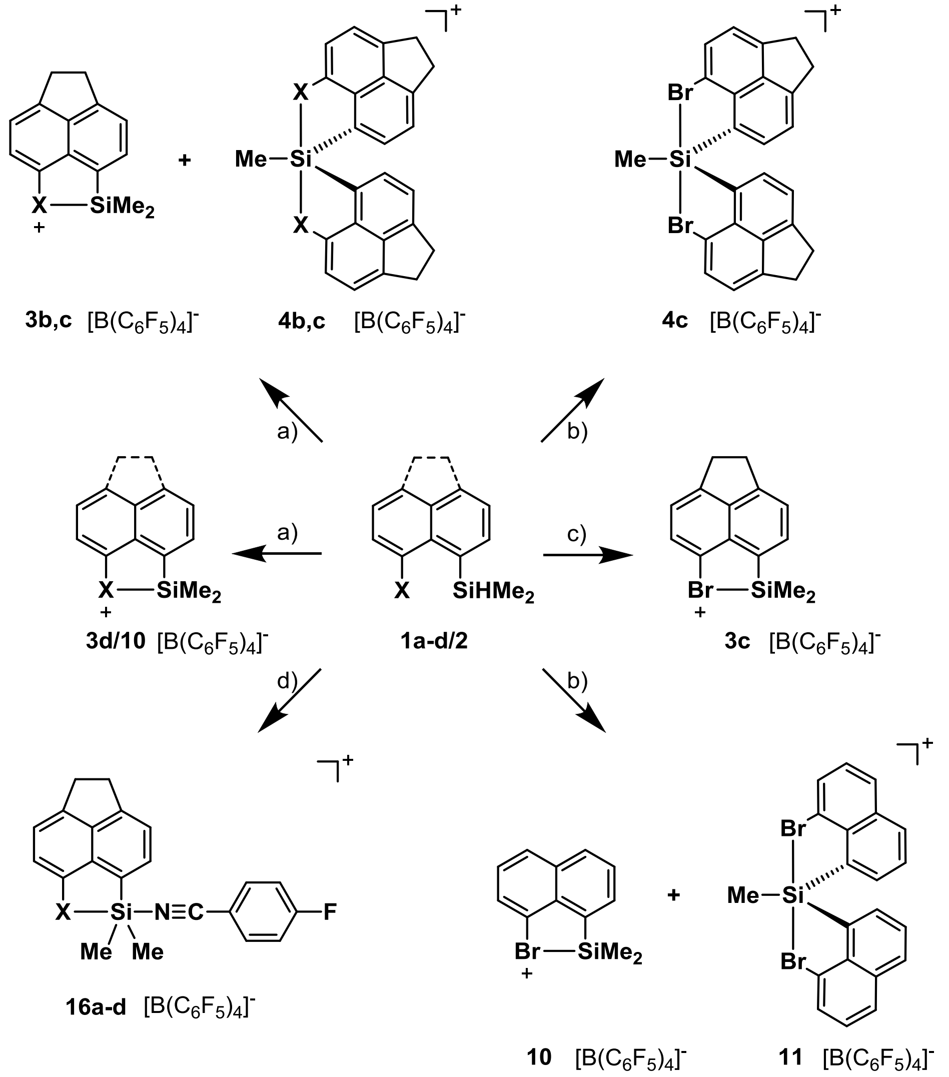

Scheme S2 - Synthesis of silyl borates **3**, **4**, **10**, **11** and **16** (**a**: X = F; **b**: X = Cl; **c**: X = Br; **d**: X = I). a) 1 equiv $[Ph_3C][B(C_6F_5)_4]$, $[Ph_3C][B(C_6F_5)_4]$ in 0.5 mL solvent was added to the silane in 0.5 mL solvent; b) 1/3 equiv $[Ph_3C][B(C_6F_5)_4]$; c) 1 equiv $[Ph_3C][B(C_6F_5)_4]$, silane in 5 mL solvent was added to $[Ph_3C][B(C_6F_5)_4]$ in 0.5 mL solvent; d) 1 equiv $[Ph_3C][B(C_6F_5)_4]$ and 1 equiv 4-fluorobenzonitrile.

Table S1 – Selected NMR parameters of silanes **1**, **2**, silylborates **3**, **4**, **10**, **11** and nitrilum borates **16** (C_6D_6, r.t. if not stated otherwise).

compound	X	δ^{29}Si exp.	δ^{29}Si calc.[a]	δ^{1}H(SiH)	^{1}J(SiH) [Hz]	^{1}J(SiC$_{Me}$) [Hz]	^{1}J(SiC$_{Aryl}$) [Hz]	δ^{19}F
silane **1a**	F	-12.4		5.10	191	53	66	-119.4
silane **1b**	Cl	-12.7		5.37	195	53	67	
silane **1c**[S3]	Br	-14.5		5.56	197			
silane **1d**[S3]	I	-19.1		5.96	199			
silane **2**[S4]	Br	-13.3		5.44	199			
silylborate **3b**	Cl	117.0	117					
silylborate **4b**	Cl	79.8	79					
silylborate **3c**	Br	107.9	109			54	76	
silylborate **4c**	Br	82.6	85			62	82	
silylborate **3d**	I	89.5	101			54	72	
silylborate **10**	Br	94.6	98			53	72	
silylborate **11**	Br	62.9	61					
nitriluim borate **16a**	F	22.1				63	83	-122.1 (C_6-F)<br>-87.8 (C_{Ph}-F)
nitriluim borate **16b**	Cl	20.9				65	85	-87.9
nitriluim borate **16c**	Br	20.1				66	85	-89.3
nitriluim borate **16d**	I	18.9				66	85	-90.7

[a] M06-L/def2-TZVP//M06-2X/def2-TZVP

5 Data from X-ray diffraction analysis

Single crystal X-ray data were measured on a *Bruker AXS Apex II* diffractometer (graphite-monochromated Mo-Kα radiation with λ = 71.073 pm, Kappa 4 circle goniometer, *Bruker Apex II* detector) or on a *Bruker AXS D8 Venture* diffractometer (multilayer optics, Mo-Kα and Cu-Kα radiation with λ = 0.71073 Å and λ = 1.54178 Å respectively, Kappa 4-circle goniometer, *Photon III C14 CPAD* detector). An empirical absorption corrections using equivalent reflections was performed with the program SADABS [S7] or TWINABS [8]. The structure was solved with the program SHELXS [S9] and refined with SHELXL [S10] using the OLEX2 GUI [S11].

Pertinent data are summarized in Tables S2 - 5. CCDC-2023360, CCDC-2023357, CCDC-2023359 and CCDC-2023358, contain the supplementary crystallographic data for this paper. These data can be obtained free of charge from The Cambridge Crystallographic Data Centre. The Director, CCDC, 12 Union Road, Cambridge CB2 1EZ, UK [Fax: (internat.) +44-1223/336-033; E-mail: deposit@ccdc.cam.ac.uk].

1.6.1 Data from X-ray diffraction analysis of silane 1a

Colorless crystals of silane **1a** that were suitable for X-ray diffraction analysis were obtained by slow evaporation of *n*-pentane at -20 °C.

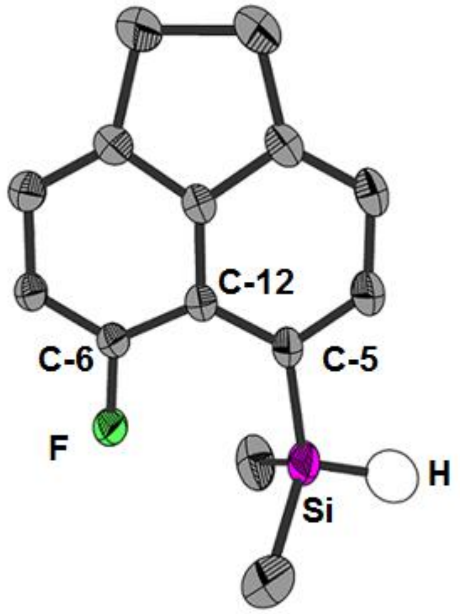

Figure S77 – Asymmetric unit of the crystal structure of silane **1a**. (Thermal ellipsoids are shown at the 50 % probability level. Hydrogen atoms are omitted for clarity; only the hydrogen atom attached to silicon is shown). There is disorder of the silane, shown is the structure with highest site occupancy (90:10). Pertinent bond lengths [pm] and bond angles [°]: F-C^6 136.22(16), Si-C^5 186.83(16), F/Si 300.87(10), Si-H 138.(3), F/H 438.68(26), F-C^6-C^{12} 117.89(12), Si-C^5-C^{12} 126.81(11), C^5-C^{12}-C^6 128.40(13), sum of bay angles ∑β 373.1.

Table S2 – Crystal data and structure refinement for silane **1a** (CCDC-2023360).

Identification code	amma13	
Empirical formula	C14 H15 F Si	
Formula weight	230.35	
Temperature	100(2) K	
Wavelength	0.71073 Å	
Crystal system	Monoclinic	
Space group	$P2_1/m$	
Unit cell dimensions	a = 8.6427(3) Å	α = 90°.
	b = 7.1034(2) Å	β = 105.5187(9)°.
	c = 10.4514(3) Å	γ = 90°.
Volume	618.25(3) $Å^3$	
Z	2	
Density (calculated)	1.237 Mg/m^3	
Absorption coefficient	0.172 mm^{-1}	
F(000)	244	
Crystal size	0.400 x 0.400 x 0.100 mm^3	
Theta range for data collection	2.022 to 33.727°	
Index ranges	-13<=h<=13, -11<=k<=11, -16<=l<=16	
Reflections collected	33968	
Independent reflections	2638 (R(int) = 0.0262)	
Observed reflections (I > 2(I))	2317	
Completeness to theta = 33.727°	100.0 %	
Absorption correction	Semi-empirical from equivalents	
Max. and min. transmission	1.0000 and 0.9543	
Refinement method	Full-matrix least-squares on F^2	
Data / restraints / parameters	2638 / 43 / 148	
Goodness-of-fit on F^2	1.174	
Final R indices (I>2sigma(I))	R1 = 0.0472, wR2 = 0.1370	
R indices (all data)	R1 = 0.0528, wR2 = 0.1425	
Extinction coefficient	n/a	
Largest diff. peak and hole	0.393 and -0.368 e.$Å^{-3}$	

1.6.2 Data from X-ray diffraction analysis of silane 1c

Colorless crystals of silane **1c** that were suitable for X-ray diffraction analysis were obtained by slow evaporation of *n*-pentane at -20 °C.

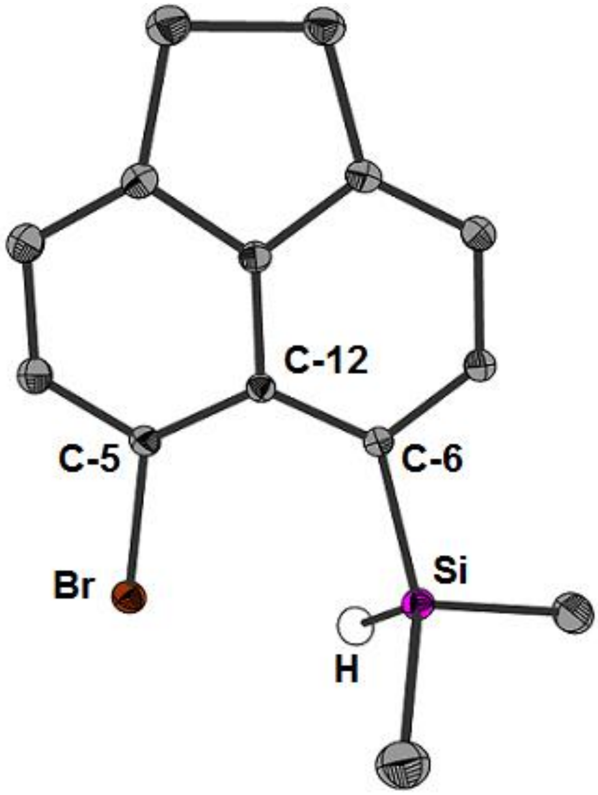

Figure S78 – Asymmetric unit of the crystal structure of silane **1c** (Thermal ellipsoids are shown at the 50 % probability level. Hydrogen atoms are omitted for clarity; only the hydrogen atom attached to silicon is shown.) Pertinent bond lengths [pm] and bond angles [°]: Br-C^5 190.28(6), Si-C^6 188.79(6), Si-H 140.0(12), Br-C^5-C^{12} 122.89(4), Si-C^6-C^{12} 128.69(4), C^5-C^{12}-C^6 130.14(5), sum of bay angles ∑β 381.7.

.

Table S3 – Crystal data and structure refinement for silane **1c** (CCDC-2023357).

Identification code	amma38	
Empirical formula	C14 H15 Br Si	
Formula weight	291.26	
Temperature	100(2) K	
Wavelength	0.71073 Å	
Crystal system	Monoclinic	
Space group	$P2_1/c$	
Unit cell dimensions	a = 9.7192(2) Å	α = 90°.
	b = 9.3072(2) Å	β = 102.0820(11)°.
	c = 14.1236(3) Å	γ = 90°.
Volume	1249.30(5) $Å^3$	
Z	4	
Density (calculated)	1.549 Mg/m^3	
Absorption coefficient	3.356 mm^{-1}	
F(000)	592	
Crystal size	0.240 x 0.240 x 0.060 mm^3	
Theta range for data collection	2.143 to 40.248°	
Index ranges	-17<=h<=17, -16<=k<=16, -25<=l<=25	
Reflections collected	117376	
Independent reflections	7853 (R(int) = 0.0281)	
Observed reflections (I > 2(I))	6930	
Completeness to theta = 40.248°	100.0 %	
Absorption correction	Numerical	
Max. and min. transmission	0.8110 and 0.4344	
Refinement method	Full-matrix least-squares on F^2	
Data / restraints / parameters	7853 / 0 / 151	
Goodness-of-fit on F^2	0.998	
Final R indices (I>2sigma(I))	R1 = 0.0183, wR2 = 0.0493	
R indices (all data)	R1 = 0.0238, wR2 = 0.0511	
Extinction coefficient	n/a	
Largest diff. peak and hole	0.669 and -0.220 $e.Å^{-3}$	

1.6.3 Data from X-ray diffraction analysis of bromonium borate 10[B(C_6F_5)$_4$]

Crystals from bromonium borate **10**[B(C_6F_5)$_4$] were obtained from its mixture with Li[B(C_6F_5)$_4$] in benzene at room temperature and were suitable for X-ray diffraction analysis. Li[B(C_6F_5)$_4$] came from minor impurities of the trityl borate.

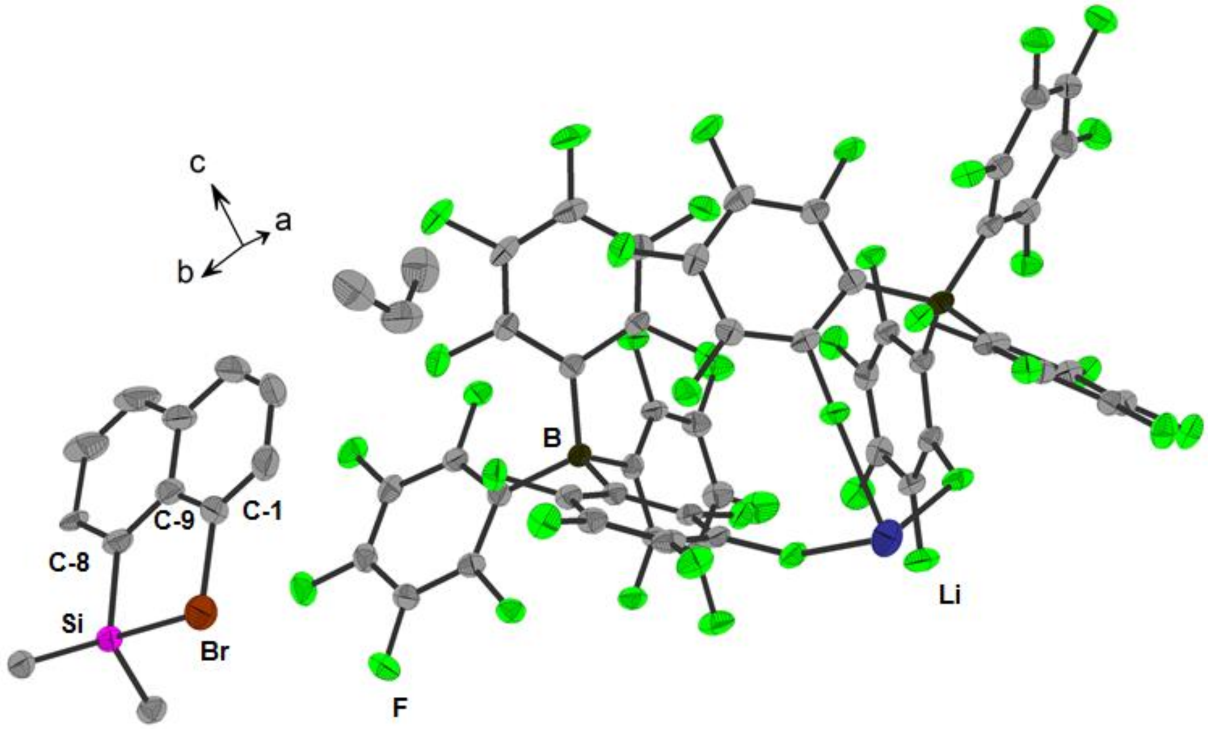

Figure S79 – Asymmetric unit of the crystal structure of **10**[B(C_6F_5)$_4$] * Li[B(C_6F_5)$_4$] * 0.5 C_6D_6 (Thermal ellipsoids are shown at the 50 % probability level. Hydrogen atoms are omitted for clarity.) Pertinent bond lengths [pm] and bond angles [°]: Br-C^1 193.1(3), Si-C^8 183.8(3), Si-Br 242.52(8), Br-C^1-C^9 115.4(2), Si-C^8-C^9 120.0(2), C^1-C^9-C^8 123.0(3), ∑α(SiC_3) 349.5, sum of bay angles ∑β 358.5.

Table S4 – Crystal data and structure refinement for **10**[B(C_6F_5)$_4$] * Li[B(C_6F_5)$_4$] * 0.5 C_6H_6 (CCDC-2023359).

Identification code	amd91	
Empirical formula	C63 H15 B2 Br F40 Li Si	
Formula weight	1668.31	
Temperature	100(2) K	
Wavelength	1.54178 Å	
Crystal system	Triclinic	
Space group	P-1	
Unit cell dimensions	a = 12.7641(4) Å	α = 86.2835(15)°.
	b = 14.0309(5) Å	β = 76.0208(14)°.
	c = 17.8695(6) Å	γ = 69.7905(15)°.
Volume	2913.70(17) Å^3	
Z	2	
Density (calculated)	1.902 Mg/m^3	
Absorption coefficient	2.777 mm^{-1}	
F(000)	1630	
Crystal size	0.070 x 0.050 x 0.030 mm^3	
Theta range for data collection	2.549 to 74.474°	
Index ranges	-15<=h<=15, -17<=k<=14, -22<=l<=22	
Reflections collected	69829	
Independent reflections	11860 (R(int) = 0.0566)	
Observed reflections (I > 2(I))	9872	
Completeness to theta = 74.474°	99.5 %	
Absorption correction	Semi-empirical from equivalents	
Max. and min. transmission	1.0000 and 0.8802	
Refinement method	Full-matrix least-squares on F^2	
Data / restraints / parameters	11860 / 0 / 975	
Goodness-of-fit on F^2	1.016	
Final R indices (I>2sigma(I))	R1 = 0.0468, wR2 = 0.1287	
R indices (all data)	R1 = 0.0574, wR2 = 0.1409	
Extinction coefficient	n/a	
Largest diff. peak and hole	0.886 and -1.398 e.Å^{-3}	

1.6.4 Data from X-ray diffraction analysis of siliconium borate $4c_2[B_{12}Br_{12}]$

Trityl borate $[Ph_3C]_2[B_{12}Br_{12}]$ (1.0 equiv, 51.5 µmol, 81 mg) and 5-bromo-6-dimethylsilylacenaphthene **1c** (6.0 equiv, 309 µmol, 90 mg) were combined in toluene at r.t. and stirred vigorously for 72 h. Then, the solution was removed and the yellow solid was washed twice with toluene and dried before 1,2-dichlorobenzene was added. A green suspension was obtained after stirring for 1 h and light green crystals of siliconium borate **4c**$_2[B_{12}Br_{12}]$ that were suitable for X-ray diffraction analysis were obtained by slow evaporation of the solvent at room temperature.

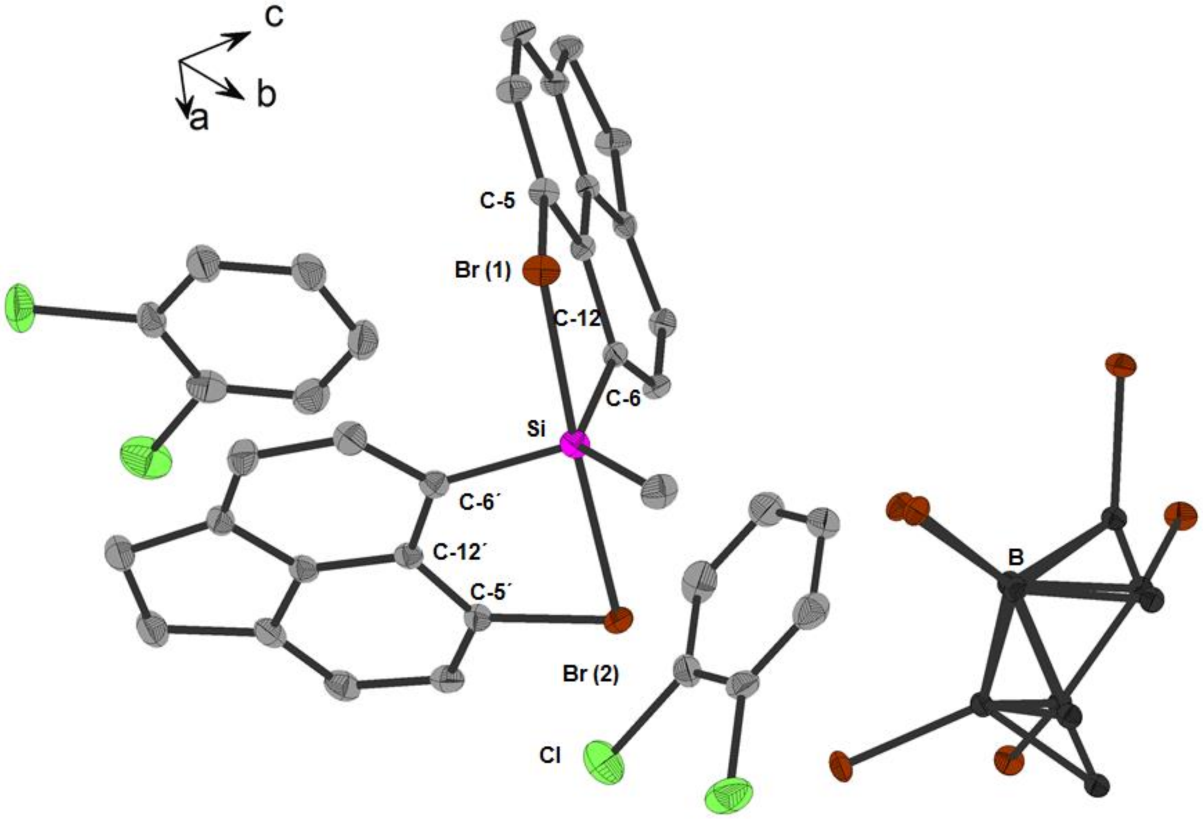

Figure S80 – Asymmetric unit of the crystal structure of **4c**$_2[B_{12}Br_{12}]$ * 3 $C_6H_4Cl_2$ (Thermal ellipsoids are shown at the 50 % probability level. Hydrogen atoms and solvent molecules are omitted for clarity.) There is disorder of one solvent molecule (50:50). Pertinent bond lengths [pm] and bond angles [°]: Br(1)-C^5 191.2(2), Br(2)-$C^{5´}$ 190.69(18), Si-C^6 186.8(2), Si-$C^{6´}$ 187.73(19), Si-Br(1) 270.00(6), Si-Br(2) 265.08(6), Br(1)-C^5-C^{12} 115.16(14), Br(2)-$C^{5´}$-$C^{12´}$ 114.98(13), Si-C^6-C^{12} 118.07(15), Si-$C^{6´}$-$C^{12´}$ 117.35(14), C^5-C^{12}-C^6 127.51(19), $C^{5´}$-$C^{12´}$-$C^{6´}$ 127.94(17), Br(1)-Si-Br(2) 176.23(2), ∑α(SiC_3) 360.0, sum of bay angles ∑β 360.8 and ∑β´ 360.3.

Table S5 – Crystal data and structure refinement for **4c**$_2$[$B_{12}Br_{12}$] * 3 $C_6H_4Cl_2$ (CCDC-2023358).

Identification code	amd80_5	
Empirical formula	C68 H50 B12 Br16 Cl6 Si2	
Formula weight	2544.24	
Temperature	100(2) K	
Wavelength	0.71073 Å	
Crystal system	Triclinic	
Space group	P-1	
Unit cell dimensions	a = 11.3404(3) Å	α = 76.4564(12)°.
	b = 12.5831(3) Å	β = 78.1190(13)°.
	c = 15.0310(4) Å	γ = 89.8238(12)°.
Volume	2038.27(9) Å^3	
Z	1	
Density (calculated)	2.073 Mg/m^3	
Absorption coefficient	8.124 mm^{-1}	
F(000)	1208	
Crystal size	0.200 x 0.100 x 0.050 mm^3	
Theta range for data collection	1.426 to 36.318°	
Index ranges	-18<=h<=18, -20<=k<=20, -25<=l<=25	
Reflections collected	43905	
Independent reflections	43905 (R(int) = ?)	
Observed reflections (I > 2(I))	33040	
Completeness to theta = 36.318°	100.0 %	
Absorption correction	Semi-empirical from equivalents	
Max. and min. transmission	1.000000 and 0.608775	
Refinement method	Full-matrix least-squares on F^2	
Data / restraints / parameters	43905 / 0 / 507	
Goodness-of-fit on F^2	1.013	
Final R indices (I>2sigma(I))	R1 = 0.0338, wR2 = 0.0638	
R indices (all data)	R1 = 0.0577, wR2 = 0.0684	
Extinction coefficient	n/a	
Largest diff. peak and hole	1.625 and -1.409 e.Å^{-3}	

2. Computational Part

All quantum chemical calculations were carried out using the Gaussian09 package.[S12] The NBO analyses were performed with the version 6.0 which was implemented in the G09 D.01 version including the natural resonance theory (NRT)[S13]. The AIMALL program was used to perform the QTAIM analysis. (T. A. Keith, AIMAll (Version 11.05.16), 2011.)

The molecular structure optimizations were performed using the M06-2X functional [S14] along with the def2tzvp basis set for the elements F, Cl, Br, I, Si, C, H and using the corresponding pseudopotential for I [S15], as well as the ultrafinegrid option. Every stationary point was identified by a subsequent frequency calculation as minimum (Number of imaginary frequencies (NIMAG): 0). The SCF energies (E(SCF)) and the computed Gibbs free energies at T = 298.15 K and p = 0.101 MPa (1 atm) in the gas phase (G^{298}) are given in Table S6 for all optimized molecular structures.

For the calculation of the mechanism of the substituent redistribution reaction (Figure 10), the Gibbs free energies, G^{298}(benzene) at T = 298.15 K in benzene were used and are given in Table S7. Therefore, the SCF energies (E(SCF)) were calculated at the M06-2X/def2tzvp level of theory with inclusion of solvent effects using the SCIPM model with benzene as solvent. In order to avoid the overestimation of entropy effects in the gas-phase, the pressure of liquid benzene (p = 28.1 MPa (277 atm) was used for the calculation.

Silicon NMR chemical shift calculations were performed using the GIAO method as implemented in the Gaussian 09 program, the M06L functional and a def2-TZVP basis set for molecular structures optimized at the M06-2X/def2tzvp level of theory. The calculated isotropic Si chemical shieldings were transferred to the TMS scale by using the calculated Si shielding for TMS (PG: T_d) obtained for the same model chemistry (σ(Si) = 350.55). The optimized molecular structures of selected compounds are given as cartesian coordinates in the attached xyz.file.

Table S6 – Calculated absolute energies, E(SCF), and free enthalpies at 298 K (0.1 MPa), G^{298}, for the compounds of interest (at M06-2X/def2 zvp). "Cation isodes" and "silane isodes" are silyl cations and silanes used for the calculation of the isodesmic reactions given in Scheme S**3**-S**5**,

**Compound**	**E(SCF) [a.u.]**	**ZPVE [kJmol^{-1}]**	**G^{298} [a.u.]**
Ace-F cation **3a**	-931.00365	631	-930.80467
Ace-F silane **1a**	-931.85331	654	-931.64597
Ace-F isodes cation **17a**	-930.99599	631	-930.79871
Ace-F isodes silane **16a**	-931.85508	653	-931.64852
Ace-Cl cation **3b**	-1291.36697	628	-1291.17012
Ace-Cl silane **1b**	-1292.19963	651	-1291.99449
Ace- Cl isodes cation **17b**	-1291.34514	627	-1291.15063

Ace-Cl isodes silane **16b**	-1292.20505	650	-1292.00079
Ace-Br cation **3c**	-3405.36322	626	-3405.16796
Ace-Br silane **1c**	-3406.19084	649	-3405.98744
Ace-Br isodes cation **17c**	-3405.33815	625	-3405.14509
Ace-Br isodes silane **16c**	-3406.19836	648	-3405.99591
Ace-I cation **3d**	-1128.80006	625	-1128.60597
Ace-I silane **1d**	-1129.62261	648	-1129.42097
Ace-I isodes cation **17d**	-1128.77320	624	-1128.58097
Ace-I isodes silane **16d**	-1129.63294	647	-1129.43156
Naph-Br cation **10**	-3327.94326	533	-3327.78120
Naph-Br silane **2**	-3328.76983	557	-3328.59947
Naph-Br isodes cation **19**	-3327.91091	533	-3327.75021
Naph-Br isodes silane **18**	-3328.78034	556	-3328.61038
$(Ace\text{-}Br)_2$ cation **4c**	-6401.74452	962	-6401.43375
$(Ace\text{-}Br)_2$ silane	-6402.55919	984	-6402.24066
$(Ace\text{-}H)_2$ cation	-1254.53462	1014	-1254.19939
$(Ace\text{-}H)_2$ silane	-1255.37806	1036	-1255.03529
Me_3Si cation	-408.94161	287	-408.86438
Me_3SiH	-409.82281	313	-409.73361
$PhFSiMe_3$ cation	-740.46710	539	-740.30350
Fluorobenzene	-331.47882	245	-331.41383
$PhClSiMe_3$ cation	-1100.82123	537	-1100,65850
Chlorobenzene	-691.82901	241	-691.76631
$PhBrSiMe_3$ cation **13**	-3214.81580	524	-3214.65531
Bromobenzene	-2805.82228	239	-2805.76127
$PhISiMe_3$ cation	-938.25141	533	-938.09235
Iodobenzene	-529.25688	238	-529.19710
acenaphthene	-463.27512	482	-463.12486
naphthalene	-385.85970	390	-385.74230
areniumion **14**	-6811.56228	1284	-6811.13600
areniumion_1,2-Si shift **15**	-6811.55520	1284	-6811.12934
$AceSiMe_2Br$ **12**	-3406.26049	656	-3406.05475

Table S7 – Calculated absolute energies, E(SCF) and free enthalpies at 298 K (28.1 MPa), G^{298}(benzene), in benzene for the compounds of interest (at M06-2X/def2tzvp).

**Compound**	**E(SCF) [a.u.]**	**G^{298}(benzene) [a.u.]**
Ace-Br cation **3c**	-3405.39875	-3405.22523
Ace-Br silane **1c**	-3406.19283	-3406.01215
areniumion **14**	-6811.59276	-6811.21703
areniumion_1,2-Si shift **15**	-6811.58631	-6811.21098
$(Ace\text{-}Br)_2$ cation **4c**	-6401.77399	-6401.49969
Me_3SiH	-409.82316	-409.74200

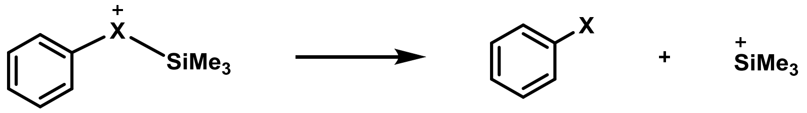

Scheme S3 – Isodesmic reaction for the calculation of the bond dissoziation energies (BDE).

Table S8 – Calculated bond dissociation energies (BDE) $kJmol^{-1}$ according to Scheme S**3**.

	**BDE (Si-X) [$kJmol^{-1}$]**
X = F	123
X = Cl	133
X = Br	136
X = I	139

The bond dissociation energy for the newly formed silicon – halogen bond is estimated using the isodesmic equations shown in Scheme S4 and the results are given in Table S9. These isodesmic equations are not ideal for two reasons: silanes **1**, **2** are destabilized compared to their isomers **16**, **18** by the *peri*-disubstitution. This leads to the prediction of too strong silicon – halogen bonds in cations **3** and **10** by the isodesmic equations. In addition, the conformation of the $SiMe_2$ units in cations **17**, **19** relative to the naphthalene / acenaphthene backbone allow significant conjugation between the 3p(Si) orbital and the π-system of the arene unit, which is not possible in the chalcogenyl stabilized cations **3**, **10**. This latter imbalance of the isodesmic equations leads to the prediction of too weak Ch – Si bonds. These opposing and in consequence cancelling effects in mind, we suggest that the calculated reaction enthalpies of the isodesmic equations that are shown in Scheme S4 are a good first approximation for the strength of the silicon – halogen linkage in cations **3** and **10**.

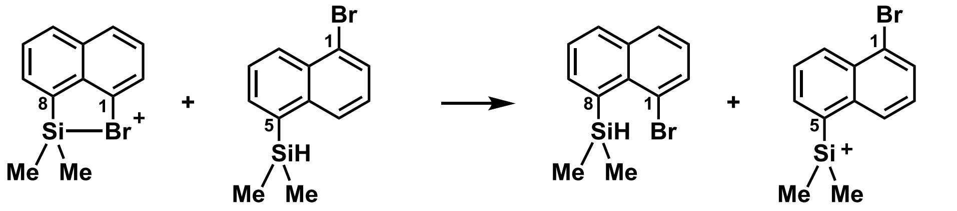

10 **18** **2** **19**

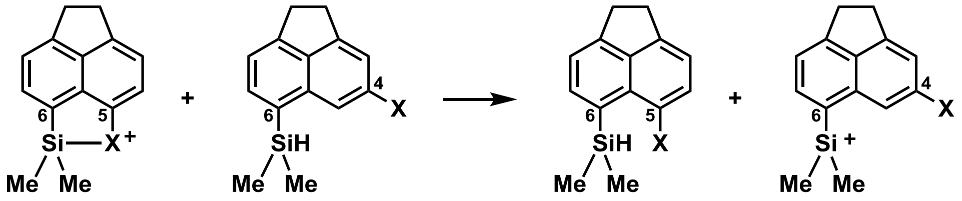

3 **16** **1** **17**

Scheme S4 – Isodesmic reaction for the calculation of the bond dissociation energys (BDE) of the halonium ions (acenaphthyl (ace), naphthyl (naph), X = F, Cl, Br, I).

Table S9 – Calculated bond dissociation energies (BDE) of the halonium ions in kJ mol^{-1} according to Scheme **S4** (acenaphthyl (ace), naphthyl (naph)).

	**BDE (Si-X) [$kJmol^{-1}$]**
Ace, X = F	25
Ace, X = Cl	72
Ace, X = Br	86
Ace, X = I	98
Naph, X = Br	113

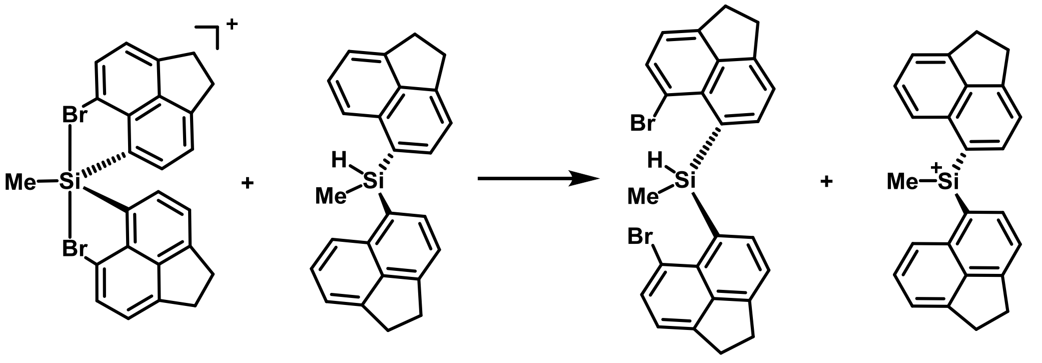

$(Ace\text{-}Br)_2$ cation **4c** **$(Ace\text{-}H)_2$ silane** **$(Ace\text{-}Br)_2$ silane** **$(Ace\text{-}H)_2$ cation**

Scheme S5 – Isodesmic reaction for the calculation of the bond dissociation energy (BDE = 76 $kJmol^{-1}$) of the siliconium ion **4c**.

3. References

[S1] W. D. Neudorff, D. Lentz, M. Anibarro, A. D. Schlüter, *Chem. Eur. J.* **2003**, *9*, 2745.

[S2] N. Lühmann, *Dissertation*, Carl von Ossietzky University Oldenburg **2011**.

[S3] S. Künzler, S. Rathjen, A. Merk, M. Schmidtmann, T. Müller, *Chem. Eur. J.* **2019**, *25*, 15123.

[S4] N. Lühmann, H. Hirao, S. Shaik, T. Müller, *Organometallics* **2011**, *30*, 4087.

[S5] A. G. Massey, A. J. Park, *J. Organomet. Chem.* **1964**, *2*, 245; J. C. W. Chien, W. M. Tsai, M. D. Rausch, *J. Am. Chem. Soc.* **1991**, *113*, 8570.

[S6] N. Tanaka, T. Kasai, *Bull. Chem. Soc. Jpn*. **1981**, *54*, 3020.

[S7] L. Krause, R. Herbst-Irmer, G. M. Sheldrick, D. Stalke, *J. Appl. Cryst.* **2015**, *48*, 3-10)

[S8] G. M. Sheldrick (**2012**), *TWINABS* 2012/1. Bruker, Madison, Wisconsin, USA.

[S9] G. M. Sheldrick, *Acta Cryst.* **2008**, *A64*, 112-122.

[S10] G. M. Sheldrick, *Acta Cryst.* **2015**, *C71*, 3-8.

[S11] O. V. Dolomanov, L. J. Bourhis, R. J. Gildea, J. A. K. Howard, H. Puschmann, J*. Appl. Cryst.* **2009**, *42*, 339-341.

[S12] *Gaussian 09*, *Revision D.01*, M. J. Frisch, G. W. Trucks, H. B. Schlegel, G. E. Scuseria, M. A. Robb, J. R. Cheeseman, G. Scalmani, V. Barone, B. Mennucci, G. A. Petersson, H. Nakatsuji, M. Caricato, X. Li, H. P. Hratchian, A. F. Izmaylov, J. Bloino, G. Zheng, J. L. Sonnenberg, M. Hada, M. Ehara, K. Toyota, R. Fukuda, J. Hasegawa, M. Ishida, T. Nakajima, Y. Honda, O. Kitao, H. Nakai, T. Vreven, J. A. Montgomery, Jr., J. E. Peralta, F. Ogliaro, M. Bearpark, J. J. Heyd, E. Brothers, K. N. Kudin, V. N. Staroverov, T. Keith, R. Kobayashi, J. Normand, K. Raghavachari, A. Rendell, J. C. Burant, S. S. Iyengar, J. Tomasi, M. Cossi, N. Rega, J. M. Millam, M. Klene, J. E. Knox, J. B. Cross, V. Bakken, C. Adamo, J. Jaramillo, R. Gomperts, R. E. Stratmann, O. Yazyev, A. J. Austin, R. Cammi, C. Pomelli, J. W. Ochterski, R. L. Martin, K. Morokuma, V. G. Zakrzewski, G. A. Voth, P. Salvador, J. J. Dannenberg, S. Dapprich, A. D. Daniels, O. Farkas, J. B. Foresman, J. V. Ortiz, J. Cioslowski, and D. J. Fox, Gaussian, Inc., Wallingford CT, 2013.

[S13] a) E. D. Glendening, F. Weinhold, *J. Comput. Chem.* **1998**, *19*, 593; b) E. D. Glendening, F. Weinhold, *J. Comput. Chem.* **1998**, *19*, 610; c) E. D. Glendening, J. K. Badenhoop, F. Weinhold, *J. Comput. Chem.* **1998**, *19*, 628.

[S14] Y. Zhao, D. Truhlar, *Theor. Chem. Acc.* **2008**, 120, 215.

[S15] a) D. Feller, *J. Comput. Chem.* **1996**, *17*, 1571; b) K. A. Peterson, D. Figgen, E. Goll, H. Stoll, M. Dolg, *J. Chem. Phys.* **2003**, *119*, 11113; c) F. Weigend, R. Ahlrichs, *Phys. Chem. Chem. Phys.* **2005**, *7*, 3297; d) K. L. Schuchardt, B. T. Didier, T. Elsethagen, L. Sun, V. Gurumoorthi, J. Chase, J. Li, T. L. Windus, *J. Chem. Inf. Model.* **2007**, *47*, 1045; e) B. P. Pritchard, D. Altarawy, B. Didier, T. D. Gibson, T. L. Windus, *J. Chem. Inf. Model.* **2019**, *59*, 4814.
